# Different Modes of Acid-Promoted Cyclooligomerization of 4-(4-Thiosemicarbazido)butan-2-one Hydrazone: 14-Membered versus 28-Membered Polyazamacrocycle Formation

**DOI:** 10.1021/acs.joc.2c01199

**Published:** 2022-11-16

**Authors:** Anastasia A. Fesenko, Mikhail S. Grigoriev, Vladimir B. Arion, Anatoly D. Shutalev

**Affiliations:** † N. D. Zelinsky Institute of Organic Chemistry, Russian Academy of Sciences, 47 Leninsky Avenue, 119991 Moscow, Russian Federation; ‡ Frumkin Institute of Physical Chemistry and Electrochemistry, Russian Academy of Sciences, 31 Leninsky Avenue, Bldg 4, 119071 Moscow, Russian Federation; § Institute of Inorganic Chemistry of the University of Vienna, Währinger Strasse 42, 1090 Vienna, Austria

## Abstract

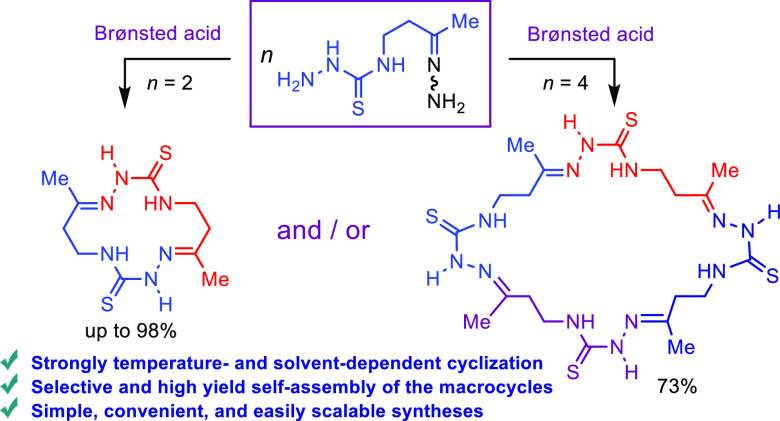

Unprecedented self-assembly of a novel 14-membered cyclic bis-thiosemicarbazone or/and a 28-membered cyclic tetrakis-thiosemicarbazone upon acid-promoted cyclooligomerization of 4-(4-thiosemicarbazido)butan-2-one hydrazone has been discovered. A thorough study of the influence of various factors on the direction of macrocyclization provided the optimal conditions for the highly selective formation of each of the macrocycles in excellent yields. Plausible pathways for macrocyclizations have been discussed. The macrocycle precursor was prepared by the reaction of readily available 4-isothiocyanatobutan-2-one with an excess of hydrazine.

## Introduction

Polyaza macrocycles (PAMs) are of considerable importance in various fields of chemistry, biochemistry, medicine, and material science. They are the constituents of various naturally occurring organic substances, including vitamin B_12_, chlorophyll, metalloproteins, cyclic peptides, etc. A unique feature of PAMs is their ability to bind different inorganic and organic cations, anions, and neutral molecules.^1,2^ PAMs and their metal complexes exhibit a broad spectrum of biological activities^3^ including anticancer,^4^ anti-HIV,^5^ antibacterial, and antifungal properties.^6^ The metal complexes are also used as radiopharmaceuticals,^7^ MRI contrast agents,^8^ NMR shift reagents,^9^ luminescent materials,^9b,10^ sensors,^10,11^ catalysts,^12^ etc.

Due to the great interest in the chemistry and applications of PAMs, a huge number of these heterocycles have been synthesized to date. Among them, 14-membered PAMs with the N_4_ binding site (cyclames, cyclic Shiff bases, etc.) are of special importance.^1^ At the same time, tetradentate 14-membered 1,2,4,8,9,11-hexaaza macrocycles remain practically unknown. Only a few polyunsaturated representatives of these heterocycles or Ni(II)-complexes have been described.^13^ Therefore, the development of general approaches to 14-membered 1,2,4,8,9,11-hexaaza macrocycles and investigation of their structure-binding ability relationships are of significance.

**Scheme 1:**
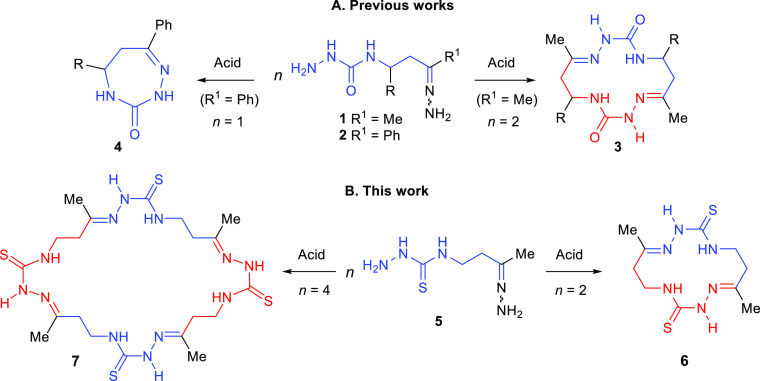
Synthesis of Cyclic Semicarbazones and Thiosemicarbazones

Recently, we discovered unprecedented self-assembly of novel 14-membered cyclic bis-semicarbazones, 1,2,4,8,9,11-hexaazacyclotetradeca-7,14-diene-3,10-diones **3**,^14^ upon acid-promoted dimerization of hydrazones of 4-(1-aryl-3-oxobut-1-yl)semicarbazides **1** (Scheme 1A) prepared in 4 steps from ethyl carbamate^14b^ or from semicarbazones of aromatic aldehydes.^15^ It is noteworthy that, under similar conditions, close analogues of **1**, hydrazones of 4-(1,3-diaryl-3-oxoprop-1-yl)semicarbazides **2**, gave only 7-membered cyclic semicarbazones **4**,^14b^ which indicates a dramatic influence of the substrate structure on the outcome of cyclization.

The route to macrocycles **3** is very simple, flexible, and easy to be scaled up. The prepared compounds were found to be able to chelate various metal cations through the N1, N4, N8, and N11 atoms.^16^ Compared with other described 14-membered 1,2,4,8,9,11-hexaazamacrocycles,^13^ compounds **3** are conformationally more flexible, providing a rather dynamic binding cavity. However, these ligands have some drawbacks such as their extremely low solubility in common solvents^15^ and rather limited possibilities for their modifications to change metal binding properties. To date, only N2,N9-dibutylated derivatives of two macrocycles **3** were prepared.^16a^

In continuation of our research on PAMs, we were interested in the synthesis of unknown dithioxo-analogues of macrocycles **3**, 14-membered cyclic bis-thiosemicarbazones (e.g., **6**, Scheme 1B). We expected that their host–guest binding properties could significantly differ from those of compounds **3** due to differences in the electronic structures of thioamides and amides,^17^ particularly, thiosemicarbazones and semicarbazones. Some of these differences arise from greater charge transfer from nitrogen atoms to the C=S bond in thioamides than to the C=O bond in amides. As a result, thioamides are stronger NH acids (Δp*K*a ∼6–7) than amides,^18^ and NH groups in thioamides are better hydrogen bond donors than in amides.^19^ The presence of thioamide groups in macrocyclic bis-thiosemicarbazones provides great opportunities for further modifications of these compounds aimed at the tuning of their binding properties. It is well-known that thioamides are significantly more reactive than amides toward various electrophilic and nucleophilic reagents, oxidants, reductants, etc.^17a^ In addition, we hoped that the target macrocyclic thiosemicarbazones would be more soluble in organic solvents than their oxo-analogues **3**.

Initially, we attempted to synthesize macrocyclic bis-thiosemicarbazones by thionation of 2,9-dibutyl-substituted *trans*-**3** (R = Ph or 4-MeOC_6_H_4_) with Lawesson’s reagent or P_2_S_5_. However, under all tested conditions, only deep decomposition of the starting material was observed. We hypothesized that an alternative synthesis could involve acid-promoted cyclization of thioxo-analogues of hydrazones **1** (e.g., **5**, Scheme 1B). However, in contrast to the preparation of **1** from ethyl carbamate,^14b^ the synthesis of their thioxo-analogues from ethyl thiocarbamate failed.^20^ Another approach to hydrazones of 4-(3-oxoprop-1-yl)thiosemicarbazides could be based on the reaction of 4-isothiocyanatobutan-2-ones with hydrazine. The initially formed products of this reaction, 4-(4-thiosemicarbazido)butan-2-ones, are known to cyclize spontaneously to the corresponding 3-amino-4-hydroxyhexahydropyrimidine-2-thiones.^21^ However, due to the ring-chain isomerism, the pyrimidines could be expected to react with an excess of hydrazine to form the target hydrazones.

Herein, we report on the synthesis of unsubstituted 4-(3-oxobutyl)thiosemicarbazide hydrazone **5** and its acid-promoted cyclooligomerization, which, depending on the reaction conditions, afforded previously unknown 14- and 28-membered cyclic bis- and tetrakis-thiosemicarbazones **6** and **7** (Scheme 1B). A plausible explanation of the data obtained is as follows.

## Results and Discussion

**Scheme 2:**
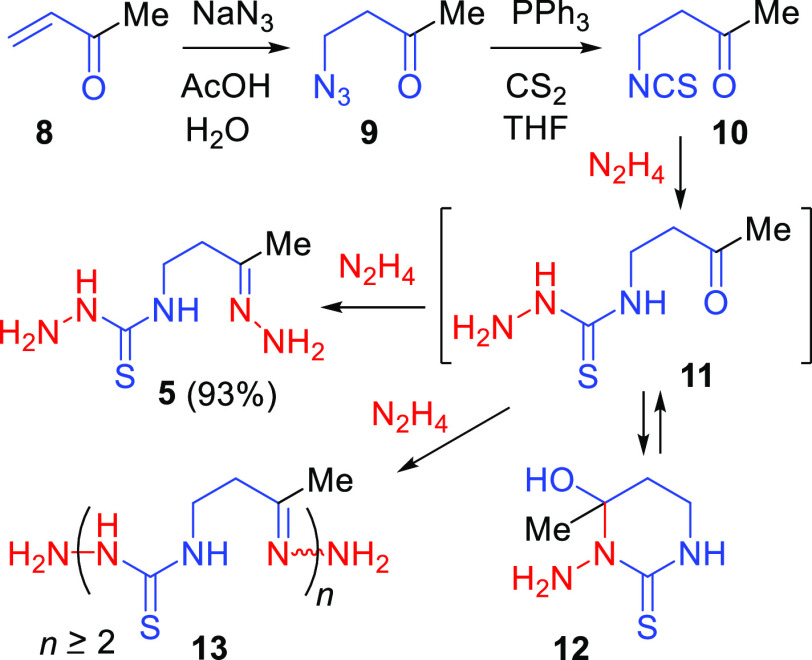
Synthesis of 4-(3-Oxobutyl)thiosemicarbazide Hydrazone (5)

The starting material, 4-isothiocyanatobutan-2-one (**10**), was prepared according to our regioselective procedure^22^ by the addition of HN_3_ to methyl vinyl ketone (**8**) followed by the reaction of the obtained azidoketone **9** with CS_2_ and PPh_3_ in 40% overall yield after vacuum distillation (Scheme 2).

We studied the reaction of isothiocyanato ketone **10** with N_2_H_4_·H_2_O (1.02–1.50 equiv) under various conditions (H_2_O, EtOH, or MeCN, room temperature, 1–24 h). However, in contrast to the previously reported reaction of other β-isothiocyanato ketones with N_2_H_4_·H_2_O,^21,23^ compound **10** failed to give pyrimidine **12** or/and its acyclic form **11**. According to ^1^H NMR spectra, under all tested conditions, only mixtures of numerous products **13** arising from oligomerization of the initially formed thiosemicarbazide **11** were obtained. The characteristic feature of these spectra was the presence of a set of singlet signals in the range of 9.78–10.36 ppm, which can be assigned to the NH proton in different N–C(*S*)–NH–N=C moieties. In addition, the CH_3_ group protons are observed in the 1.76–2.00 ppm region indicating the presence of various CH_3_C=N fragments. For example, the product prepared by the reaction of **10** with 1.01 equiv of N_2_H_4_·H_2_O (H_2_O, rt, 24 h) showed a number of thiosemicarbazide NH_2_ groups, which was about 6 times less than the number of CH_3_ groups.

Since the synthesis of compounds **11** and **12** failed, we attempted to prepare the macrocycle precursor, hydrazone **5**, directly from isothiocyanate **10** by using a large excess of hydrazine to suppress the oligomerization. Gratifyingly, compound **5** as a 92:8 mixture of (*E*)- and (*Z*)-isomers was synthesized in 93% yield by slow addition of a solution of **10** in EtOH to a solution of N_2_H_4_·H_2_O (10 equiv) in EtOH at room temperature under stirring.

The stereochemical assignments for **5** were based on the comparison of the experimental chemical shifts of the aliphatic carbons (DMSO solution) in the NCH_2_CH_2_C(=NNH_2_)CH_3_ moiety for the major isomer of **5** (40.2, 37.7, and 14.2 ppm, respectively) and the minor isomer of **5** (38.5, 29.0, and 22.9 ppm, respectively) with those calculated by the GIAO method at the WC04/6-311+G(2d,p) level of theory using the density functional theory (DFT) B3LYP/6-311++G(d,p) optimized geometries (DMSO solution) for both (*E*)-**5** (40.1, 38.7, and 14.1 ppm, respectively) and (*Z*)-**5** (37.5, 28.4, and 24.2 ppm, respectively). In addition, the ^1^H,^1^H NOESY experiment in DMSO-d_6_ showed that, for the major isomer of **5**, a diagnostic NOE was observed between the CH_3_ and C=NNH_2_ protons, thus indicating the (*E*)-configuration of the C=N double bond.

**Scheme 3:**
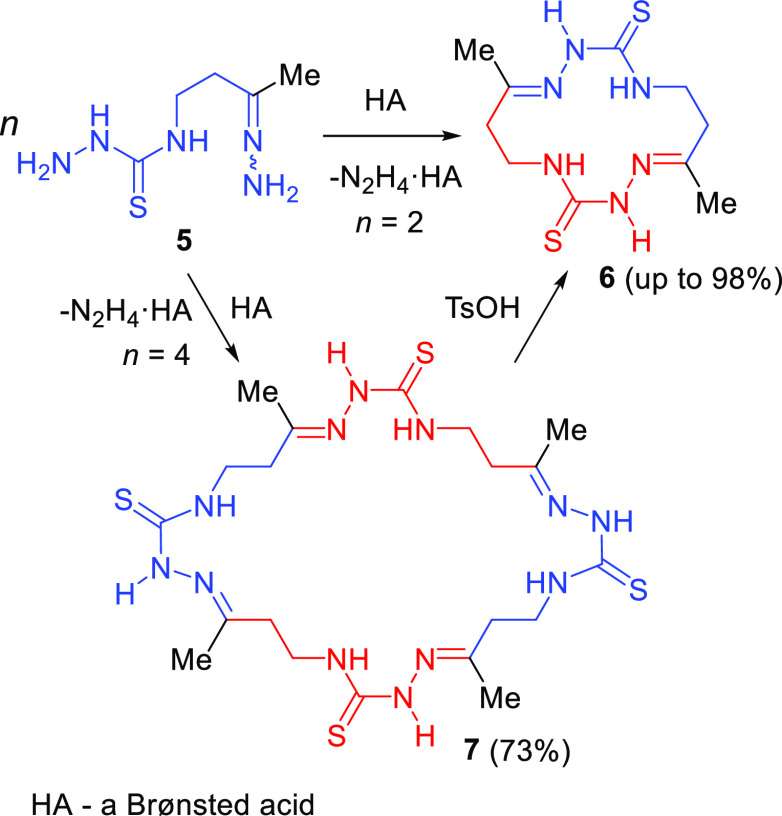
Acid-Promoted Cyclization of Hydrazone 5

Next, we studied the acid-promoted macrocyclization of hydrazone **5** with a loading of about 1 mmol under different conditions varying catalyst identity and amount, solvent, concentration of **5**, reaction time, and temperature. In these experiments, reaction flasks were charged either with **5** and solid acid TsOH·H_2_O followed by the addition of a solvent, or with **5** and a solvent followed by the addition of a liquid acid. The reaction mixtures were stirred at a certain temperature, and the precipitated macrocyclic product(s) was (were) isolated in high yields by removal of solvent, treatment with aq NaHCO_3_, and filtration. In general, under the conditions applied, hydrazone **5** was converted either into 14-membered cyclic bis-thiosemicarbazone **6** or into mixtures of **6** with 28-membered cyclic tetrakis-thiosemicarbazone **7** in different ratios (Scheme 3; for specific data, see Table 1).

**Table 1:** Acid-Promoted Cyclooligomerization of Hydrazone **5** (about 1 mmol Loading) To Give Macrocycles **6** and **7**

entry	solvent	promotor	equiv of the promotor	conc. of **5** (mmol/mL)	reaction conditions	product(s)	molar ratio of **6**/**7**	yield (%)^a^
1	MeCN	TsOH	1.10	0.20	reflux, 30 min	**6** + **7**	94:6	89
2	MeCN	TsOH	1.10	0.19	reflux, 2 h	**6**		93
3	MeCN	TsOH	0.09	0.23	reflux, 2 h	**6**		b
4	MeCN	TsOH	1.10	0.50	rt, 8 h	**6** + **7**	33:67	c
5	MeCN	TsOH	1.11	0.20	rt, 24 h	**6** + **7**	33:67	d
6	MeCN	TFA	1.49	0.21	reflux, 2 h	**6**		86
7	EtOH	TsOH	1.10	0.20	reflux, 30 min	**6** + **7**	75:25	87
8	EtOH	TsOH	1.10	0.20	reflux, 2 h	**6** + **7**	92:8	86
9	EtOH	TsOH	1.10	0.22	reflux, 8 h	**6**		94
10	EtOH	TsOH	1.09	0.25	rt, 4 h	**6** + **7**	27:73	95
11	EtOH	TsOH	1.11	0.25	rt, 8.08 h	**6** + **7**	31:69	97
12	EtOH	TsOH	1.11	0.07	rt, 8 h	**6** + **7**	29:71	94
13	EtOH	TsOH	1.10	0.50	rt, 8 h	**6** + **7**	24:76	97
14	EtOH	TsOH	1.11	0.19	rt, 24 h	**6** + **7**	34:66	92
15	EtOH	TsOH	1.10	0.51	ice bath, 7 h	**6** + **7**	12:88	91
16	EtOH	TsOH	1.10	0.51	–14 to −5 °C, 1.33 h, then ice bath, 7 h	**6** + **7**	9:91	96
17	EtOH	TsOH + N_2_H_4_·TsOH	1.11 + 1.00	0.26	rt, 8.17 h	**6** + **7**	32:68	97
18	EtOH	TFA	1.49	0.21	reflux, 2 h	**6** + **7**	66:34	90
19	EtOH	HCl	1.49	0.22	rt, 8 h	**6** + **7**	27:73	e
20	EtOH	TfOH	1.10	0.20	rt, 8 h	**6** + **7**	27:73	e
21	MeOH	TsOH	1.10	0.21	rt, 8 h	**6** + **7**	22:78	93
22	MeOH	TsOH	1.10	0.99	rt, 8 h	**6** + **7**	29:71	96
23	MeOH	TsOH	1.60	0.20	rt, 8 h	**6** + **7**	34:66	75
24	MeOH	TsOH	1.62	0.69	rt, 8 h	**6** + **7**	54:46	84
25	MeOH	TsOH	1.11	0.20	rt, 24 h	**6** + **7**	24:76	94
26	MeOH	TsOH + N_2_H_4_·TsOH	1.11 + 1.00	0.68	rt, 24 h	**6** + **7**	32:68	97
27	MeOH	TFA	1.51	0.20	rt, 8 h	**6** + **7**	30:70	d
28	MeOH	TFA	3.01	0.21	rt, 8 h	**6** + **7**	36:64	d
29	THF	TsOH	1.11	0.20	reflux, 2 h	**6**		81
30	MeOH–H_2_O, 1:1	TsOH	1.11	0.20	rt, 24 h	**6** + **7**	42:58	c
31	H_2_O	TsOH	1.13	0.20	reflux, 1 h	**6** + **7**	97:3	f
32	H_2_O	TsOH	1.11	0.20	rt, 8 h	**6** + **7**	65:35	c

a Isolated yields.

b Level of conversion of hydrazone **5** is 6%.

c Significant amount (>50%) of unidentified by-products was also formed.

d Significant amount (40–50%) of unidentified by-products was also formed.

e About 15% of unidentified by-products was also formed.

f About 20% of unidentified by-products was also formed.

We found out that solely 14-membered macrocycle **6** is formed by refluxing **5** in aprotic solvents (MeCN or THF) for 2 h in the presence of excess TsOH (1.10–1.11 equiv) or TFA (1.49 equiv) (entries 2, 6, and 29). This compound was obtained in up to 93% isolated yield and with >98% purity according to ^1^H NMR data. Under similar conditions (TsOH or TFA, reflux, 2 h), but in EtOH as a protic solvent, mixtures of macrocycles **6** and **7** in a ratio of 92:8 or 66:34 were prepared (entries 8 and 18). However, after prolonged reflux (8 h) in EtOH in the presence of TsOH (1.10 equiv) pure **6** was isolated in a 94% yield (entry 9). It is noteworthy that the use of 0.09 equiv of TsOH (MeCN, reflux, 2 h) led to a significant decrease of the conversion rate of **5** (entry 2 vs entry 3).

An unprecedented formation of unique 28-membered macrocycle **7** upon acid-promoted cyclization of hydrazone **5** prompted us to explore the influence of reaction conditions on the yield of **7**. The obtained data showed that the amount of **7** increased with reducing the reaction time (entry 2 vs entry 1; entry 9 vs entry 8 vs entry 7) and, especially, temperature (entries 1–2 vs entry 5; entries 7–9 vs entries 10–11 vs entry 15 vs entry 16; entry 32 vs entry 31), when replacing an aprotic solvent by a protic one (entries 2 and 29 vs entry 8; entry 4 vs entry 13; entry 5 vs entry 25). A concentration of **5** had a minor effect on the amount of **7** both in EtOH (entry 12 vs entry 13) and in MeOH (entry 21 vs entry 22; entry 23 vs entry 24). Greater excess of the catalyst decreased the amount of **7** (entry 21 vs entry 23; entry 27 vs entry 28). An additive of N_2_H_4_·TsOH as a likely templating agent in TsOH-promoted cyclization of **5** at room temperature had no effect on the macrocycles ratio in EtOH (entry 11 vs entry 17) and a minor effect in MeOH (entry 25 vs entry 26).

Thus, the optimized conditions to prepare 28-membered macrocycle **7** involve the addition of **5** to a solution of TsOH·H_2_O in EtOH at −14 °C (ice/salt bath) followed by stirring at temperatures from −14 to −5 °C for 80 min, and then at 0 °C for 7 h. As a result, a 91:9 mixture of macrocycles **7** and **6** was isolated in 96% yield (entry 16). Crystallization of this mixture from dimethylformamide (DMF) gave **7** in analytically pure form.

Conversion of **5** to macrocycles **6** and **7** requires the use of strong acid catalysts, of which TsOH was found to be the best choice, especially at room temperature (entry 11 vs entries 19 and 20; entry 23 vs entry 27). Treatment of hydrazone **5** with AcOH (1.07 equiv) in EtOH at room temperature for 8 h left the substrate practically intact. However, reflux of **5** with AcOH (1.25 equiv) in EtOH for 2 h gave only a mixture of oligomerization products similar to those obtained by the reaction of isothiocyanate **10** with 1.02–1.50 equiv of N_2_H_4_·H_2_O (vide supra). Interestingly, slow oligomerization of **5** occurred in refluxing EtOH even in the absence of any acid catalyst (about 13% of oligomers after 1 h).

The data collected in Table 1 clearly show that the 28-membered macrocycle **7**, initially arising via oligomerization-cyclization of **5**, can convert into the 14-membered macrocycle **6** under particular reaction conditions. Indeed, refluxing **5** in EtOH with TsOH (1.10 equiv) for 30 min, 2 h, and 8 h afforded the products containing 25, 8, and 0% of macrocycle **7**, respectively, in 86–94% isolated yields (entries 7–9). Next, we explored the possibility of interconversion between the obtained macrocycles under various conditions. ^1^H NMR experiments showed that upon heating DMSO-d_6_ solutions of **6**, **7**, or **6** + **7** (1:1) in NMR tubes in the temperature range of 83–135 °C for 35–120 min, no transformations occurred. Reflux of a 50:50 mixture of **6** and **7** in *n*-BuOH for 5 h changed the ratio to 68:32 indicating a slow transformation of **7** into **6** under alcoholysis conditions. The acidic catalyst, TsOH, accelerated this conversion, especially in an aprotic solvent. Indeed, reflux of **7** in EtOH with 0.11 equiv of TsOH·H_2_O for 4 h or with 1.12 equiv of TsOH·H_2_O for 5 h delivered mixtures of **7** and **6** in 94:6 or 80:20 ratio, respectively. Treatment of **7** with TsOH·H_2_O (0.30 equiv) and TsOH·N_2_H_4_ (2.00 equiv) in EtOH (reflux, 5 h) afforded a 79:21 mixture of **7** and **6**. Refluxing a 94:6 mixture of **7** and **6** in the presence of TsOH (1.10 equiv) in MeCN for 5 h resulted in a mixture of **7** and **6** in a 34:66 ratio.

**Scheme 4:**
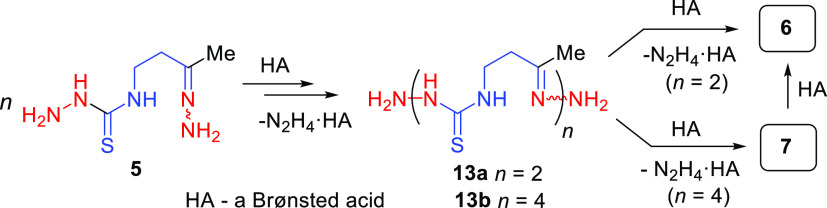
Plausible Pathways for the Acid-Promoted Transformation of 5 into Macrocycles 6 or/and 7

Thus, we assume that the acid-promoted transformation of **5** proceeds through its dimerization to give **13a**, which then further dimerizes to form **13b**. Cyclizations of the dimer and the tetramer generate macrocycles **6** and **7**, respectively (Scheme 4).

**Figure 1:**
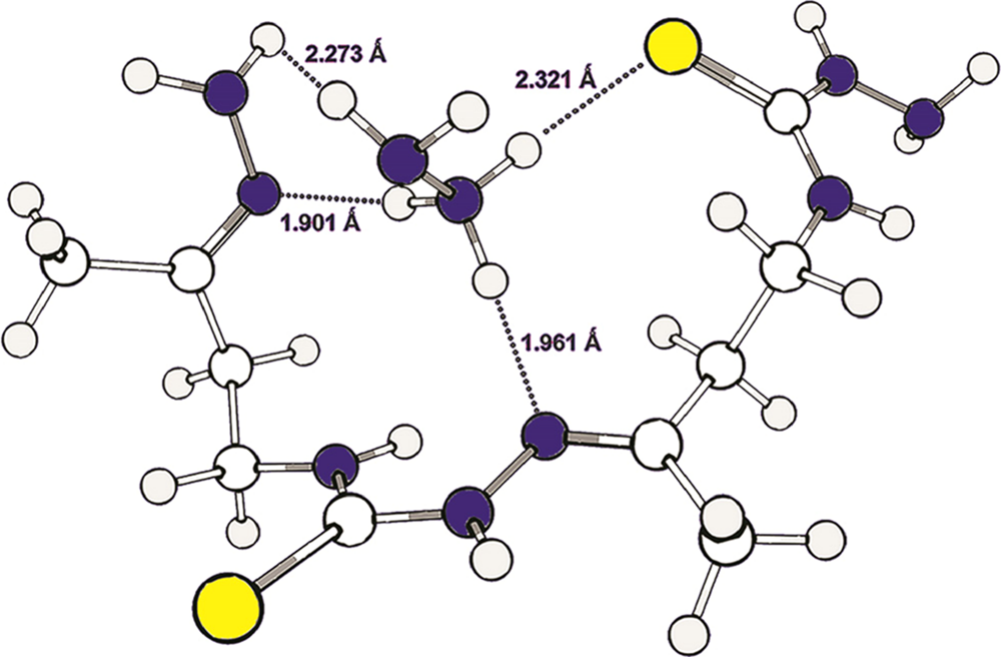
Calculated structure of the cation of complex **16** in DMSO solution.

**Scheme 5:**
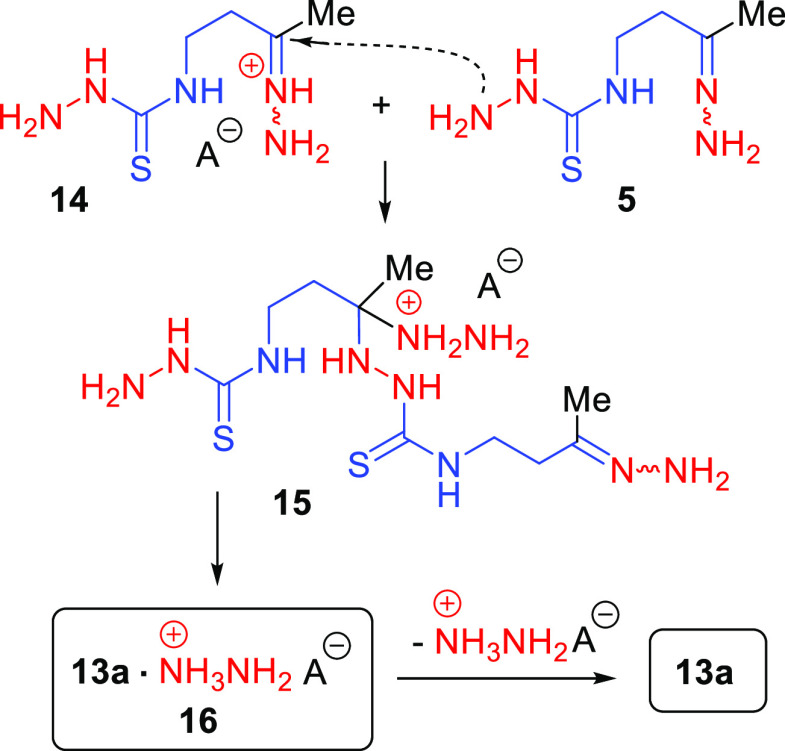
Pathway for the Acid-Promoted Dimerization of Hydrazone 5

To explain the huge effect of the reaction conditions on the ratio **6**/**7**, the DFT B3LYP/6–311++G(d,p) calculations were performed. Optimized geometries for various conformers of hydrazone **5**, macrocycles **6** and **7**, as well as for some key intermediates were calculated for solutions in DMSO and EtOH using the polarizable continuum model (PCM) model. The formation of dimer **13a** starts with the activation of **5** by protonation at the imine nitrogen with a Brønsted acid (HA) to give salt **14** (Scheme 5). This protonation is energetically the most favorable compared with the protonation at other possible sites. Nucleophilic attack of the thiosemicarbazide NH_2_ group in **5** on the carbon atom of the protonated imino group of **14** provides one of the intermediates, compound **15**. The two-step elimination of the hydrazinium cation from the latter results in a complex of dimer **13a** with this cation, compound **16**. In this complex, the hydrazinium cation located in the cavity of the dimer is linked by at least three hydrogen bonds with donor atoms (Figure 1). As a result, the possibilities of intramolecular cyclization of **16** to form a 14-membered macrocycle **6** are blocked. Thus, at low temperatures, dimerization of **16** takes place, followed by cyclization of the intermediate tetramer **13b** to 28-membered macrocycle **7**. An increase in the reaction temperature, for example, boiling in EtOH or MeCN, promotes the destruction of complex **16** and cyclization of free dimer **13a** into 14-membered macrocycle **6**.

**Figure 2:**
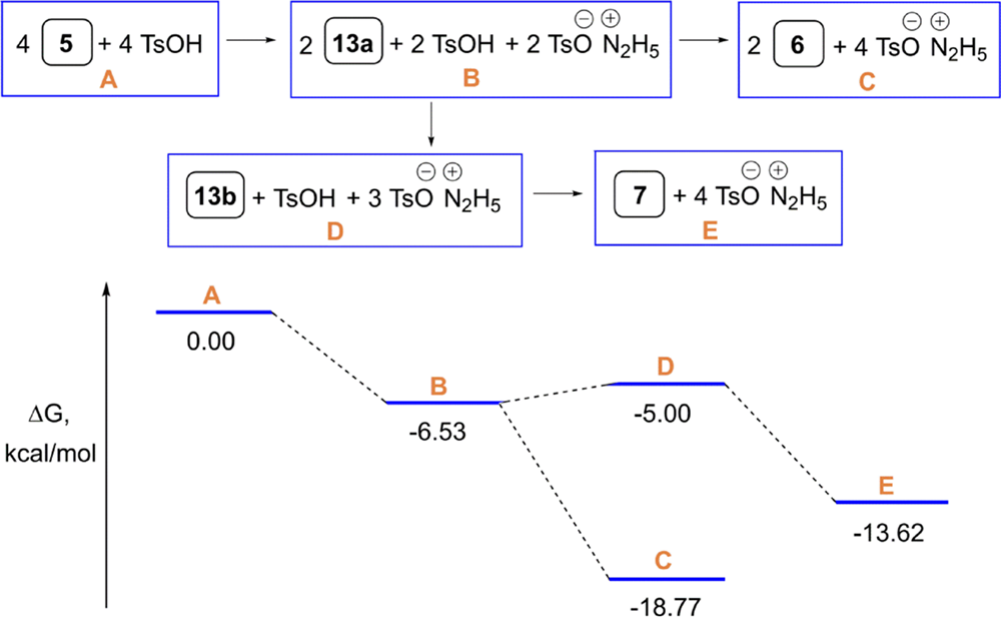
Gibbs free energy diagram [B3LYP/6-311++G(d,p)] for the TsOH-promoted transformation of hydrazone **5** into macrocycles **6** and **7** in EtOH solution. Free energies in kcal/mol at 298 K and 1 atm.

The DFT B3LYP/6-311++G(d,p) calculations were also performed to estimate thermodynamic parameters for the TsOH-promoted transformation of hydrazone **5** (EtOH solution) into dimer **13a** followed by the conversion of the latter to either macrocycle **6** or tetramer **13b** and then macrocycle **7**. Relative Gibbs free energies of the starting (**A**), final (**C** and **E**), and intermediate (**B** and **D**) molecular systems (Figure 2) were calculated using the Gibbs free energies for the most stable conformers of hydrazone (*E*)-**5**, macrocycles **6** and **7**, dimer (*E*,*E*)-**13a**, tetramer (*E*,*E*,*E*,*E*)-**13b**, TsOH, and hydrazonium tosylate.

Figure 2 shows that the reaction of (*E*)-**5** with TsOH in EtOH is a thermodynamically favorable process. Notably, the formation of 14-membered macrocycle **6** is more advantageous than the formation of 28-membered macrocycle **7**. However, it should be underlined that this reaction actually proceeds under heterogeneous conditions (vide supra) and macrocycles **6** and **7** rapidly begin to precipitate after mixing the reactants in the solvent. Clearly, the heterogeneous nature of the reaction dramatically changes its thermodynamic characteristics, in particular, the macrocyclization becomes significantly more favorable.

As mentioned previously, the data summarized in Table 1 were obtained with about 1 mmol loading of hydrazone **5** (0.18 ± 0.04 g). Disappointingly, attempts to prepare solely macrocycle **6** with 0.6–2 grams of starting **5** (1.1 equiv of TsOH, MeCN, reflux, 2–4 h) resulted, without any obvious dependences, in mixtures of macrocycles **6** and **7** in different ratios, in which the content of the latter sometimes reached 29%. By careful observation of reaction mixtures, we have found out that high homogeneity of the mixtures and efficient mixing at the beginning of the reactions are key premises for the exceptional formation of a 14-membered macrocycle **6**. Finally, we have developed a preparative protocol for the synthesis of **6** in multi-gram quantities, which involves the addition of a warm solution of TsOH in MeCN to a boiling stirred suspension of **5** in MeCN. We tested this procedure repeatedly with loadings of hydrazone **5** up to 6.48 g, and in all cases, it afforded only macrocycle **6**.

In contrast to the acid-promoted transformation of hydrazone **5** to give 14- and/or 28-membered macrocycles **6** and/or **7**, hydrazones of 4-(1-aryl-3-oxobut-1-yl)semicarbazides **1** under similar conditions are converted exclusively to 14-membered macrocycles **3** (Scheme 1).^14b,14c^ Thus, hydrazones **1** undergo only dimerization to afford the corresponding dimers, which then cyclize to macrocycles **3**. The low reactivity of these dimers toward to their further dimerization to give tetramers can be explained mainly by the steric hindrance from the two bulky aryl groups and the significant conformational rigidity of the dimers. This was confirmed by the DFT calculations (EtOH solution) for the phenyl-substituted hydrazone (*E*)-**1** (R = Ph) and its (*E,E*)-dimer (see the Supporting Information).

**Figure 3:**
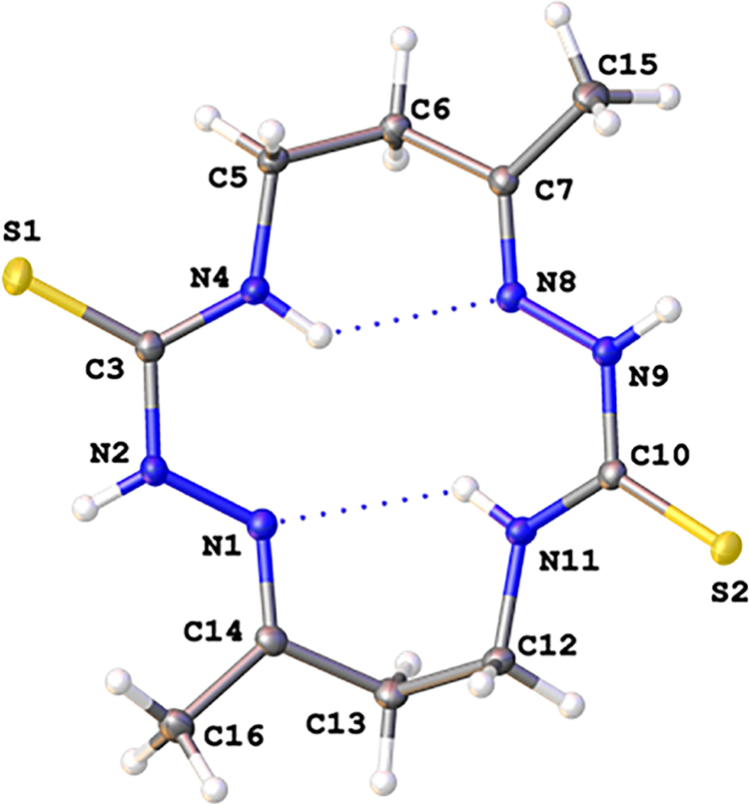
View of a molecular X-ray structure of **6** (crystallization from DMF). Displacement ellipsoids are shown at a 50% probability level. Dotted lines indicate H-bonds.

**Figure 4:**
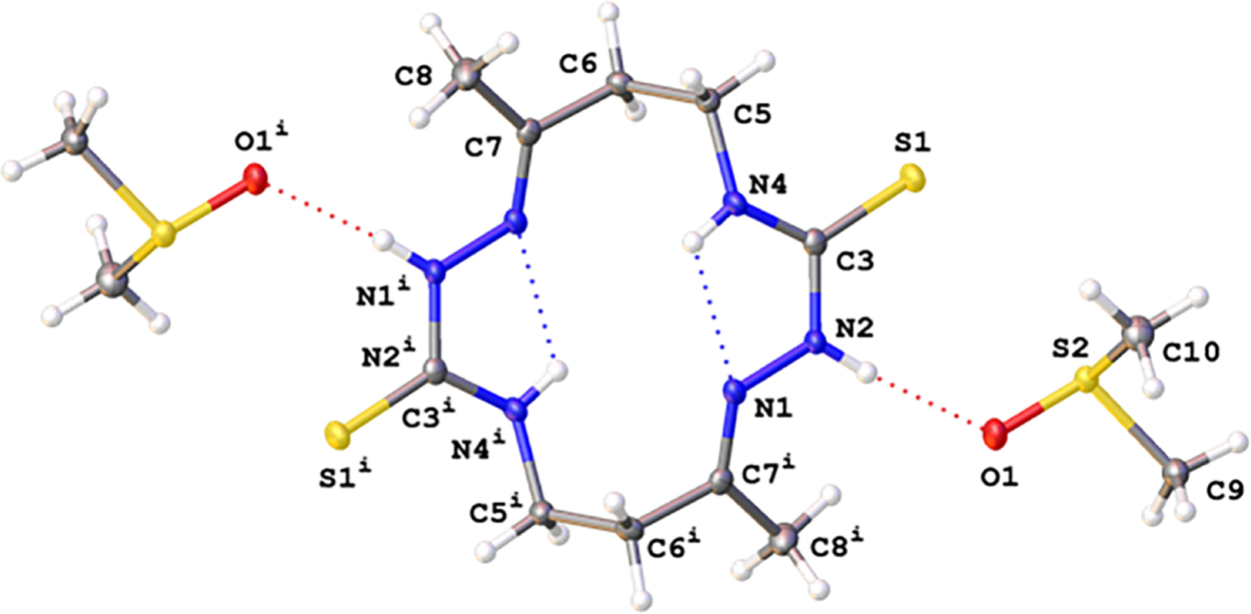
View of a molecular X-ray structure of the DMSO solvate of **6** (**6·**2DMSO) (crystallization from DMSO). Displacement ellipsoids are shown at a 50% probability level. Dotted lines indicate H-bonds. Symmetry transformation: *i* – (1 – *x*, 1 – *y*, 1 – *z*).

**Figure 5:**
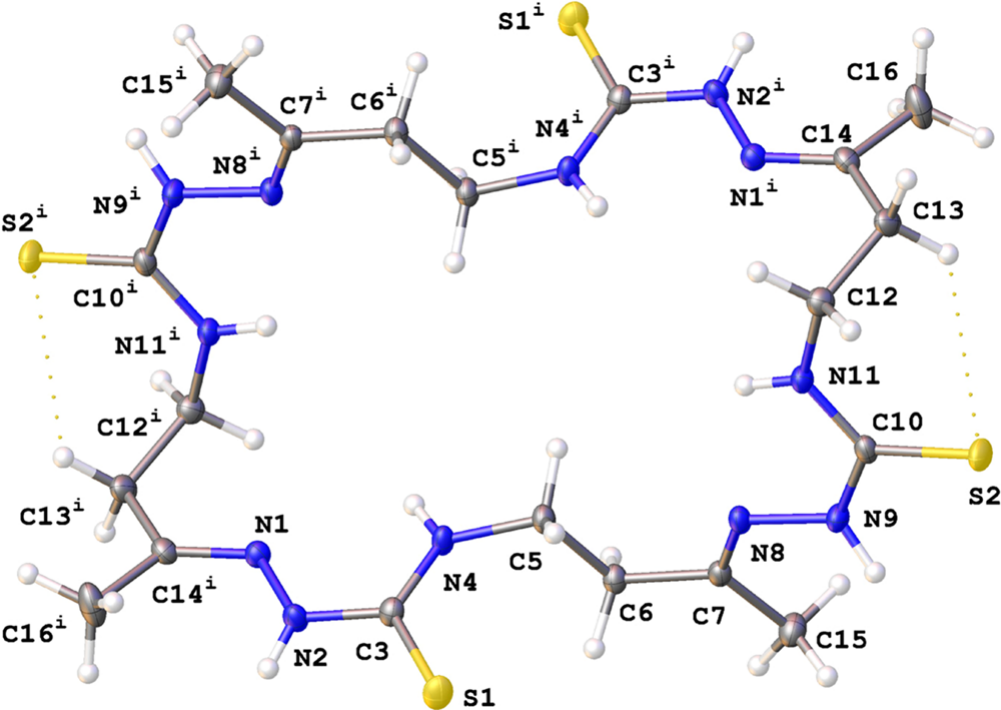
View of a molecular X-ray structure of **7** in its DMF solvate (**7·**6DMF) (crystallization from DMF). Displacement ellipsoids are shown at a 50% probability level. Dotted lines indicate H-bonds. Symmetry transformation: *i* – (1 – *x*, 1 – *y*, 1 – *z*). The CH_3_ groups at the C14 and C14^*i*^ are disordered over two positions with an occupancy ratio of 0.67:0.33, and only the major orientation of these groups is shown.

The structure of the synthesized macrocycles **6** and **7** was unambiguously confirmed by IR, 1D, and 2D NMR spectroscopy, high- and low-resolution mass spectrometry, elemental analysis, as well as by single crystal X-ray diffraction (Figures 3, 4, 5).

The ^1^H and ^13^C{1H} NMR spectra of **6** and **7** in DMSO-d_6_ show only five carbon signals and five proton multiplets, which indicates equivalence of all thiosemicarbazone fragments in their molecules. The ^1^H,^1^H NOESY experiments for **6** and **7** in DMSO-d_6_ demonstrated that a diagnostic NOE was observed between the CH_3_ and HN–N=C protons, thus proving the (*E*)-configuration of all the C=N double bonds.

Interestingly, in ^1^H NMR spectra of various samples of pure **7** in DMSO-d_6_, in addition to the five main proton multiplets, low-intensity additional multiplets of analogous protons are also observed. The total intensity of the latter increases gradually with increasing temperature up to 90 °C (an NMR tube experiment). After cooling to room temperature, this intensity decreases to the initial level. These multiplets can be assigned to non-symmetrical minor conformers of **7**, the probability of which is increased due to the large ring size. Thus, at 90 °C in DMSO-d_6_, macrocycle **7** exists as an equilibrium mixture of three conformers: the symmetrical major and two asymmetric minors in a molar ratio of 73:22:5.

It is noteworthy that, according to X-ray analysis, the conformation of **6** in a crystal grown from a solution in DMSO differs significantly from the conformation in a crystal grown from a solution in DMF (Figures 3 vs 4).

## Conclusions

In summary, unprecedented self-assembly of 14- and 28-membered bis- and tetrakis-thiosemicarbazone macrocycles via strong acid-promoted cyclooligomerization of 4-(4-thiosemicarbazido)butan-2-one hydrazone has been discovered. A thorough study of the influence of various factors on the direction of macrocyclization provided the optimal conditions for the highly selective formation of each of the macrocycles in excellent yields. Thus, the formation of the 28-membered macrocycle is mainly favored by low temperatures and a protic solvent, while that of the 14-membered macrocycle by an aprotic solvent under reflux. These results are in accord with the formation of a rather stable at low temperatures complex of the intermediate dimer with the hydrazinium cation, in which the possibilities of intramolecular cyclization into the 14-membered macrocycle are blocked. We believe that the obtained macrocycles, which are readily prepared in multi-gram quantities, will serve as novel platforms for the investigation of host–guest interactions and for the exploration of the chemistry of these unique macroheterocycles.

## Experimental Section

### General Procedures

All solvents and liquid reagents purchased from commercial sources were distilled prior to use. Petroleum ether had a distillation range of 40–70 °C. When needed, 95% ethanol was used. 4-Isothiocyanatobutan-2-one was prepared according to our regioselective procedure^19^ by the addition of HN_3_ to methyl vinyl ketone followed by a reaction of the obtained 4-azidobutan-2-one with CS_2_ and PPh_3_ in 40% overall yield after vacuum distillation.

FTIR spectra were recorded using a Bruker Alpha-T spectrophotometer in KBr and a Bruker Vector 22 spectrophotometer in Nujol. Band characteristics in the IR spectra are defined as very strong (vs), strong (s), medium (m), weak (w), shoulder (sh), and broad (br). NMR spectra (solutions in DMSO-d_6_) were acquired using a Bruker DPX-300 spectrometer at 300.13 (^1^H) and 75.48 (^13^C) MHz or Bruker Avance III 600 spectrometer at 600.13 (^1^H) and 150.90 (^13^C) MHz. ^1^H NMR chemical shifts are referenced to the residual proton signal in DMSO-d_6_ (2.50 ppm). In ^13^C{H} NMR spectra, a central signal of DMSO-d_6_ (39.50 ppm) was used as a reference. Multiplicities are reported as singlet (s), doublet (d), triplet (t), quartet (q), and some combinations of these, multiplet (m). Selective ^1^H–^1^H decoupling, DEPT-135 experiments as well as HMQC, HMBC, and NOESY correlation techniques were used to aid in the assignment of ^1^H and ^13^C NMR signals. High-resolution mass spectra (HRMS) were obtained using a Bruker mikrOTOF II focus spectrometer (electrospray ionization (ESI)]. Low resolution mass spectra were recorded on a Finnigan MAT INCOS 50 instrument (electron impact, 70 eV). Elemental analyses (CHN) were performed using a Thermo Finnigan Flash EA1112 apparatus. All yields refer to isolated and spectroscopically pure compounds. The color of substances was white. Single crystals of macrocycle **6** suitable for X-ray crystallographic analysis were obtained by slow crystallization from a saturated solution in dry DMF (63.8 mg of **6** and 1.5 mL of DMF) at room temperature. Crystallization of **6** from a saturated solution in dry DMSO (10.5 mg of **6** and 3.0 mL of DMSO) gave single crystals of DMSO solvate of **6**·(**6**·2DMSO). Single crystals of DMF solvate of macrocycle **7**·(**7**·6DMF) were formed by slow crystallyzation from a saturated solution in dry DMF (11.4 mg of **7** and 1.0 mL of DMF) at room temperature. For details on the X-ray diffraction experiments, see the Supporting Information.

The geometry optimizations were carried out at the B3LYP level of theory using Gaussian 16 suite^24^ of quantum chemical programs. Pople’s basis sets, 6-311++G(d,p), were employed for geometry optimization. The effect of continuum solvation was incorporated by using the PCM. Enthalpies and Gibbs free energies were obtained by adding unscaled zero-point vibrational energy corrections and thermal contributions to the energies (temperature 298.150 Kelvin, pressure 1.000 atm).

### Reaction of 4-Isothiocyanatobutan-2-one (10) with 1.02–1.50 Equivalents of Hydrazine Hydrate: Synthesis of Oligomers 13 (Typical Procedure)

To a cooled ice bath, a stirred emulsion of isothiocyanate **10** (0.445 g, 3.52 mmol) in water (4 mL) was dropwise added a solution of N_2_H_4_·H_2_O (0.178 g, 3.56 mmol) in water (1 mL) over 3 min. The ice bath was removed, and the resulting mixture was stirred at room temperature. After 2.5 h from the beginning of the reaction, the formed white oil was ground using a spatula until crystallization was complete. The obtained suspension was additionally stirred at room temperature for 21.5 h and cooled to 0 °C. The white precipitate was filtered, washed with ice-cold water, petroleum ether, and dried to give a mixture of oligomers **13** (0.470 g) (for ^1^H NMR spectrum, see the Supporting Information, page S2).

### Hydrazone of 4-(3-Oxobut-1-yl)semicarbazide (**5**)

To a stirred solution of N_2_H_4_·H_2_O (37.600 g, 751.10 mmol) in EtOH (130 mL) was dropwise added a solution of isothiocyanate **10** (6.477 g, 50.14 mmol) in EtOH (125 mL) at room temperature over 2 h 45 min. After about 1 h from the beginning of the reaction, the solid started to precipitate. After the addition was completed, the reaction mixture was stirred at room temperature for 3 h, the solvent was evaporated in a vacuum to half volume, and the obtained suspension was cooled (−18 °C). The precipitate was filtered, washed with cold (−18 °C) EtOH (2 times), ice cold water (6 times), petroleum ether (2 times), cold (+4 °C) ether (4 times), and dried to give hydrazone **5** (8.208 g, 93%; white solid) as a mixture of *E*/*Z* isomers in a ratio of 92:8, respectively. An analytically pure sample (*E*/*Z =* 96:4) was obtained after crystallization from DMF. Mp 171.5–172 °C (decomp., DMF). IR (KBr) ν, cm^–1^: 3294 (s), 3249 (s), 3165 (s), 3128 (s) (ν NH), 1654 (s) (ν C=N, δ NH_2_), 1552 (vs), 1522 (s) (thioamide-II); ^1^H NMR of (*E*)-isomer (600.13 MHz, DMSO-d_6_) δ: 8.53 (1H, s, NH-N), 7.83 (H, br s, NH), 5.58 (2H, s, NH_2_N=C), 4.39 (2H, s, N*H*_2_NH), 3.58–3.63 (2H, m, NCH_2_), 2.29–2.32 (2H, m, CH_2_C=N), 1.66 (3H, s, CH_3_); ^1^H NMR of (*Z*)-isomer (600.13 MHz, DMSO-d_6_) δ: 8.64 (1H, s, NH-N), 7.96 (H, br s, NH), 5.73 (2H, s, NH_2_N=C), 4.43 (2H, s, N*H*_2_NH), 3.51–3.56 (2H, m, NCH_2_), 2.35–2.39 (2H, m, CH_2_C=N), 1.77 (3H, s, CH_3_); ^13^C{H} NMR of (*E*)-isomer (150.90 MHz, DMSO-d_6_) δ: 180.9 (C=S), 145.4 (C=N), 40.2 (NCH_2_), 37.7 (*C*H_2_C=N), 14.2 (CH_3_); ^13^C{H} NMR of (*Z*)-isomer (150.90 MHz, DMSO-d_6_) δ: 144.4 (C=N), 38.5 (NCH_2_), 29.0 (*C*H_2_C=N), 22.9 (CH_3_); carbon signal of C=S group was not observed in the spectrum. Anal. calcd for C_5_H_13_N_5_S: C, 34.27; H, 7.48; N, 39.96. Found: C, 34.24; H, 7.49; N, 39.91. HRMS (ESI-TOF) *m*/*z* calcd for C_5_H_14_N_5_S [M + H]^+^ 176.0964, found 176.0970.

### (1*E*,7*E*)-7,14-Dimethyl-1,2,4,8,9,11-hexaazacyclotetradeca-7,14-diene-3,10-dithione (**6**)

#### Method A (About 1 mmol Scale Procedure)

A round-bottom flask was successively charged with hydrazone **5** (0.202 g, 1.15 mmol), TsOH·H_2_O (0.242 g, 1.27 mmol), and MeCN (6 mL) at room temperature. The reaction mixture was refluxed under stirring on a hot plate magnetic stirrer for 2 h, the resulting suspension was evaporated under reduced pressure to dryness. To the dry residue was added a saturated aqueous solution of NaHCO_3_, the mixture was triturated until suspension formed, and cooled (0 °C). The precipitate was filtered, washed with ice-cold H_2_O, petroleum ether, and dried to give macrocycle **6** (0.153 g, 93%; white solid). An analytically pure sample was obtained after crystallization from EtOH. Mp 211–211.5 °C (decomp., EtOH). IR (KBr) ν, cm^–1^: 3361 (w), 3339 (s), 3308 (s), 3164 (br vs) (ν NH), 1634 (w) (ν C=N), 1553 (vs), 1495 (s) (thioamide-II), 1087 (m) (ν N-N); ^1^H NMR (600.13 MHz, DMSO-d_6_) δ: 10.32 (2 × 1H, br s, two NH-N), 8.34 (2 × 1H, br t, ^3^*J* = 5.3 Hz, two NH), 3.65–3.69 (2 × 2H, m, two NCH_2_), 2.45–2.48 (2 × 2H, m, two CH_2_-C=N), 1.97 (2 × 3H, s, two CH_3_); ^13^C{H} NMR (150.90 MHz, DMSO-d_6_, at 45 °C) δ: 177.1 (two C=S), 155.3 (two C=N), 40.2 (two NCH_2_), 35.6 (two N=C–*C*H_2_), 18.0 (two CH_3_); MS (EI) *m/z*: 287 [19, (M + 1)^+^], 286 (41, M^+^), 211 (15), 200 (10), 164 (18), 149 (12), 143 (32), 128 (20), 127 (27), 116 (15), 114 (10), 110 (12), 102 (15), 96 (10), 86 (31), 85 (33), 59 (35), 54 (42), 42 (100), 31 (75). Anal. calcd for C_10_H_18_N_6_S_2_: C, 41.93; H, 6.33; N, 29.34. Found: C, 41.96; H, 6.29; N, 29.38. HRMS (ESI-TOF) *m*/*z* calcd for C_10_H_19_N_6_S_2_ [M + H]^+^ 287.1107, found 287.1111; *m*/*z* calcd for C_10_H_18_N_6_NaS_2_ [M + Na]^+^ 309.0927, found 309.0928.

#### Method B (a Multi-Gram Procedure)

To an intensively stirred suspension of hydrazone **5** (6.141 g, 34.84 mmol) in hot (∼80 °C) MeCN (50 mL) was added a solution of TsOH·H_2_O (7.299 g, 38.37 mmol) in hot (∼80 °C) MeCN (55 mL) in one portion, the obtained mixture was stirred under reflux on a hot plate magnetic stirrer for 3 h, then the solvent was removed in vacuum. To the resulting dry residue was added a saturated aqueous solution of NaHCO_3_, and the mixture was triturated until suspension formed and cooled (0 °C). The white precipitate was filtered, washed with ice-cold H_2_O, petroleum ether, and dried to give macrocycle **6** (4.870 g, 98%).

### (1*E*,7*E*,14*E*,21*E*)-7,14,21,28-Tetramethyl-1,2,4,8,9,11,15,16,18,23,25-undecaazacyclooctacosa-7,14,21,28-tetraene-3,10,17,24-tetrathione (**7**)

A stirred solution of TsOH·H_2_O (0.212 g, 1.11 mmol) in EtOH (2 mL) was cooled in NaCl (33 g)/ice bath (100 g) for 5 min (during this time temperature of the bath raised from −14 to −10.5 °C), then hydrazone **5** (0.174 g, 1.01 mmol) was added in one portion. After 20 min (temperature of bath raised to −5 °C) fresh bath (−17 °C) was used, and the reaction mixture was stirred for 1 h (temperature of bath raised to 0 °C). Then, stirring of the resulting suspension was continued in an ice bath (temperature of bath +3 to +5 °C) for an overall duration of 7 h. The solvent was removed in a vacuum. The residue was triturated with a saturated aqueous solution of NaHCO_3_, and the obtained suspension was cooled (0 °C). The white crystalline precipitate was filtered, washed with ice-cold H_2_O, petroleum ether, and dried to give a 91:9 mixture of **7** and **6** (0.139 g). After the crystallization of this product from boiling DMF, according to ^1^H NMR spectroscopy data, a solvate (0.124 g) of macrocycle **7** with 58 mol % DMF was obtained. The calculated yield of **7** was 0.105 g (73%). To remove residual DMF, the solvate (0.051 g) was stirred with MeOH (3 mL) for 48 h at room temperature, followed by cooling (0 °C), filtration, and drying to give pure **7** (0.036 g) as a white solid. Mp 211.5 °C (decomp., DMF). IR (KBr) ν, cm^–1^: 3454 (br m), 3375 (s), 3334 (s), 3196 (br vs) (ν NH), 1672 (m), 1653 (m) (ν C=N), 1545 (vs), 1526 (vs), 1486 (vs) (thioamide-II), 1079 (m) (ν N-N); ^1^H NMR (600.13 MHz, DMSO-d_6_) δ: 9.87 (4 × 1H, br s, four NH-N), 8.20 (4 × 1H, br t, ^3^*J* = 6.0 Hz, four NH), 3.75–3.80 (4 × 2H, m, four NCH_2_), 2.55–2.60 (4 × 2H, m, four CH_2_-C=N), 1.90 (4 × 3H, s, four CH_3_); ^13^C{H} NMR (150.90 MHz, DMSO-d_6_) δ: 177.0 (four C=S), 151.4 (four C=N), 39.1 (four NCH_2_), 37.2 (four N=C-*C*H_2_), 17.2 (four CH_3_). Anal. calcd for C_20_H_36_N_12_S_4_: C, 41.93; H, 6.33; N, 29.34. Found: C, 41.74; H, 6.06; N, 29.07. HRMS (ESI-TOF) *m*/*z* calcd for C_20_H_37_N_12_S_4_ [M + H]^+^ 573.2141, found 573.2136; *m*/*z* calcd for C_20_H_36_N_12_NaS_4_ [M + Na]^+^ 595.1961, found 595.1951; *m*/*z* calcd for C_20_H_36_KN_12_S_4_ [M + K]^+^ 611.1700, found 611.1688.

## Notes

The data underlying this study are available in the published article and its online supplementary material.

## Supporting Information Available

Copies of IR, 1D, and 2D NMR spectra of compounds **5**–**7**, single-crystal X-ray diffraction data of compounds **6** and **7**, computational details (PDF)
