# Supplementary material for: Different Modes of Acid-Promoted Cyclooligomerization of 4-(4-Thiosemicarbazido)butan-2-one Hydrazone: 14-Membered versus 28-Membered Polyazamacrocycle Formation
Source: J Org Chem. 2022 Nov 16;87(23):15722–31. doi: 10.1021/acs.joc.2c01199 (PMC9724087; doi:10.1021/acs.joc.2c01199)
Supplement: Supplementary file 1 — jo2c01199_si_001.pdf [file jo2c01199_si_001.pdf]

# Supporting Information

## Different modes of acid-promoted cyclooligomerization of 4-(4-thiosemicarbazido)butan-2-one hydrazone: 14-membered versus 28-membered polyazamacrocycle formation

Anastasia A. Fesenko,[a] Mikhail S. Grigoriev,[b] Vladimir B. Arion,[c] and Anatoly D. Shutalev[a,]*

[a] *N. D. Zelinsky Institute of Organic Chemistry, Russian Academy of Sciences, 47 Leninsky Ave., 119991 Moscow, Russian Federation*

[b] *Frumkin Institute of Physical Chemistry and Electrochemistry, Russian Academy of Sciences, 31 Leninsky Ave., Bldg 4, 119071 Moscow, Russian Federation*

[c] *University of Vienna, Institute of Inorganic Chemistry, Währinger Strasse 42, A-1090 Vienna, Austria*

### Contents

1. Copy of $^{1}$H NMR spectrum of a mixture of oligomers **13** S2
2. Copies of 1D and 2D NMR spectra of hydrazone **5** S3–S7
3. Copies of $^{1}$H NMR spectra of crude products of cyclooligomerization of **5** under various conditions S8–S9
4. Copies of 1D and 2D NMR spectra of 14-membered macrocycle **6** S10–S15
5. Mass spectrum of 14-membered macrocycle **6** (electron impact, 70 eV) S16
6. Copies of 1D and 2D NMR spectra of 28-membered macrocycle **7** S17–S25
7. Copies of IR spectra of compounds **5**, **6**, and **7** S26–S28
8. X-ray diffraction data S29–S37
9. Computational details S38–S101
10. References and notes S102

$^{1}$H NMR spectrum of the crude product prepared by the reaction of isothiocyanate **10** with 1.01 equiv of $N_2H_4{\cdot}H_2O$ ($H_2O$, rt, 24 h) (300.13 MHz, DMSO-$d_6$)

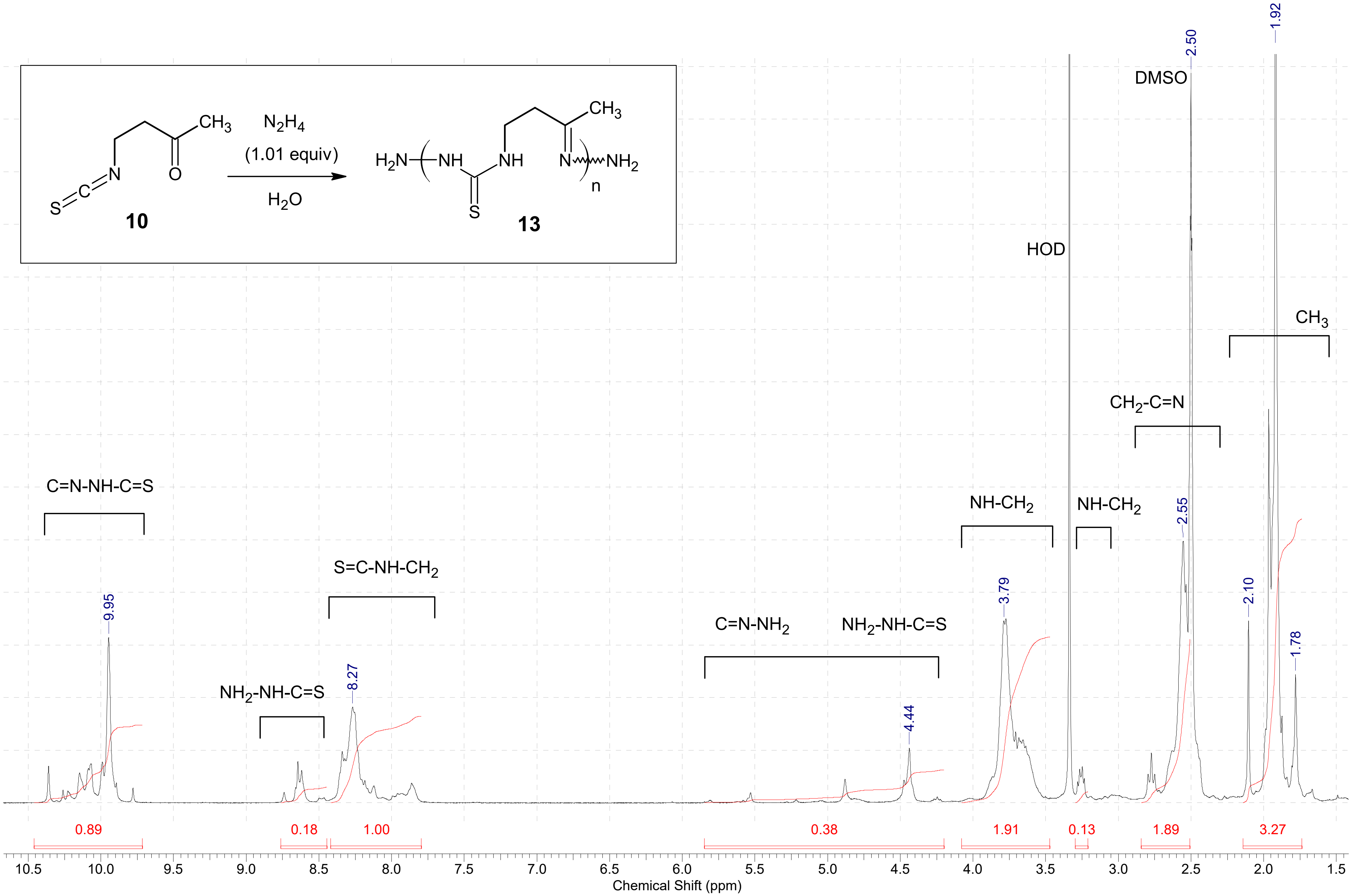

$^{1}$H NMR spectrum of crude hydrazone **5** (mixture of *E*- and *Z*-isomers in a ratio of 92:8) (600.13 MHz, DMSO-$d_6$)

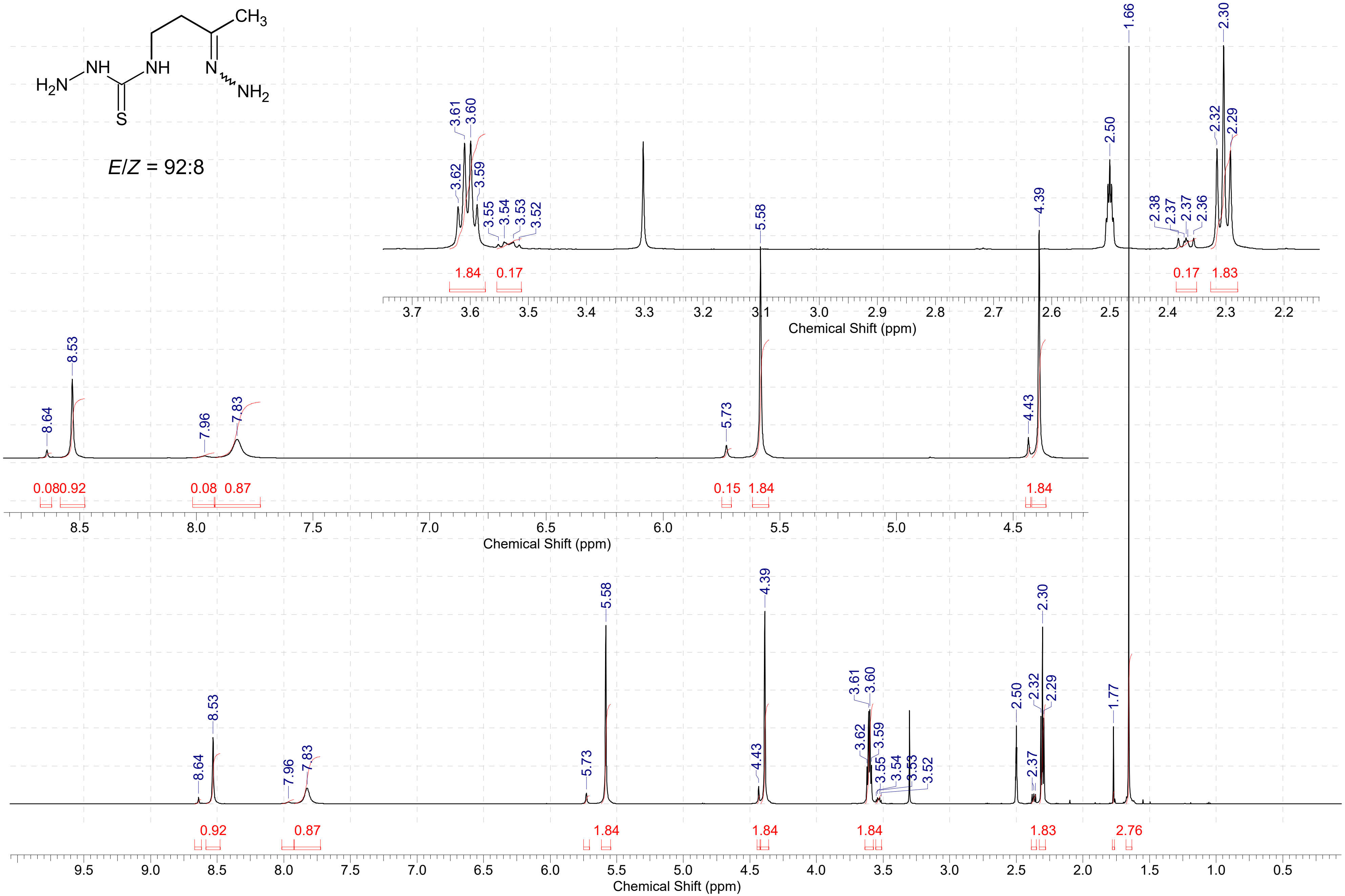

$^{13}C\{1H\}$ NMR spectrum of crude hydrazone **5** (mixture of *E*- and *Z*-isomers in a ratio of 92:8) (150.90 MHz, DMSO-$d_6$)

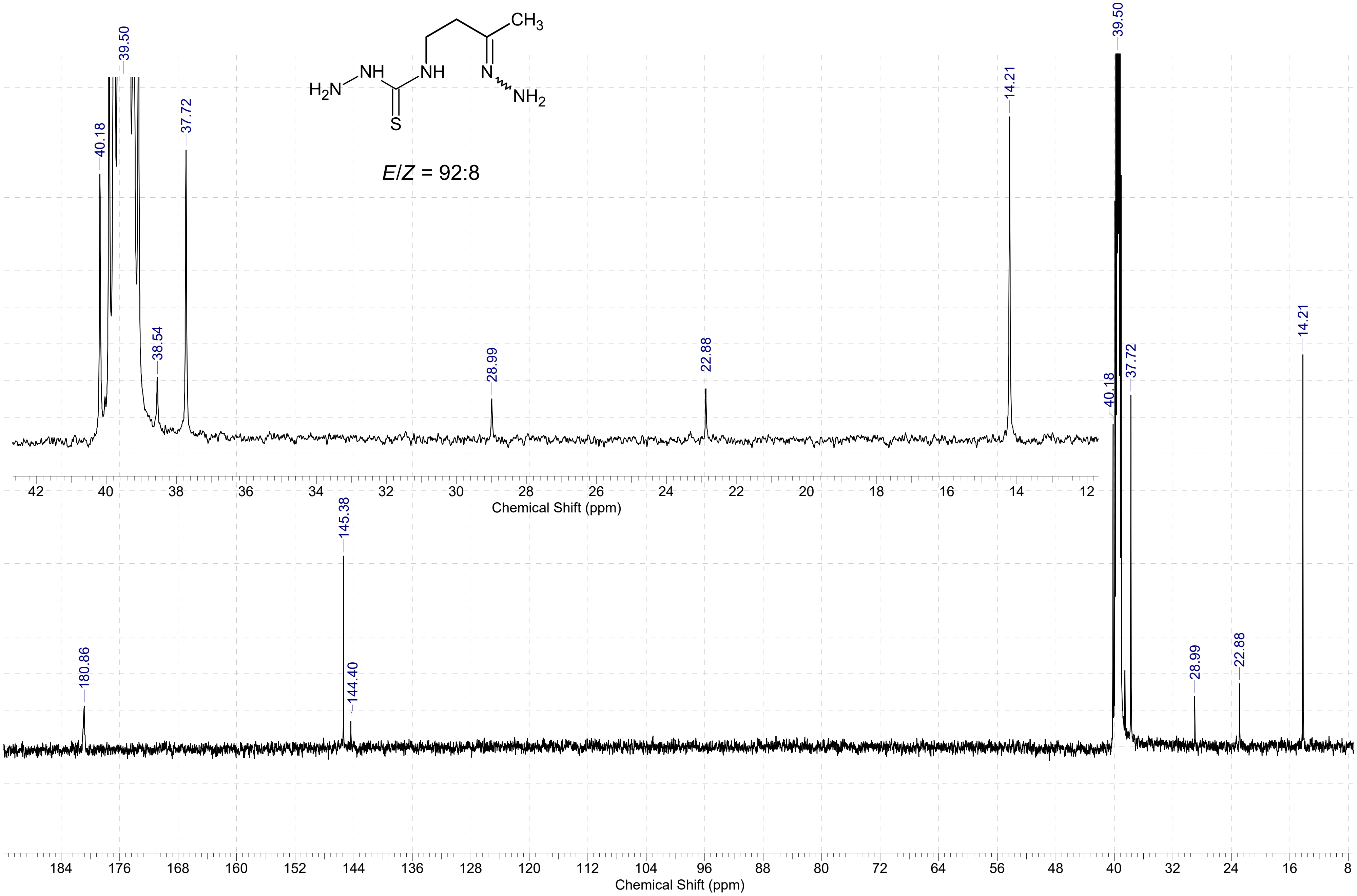

$^{1}$H,$^{13}$C HSQC spectrum of crude hydrazone **5** (mixture of *E*- and *Z*-isomers in a ratio of 92:8) (Bruker Avance III, DMSO-$d_6$)

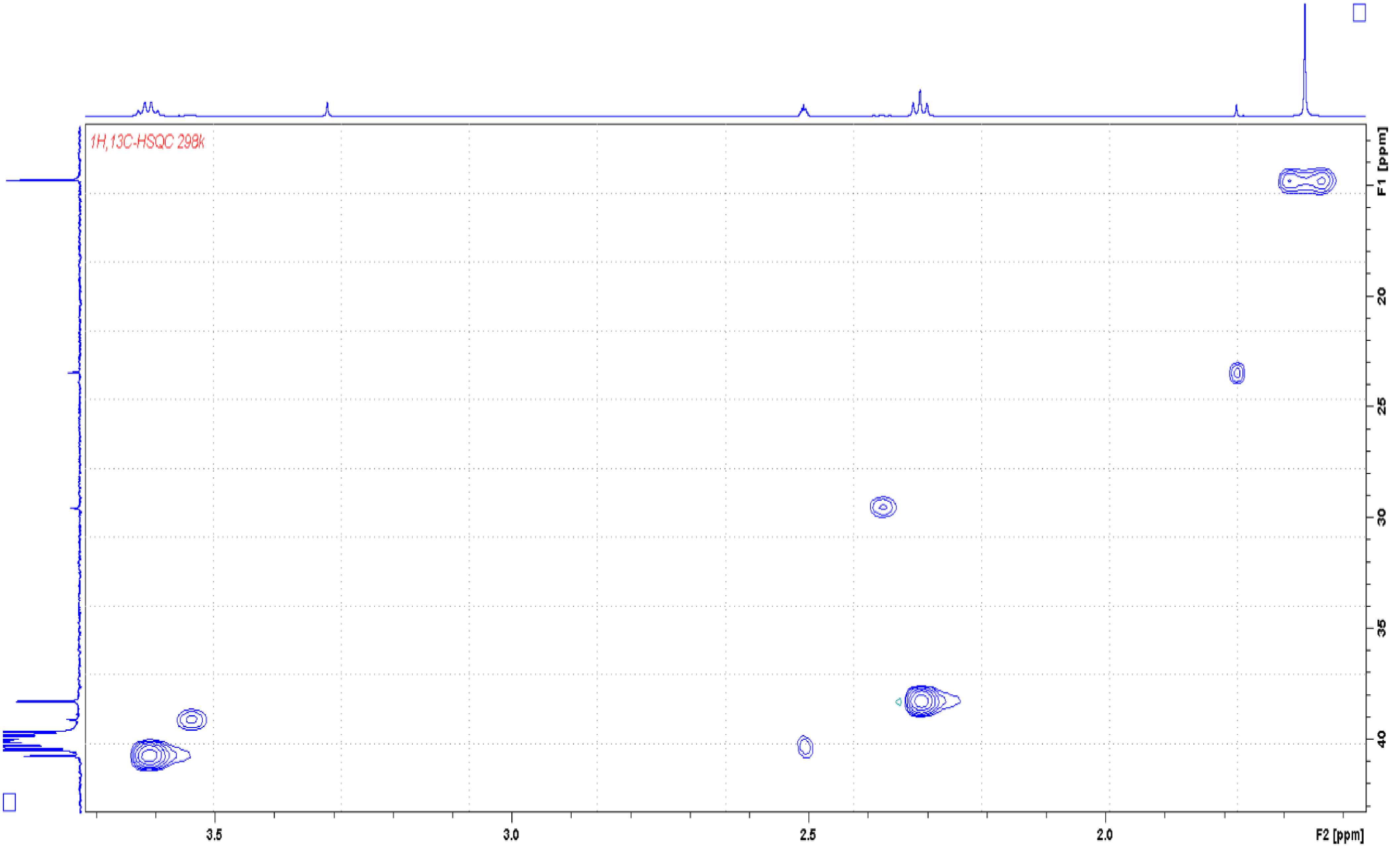

$^{1}$H,$^{1}$H NOESY spectrum of crude hydrazone **5** (mixture of *E*- and *Z*-isomers in a ratio of 92:8) (600.13 MHz, DMSO-$d_6$)

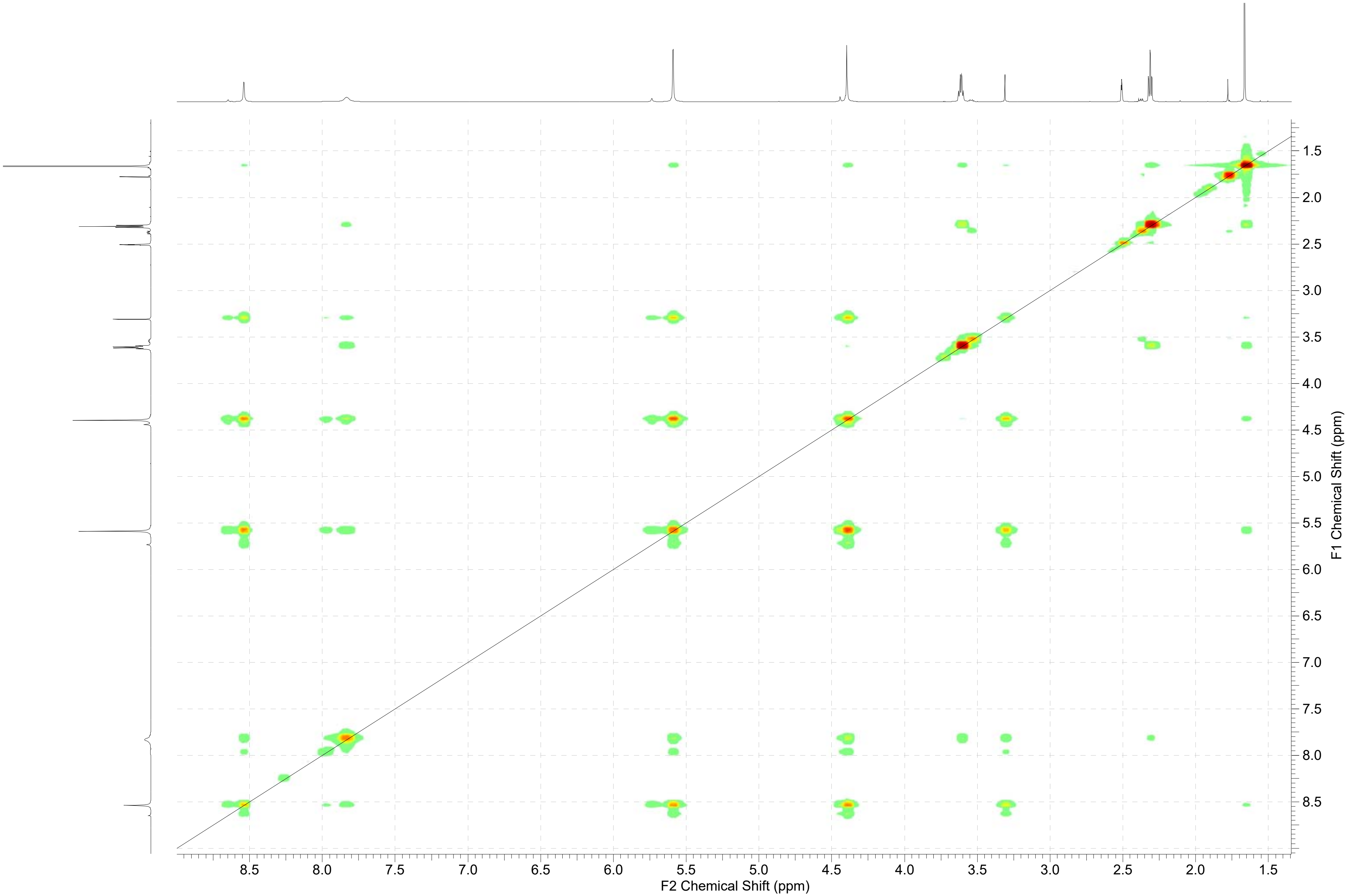

Fragment of $^{1}H,^{1}H$ NOESY spectrum of crude hydrazone **5** (mixture of *E*- and *Z*-isomers in a ratio of 92:8) (600.13 MHz, DMSO-$d_6$)

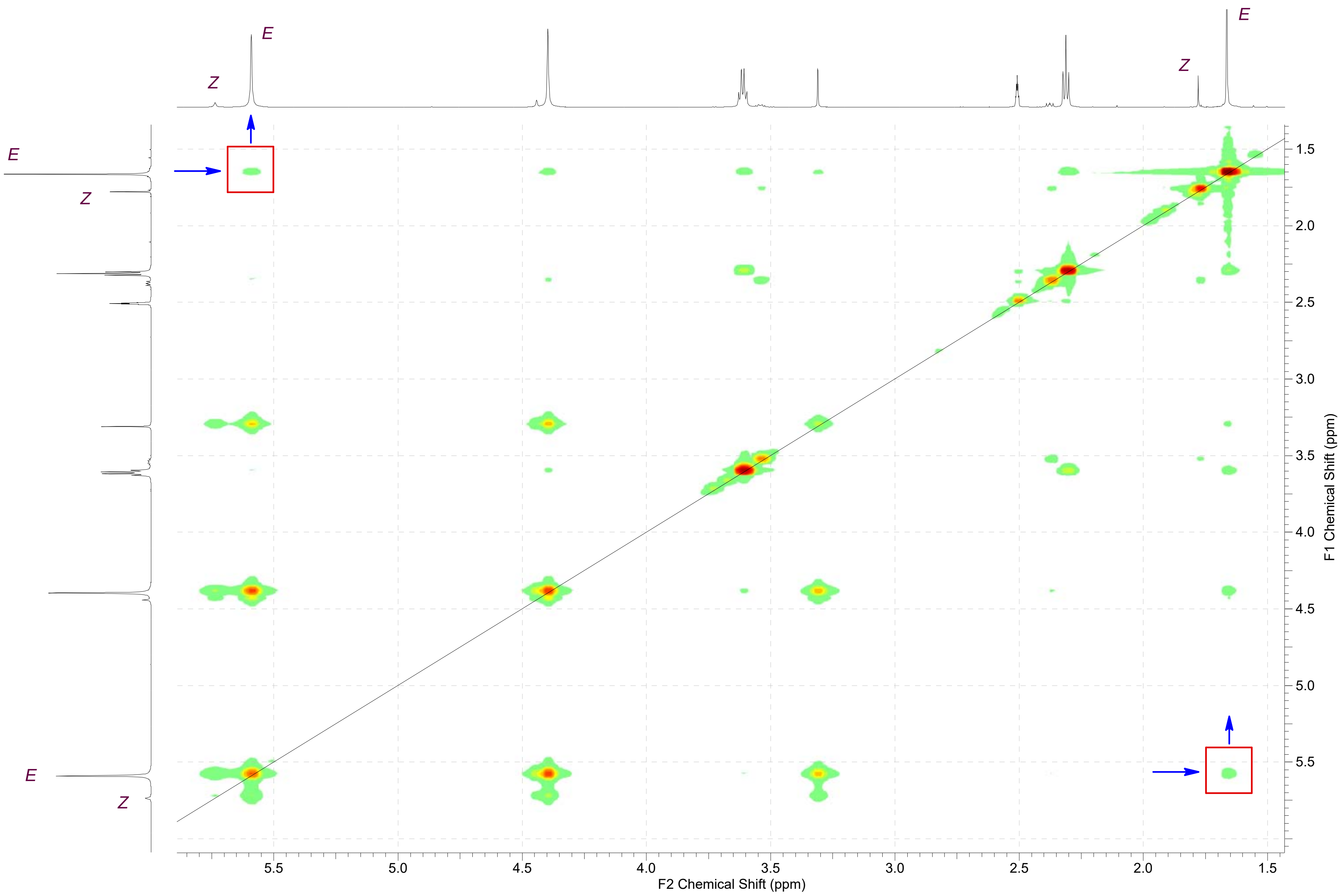

$^{1}$H NMR spectra of crude products formed by the acid-catalyzed cyclization of hydrazone **5** under various conditions (Table 1, entries 1, 2, 7, 8) (DMSO-$d_6$)

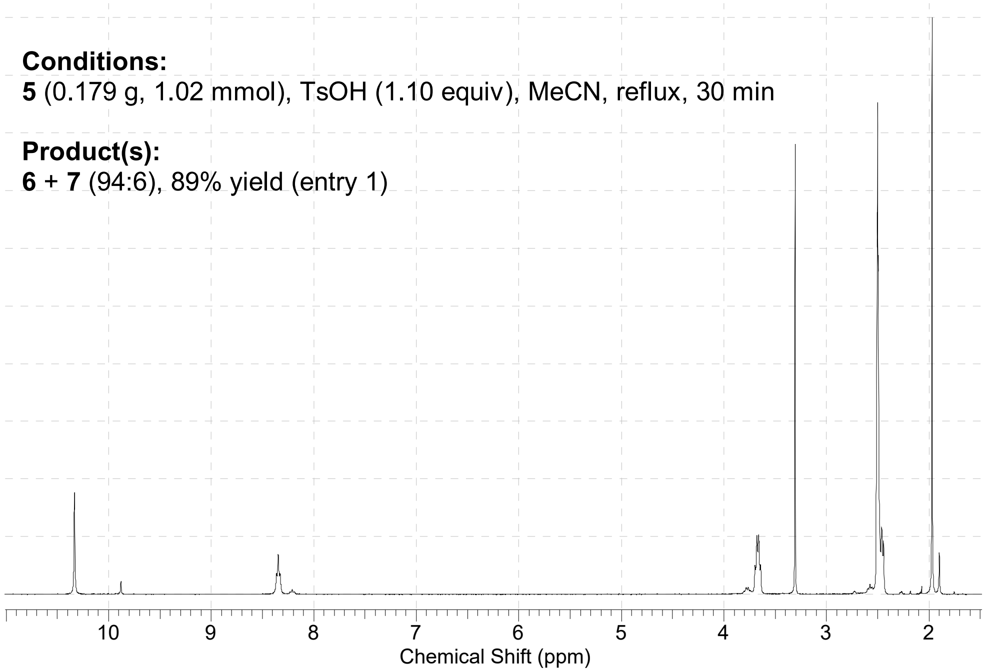


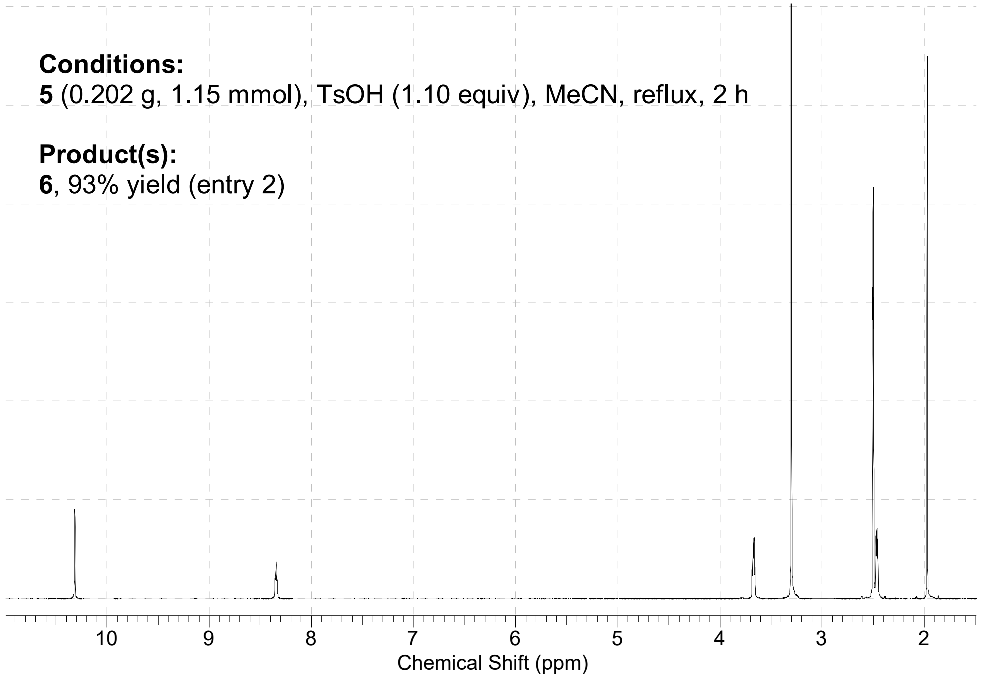


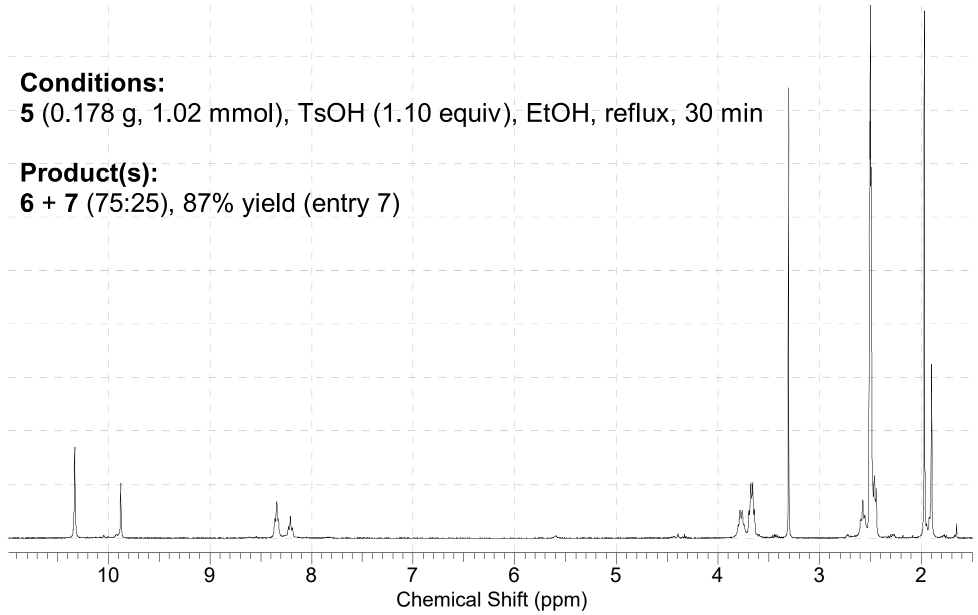


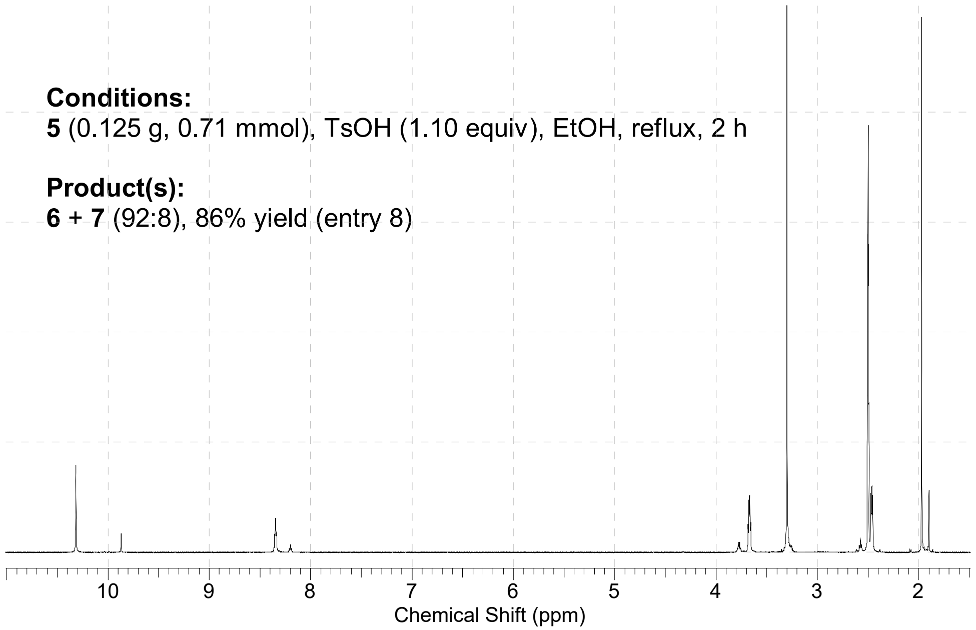

$^{1}$H NMR spectra of crude products formed by the acid-catalyzed cyclization of hydrazone **5** under various conditions (Table 1, entries 5, 14-16) (DMSO-$d_6$)

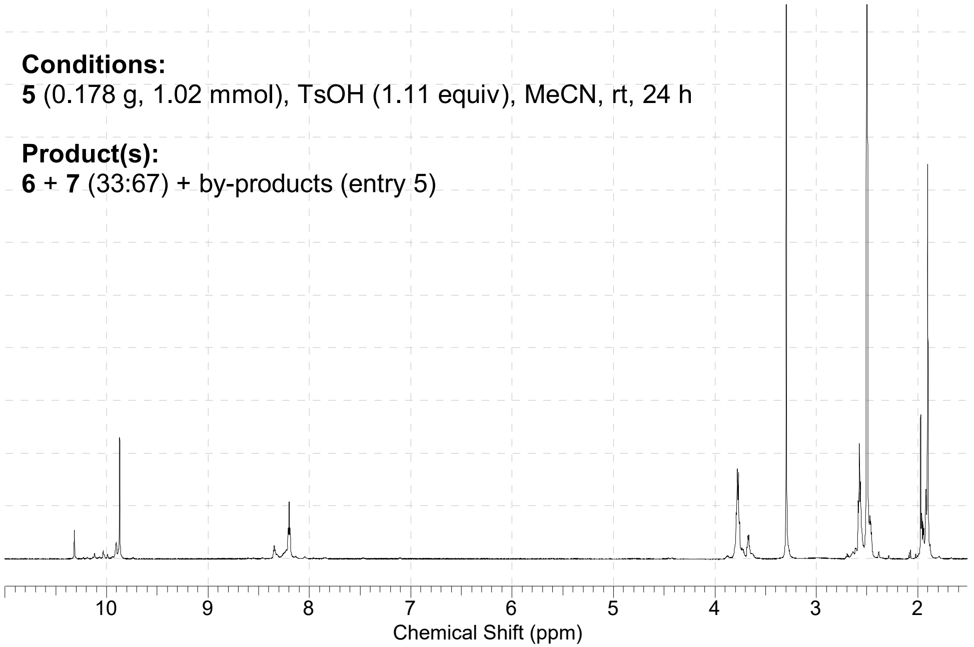


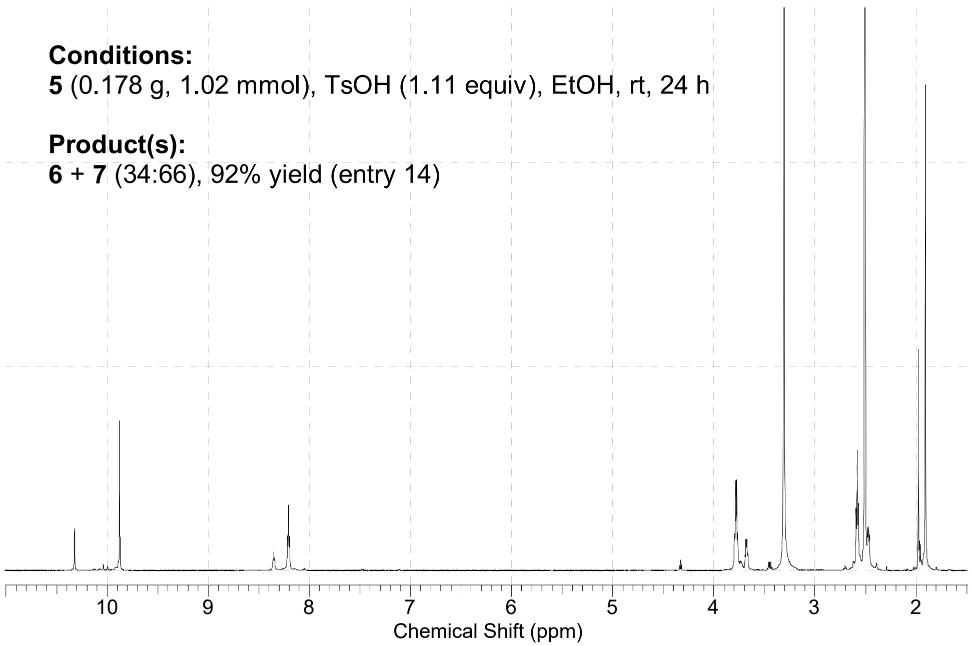


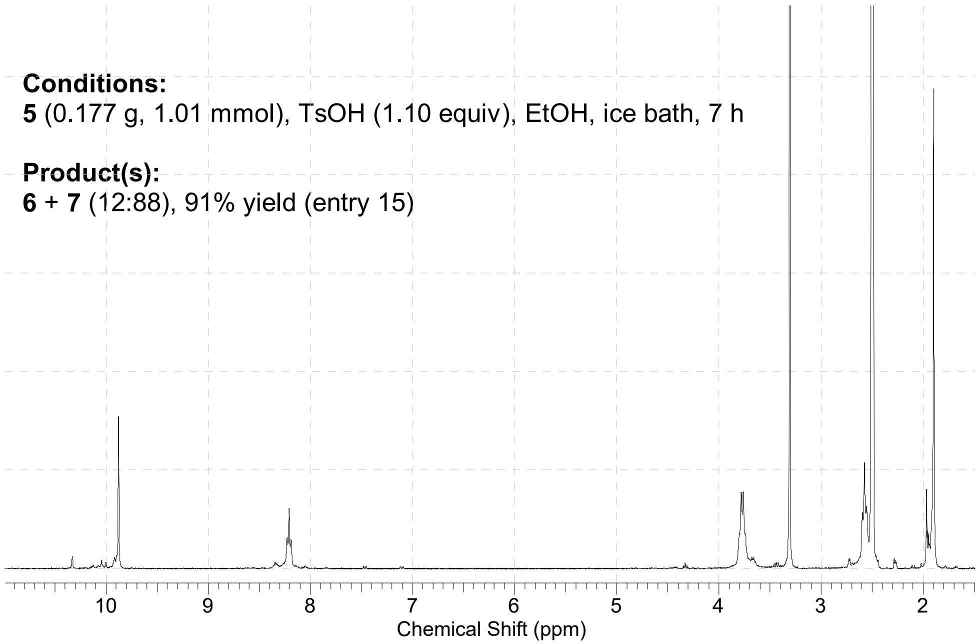


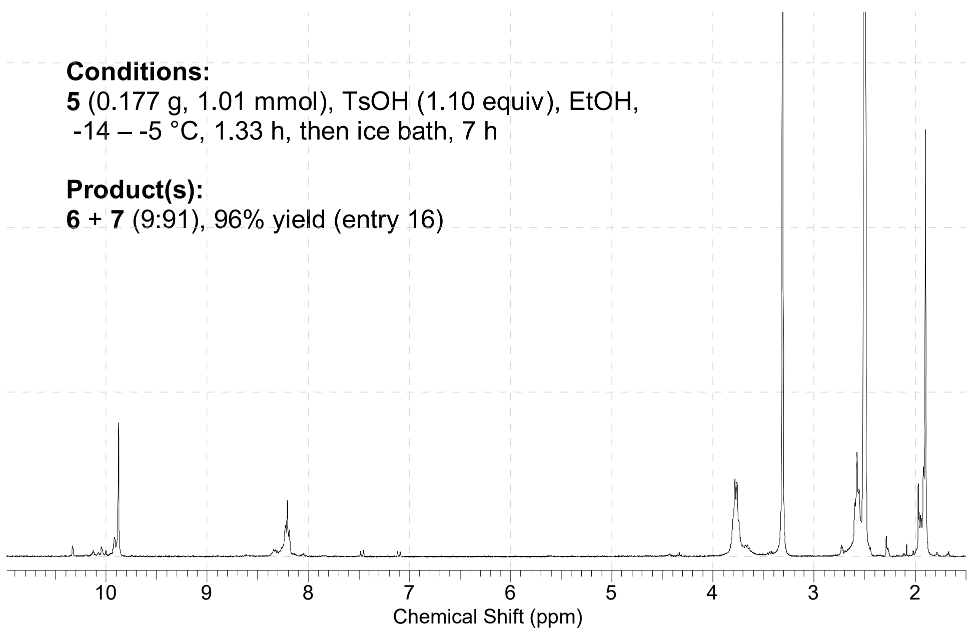

$^{1}$H NMR spectrum of 14-membered cyclic bis-thiosemicarbazone **6** (600.13 MHz, DMSO-$d_6$)

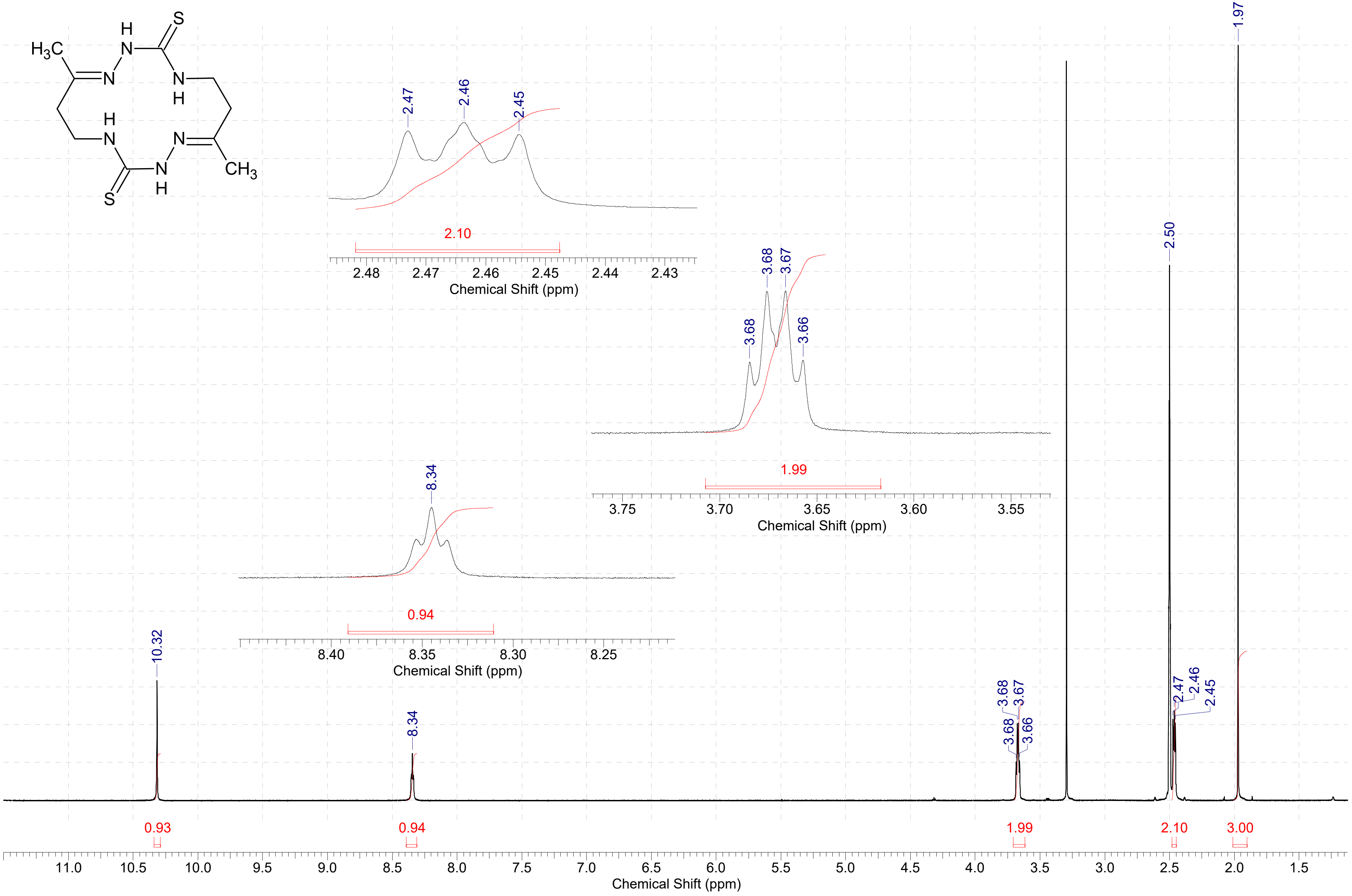

$^{13}C\{1H\}$ NMR spectrum of 14-membered cyclic bis-thiosemicarbazone **6** (150.90 MHz, DMSO-$d_6$)

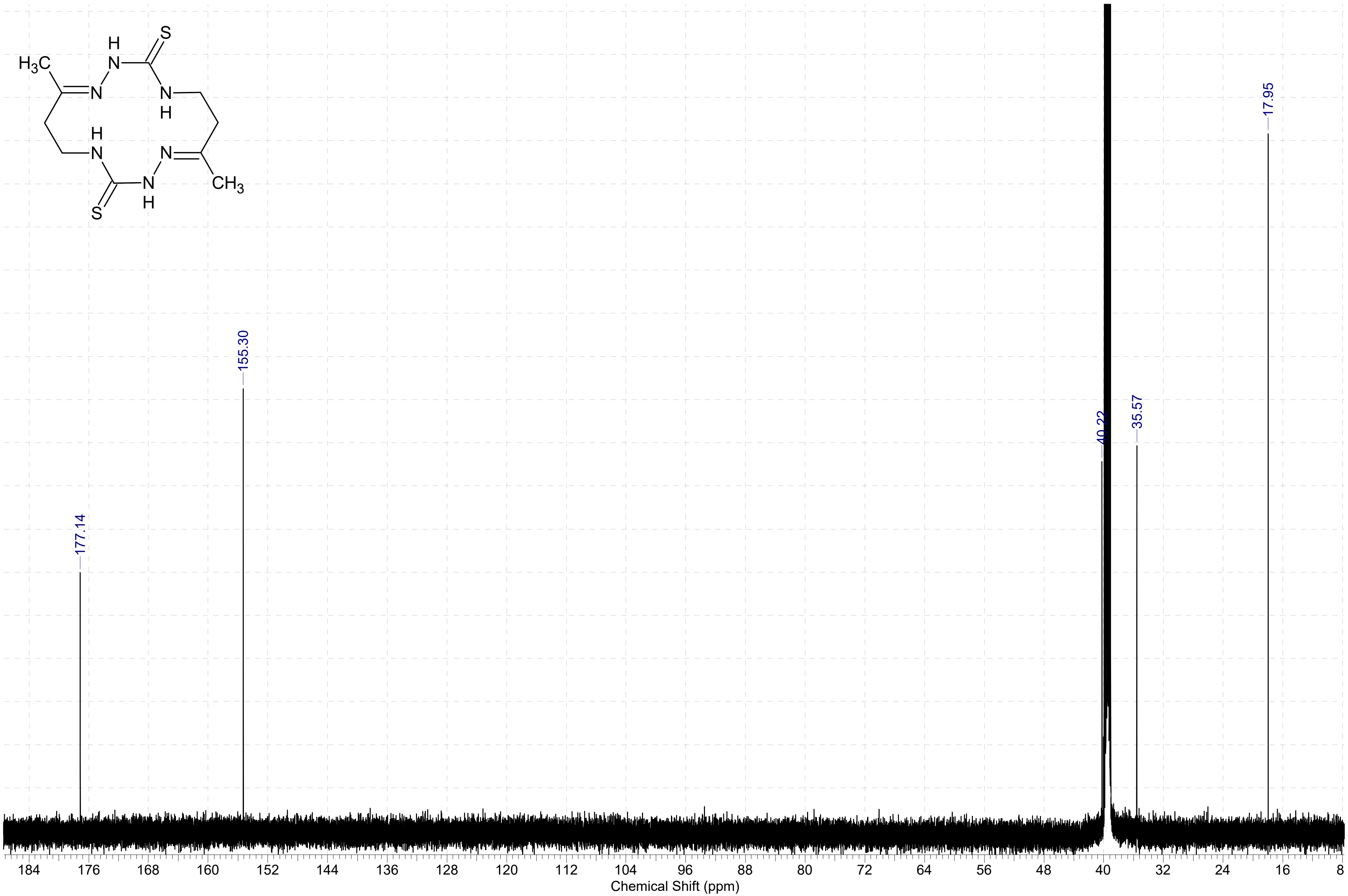

$^{1}$H,$^{13}$C HMBC spectrum of 14-membered cyclic bis-thiosemicarbazone **6** (Bruker Avance III, 45 °C, DMSO-$d_6$)

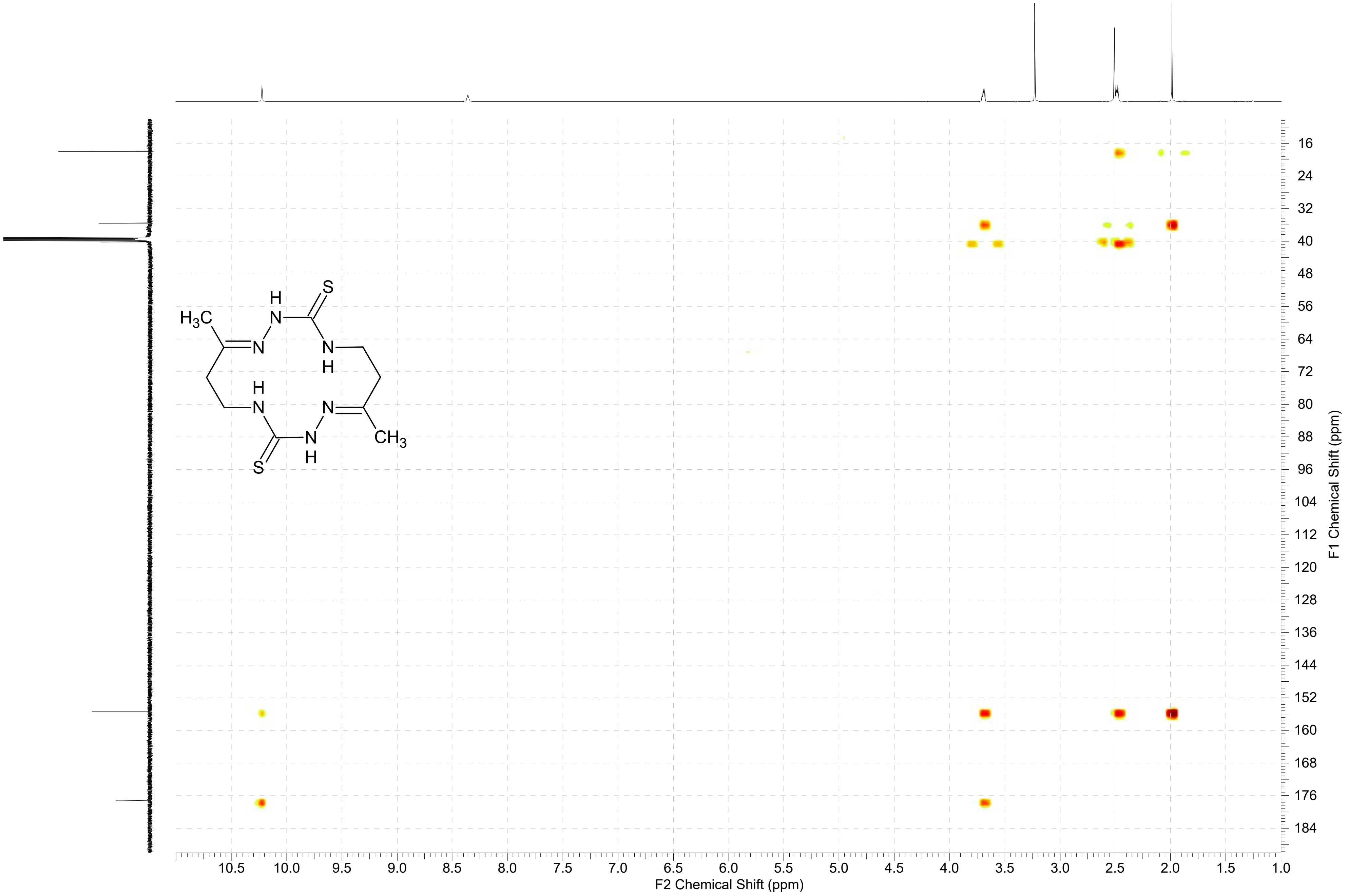

Fragment of $^{1}$H,$^{13}$C HMBC spectrum of 14-membered cyclic bis-thiosemicarbazone **6** (Bruker Avance III, 45 °C, DMSO-$d_6$)

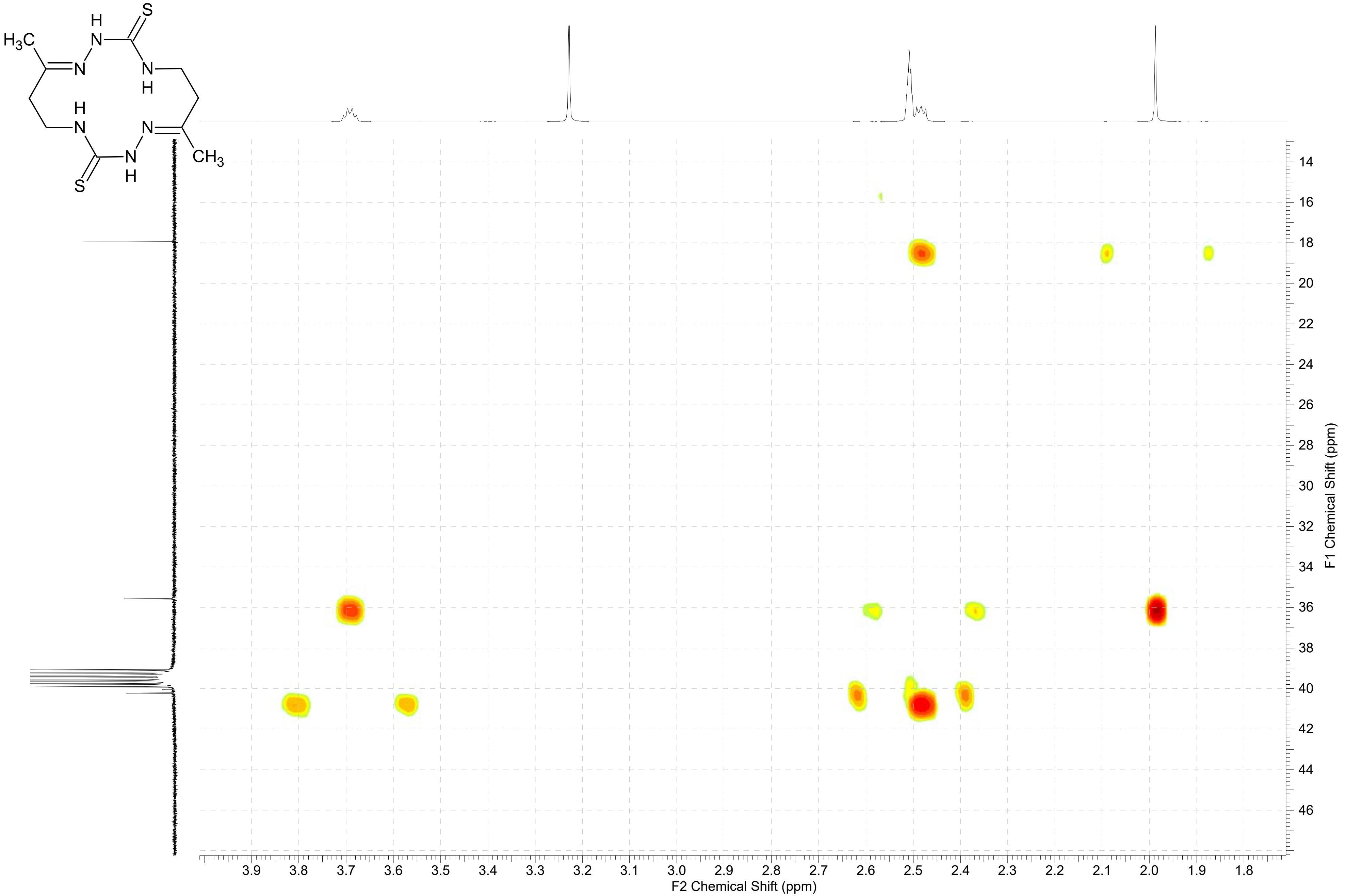

Fragment of $^{1}H,^{13}C$ HMBC spectrum of 14-membered cyclic bis-thiosemicarbazone **6** (Bruker Avance III, 45 °C, DMSO-$d_6$)

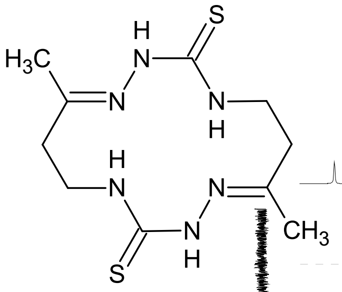

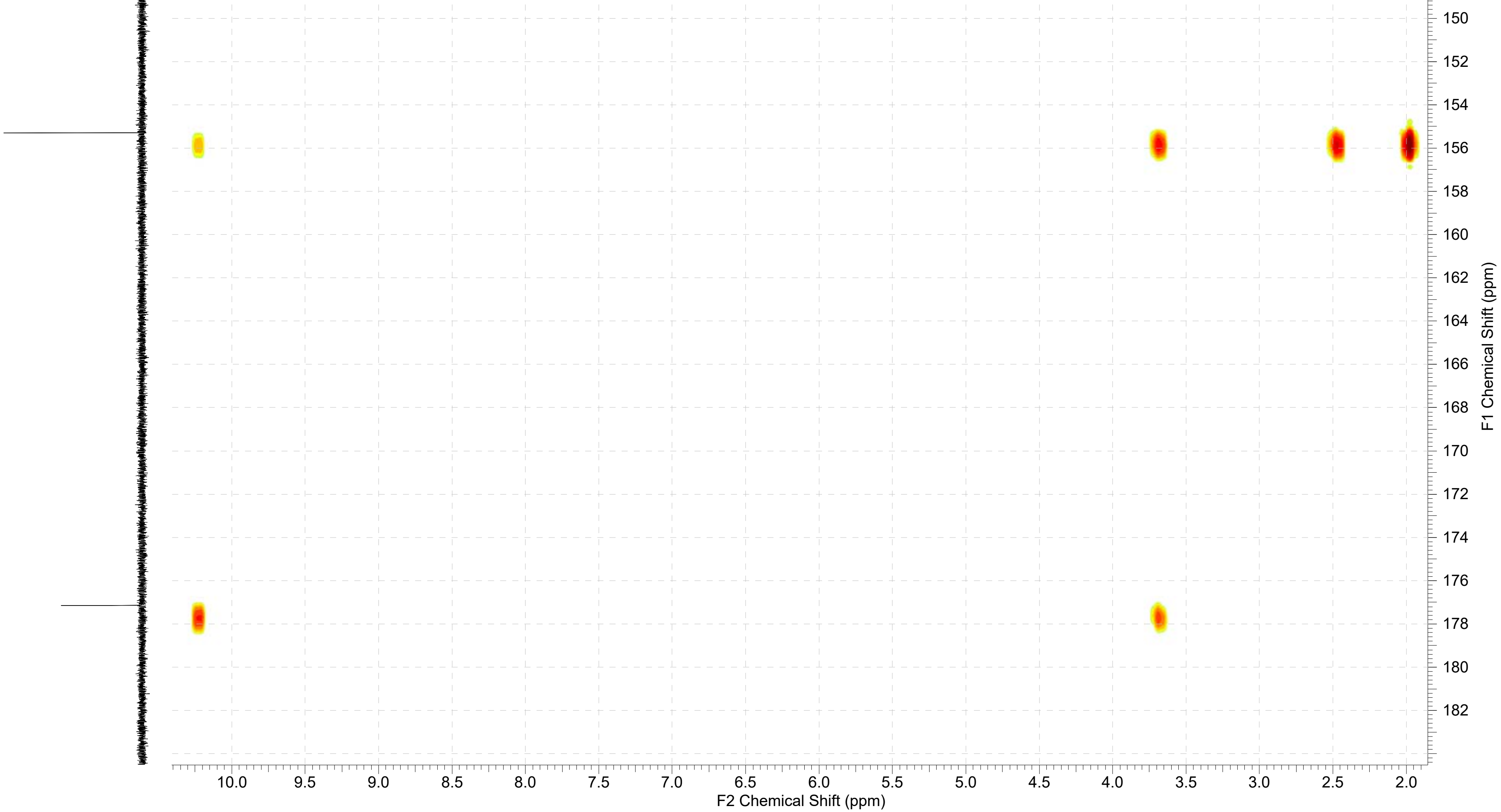

$^{1}H,^{1}H$ NOESY spectrum of 14-membered cyclic bis-thiosemicarbazone **6** (Bruker Avance III, 45 °C, DMSO-$d_6$)

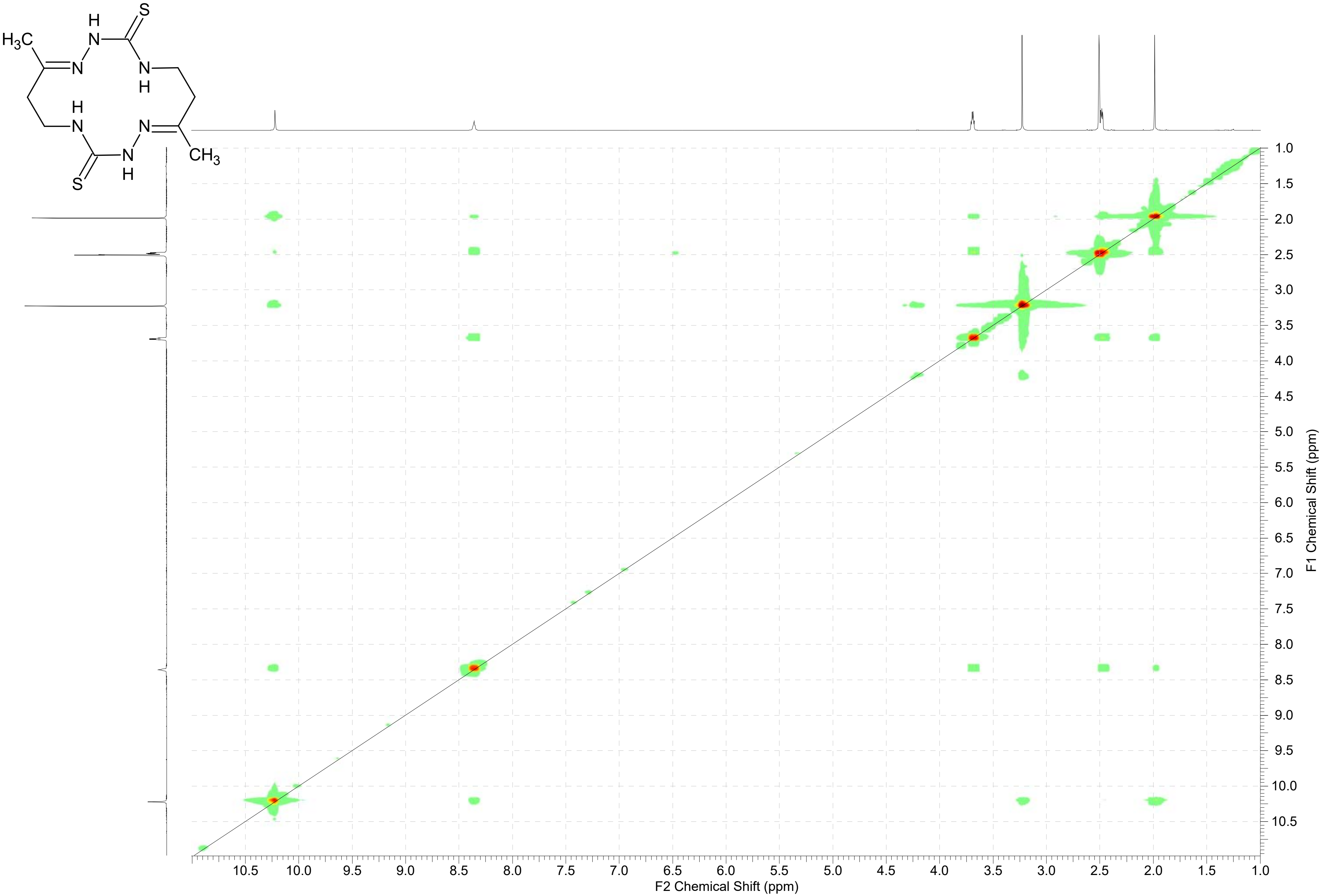

Mass-spectrum of 14-membered cyclic bis-thiosemicarbazone **6** (electron impact, 70 eV)

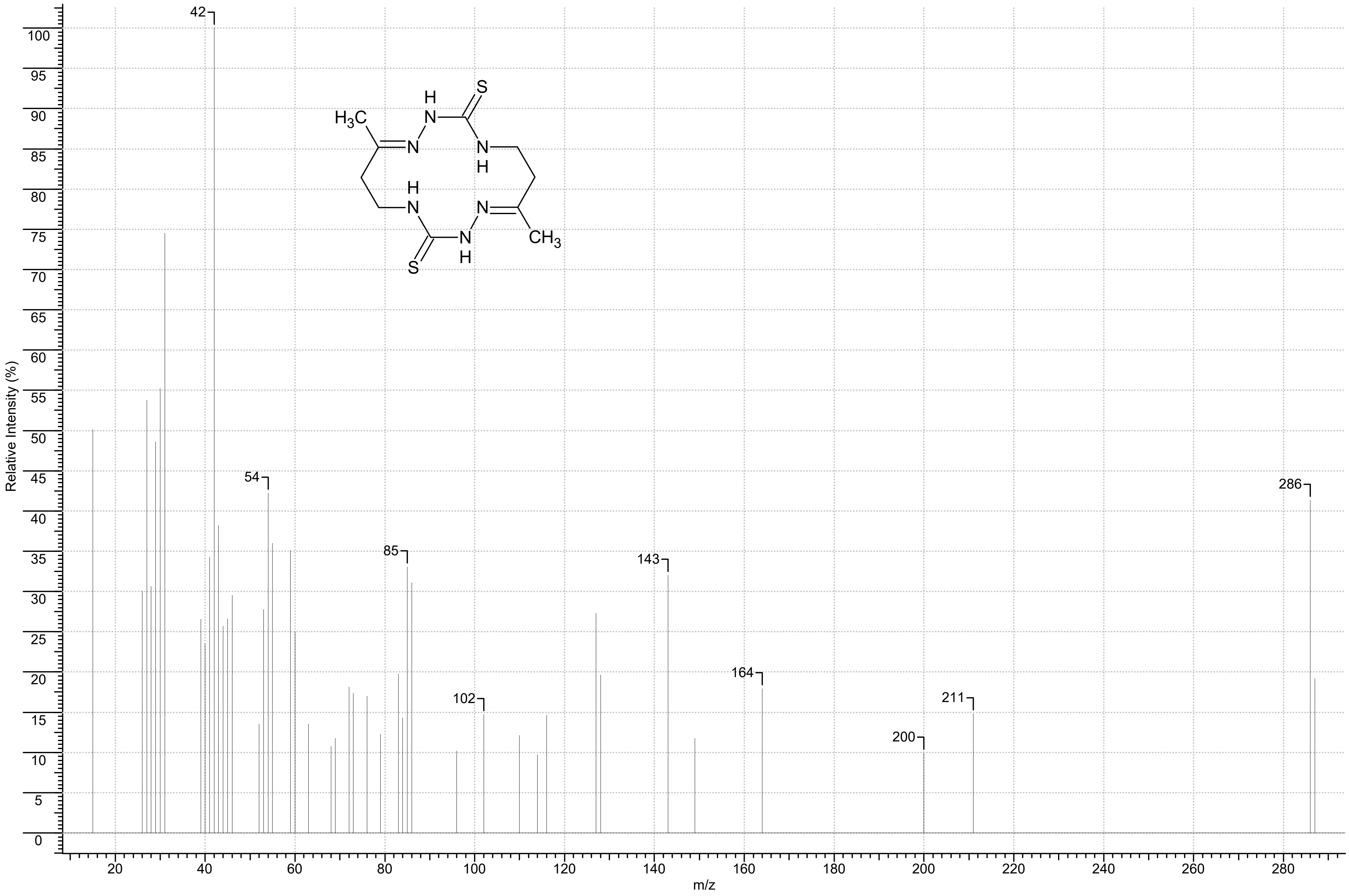

$^{1}$H NMR spectrum of 28-membered cyclic tetrakis-thiosemicarbazone **7** (600.13 MHz, 30 °C, DMSO-$d_6$)

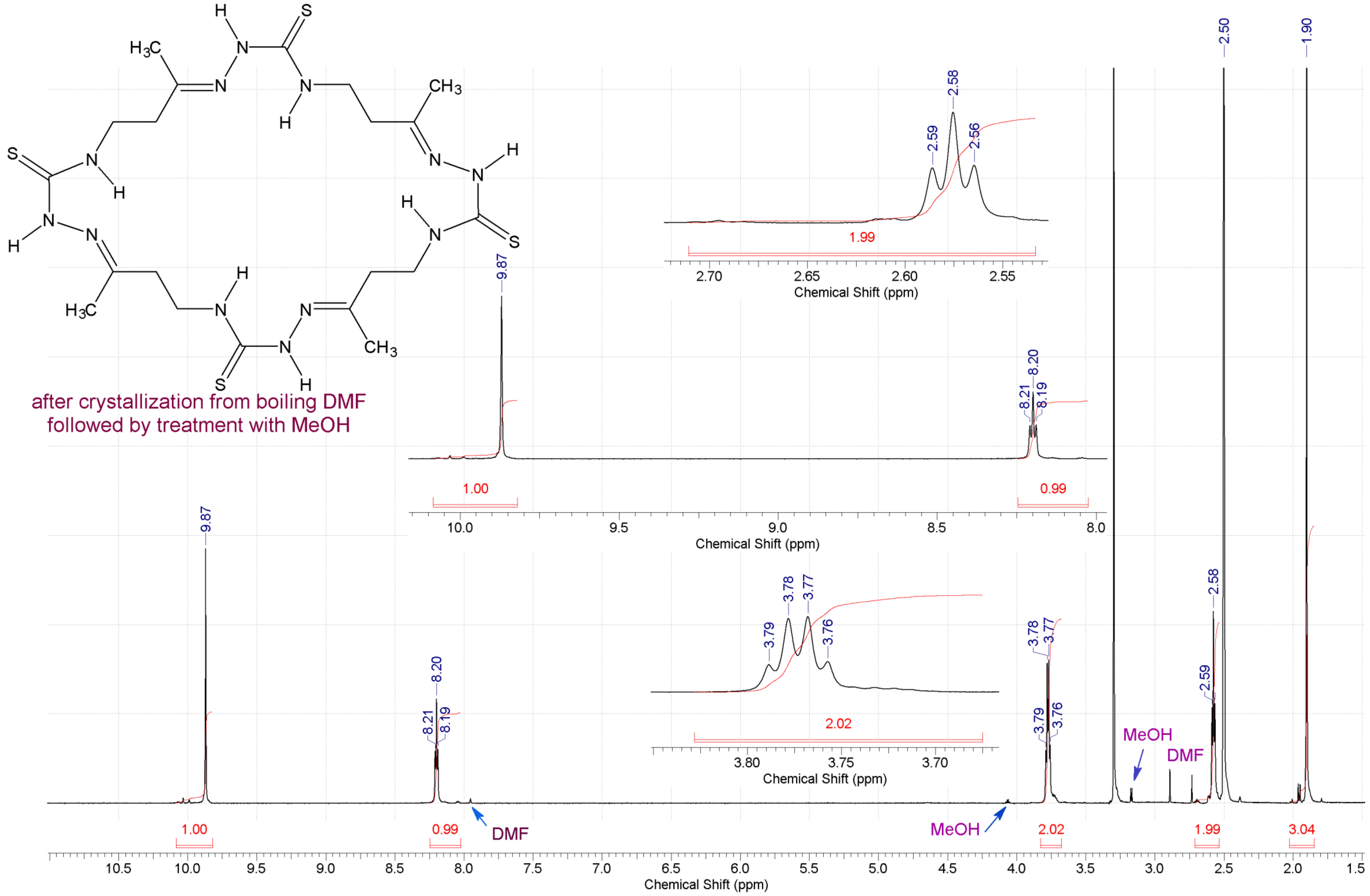

Fragments of $^{1}$H NMR spectrum of 28-membered macrocycle **7**, showing the proton signals of its minor conformers (600.13 MHz, 30 °C, DMSO-$d_6$)

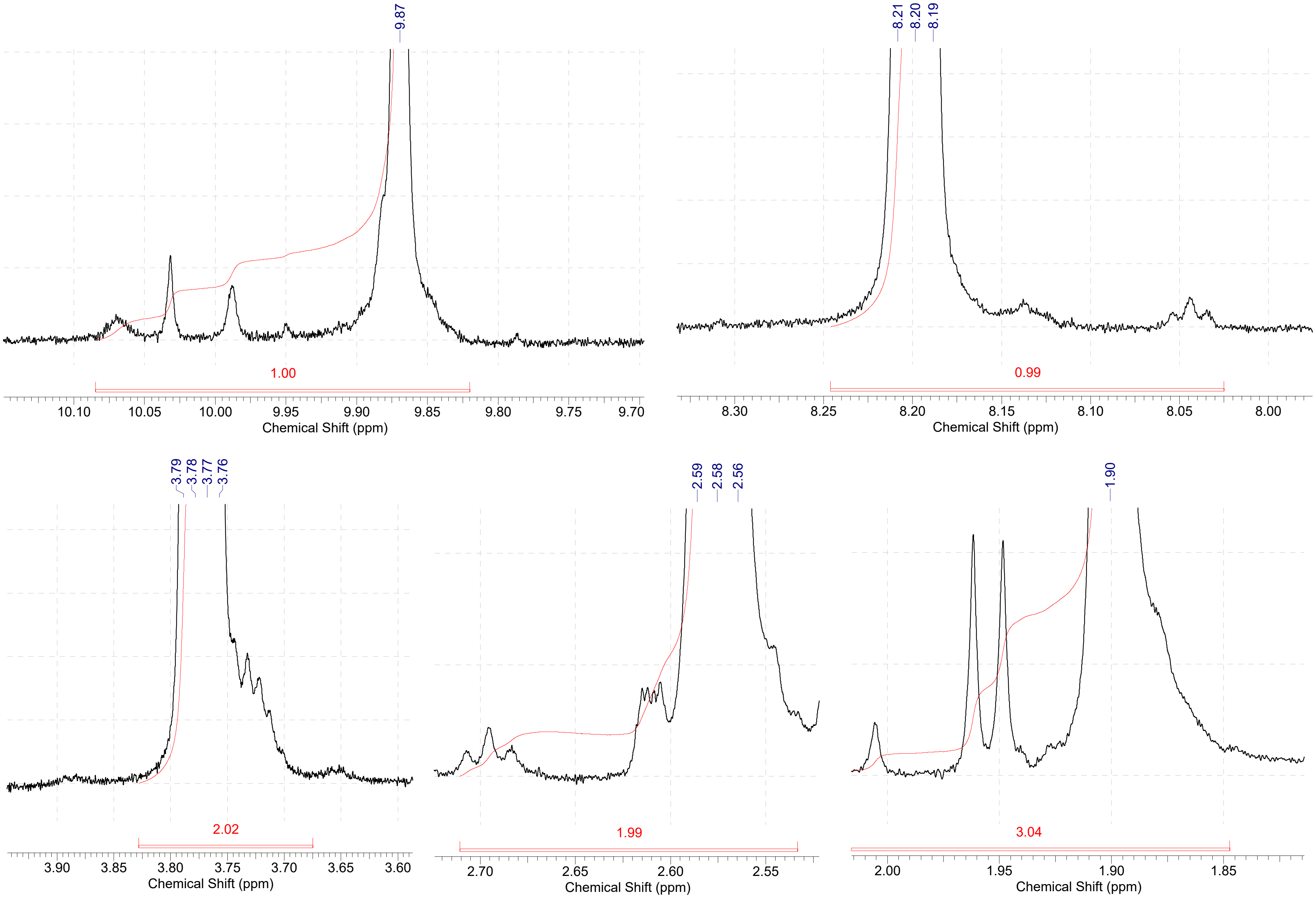

Temperature dependence of the $^{1}$H NMR spectrum of macrocycle **7** (NH proton range) (600.13 MHz, DMSO-$d_6$)
30 °C (blue) → 50 °C (red) → 60 °C (green) → 90 °C (purple) → 30 °C (yellow)

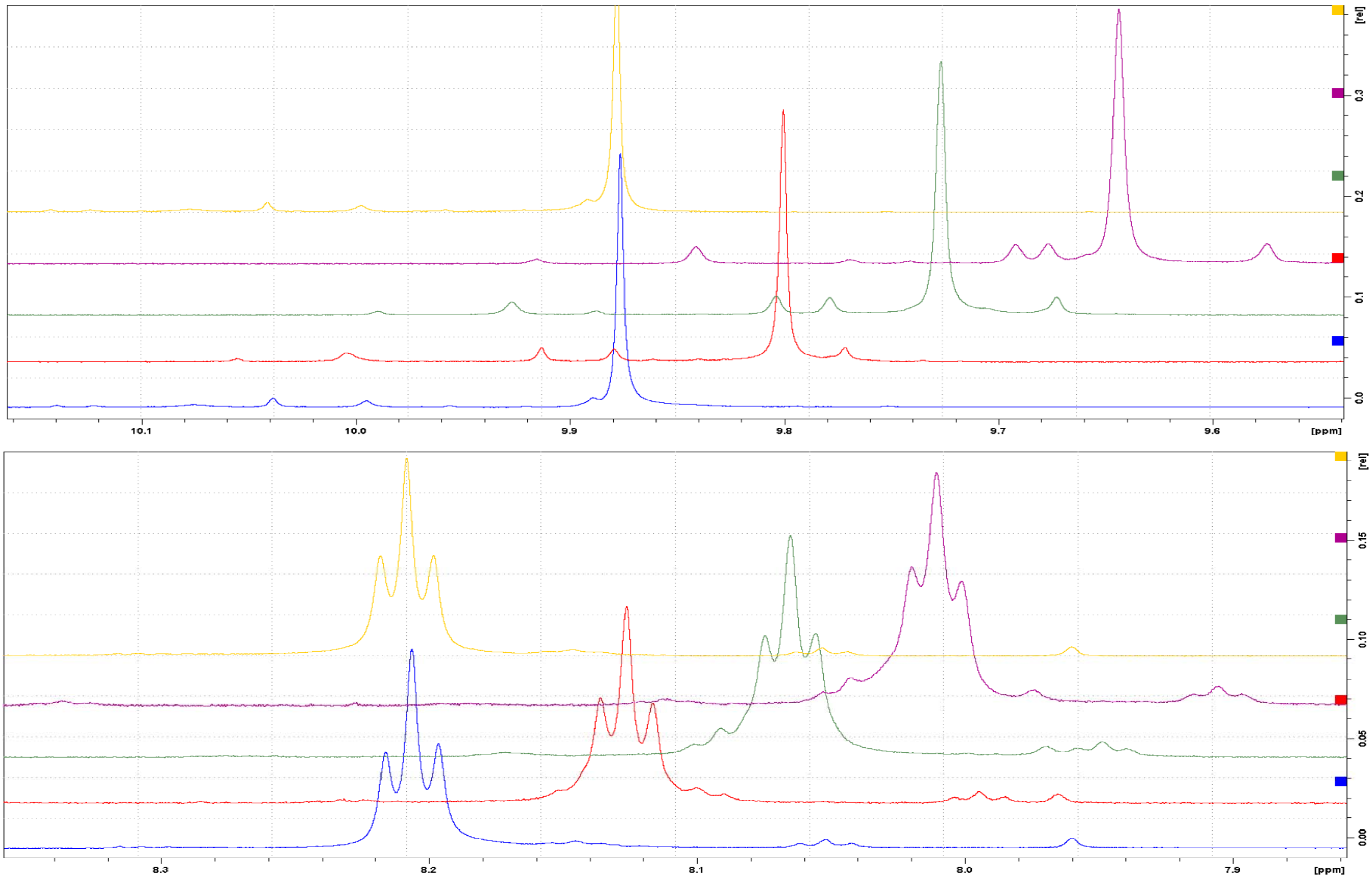

$^{13}$C{1H} NMR spectrum of 28-membered cyclic tetrakis-thiosemicarbazone **7** (150.90 MHz, DMSO-$d_6$)

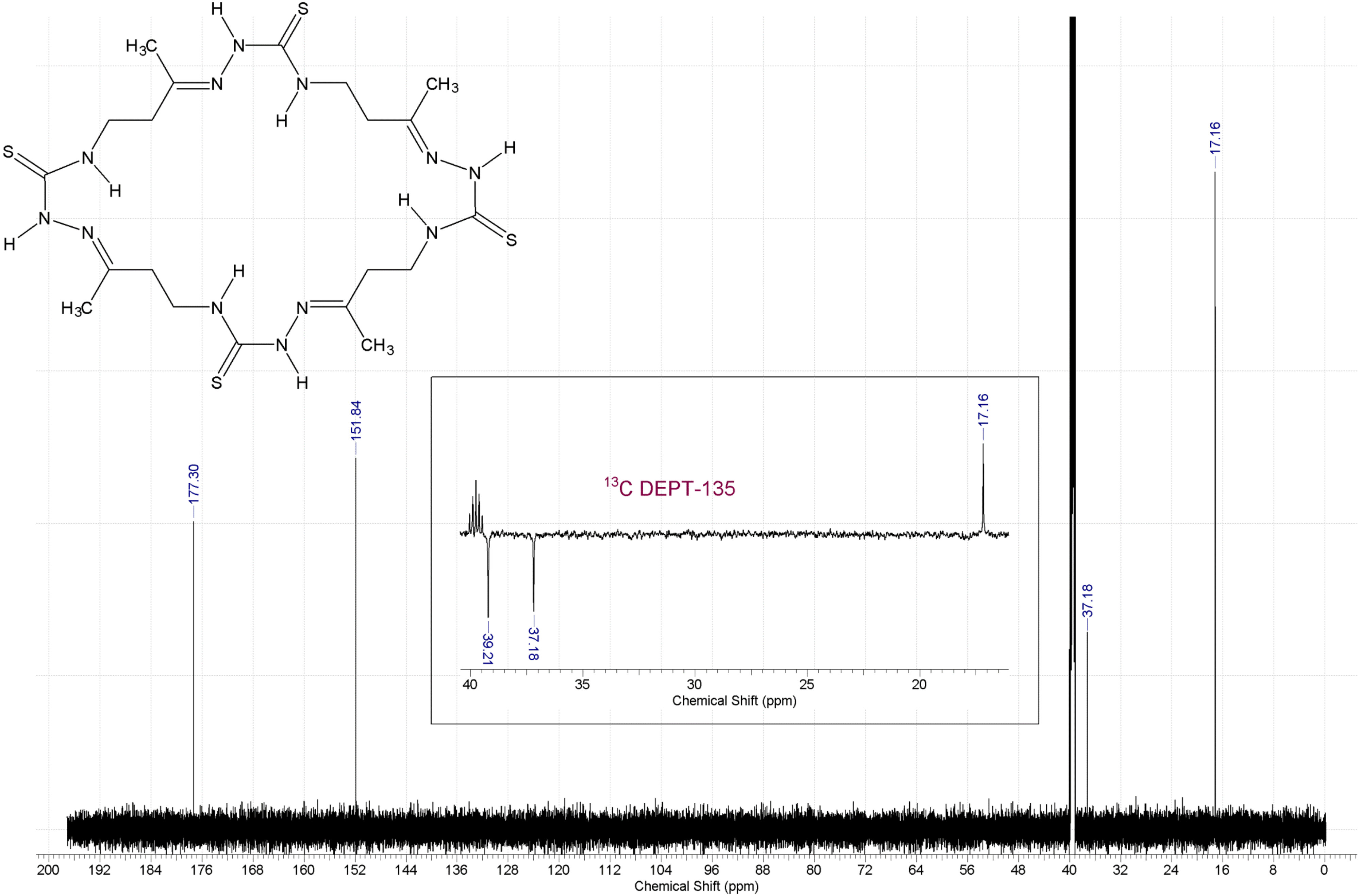

$^{1}H,^{13}C$ HSQC spectrum of 28-membered cyclic tetrakis-thiosemicarbazone **7** (Bruker Avance III, DMSO-$d_6$)

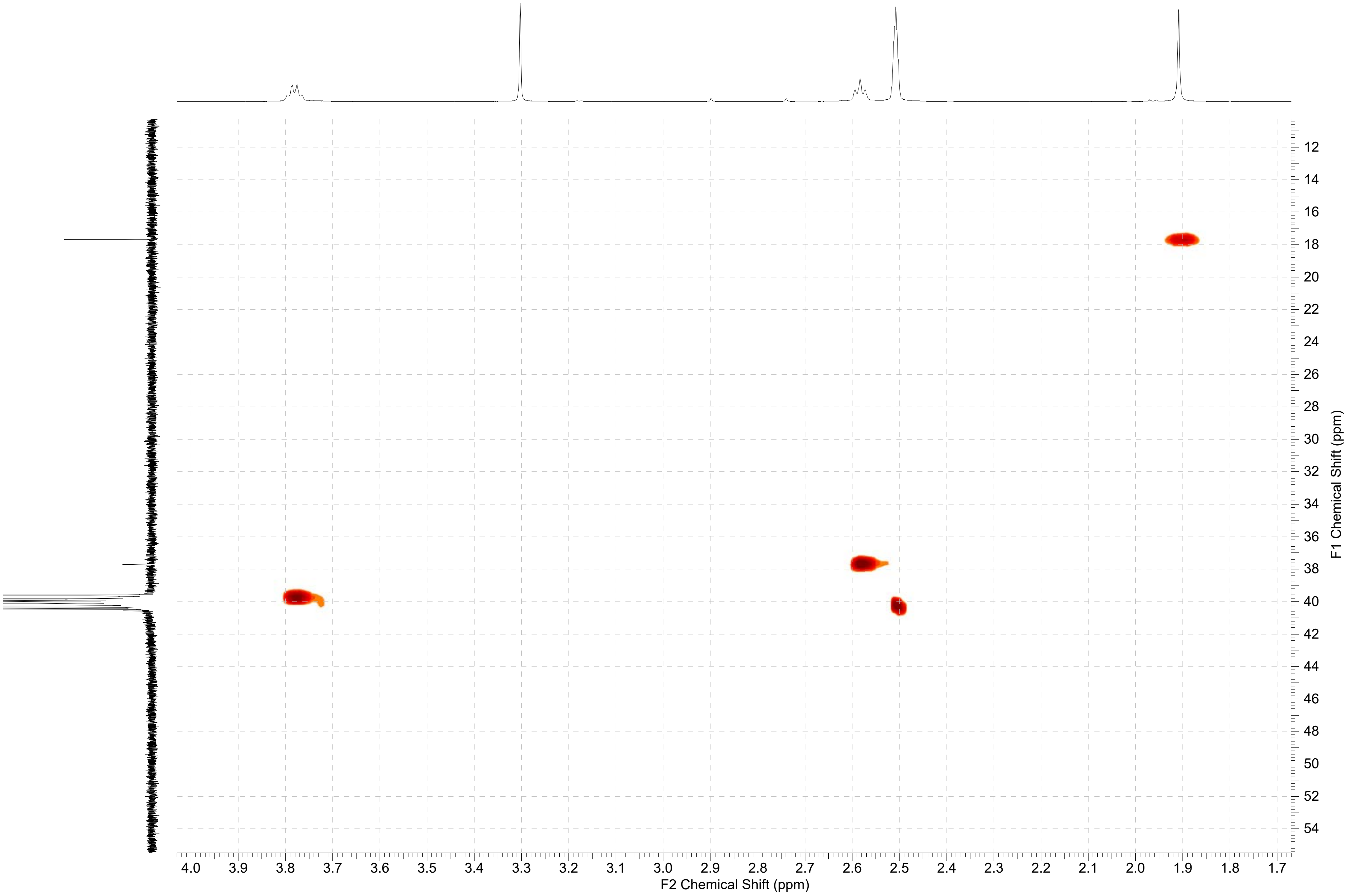

$^{1}H,^{13}C$ HMBC spectrum of 28-membered cyclic tetrakis-thiosemicarbazone **7** (Bruker Avance III, DMSO-$d_6$)

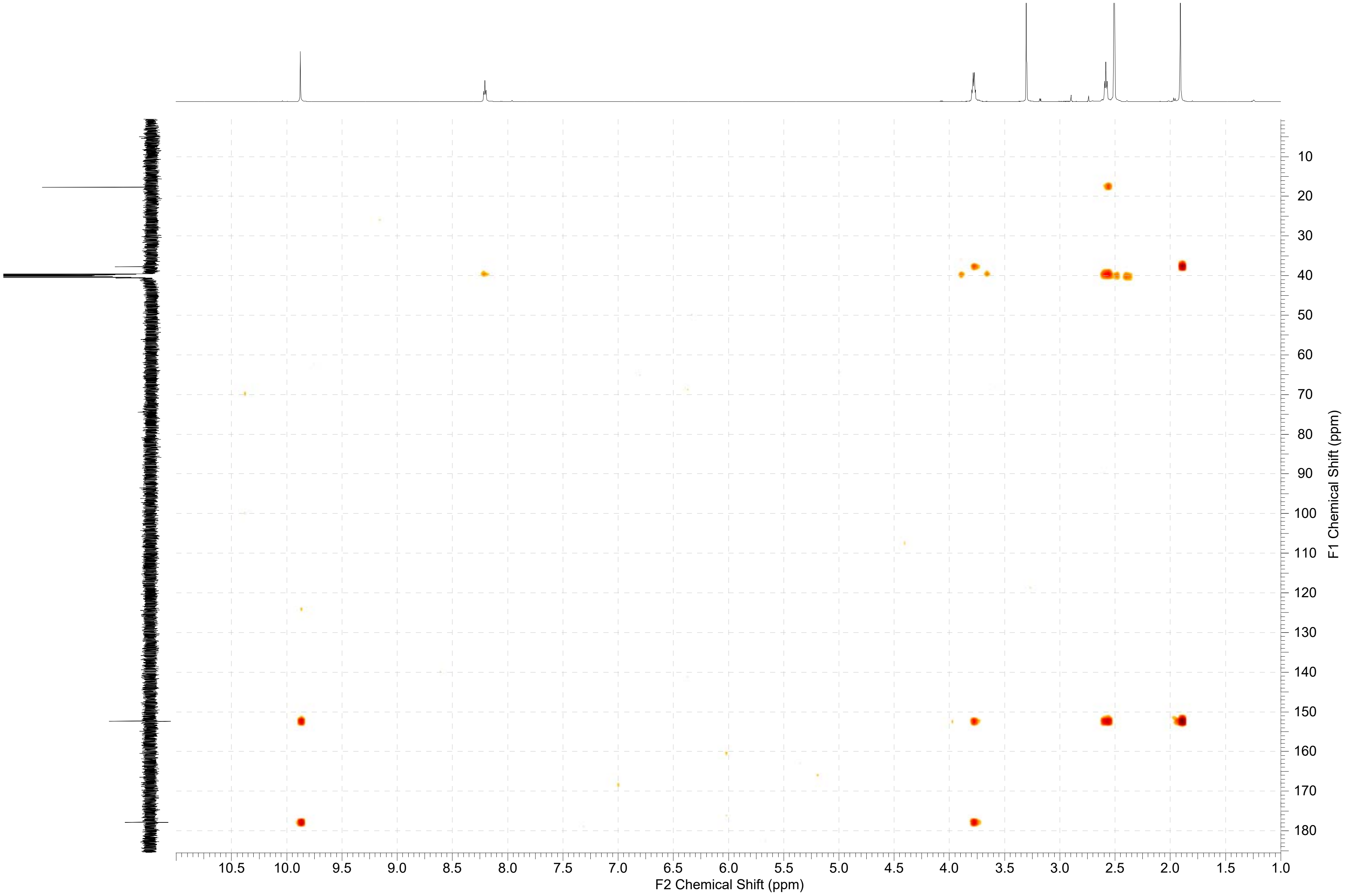

Fragment of $^{1}H,^{13}C$ HMBC spectrum of 28-membered cyclic tetrakis-thiosemicarbazone**7** (Bruker Avance III, DMSO-$d_6$)

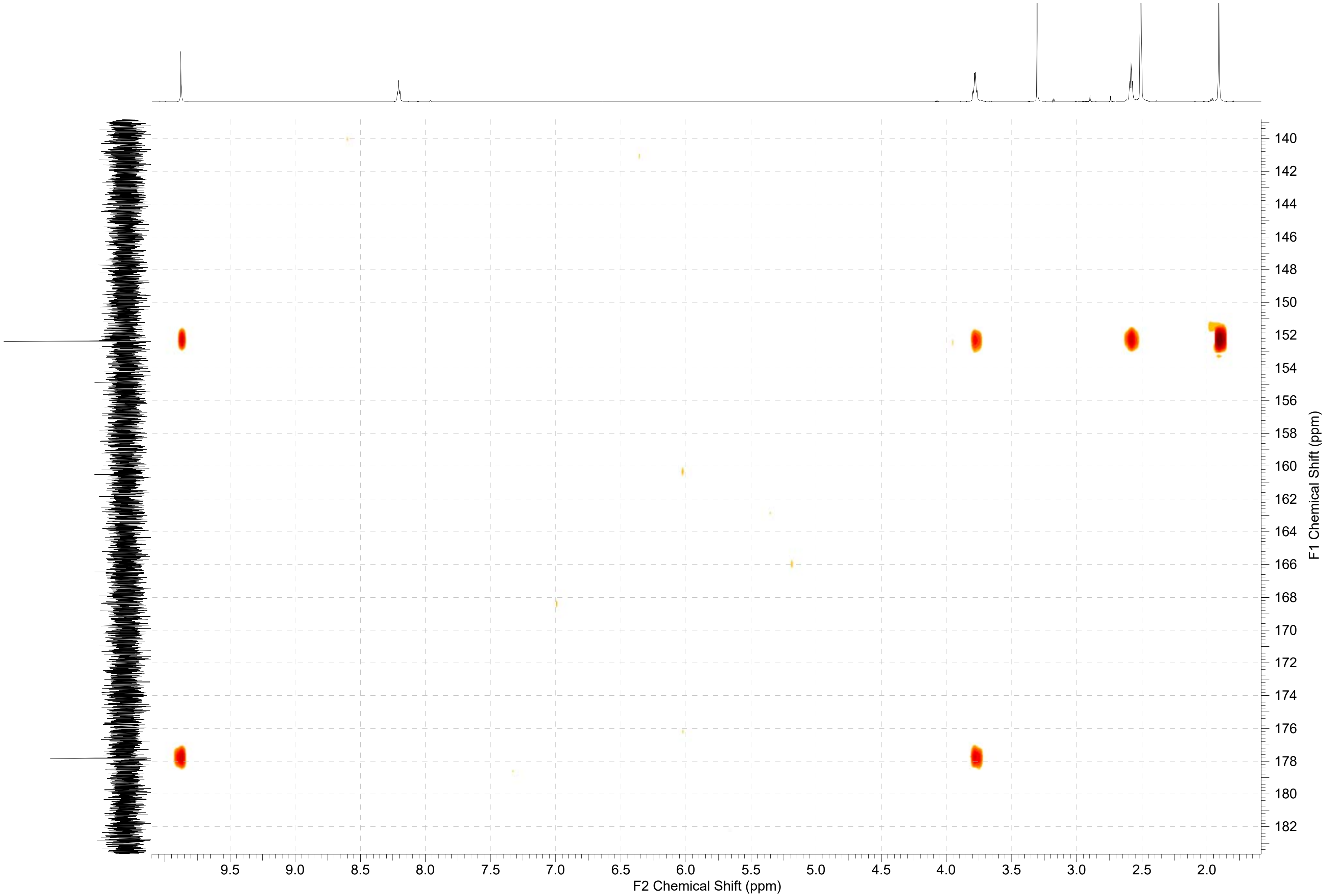

Fragment of $^{1}$H,$^{13}$C HMBC spectrum of 28-membered cyclic tetrakis-thiosemicarbazone **7** (Bruker Avance III, DMSO-$d_6$)

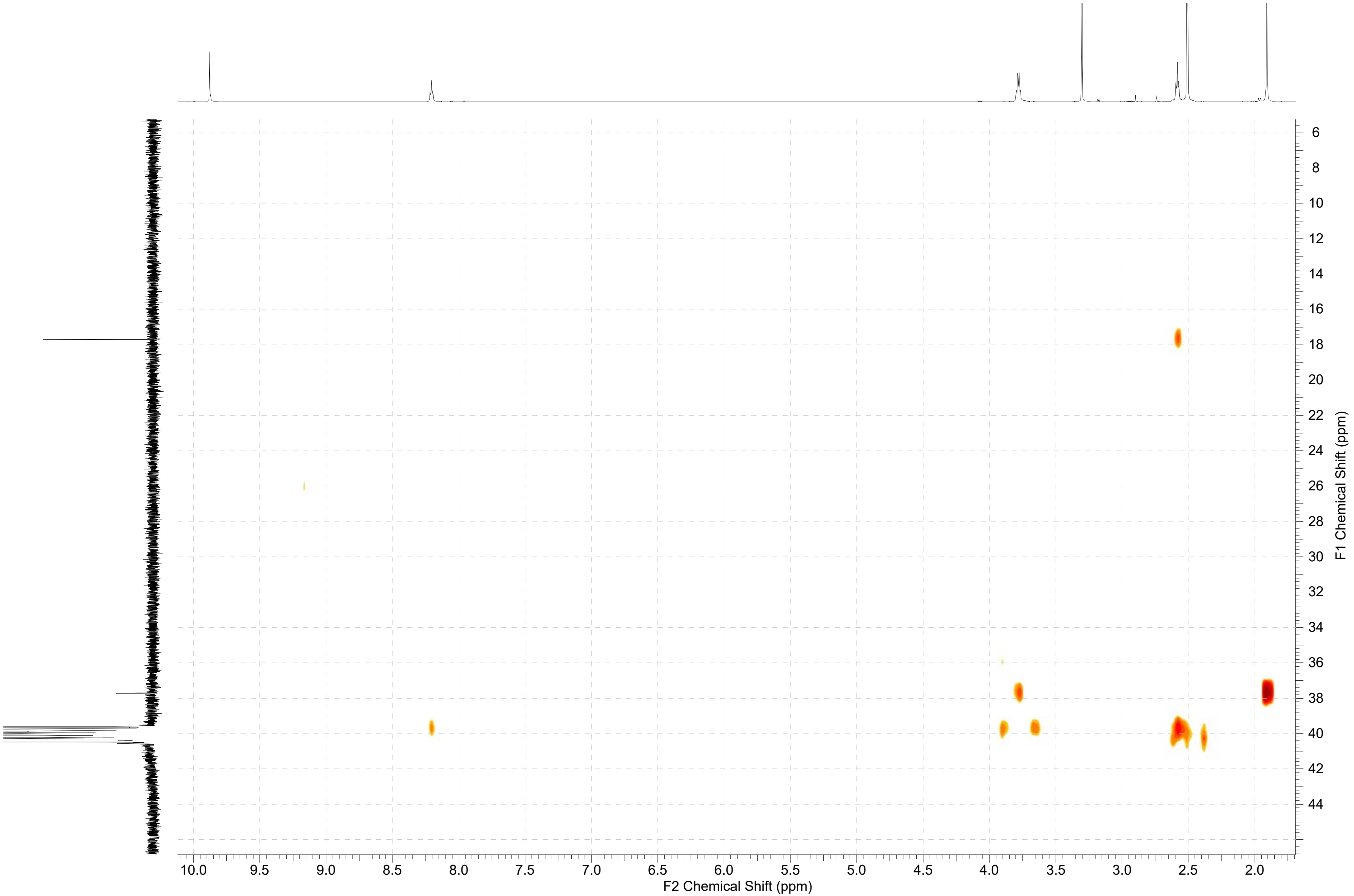

$^{1}H,^{1}H$ NOESY spectrum of 28-membered cyclic tetrakis-thiosemicarbazone **7** (Bruker Avance III, DMSO-$d_6$)

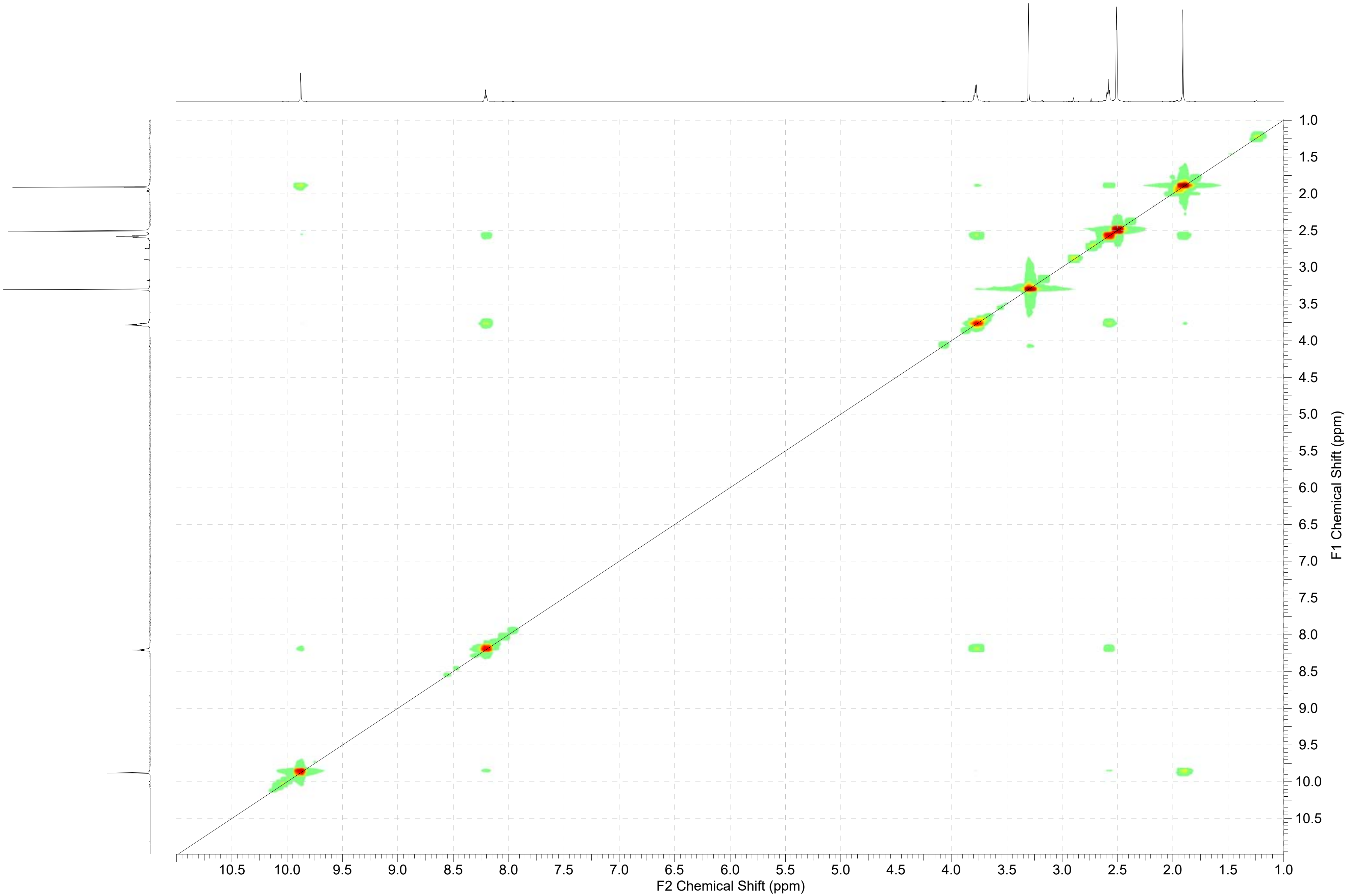

IR spectrum of compound **5** (KBr).

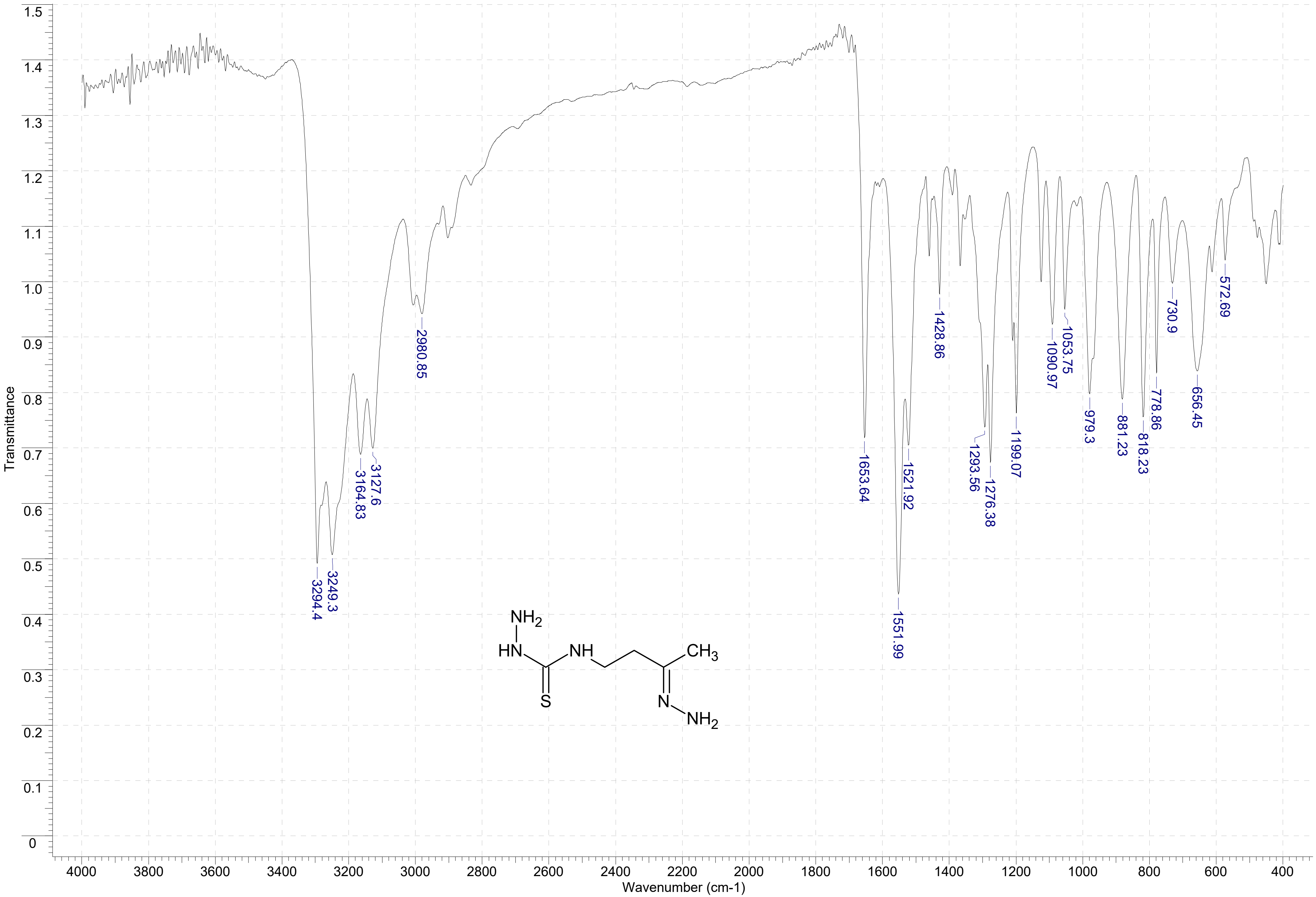

IR spectrum of compound **6** (KBr).

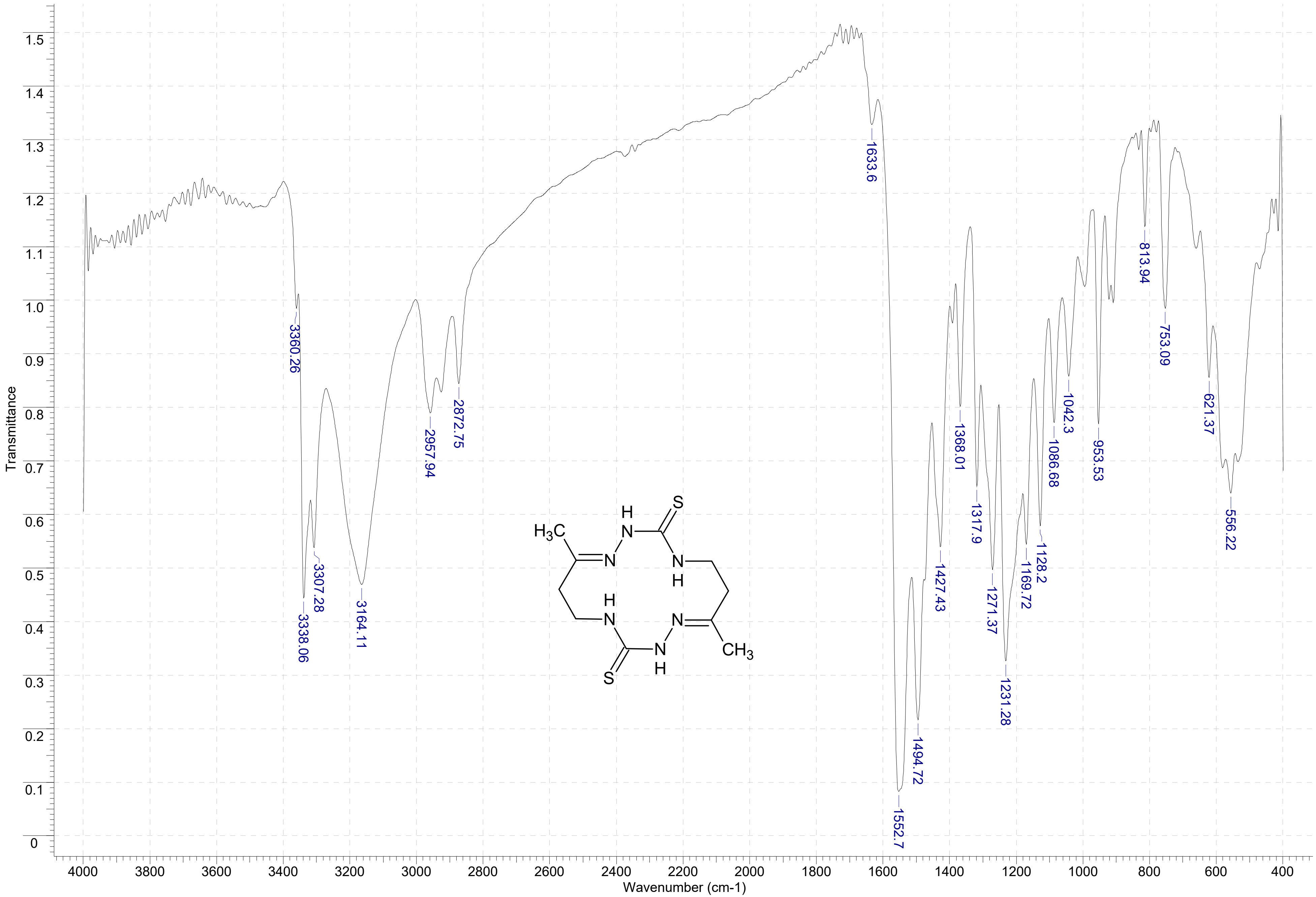

IR spectrum of compound **7** (KBr).

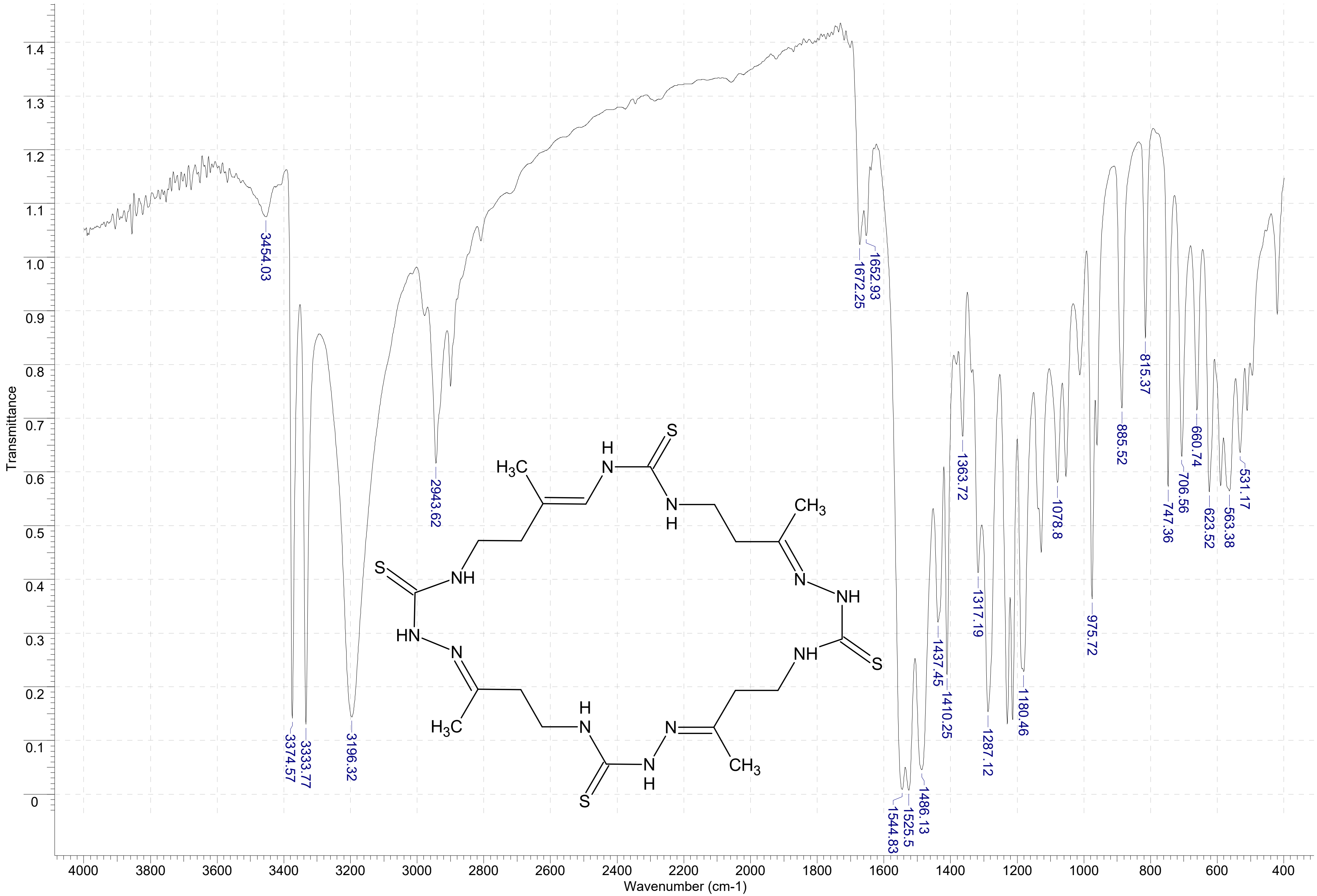

## X-ray diffraction data

X-ray diffraction experiments were carried out on a Bruker KAPPA APEX II area-detector diffractometer.[1] Unit cell parameters were refined over the whole dataset.[2] Absorption corrections were introduced using the SADABS program.[3] The structures were solved by a direct method using the SHELXS-2014 program[4] and refined by full-matrix least-squares on $F^2$ in the anisotropic approximation for all non-hydrogen atoms (SHELXL-2018[5]). The H atoms of $CH_2$ and $CH_3$ groups were placed in geometrically calculated positions with isotropic temperature factors equal to $1.2U_{eq}(C)$ for $CH_2$ and $1.5U_{eq}(C)$ for $CH_3$ groups, the orientation of $CH_3$ groups was refined. The H atoms of NH groups were objectively located from the difference Fourier synthesis and refined with isotropic temperature factors equal to $1.2U_{eq}(N)$. Crystal data, data collection and structure refinement details are summarized in Table S1.

Single crystals of macrocycle **6** suitable for X-ray crystallographic analysis were obtained by slow crystallyzation from a saturated solution in dry DMF (63.8 mg of **6** and 1.5 mL of DMF) at room temperature. Crystallization of **6** from a saturated solution in dry DMSO (10.5 mg of **6** and 3.0 mL of DMSO) gave single crystals of DMSO solvate of **6**·(**6**·2DMSO). Single crystals of DMF solvate of macrocycle **7**·(**7**·6DMF) were formed by slow crystallyzation from a saturated solution in dry DMF (11.4 mg of **7** and 1.0 mL of DMF) at room temperature.

**Table S1.** Crystallographic data and details of data collection and structure refinement.

| Compound | **6** | **6**·2DMSO | **7**·6DMF |
|---|---|---|---|
| Empirical formula | $C_{10}H_{18}N_6S_2$ | $C_{14}H_{30}N_6O_2S_4$ | $C_{38}H_{78}N_{18}O_6S_4$ |
| $M$ | 286.42 | 442.68 | 1011.42 |
| $T$, K | 100(2) | 100(2) | 100(2) |
| Crystal system | monoclinic | triclinic | triclinic |
| Space group | $P2_1/n$ | $P$–1 | $P$–1 |
| $a$, Å | 9.4407(3) | 6.3135(3) | 7.2849(3) |
| $b$, Å | 5.0543(2) | 7.6399(3) | 12.9017(5) |
| $c$, Å | 28.2388(7) | 12.1729(4) | 14.4114(5) |
| α, ° | 90 | 78.310(2) | 82.762(2) |
| β, ° | 93.706(1) | 82.314(2) | 89.570(2) |
| γ, ° | 90 | 66.644(2) | 82.465(2) |
| $V$, Å$^3$; $Z$ | 1344.63(8); 4 | 526.93(4), 1 | 1332.04(9), 1 |
| $\rho_{calc.}$, g/cm$^3$ | 1.415 | 1.395 | 1.261 |
| $\mu(MoK_\alpha)$, mm$^{-1}$ | 0.389 | 0.473 | 0.237 |

| | | | |
|---|---|---|---|
| $2\theta_{max}$, ° | 70 | 60 | 70 |
| No. of observed/independent reflections | 23024/5902 | 6310/2964 | 31848/11685 |
| No. of independent reflections with $I > 2\sigma(I)$ | 5018 | 2348 | 7077 |
| No. of refined parameters | 177 | 125 | 319 |
| $R(F)$; $wR(F^2)$ [$I > 2\sigma(I)$] | 0.0334; 0.0853 | 0.0371; 0.0849 | 0.0496; 0.1029 |
| $R(F)$; $wR(F^2)$ [all data] | 0.0410; 0.0894 | 0.0517; 0.0928 | 0.0998; 0.1216 |
| *GOOF* | 1.036 | 1.020 | 1.005 |
| $\Delta\rho_{max}$ and $\Delta\rho_{min}$, $e\cdot Å^{-3}$ | 0.490; –0.403 | 0.361; –0.451 | 0.409; –0.400 |

**Structure 6**

The molecules of **6** occupy general positions. Bond lengths and bond angles in the structure **6** are given in Tables S2-S3. The structure contains intramolecular H-bonds N-H…N (Fig. S1, Table S4). Intermolecular H-bonds N-H...S (Fig. S2, Table S4) link the molecules into the chains along the [100] direction.

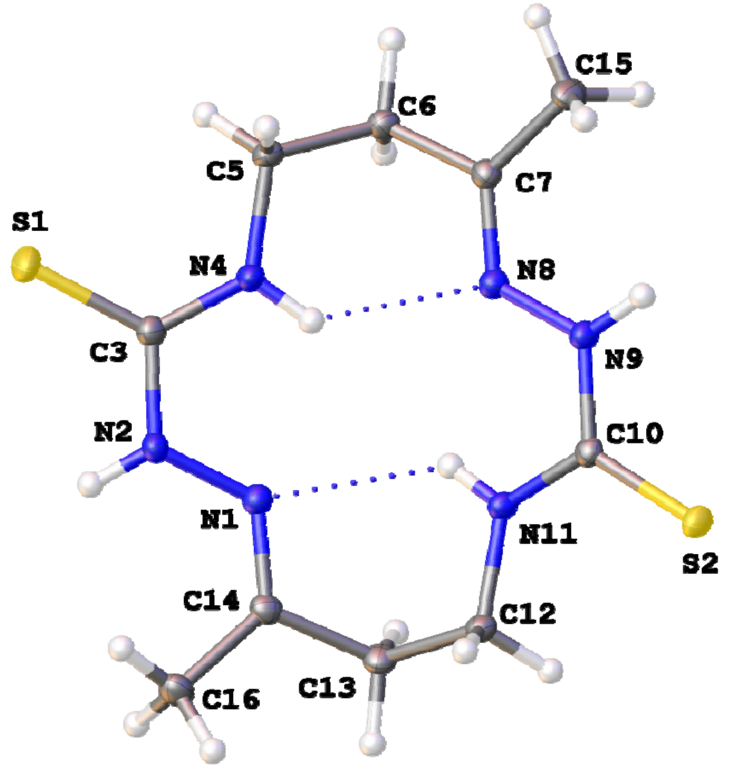


**Figure S1.** A view of **6**. Displacement ellipsoids are shown at 50% probability level. Dotted lines indicate H-bonds.

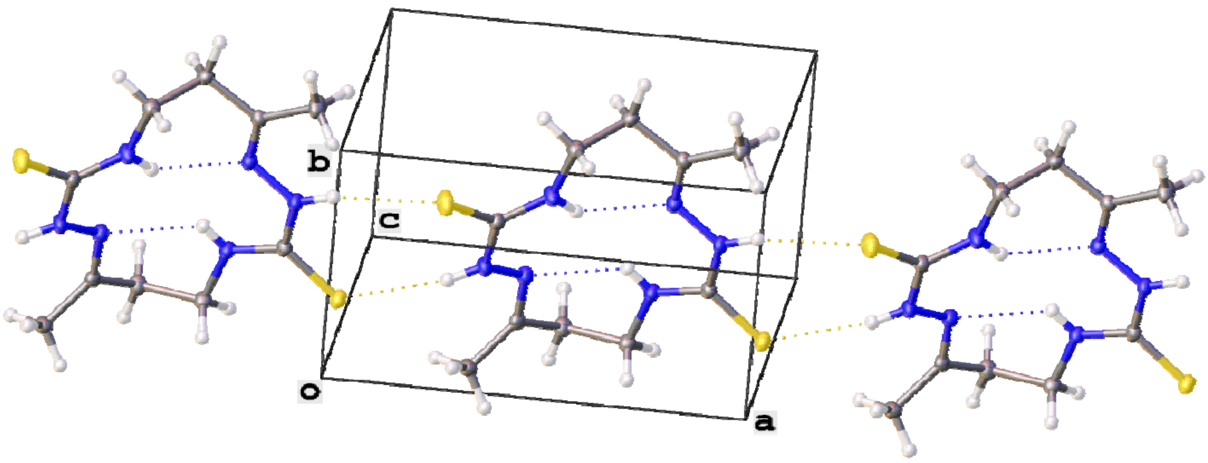


**Figure S2.** Chains formed by N-H..S bonds in the structure **6**. Dotted lines indicate H-bonds.

**Table S2.** Bond lengths for **6**.

| Atom | Atom | Length/Å |
|---|---|---|
| S1 | C3 | 1.6808(9) |
| S2 | C10 | 1.6844(9) |
| N1 | N2 | 1.3875(11) |
| N1 | C14 | 1.2872(11) |
| N2 | C3 | 1.3684(12) |
| N4 | C3 | 1.3286(12) |
| N4 | C5 | 1.4563(12) |
| N8 | N9 | 1.3843(11) |
| N8 | C7 | 1.2870(11) |

| Atom | Atom | Length/Å |
|---|---|---|
| N9 | C10 | 1.3597(11) |
| N11 | C10 | 1.3389(11) |
| N11 | C12 | 1.4558(11) |
| C5 | C6 | 1.5297(13) |
| C6 | C7 | 1.5151(12) |
| C7 | C15 | 1.4918(13) |
| C12 | C13 | 1.5325(13) |
| C13 | C14 | 1.5159(13) |
| C14 | C16 | 1.4978(13) |

**Table S3.** Bond angles for **6**.

| Atom | Atom | Atom | Angle/° |
|---|---|---|---|
| C14 | N1 | N2 | 119.03(8) |
| C3 | N2 | N1 | 117.96(7) |
| C3 | N4 | C5 | 125.65(8) |
| C7 | N8 | N9 | 119.08(8) |
| C10 | N9 | N8 | 118.07(7) |
| C10 | N11 | C12 | 124.80(8) |
| N2 | C3 | S1 | 119.85(7) |
| N4 | C3 | S1 | 124.13(7) |
| N4 | C3 | N2 | 116.01(8) |
| N4 | C5 | C6 | 109.29(7) |
| C7 | C6 | C5 | 114.43(8) |

| Atom | Atom | Atom | Angle/° |
|---|---|---|---|
| N8 | C7 | C6 | 115.74(8) |
| N8 | C7 | C15 | 125.46(8) |
| C15 | C7 | C6 | 118.80(8) |
| N9 | C10 | S2 | 121.21(7) |
| N11 | C10 | S2 | 122.87(7) |
| N11 | C10 | N9 | 115.92(8) |
| N11 | C12 | C13 | 109.59(7) |
| C14 | C13 | C12 | 114.31(7) |
| N1 | C14 | C13 | 115.93(8) |
| N1 | C14 | C16 | 126.23(9) |
| C16 | C14 | C13 | 117.85(8) |

**Table S4.** Hydrogen bonds for **6**.

| D | H | A | d(D-H)/Å | d(H-A)/Å | d(D-A)/Å | D-H-A/° |
|---|---|---|---|---|---|---|
| N2 | H2 | $S2^1$ | 0.870(14) | 2.555(14) | 3.3954(8) | 162.8(12) |
| N4 | H4 | N8 | 0.869(14) | 2.182(14) | 2.8434(11) | 132.7(12) |
| N9 | H9 | $S1^2$ | 0.885(13) | 2.779(13) | 3.6505(8) | 168.3(12) |
| N11 | H11 | N1 | 0.818(14) | 2.296(14) | 2.9034(11) | 131.5(12) |

Symmetry transformations: 1 – (-1+*x*, *y*, *z*); 2 – (1+*x*, *y*, *z*).

## Structure 6·2DMSO

The structure contains a centrosymmetric molecule of **6** and one crystallographically independent DMSO solvate molecule (Fig. S3). Bond lengths and bond angles in the structure **6** are given in Tables S5-S6. Similar to the previous structure, the present one contains intramolecular H-bonds N-H…N (Fig. S3, Table S7). Solvate DMSO molecules are linked with the molecule of **6** by H-bonds N-H...O.

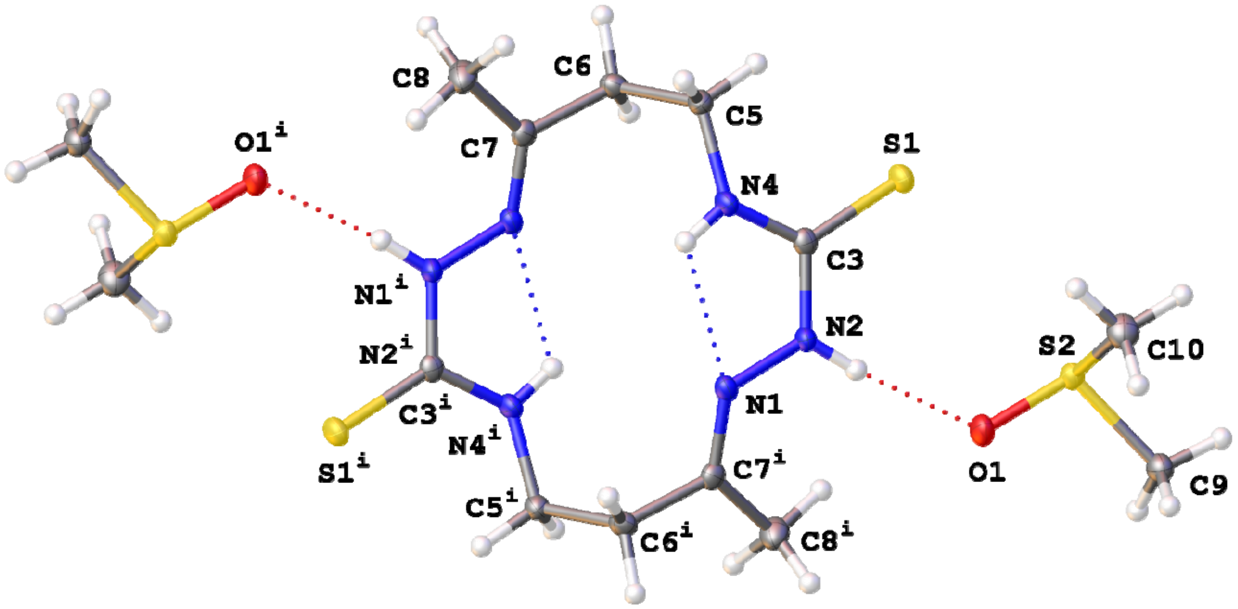


**Figure S3.** A view of **6**·2DMSO. Displacement ellipsoids are shown at 50% probability level. Dotted lines indicate H-bonds. Symmetry transformation: i – (1-*x*, 1-*y*, 1-*z*).

**Table S5.** Bond lengths for **6**·2DMSO.

| Atom | Atom | Length/Å |
|---|---|---|
| S1 | C3 | 1.6883(16) |
| S2 | O1 | 1.5072(13) |
| S2 | C9 | 1.7858(18) |
| S2 | C10 | 1.7872(18) |
| N1 | N2 | 1.3869(19) |
| N1 | C7$^{i}$ | 1.286(2) |

| Atom | Atom | Length/Å |
|---|---|---|
| N2 | C3 | 1.357(2) |
| N4 | C3 | 1.334(2) |
| N4 | C5 | 1.449(2) |
| C5 | C6 | 1.535(2) |
| C6 | C7 | 1.502(2) |
| C7 | C8 | 1.499(2) |

Symmetry transformation: i – (1-*x*, 1-*y*, 1-*z*).

**Table S6.** Bond angles for **6**·2DMSO.

| Atom | Atom | Atom | Angle/° |
|---|---|---|---|
| O1 | S2 | C9 | 104.62(8) |
| O1 | S2 | C10 | 106.16(8) |
| C9 | S2 | C10 | 97.28(9) |
| C7$^{1}$ | N1 | N2 | 118.59(14) |
| C3 | N2 | N1 | 117.53(14) |
| C3 | N4 | C5 | 125.84(15) |
| N2 | C3 | S1 | 119.49(12) |

| Atom | Atom | Atom | Angle/° |
|---|---|---|---|
| N4 | C3 | S1 | 124.83(14) |
| N4 | C3 | N2 | 115.68(15) |
| N4 | C5 | C6 | 110.47(14) |
| C7 | C6 | C5 | 111.63(13) |
| N1$^{i}$ | C7 | C6 | 115.57(15) |
| N1$^{i}$ | C7 | C8 | 126.95(16) |
| C8 | C7 | C6 | 117.40(15) |

Symmetry transformation: i – (1-*x*, 1-*y*, 1-*z*).

**Table S7.** Hydrogen bonds for **6**·2DMSO.

| D | H | A | d(D-H)/Å | d(H-A)/Å | d(D-A)/Å | D-H-A/° |
|---|---|---|---|---|---|---|
| N4 | H4 | N1 | 0.81(2) | 2.15(2) | 2.577(2) | 113.4(18) |
| N2 | H2 | O1 | 0.85(2) | 1.95(2) | 2.7859(19) | 169.3(19) |

Conformations of the heterocyclic ring in **6** and in **6·2DMSO** are different. As it is seen from Table S8, the difference is observed for torsion angles around N4-C5, C5-C6 and C6-C7 (and corresponding N11-C12, C12-C13 and C14-C14 for **6**) bonds.

**Table S8.** Comparison of corresponding torsion angles for **6**·2DMSO and **6**.

| **6**·2DMSO | | **6** | | | |
|---|---|---|---|---|---|
| Atoms | Angle/° | Atoms | Angle/° | Atoms | Angle/° |
| C7$^{i}$N1N2C3 | 162.95(14) | C14N1N2C3 | 173.60(8) | C7N8N9C10 | 178.27(8) |
| N1N2C3N4 | -1.0(2) | N1N2C3N4 | -8.83(12) | N8N9C10N11 | -1.10(12) |

| | | | | | |
|---|---|---|---|---|---|
| N2C3N4C5 | -166.47(14) | N2C3N4C5 | -177.02(9) | N9C10N11C12 | -170.18(8) |
| C3N4C5C6 | 108.34(17) | C3N4C5C6 | -163.38(9) | C10N11C12C13 | -176.81(8) |
| N4C5C6C7 | 66.30(18) | N4C5C6C7 | -63.34(10) | N11C12C13C14 | -65.51(10) |
| C5C6C7N1$^i$ | -89.27(17) | C5C6C7N8 | 65.22(11) | C12C13C14N1 | 67.64(11) |
| C6C7N1$^i$N2$^i$ | 177.50(0.13) | C6C7N8N9 | -179.19(8) | C13C14N1N2 | 178.85(8) |

Symmetry transformation: i – (1-*x*, 1-*y*, 1-*z*).

**Structure 7·6DMF**

The structure contains a centrosymmetric molecule of **7** (Fig. S4) and three crystallographically independent DMF solvate molecules. Bond lengths and bond angles in the structure **7**·6DMF are given in Tables S9-S10. Two of four $CH_3$ groups are disordered. The molecule of  contains weak intramolecular H-bonds C-H…S (Fig. S4, Table S11). Solvate DMF molecules are linked with the molecule of **7** by H-bonds of different types (Fig. S5, Table S11). All NH groups of **7** are involved into H-bonding with solvate DMF molecules, thus, there are no intramolecular N-H…N bonds.

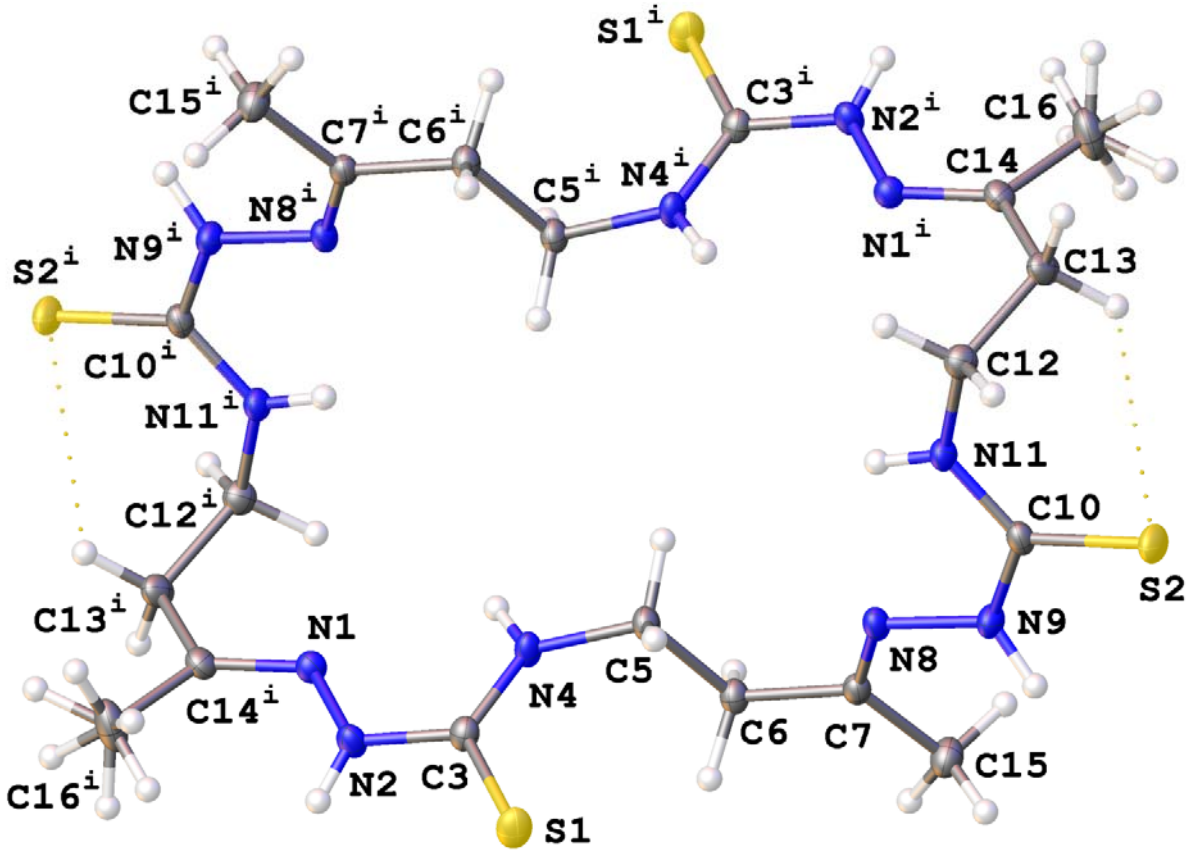


**Figure S4.** A view of molecule **7** in the structure **7**·6DMF. Displacement ellipsoids are shown at 50% probability level. Dotted lines indicate H-bonds. Symmetry transformation: i – (1-*x*, 1-*y*, 1-*z*). The $CH_3$ groups at the C14 and C14$^i$ are disordered over two positions with occupancy ratio of 0.67:0.33

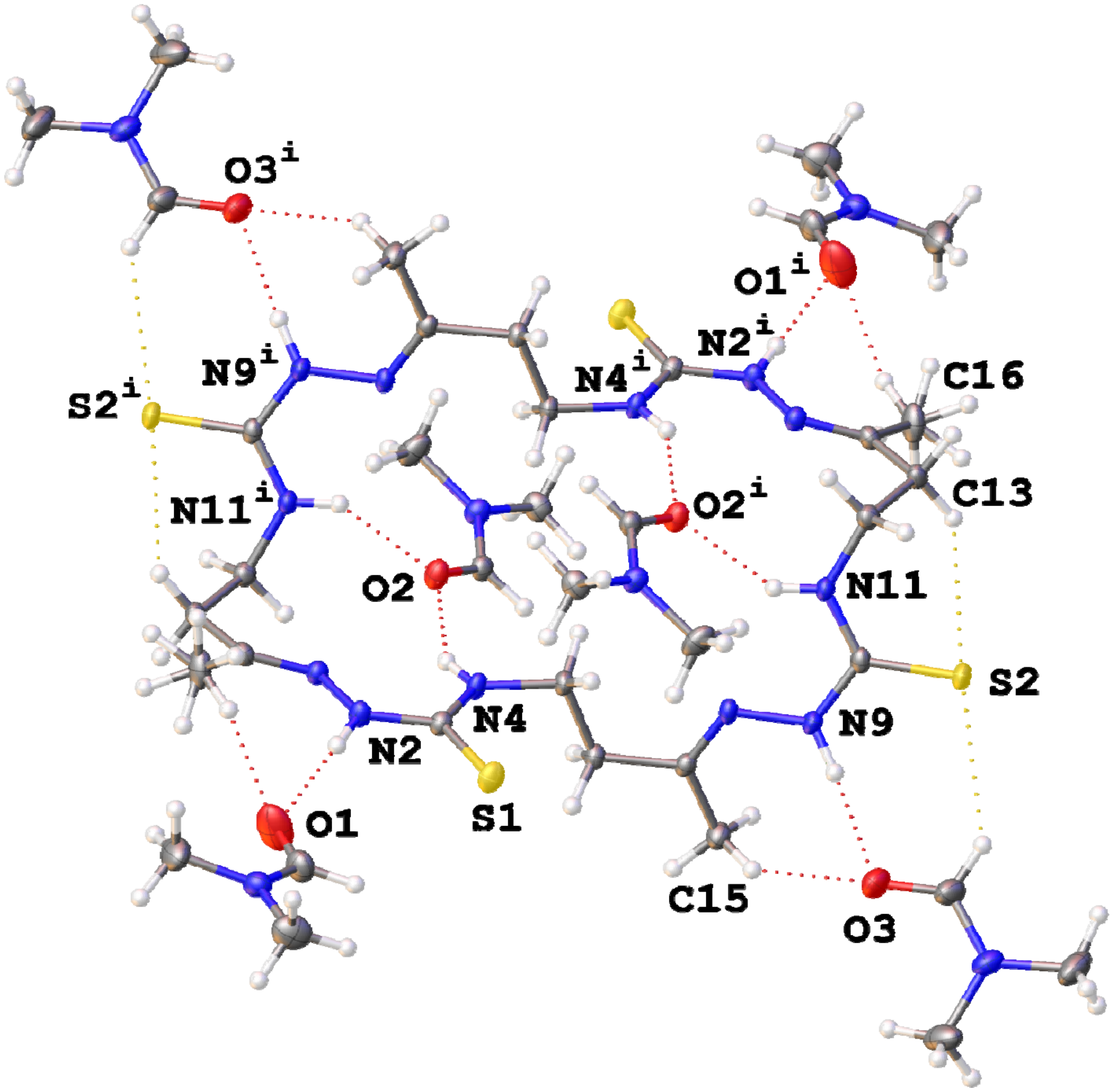


**Figure S5.** H-bonds in the structure **7**·6DMF. Symmetry transformation: i – (1-*x*, 1-*y*, 1-*z*).

**Table S9.** Bond lengths for **7**·6DMF.

| Atom | Atom | Length/Å | Atom | Atom | Length/Å |
|---|---|---|---|---|---|
| S1 | C3 | 1.6950(13) | N21 | C17 | 1.3294(19) |
| S2 | C10 | 1.6935(12) | N21 | C18 | 1.4522(18) |
| O1 | C17 | 1.223(2) | N21 | C19 | 1.445(2) |
| O2 | C20 | 1.2325(15) | N22 | C20 | 1.3310(17) |
| O3 | C23 | 1.2377(17) | N22 | C21 | 1.4536(17) |
| N1 | N2 | 1.3910(15) | N22 | C22 | 1.4555(16) |
| N1 | $C14^{i}$ | 1.2840(15) | N23 | C23 | 1.3244(19) |
| N2 | C3 | 1.3565(15) | N23 | C24 | 1.451(2) |

| Atom | Atom | Length/Å |
|---|---|---|
| N4 | C3 | 1.3314(17) |
| N4 | C5 | 1.4571(15) |
| N8 | N9 | 1.3978(14) |
| N8 | C7 | 1.2828(17) |
| N9 | C10 | 1.3621(17) |
| N11 | C10 | 1.3316(16) |
| N11 | C12 | 1.4597(17) |

| Atom | Atom | Length/Å |
|---|---|---|
| N23 | C25 | 1.447(2) |
| C5 | C6 | 1.5234(17) |
| C6 | C7 | 1.5049(16) |
| C7 | C15 | 1.5054(18) |
| C12 | C13 | 1.5226(18) |
| C13 | C14 | 1.4984(19) |
| C14 | C16 | 1.5003(19) |

Symmetry transformation: i – (1-*x*, 1-*y*, 1-*z*).

**Table S10.** Bond angles for **7**·6DMF.

| Atom | Atom | Atom | Angle/° |
|---|---|---|---|
| C14$^{i}$ | N1 | N2 | 117.03(11) |
| C3 | N2 | N1 | 118.79(11) |
| C3 | N4 | C5 | 123.21(11) |
| C7 | N8 | N9 | 116.13(10) |
| C10 | N9 | N8 | 118.28(10) |
| C10 | N11 | C12 | 123.95(10) |
| C17 | N21 | C18 | 120.91(14) |
| C17 | N21 | C19 | 121.10(13) |
| C19 | N21 | C18 | 117.93(14) |
| C20 | N22 | C21 | 122.28(11) |
| C20 | N22 | C22 | 121.45(11) |
| C21 | N22 | C22 | 116.24(12) |
| C23 | N23 | C24 | 120.94(13) |
| C23 | N23 | C25 | 122.00(14) |
| C25 | N23 | C24 | 117.00(14) |
| N2 | C3 | S1 | 118.40(10) |
| N4 | C3 | S1 | 124.04(9) |

| Atom | Atom | Atom | Angle/° |
|---|---|---|---|
| N4 | C3 | N2 | 117.51(11) |
| N4 | C5 | C6 | 111.66(10) |
| C7 | C6 | C5 | 115.12(11) |
| N8 | C7 | C6 | 117.84(11) |
| N8 | C7 | C15 | 125.57(11) |
| C6 | C7 | C15 | 116.57(11) |
| N9 | C10 | S2 | 119.01(10) |
| N11 | C10 | S2 | 123.76(10) |
| N11 | C10 | N9 | 117.20(11) |
| N11 | C12 | C13 | 112.75(11) |
| C14 | C13 | C12 | 116.05(10) |
| N1$^{i}$ | C14 | C13 | 117.31(11) |
| N1$^{i}$ | C14 | C16 | 126.23(13) |
| C13 | C14 | C16 | 116.46(11) |
| O1 | C17 | N21 | 124.72(15) |
| O2 | C20 | N22 | 125.88(12) |
| O3 | C23 | N23 | 125.25(16) |

Symmetry transformation: i – (1-*x*, 1-*y*, 1-*z*).

**Table S11.** Hydrogen bonds for **7**·6DMF.

| D | H | A | d(D-H)/Å | d(H-A)/Å | d(D-A)/Å | D-H-A/° |
|---|---|---|---|---|---|---|
| N2 | H2 | O1 | 0.813(17) | 2.025(18) | 2.8353(17) | 174.6(16) |
| N4 | H4 | O2 | 0.838(17) | 2.203(17) | 2.9448(15) | 147.6(14) |
| N9 | H9 | O3 | 0.902(16) | 1.997(17) | 2.8917(16) | 171.4(14) |
| N11 | H11 | $O2^{i}$ | 0.849(15) | 2.188(15) | 2.8737(13) | 137.7(14) |
| C13 | H13B | S2 | 0.99 | 2.80 | 3.4254(14) | 122.1 |
| C15 | H15B | O3 | 0.98 | 2.40 | 3.2670(18) | 147.4 |
| C16 | H16D | $O1^{i}$ | 0.98 | 2.15 | 3.086(2) | 158.6 |
| C23 | H23A | S2 | 0.95 | 2.80 | 3.5696(17) | 138.5 |

Symmetry transformation: i – (1-*x*, 1-*y*, 1-*z*).

## Computational details

The geometry optimizations were carried out at the B3LYP level of theory using Gaussian 16 suite[6] of quantum chemical programs. Pople's basis sets, 6-311++G(d,p), was employed for geometry optimization. The effect of continuum solvation was incorporated by using the polarizable continuum model (PCM). Enthalpies and Gibbs free energies were obtained by adding unscaled zero-point vibrational energy corrections (ZPVE) and thermal contributions to the energies (temperature 298.150 Kelvin, pressure 1.000 atm).

### Starting compound – hydrazone 5

DMSO solution

*Computational data for various conformers of hydrazone (E)-***5**

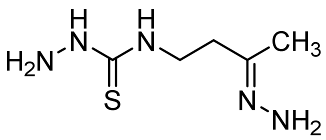

**Data 1:** Cartesian coordinates and energies of the optimized geometry for the conformer **A** of *(E)*-**5** in DMSO solution.

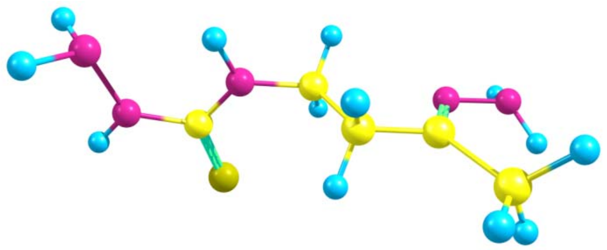

| | |
|---|---|
| Electronic Energy = | -870.449596380 a.u. |
| Zero-point correction= | 0.201422 (Hartree/Particle) |
| Thermal correction to Energy= | 0.214960 |
| Thermal correction to Enthalpy= | 0.215904 |
| Thermal correction to Gibbs Free Energy= | 0.159528 |
| Sum of electronic and zero-point Energies= | -870.248174 |
| Sum of electronic and thermal Energies= | -870.234637 |
| Sum of electronic and thermal Enthalpies= | -870.233693 |
| Sum of electronic and thermal Free Energies= | -870.290069 |

Standard orientation:

| Center Number | Atomic Number | Atomic Type | Coordinates (Angstroms) X | Y | Z |
|---|---|---|---|---|---|
| 1 | 7 | 0 | 3.663818 | -1.637115 | -0.209934 |
| 2 | 7 | 0 | 3.407946 | -0.295808 | 0.118279 |
| 3 | 6 | 0 | 2.172649 | 0.245910 | -0.044739 |
| 4 | 16 | 0 | 1.934054 | 1.894991 | 0.342078 |
| 5 | 7 | 0 | 1.228739 | -0.578265 | -0.511044 |
| 6 | 6 | 0 | -0.175659 | -0.250040 | -0.735843 |

| | | | | | |
|---|---|---|---|---|---|
| 7 | 6 | 0 | -1.044963 | -0.592195 | 0.476951 |
| 8 | 6 | 0 | -2.518631 | -0.313387 | 0.306650 |
| 9 | 7 | 0 | -2.930636 | 0.156068 | -0.811818 |
| 10 | 6 | 0 | -3.421442 | -0.619736 | 1.476058 |
| 11 | 7 | 0 | -4.299865 | 0.352466 | -0.978006 |
| 12 | 1 | 0 | 4.016904 | -2.127963 | 0.606344 |
| 13 | 1 | 0 | 4.360016 | -1.685104 | -0.948632 |
| 14 | 1 | 0 | 4.148841 | 0.312159 | 0.439295 |
| 15 | 1 | 0 | 1.540997 | -1.529858 | -0.676534 |
| 16 | 1 | 0 | -4.442142 | 1.030540 | -1.715654 |
| 17 | 1 | 0 | -4.791136 | 0.652813 | -0.138022 |
| 18 | 1 | 0 | -0.506463 | -0.809994 | -1.611345 |
| 19 | 1 | 0 | -0.247539 | 0.809821 | -0.972173 |
| 20 | 1 | 0 | -0.919844 | -1.653078 | 0.730741 |
| 21 | 1 | 0 | -0.681391 | -0.035232 | 1.349206 |
| 22 | 1 | 0 | -4.224148 | -1.303201 | 1.179130 |
| 23 | 1 | 0 | -2.864235 | -1.075336 | 2.294533 |
| 24 | 1 | 0 | -3.896476 | 0.289844 | 1.863068 |

**Data 2:** Cartesian coordinates and energies of the optimized geometry for the conformer **B** of *(E)*-**5** in DMSO solution.

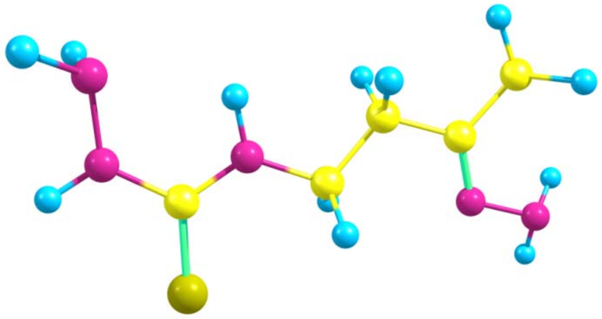

| | |
|---|---|
| Electronic Energy = | -870.448931076 a.u. |
| Zero-point correction= | 0.201132 (Hartree/Particle) |
| Thermal correction to Energy= | 0.214855 |
| Thermal correction to Enthalpy= | 0.215799 |
| Thermal correction to Gibbs Free Energy= | 0.158573 |
| Sum of electronic and zero-point Energies= | -870.247799 |
| Sum of electronic and thermal Energies= | -870.234076 |
| Sum of electronic and thermal Enthalpies= | -870.233132 |
| Sum of electronic and thermal Free Energies= | -870.290358 |

Standard orientation:

| Center Number | Atomic Number | Atomic Type | Coordinates (Angstroms) X | Y | Z |
|---|---|---|---|---|---|
| 1 | 7 | 0 | -3.352477 | -1.942848 | -0.006061 |
| 2 | 7 | 0 | -3.421871 | -0.540599 | 0.001978 |
| 3 | 6 | 0 | -2.294641 | 0.218312 | 0.002601 |
| 4 | 16 | 0 | -2.430294 | 1.921325 | 0.009982 |
| 5 | 7 | 0 | -1.136148 | -0.447113 | -0.002233 |
| 6 | 6 | 0 | 0.192748 | 0.162200 | -0.004344 |
| 7 | 6 | 0 | 1.252454 | -0.934893 | -0.001714 |
| 8 | 6 | 0 | 2.678374 | -0.439038 | -0.004114 |
| 9 | 7 | 0 | 2.890538 | 0.823432 | -0.031522 |
| 10 | 6 | 0 | 3.770394 | -1.479361 | 0.016456 |
| 11 | 7 | 0 | 4.208264 | 1.267639 | -0.093312 |
| 12 | 1 | 0 | -3.816094 | -2.305870 | -0.834043 |
| 13 | 1 | 0 | -3.814305 | -2.315128 | 0.818783 |
| 14 | 1 | 0 | -4.312250 | -0.062244 | 0.003925 |
| 15 | 1 | 0 | -1.223806 | -1.458507 | -0.007416 |
| 16 | 1 | 0 | 4.245081 | 2.221631 | 0.242132 |
| 17 | 1 | 0 | 4.875337 | 0.696534 | 0.422180 |
| 18 | 1 | 0 | 0.304024 | 0.804036 | 0.871924 |

| | | | | | |
|---|---|---|---|---|---|
| 19 | 1 | 0 | 0.303630 | 0.799914 | -0.883526 |
| 20 | 1 | 0 | 1.115231 | -1.581455 | 0.875030 |
| 21 | 1 | 0 | 1.114607 | -1.586671 | -0.874862 |
| 22 | 1 | 0 | 4.360028 | -1.421280 | 0.939371 |
| 23 | 1 | 0 | 3.357157 | -2.485879 | -0.048436 |
| 24 | 1 | 0 | 4.461942 | -1.333192 | -0.820042 |

**Data 3:** Cartesian coordinates and energies of the optimized geometry for the conformer **C** of *(E)*-**5** in DMSO solution.

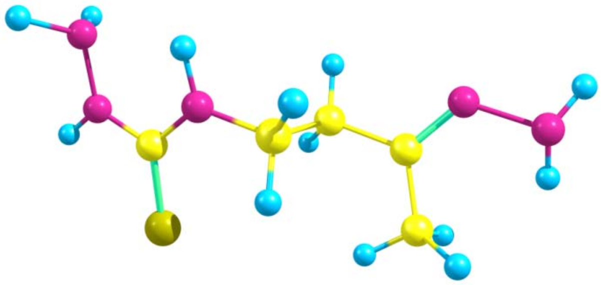

| | |
|---|---|
| Electronic Energy = | -870.449696257 a.u. |
| Zero-point correction= | 0.201773 (Hartree/Particle) |
| Thermal correction to Energy= | 0.215190 |
| Thermal correction to Enthalpy= | 0.216134 |
| Thermal correction to Gibbs Free Energy= | 0.160289 |
| Sum of electronic and zero-point Energies= | -870.247923 |
| Sum of electronic and thermal Energies= | -870.234506 |
| Sum of electronic and thermal Enthalpies= | -870.233562 |
| Sum of electronic and thermal Free Energies= | -870.289407 |

Standard orientation:

| Center Number | Atomic Number | Atomic Type | Coordinates (Angstroms) X | Y | Z |
|---|---|---|---|---|---|
| 1 | 7 | 0 | 3.578445 | -1.804161 | -0.104101 |
| 2 | 7 | 0 | 3.398885 | -0.415070 | -0.206627 |
| 3 | 6 | 0 | 2.201009 | 0.161832 | 0.069930 |
| 4 | 16 | 0 | 2.050797 | 1.861040 | -0.045198 |
| 5 | 7 | 0 | 1.217977 | -0.673830 | 0.425661 |
| 6 | 6 | 0 | -0.163136 | -0.310540 | 0.712202 |
| 7 | 6 | 0 | -1.076874 | -0.441735 | -0.523809 |
| 8 | 6 | 0 | -2.501306 | -0.041340 | -0.229120 |
| 9 | 7 | 0 | -3.376353 | -0.978221 | -0.174517 |
| 10 | 6 | 0 | -2.817355 | 1.416242 | -0.000104 |
| 11 | 7 | 0 | -4.681170 | -0.641906 | 0.164637 |
| 12 | 1 | 0 | 4.282014 | -2.010377 | 0.599555 |
| 13 | 1 | 0 | 3.888490 | -2.176045 | -0.997152 |
| 14 | 1 | 0 | 4.169424 | 0.193321 | -0.447236 |
| 15 | 1 | 0 | 1.474129 | -1.656141 | 0.423951 |
| 16 | 1 | 0 | -5.305641 | -1.360402 | -0.178212 |
| 17 | 1 | 0 | -4.989624 | 0.272331 | -0.160862 |
| 18 | 1 | 0 | -0.170709 | 0.709825 | 1.094396 |
| 19 | 1 | 0 | -0.518484 | -0.973141 | 1.504564 |
| 20 | 1 | 0 | -0.673628 | 0.193039 | -1.320503 |
| 21 | 1 | 0 | -1.063860 | -1.475591 | -0.875469 |
| 22 | 1 | 0 | -3.282253 | 1.569566 | 0.979938 |
| 23 | 1 | 0 | -1.919668 | 2.031363 | -0.056641 |
| 24 | 1 | 0 | -3.521453 | 1.791182 | -0.753142 |

**Data 4:** Cartesian coordinates and energies of the optimized geometry for the conformer **D** of *(E)*-**5** in DMSO solution.

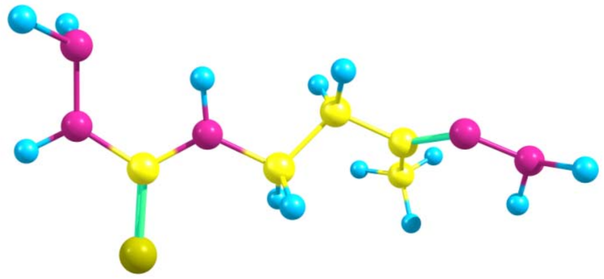

| | |
|---|---|
| Electronic Energy = | -870.448971210 a.u. |
| Zero-point correction= | 0.201436 (Hartree/Particle) |
| Thermal correction to Energy= | 0.215047 |
| Thermal correction to Enthalpy= | 0.215991 |
| Thermal correction to Gibbs Free Energy= | 0.159060 |
| Sum of electronic and zero-point Energies= | -870.247535 |
| Sum of electronic and thermal Energies= | -870.233924 |
| Sum of electronic and thermal Enthalpies= | -870.232980 |
| Sum of electronic and thermal Free Energies= | -870.289912 |

Standard orientation:

| Center Number | Atomic Number | Atomic Type | Coordinates (Angstroms) X | Y | Z |
|---|---|---|---|---|---|
| 1 | 7 | 0 | -3.327060 | -1.981145 | 0.175022 |
| 2 | 7 | 0 | -3.424397 | -0.590166 | 0.010367 |
| 3 | 6 | 0 | -2.313613 | 0.185813 | -0.082086 |
| 4 | 16 | 0 | -2.484344 | 1.874172 | -0.277995 |
| 5 | 7 | 0 | -1.141628 | -0.454090 | -0.008506 |
| 6 | 6 | 0 | 0.174457 | 0.162251 | -0.139841 |
| 7 | 6 | 0 | 1.261203 | -0.824347 | 0.309566 |
| 8 | 6 | 0 | 2.643411 | -0.222002 | 0.245518 |
| 9 | 7 | 0 | 3.443083 | -0.683433 | -0.644918 |
| 10 | 6 | 0 | 3.014460 | 0.864564 | 1.223852 |
| 11 | 7 | 0 | 4.738330 | -0.183449 | -0.685632 |
| 12 | 1 | 0 | -3.802585 | -2.451175 | -0.589848 |
| 13 | 1 | 0 | -3.762444 | -2.256311 | 1.050943 |
| 14 | 1 | 0 | -4.324042 | -0.130577 | -0.023156 |
| 15 | 1 | 0 | -1.208933 | -1.464162 | 0.070335 |
| 16 | 1 | 0 | 5.132628 | -0.366193 | -1.599195 |
| 17 | 1 | 0 | 4.826317 | 0.805286 | -0.459548 |
| 18 | 1 | 0 | 0.195785 | 1.070525 | 0.464827 |
| 19 | 1 | 0 | 0.348324 | 0.458530 | -1.179860 |
| 20 | 1 | 0 | 1.050021 | -1.137589 | 1.339272 |
| 21 | 1 | 0 | 1.239569 | -1.713770 | -0.324978 |
| 22 | 1 | 0 | 3.190732 | 1.821693 | 0.718077 |
| 23 | 1 | 0 | 2.228288 | 1.020689 | 1.962270 |
| 24 | 1 | 0 | 3.938032 | 0.604603 | 1.752398 |

**Data 5:** Cartesian coordinates and energies of the optimized geometry for the conformer **E** of *(E)*-**5** in DMSO solution.

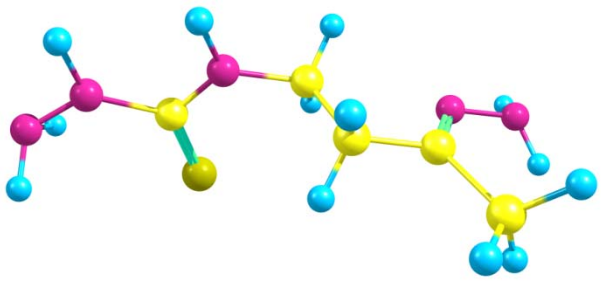

| | |
|---|---|
| Electronic Energy = | -870.443101712 a.u. |
| Zero-point correction= | 0.200946 (Hartree/Particle) |
| Thermal correction to Energy= | 0.214845 |
| Thermal correction to Enthalpy= | 0.215789 |
| Thermal correction to Gibbs Free Energy= | 0.158754 |
| Sum of electronic and zero-point Energies= | -870.242155 |
| Sum of electronic and thermal Energies= | -870.228257 |
| Sum of electronic and thermal Enthalpies= | -870.227313 |
| Sum of electronic and thermal Free Energies= | -870.284348 |

Standard orientation:

| Center Number | Atomic Number | Atomic Type | Coordinates (Angstroms) X | Y | Z |
|---|---|---|---|---|---|
| 1 | 7 | 0 | 4.506596 | -0.303948 | 0.243424 |
| 2 | 7 | 0 | 3.284563 | -0.783063 | -0.246145 |
| 3 | 6 | 0 | 2.102182 | -0.108507 | -0.213987 |
| 4 | 16 | 0 | 1.977059 | 1.428980 | 0.506283 |
| 5 | 7 | 0 | 1.069128 | -0.750879 | -0.795723 |
| 6 | 6 | 0 | -0.310763 | -0.272968 | -0.873607 |
| 7 | 6 | 0 | -1.155515 | -0.749988 | 0.310303 |
| 8 | 6 | 0 | -2.598495 | -0.307766 | 0.282589 |
| 9 | 7 | 0 | -3.003948 | 0.404363 | -0.702139 |
| 10 | 6 | 0 | -3.480679 | -0.746178 | 1.425274 |
| 11 | 7 | 0 | -4.350835 | 0.757824 | -0.746861 |
| 12 | 1 | 0 | 4.424902 | -0.162065 | 1.247656 |
| 13 | 1 | 0 | 4.691266 | 0.609249 | -0.166525 |
| 14 | 1 | 0 | 3.354880 | -1.660312 | -0.741982 |
| 15 | 1 | 0 | 1.236985 | -1.680847 | -1.157320 |
| 16 | 1 | 0 | -4.451378 | 1.574798 | -1.335616 |
| 17 | 1 | 0 | -4.772925 | 0.937943 | 0.162289 |
| 18 | 1 | 0 | -0.728222 | -0.639806 | -1.812057 |
| 19 | 1 | 0 | -0.292957 | 0.813747 | -0.920282 |
| 20 | 1 | 0 | -1.129024 | -1.846375 | 0.362334 |
| 21 | 1 | 0 | -0.699424 | -0.398340 | 1.243674 |
| 22 | 1 | 0 | -4.360349 | -1.282461 | 1.053443 |
| 23 | 1 | 0 | -2.941053 | -1.399643 | 2.110624 |
| 24 | 1 | 0 | -3.844548 | 0.112796 | 2.001912 |

**Data 6:** Cartesian coordinates and energies of the optimized geometry for the conformer **F** of *(E)*-**5** in DMSO solution.

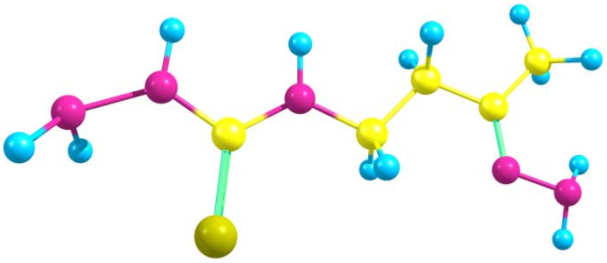

Electronic Energy = -870.442496831 a.u.

| | |
|---|---|
| Zero-point correction= | 0.200594 (Hartree/Particle) |
| Thermal correction to Energy= | 0.214761 |
| Thermal correction to Enthalpy= | 0.215705 |
| Thermal correction to Gibbs Free Energy= | 0.157419 |
| Sum of electronic and zero-point Energies= | -870.241903 |
| Sum of electronic and thermal Energies= | -870.227736 |
| Sum of electronic and thermal Enthalpies= | -870.226792 |
| Sum of electronic and thermal Free Energies= | -870.285077 |

Standard orientation:

| Center Number | Atomic Number | Atomic Type | Coordinates (Angstroms) X | Y | Z |
|---|---|---|---|---|---|
| 1 | 7 | 0 | -4.593519 | -0.626148 | 0.006303 |
| 2 | 7 | 0 | -3.242498 | -0.992609 | -0.028259 |
| 3 | 6 | 0 | -2.189432 | -0.129994 | -0.006670 |
| 4 | 16 | 0 | -2.413089 | 1.555692 | 0.035114 |
| 5 | 7 | 0 | -0.975484 | -0.713054 | -0.021129 |
| 6 | 6 | 0 | 0.303086 | 0.000489 | -0.028778 |
| 7 | 6 | 0 | 1.445947 | -1.009256 | 0.007161 |
| 8 | 6 | 0 | 2.827544 | -0.400137 | 0.003152 |
| 9 | 7 | 0 | 2.937403 | 0.874592 | -0.047375 |
| 10 | 6 | 0 | 3.999932 | -1.347823 | 0.049049 |
| 11 | 7 | 0 | 4.215002 | 1.422234 | -0.111393 |
| 12 | 1 | 0 | -4.795850 | -0.037972 | -0.799071 |
| 13 | 1 | 0 | -4.760469 | -0.057140 | 0.833532 |
| 14 | 1 | 0 | -3.082375 | -1.989721 | -0.031259 |
| 15 | 1 | 0 | -0.934925 | -1.723184 | -0.064691 |
| 16 | 1 | 0 | 4.173871 | 2.381513 | 0.208065 |
| 17 | 1 | 0 | 4.923380 | 0.914342 | 0.415015 |
| 18 | 1 | 0 | 0.352723 | 0.666286 | 0.834351 |
| 19 | 1 | 0 | 0.371650 | 0.625617 | -0.921468 |
| 20 | 1 | 0 | 1.352536 | -1.644344 | 0.897917 |
| 21 | 1 | 0 | 1.369637 | -1.689535 | -0.851975 |
| 22 | 1 | 0 | 4.586459 | -1.213849 | 0.966011 |
| 23 | 1 | 0 | 3.669504 | -2.385927 | 0.016909 |
| 24 | 1 | 0 | 4.674491 | -1.171927 | -0.795672 |

**Data 7:** Cartesian coordinates and energies of the optimized geometry for the conformer **G** of *(E)*-**5** in DMSO solution.

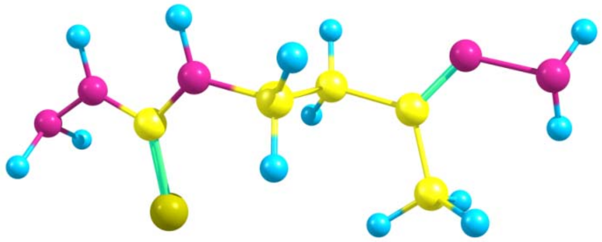

| | |
|---|---|
| Electronic Energy = | -870.443180842 a.u. |
| Zero-point correction= | 0.201120 (Hartree/Particle) |
| Thermal correction to Energy= | 0.214959 |
| Thermal correction to Enthalpy= | 0.215904 |
| Thermal correction to Gibbs Free Energy= | 0.158893 |
| Sum of electronic and zero-point Energies= | -870.242061 |
| Sum of electronic and thermal Energies= | -870.228221 |
| Sum of electronic and thermal Enthalpies= | -870.227277 |
| Sum of electronic and thermal Free Energies= | -870.284288 |

Standard orientation:

| Center Number | Atomic Number | Atomic Type | Coordinates (Angstroms) X | Y | Z |
|---|---|---|---|---|---|
| 1 | 7 | 0 | 4.490371 | -0.372056 | -0.438150 |
| 2 | 7 | 0 | 3.247684 | -0.936862 | -0.123008 |
| 3 | 6 | 0 | 2.111566 | -0.240058 | 0.151711 |
| 4 | 16 | 0 | 2.067900 | 1.458705 | 0.063640 |
| 5 | 7 | 0 | 1.051998 | -1.000425 | 0.500807 |
| 6 | 6 | 0 | -0.302148 | -0.518866 | 0.754872 |
| 7 | 6 | 0 | -1.179187 | -0.510531 | -0.513984 |
| 8 | 6 | 0 | -2.565015 | 0.022472 | -0.246885 |
| 9 | 7 | 0 | -3.532521 | -0.820318 | -0.257089 |
| 10 | 6 | 0 | -2.738240 | 1.494901 | 0.033910 |
| 11 | 7 | 0 | -4.806214 | -0.363922 | 0.058358 |
| 12 | 1 | 0 | 4.744882 | 0.293303 | 0.288928 |
| 13 | 1 | 0 | 4.399495 | 0.158915 | -1.301332 |
| 14 | 1 | 0 | 3.267856 | -1.940620 | -0.011429 |
| 15 | 1 | 0 | 1.166999 | -2.005727 | 0.484669 |
| 16 | 1 | 0 | -5.490100 | -0.996881 | -0.335818 |
| 17 | 1 | 0 | -5.006492 | 0.591486 | -0.231481 |
| 18 | 1 | 0 | -0.227419 | 0.480049 | 1.182846 |
| 19 | 1 | 0 | -0.744574 | -1.177382 | 1.505386 |
| 20 | 1 | 0 | -0.686216 | 0.109704 | -1.270404 |
| 21 | 1 | 0 | -1.257980 | -1.526604 | -0.906756 |
| 22 | 1 | 0 | -3.203666 | 1.657518 | 1.012278 |
| 23 | 1 | 0 | -1.782612 | 2.018165 | 0.014678 |
| 24 | 1 | 0 | -3.387663 | 1.966368 | -0.713980 |

**Data 8:** Cartesian coordinates and energies of the optimized geometry for the conformer **H** of *(E)*-**5** in DMSO solution.

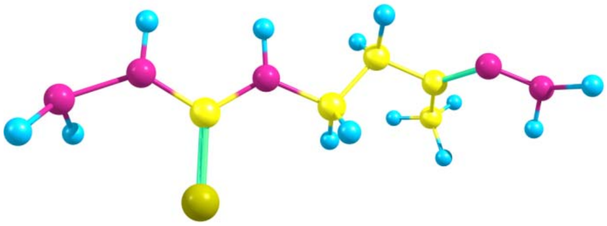

| | |
|---|---|
| Electronic Energy = | -870.442550713 a.u. |
| Zero-point correction= | 0.200868 (Hartree/Particle) |
| Thermal correction to Energy= | 0.214911 |
| Thermal correction to Enthalpy= | 0.215855 |
| Thermal correction to Gibbs Free Energy= | 0.158234 |
| Sum of electronic and zero-point Energies= | -870.241683 |
| Sum of electronic and thermal Energies= | -870.227640 |
| Sum of electronic and thermal Enthalpies= | -870.226695 |
| Sum of electronic and thermal Free Energies= | -870.284316 |

Standard orientation:

| Center Number | Atomic Number | Atomic Type | Coordinates (Angstroms) X | Y | Z |
|---|---|---|---|---|---|
| 1 | 7 | 0 | -4.604000 | -0.648549 | 0.023437 |
| 2 | 7 | 0 | -3.249318 | -0.997952 | 0.085301 |
| 3 | 6 | 0 | -2.205777 | -0.131895 | -0.027735 |
| 4 | 16 | 0 | -2.443531 | 1.527942 | -0.309644 |
| 5 | 7 | 0 | -0.985732 | -0.690231 | 0.100489 |
| 6 | 6 | 0 | 0.284627 | 0.022404 | -0.024655 |
| 7 | 6 | 0 | 1.446966 | -0.929877 | 0.288889 |
| 8 | 6 | 0 | 2.784806 | -0.233478 | 0.233978 |
| 9 | 7 | 0 | 3.563855 | -0.540044 | -0.737702 |

| | | | | | |
|---|---|---|---|---|---|
| 10 | 6 | 0 | 3.138474 | 0.760951 | 1.311559 |
| 11 | 7 | 0 | 4.821058 | 0.048161 | -0.780613 |
| 12 | 1 | 0 | -4.795239 | -0.233170 | -0.885604 |
| 13 | 1 | 0 | -4.792210 | 0.070200 | 0.719187 |
| 14 | 1 | 0 | -3.080561 | -1.972470 | 0.290207 |
| 15 | 1 | 0 | -0.931099 | -1.693255 | 0.223177 |
| 16 | 1 | 0 | 5.178577 | -0.008646 | -1.725293 |
| 17 | 1 | 0 | 4.858067 | 1.010833 | -0.451396 |
| 18 | 1 | 0 | 0.282428 | 0.873837 | 0.659466 |
| 19 | 1 | 0 | 0.390768 | 0.418672 | -1.039133 |
| 20 | 1 | 0 | 1.300631 | -1.352793 | 1.290473 |
| 21 | 1 | 0 | 1.453419 | -1.753795 | -0.428927 |
| 22 | 1 | 0 | 3.237862 | 1.776448 | 0.909562 |
| 23 | 1 | 0 | 2.379655 | 0.787585 | 2.093321 |
| 24 | 1 | 0 | 4.098580 | 0.501159 | 1.770656 |

*Computational data for various conformers of hydrazone (Z)-**5***

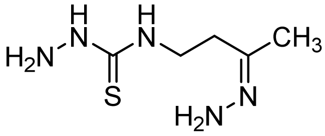


**Data 9:** Cartesian coordinates and energies of the optimized geometry for the conformer **A** of *(Z)*-**5** in DMSO solution.

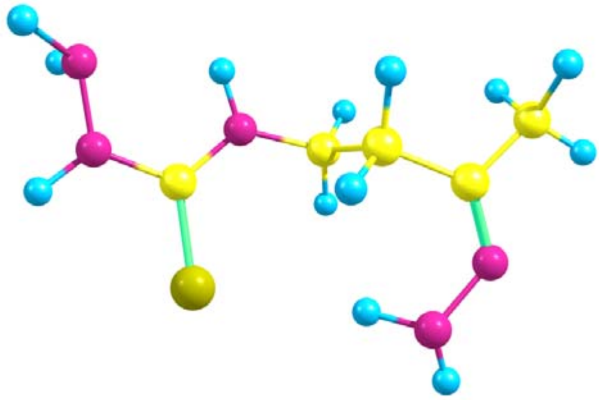

| | |
|---|---|
| Electronic Energy = | -870.449164086 a.u. |
| Zero-point correction= | 0.202104 (Hartree/Particle) |
| Thermal correction to Energy= | 0.215299 |
| Thermal correction to Enthalpy= | 0.216243 |
| Thermal correction to Gibbs Free Energy= | 0.161485 |
| Sum of electronic and zero-point Energies= | -870.247060 |
| Sum of electronic and thermal Energies= | -870.233865 |
| Sum of electronic and thermal Enthalpies= | -870.232921 |
| Sum of electronic and thermal Free Energies= | -870.287679 |

Standard orientation:

| Center Number | Atomic Number | Atomic Type | Coordinates (Angstroms) X | Y | Z |
|---|---|---|---|---|---|
| 1 | 7 | 0 | -3.188760 | -0.121938 | -0.235277 |
| 2 | 6 | 0 | -1.907926 | 0.172231 | 0.099036 |
| 3 | 16 | 0 | -1.414550 | 1.809085 | 0.163994 |
| 4 | 7 | 0 | -1.112723 | -0.871128 | 0.365350 |
| 5 | 6 | 0 | 0.299865 | -0.786849 | 0.706969 |
| 6 | 6 | 0 | 1.218369 | -0.744577 | -0.536601 |
| 7 | 6 | 0 | 2.656627 | -0.454992 | -0.164727 |
| 8 | 6 | 0 | 3.568052 | -1.626977 | 0.080215 |

| | | | | | |
|---|---|---|---|---|---|
| 9 | 7 | 0 | -3.639636 | -1.451099 | -0.280230 |
| 10 | 7 | 0 | 3.146819 | 0.724143 | -0.038702 |
| 11 | 7 | 0 | 2.349393 | 1.827478 | -0.323125 |
| 12 | 1 | 0 | -3.816846 | 0.648134 | -0.421026 |
| 13 | 1 | 0 | -1.548030 | -1.782236 | 0.264987 |
| 14 | 1 | 0 | 1.358755 | 1.742620 | -0.091364 |
| 15 | 1 | 0 | 2.745150 | 2.639576 | 0.131725 |
| 16 | 1 | 0 | -3.993471 | -1.660880 | -1.209116 |
| 17 | 1 | 0 | -4.388303 | -1.583706 | 0.393958 |
| 18 | 1 | 0 | 0.447753 | 0.100532 | 1.322576 |
| 19 | 1 | 0 | 0.537258 | -1.658454 | 1.318914 |
| 20 | 1 | 0 | 0.846381 | 0.018015 | -1.226352 |
| 21 | 1 | 0 | 1.160344 | -1.706287 | -1.052752 |
| 22 | 1 | 0 | 3.664285 | -2.236300 | -0.825637 |
| 23 | 1 | 0 | 4.559000 | -1.289047 | 0.384628 |
| 24 | 1 | 0 | 3.164957 | -2.282538 | 0.860081 |

---------------------------------------------------------------------

**Data 10:** Cartesian coordinates and energies of the optimized geometry for the conformer **B** of *(Z)*-**5** in DMSO solution.

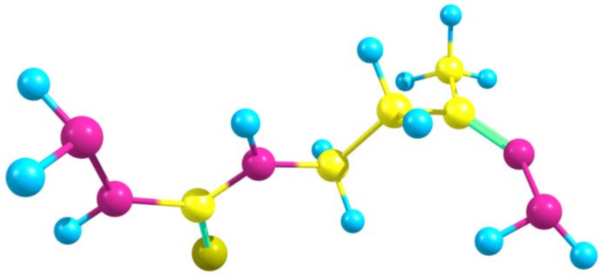

| | |
|---|---|
| Electronic Energy = | -870.446740990 a.u. |
| Zero-point correction= | 0.201421 (Hartree/Particle) |
| Thermal correction to Energy= | 0.214149 |
| Thermal correction to Enthalpy= | 0.215093 |
| Thermal correction to Gibbs Free Energy= | 0.161499 |
| Sum of electronic and zero-point Energies= | -870.245320 |
| Sum of electronic and thermal Energies= | -870.232592 |
| Sum of electronic and thermal Enthalpies= | -870.231648 |
| Sum of electronic and thermal Free Energies= | -870.285242 |

Standard orientation:

| Center Number | Atomic Number | Atomic Type | Coordinates (Angstroms) X | Y | Z |
|---|---|---|---|---|---|
| 1 | 7 | 0 | 3.243846 | -0.616089 | 0.076943 |
| 2 | 6 | 0 | 2.156921 | 0.193853 | -0.000287 |
| 3 | 16 | 0 | 2.363179 | 1.888161 | 0.030580 |
| 4 | 7 | 0 | 0.971815 | -0.419306 | -0.096017 |
| 5 | 6 | 0 | -0.320380 | 0.250840 | -0.195163 |
| 6 | 6 | 0 | -1.445020 | -0.796237 | -0.249106 |
| 7 | 6 | 0 | -2.815317 | -0.162457 | -0.384388 |
| 8 | 6 | 0 | -3.324449 | 0.093934 | -1.776881 |
| 9 | 7 | 0 | 3.110495 | -2.013383 | 0.047334 |
| 10 | 7 | 0 | -3.575492 | 0.155692 | 0.597530 |
| 11 | 7 | 0 | -3.165899 | -0.122946 | 1.898142 |
| 12 | 1 | 0 | 4.153067 | -0.179470 | 0.144080 |
| 13 | 1 | 0 | 1.009804 | -1.433711 | -0.107945 |
| 14 | 1 | 0 | -2.171957 | -0.001568 | 2.075924 |
| 15 | 1 | 0 | -3.695021 | 0.460051 | 2.533518 |
| 16 | 1 | 0 | 3.492273 | -2.413629 | 0.899574 |
| 17 | 1 | 0 | 3.616919 | -2.390796 | -0.748552 |
| 18 | 1 | 0 | -0.447898 | 0.920009 | 0.659900 |
| 19 | 1 | 0 | -0.341729 | 0.874548 | -1.092744 |
| 20 | 1 | 0 | -1.407211 | -1.428328 | 0.643735 |
| 21 | 1 | 0 | -1.278387 | -1.448514 | -1.111448 |

| | | | | | |
|---|---|---|---|---|---|
| 22 | 1 | 0 | -3.422150 | -0.847115 | -2.329691 |
| 23 | 1 | 0 | -4.295392 | 0.588611 | -1.746568 |
| 24 | 1 | 0 | -2.627071 | 0.721971 | -2.341645 |

**Data 11:** Cartesian coordinates and energies of the optimized geometry for the conformer **C** of *(Z)*-**5** in DMSO solution.

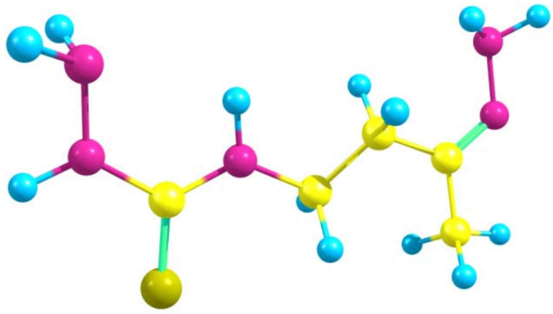

| | |
|---|---|
| Electronic Energy = | -870.446073082 a.u. |
| Zero-point correction= | 0.201383 (Hartree/Particle) |
| Thermal correction to Energy= | 0.214985 |
| Thermal correction to Enthalpy= | 0.215929 |
| Thermal correction to Gibbs Free Energy= | 0.159216 |
| Sum of electronic and zero-point Energies= | -870.244690 |
| Sum of electronic and thermal Energies= | -870.231088 |
| Sum of electronic and thermal Enthalpies= | -870.230144 |
| Sum of electronic and thermal Free Energies= | -870.286857 |

Standard orientation:

| Center Number | Atomic Number | Atomic Type | Coordinates (Angstroms) X | Y | Z |
|---|---|---|---|---|---|
| 1 | 7 | 0 | -3.205477 | 0.896793 | 0.028296 |
| 2 | 6 | 0 | -2.247578 | -0.064434 | -0.019514 |
| 3 | 16 | 0 | -2.704212 | -1.704910 | -0.142082 |
| 4 | 7 | 0 | -0.981606 | 0.363803 | 0.035335 |
| 5 | 6 | 0 | 0.200700 | -0.491448 | -0.003396 |
| 6 | 6 | 0 | 1.455339 | 0.378130 | 0.086179 |
| 7 | 6 | 0 | 2.771018 | -0.375272 | 0.056585 |
| 8 | 6 | 0 | 2.802196 | -1.870210 | 0.228912 |
| 9 | 7 | 0 | -2.865038 | 2.255461 | 0.126164 |
| 10 | 7 | 0 | 3.897674 | 0.219153 | -0.097756 |
| 11 | 7 | 0 | 3.899394 | 1.599710 | -0.288775 |
| 12 | 1 | 0 | -4.171736 | 0.602677 | -0.012381 |
| 13 | 1 | 0 | -0.870159 | 1.370528 | 0.103906 |
| 14 | 1 | 0 | 3.222434 | 2.110425 | 0.274541 |
| 15 | 1 | 0 | 4.829093 | 1.947850 | -0.092839 |
| 16 | 1 | 0 | -3.264537 | 2.650038 | 0.972925 |
| 17 | 1 | 0 | -3.231085 | 2.760526 | -0.675880 |
| 18 | 1 | 0 | 0.156038 | -1.203836 | 0.823287 |
| 19 | 1 | 0 | 0.197358 | -1.069184 | -0.931239 |
| 20 | 1 | 0 | 1.409508 | 0.974071 | 1.009526 |
| 21 | 1 | 0 | 1.455466 | 1.098810 | -0.741392 |
| 22 | 1 | 0 | 2.204489 | -2.376995 | -0.535304 |
| 23 | 1 | 0 | 3.830011 | -2.226214 | 0.160521 |
| 24 | 1 | 0 | 2.395849 | -2.165181 | 1.202192 |

**Data 12:** Cartesian coordinates and energies of the optimized geometry for the conformer **D** of *(Z)*-**5** in DMSO solution.

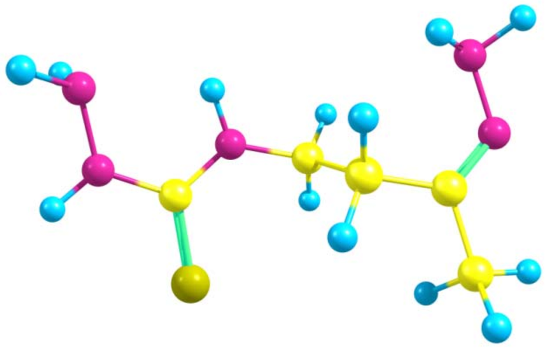

| | |
|---|---|
| Electronic Energy = | -870.448101989 a.u. |
| Zero-point correction= | 0.201989 (Hartree/Particle) |
| Thermal correction to Energy= | 0.215308 |
| Thermal correction to Enthalpy= | 0.216252 |
| Thermal correction to Gibbs Free Energy= | 0.160651 |
| Sum of electronic and zero-point Energies= | -870.246113 |
| Sum of electronic and thermal Energies= | -870.232794 |
| Sum of electronic and thermal Enthalpies= | -870.231850 |
| Sum of electronic and thermal Free Energies= | -870.287451 |

Standard orientation:

| Center Number | Atomic Number | Atomic Type | Coordinates (Angstroms) X | Y | Z |
|---|---|---|---|---|---|
| 1 | 7 | 0 | -3.216555 | -0.461877 | -0.238879 |
| 2 | 6 | 0 | -2.052981 | 0.152268 | 0.096133 |
| 3 | 16 | 0 | -2.011822 | 1.857488 | 0.201850 |
| 4 | 7 | 0 | -1.011008 | -0.656464 | 0.327411 |
| 5 | 6 | 0 | 0.346132 | -0.240465 | 0.650015 |
| 6 | 6 | 0 | 1.231481 | -0.077264 | -0.605491 |
| 7 | 6 | 0 | 2.645415 | 0.330316 | -0.245607 |
| 8 | 6 | 0 | 2.982920 | 1.794499 | -0.322355 |
| 9 | 7 | 0 | -3.306607 | -1.861675 | -0.309744 |
| 10 | 7 | 0 | 3.565950 | -0.474757 | 0.141842 |
| 11 | 7 | 0 | 3.272828 | -1.826348 | 0.291587 |
| 12 | 1 | 0 | -4.030100 | 0.120162 | -0.384781 |
| 13 | 1 | 0 | -1.204992 | -1.645433 | 0.205975 |
| 14 | 1 | 0 | 2.582602 | -2.192533 | -0.362437 |
| 15 | 1 | 0 | 4.132465 | -2.354228 | 0.210372 |
| 16 | 1 | 0 | -3.613662 | -2.137925 | -1.237820 |
| 17 | 1 | 0 | -3.979800 | -2.199133 | 0.372666 |
| 18 | 1 | 0 | 0.293487 | 0.701936 | 1.194200 |
| 19 | 1 | 0 | 0.769945 | -0.994740 | 1.314481 |
| 20 | 1 | 0 | 0.788472 | 0.686201 | -1.248401 |
| 21 | 1 | 0 | 1.221096 | -1.012632 | -1.176123 |
| 22 | 1 | 0 | 2.294346 | 2.385845 | 0.291512 |
| 23 | 1 | 0 | 4.002633 | 1.974111 | 0.019180 |
| 24 | 1 | 0 | 2.882617 | 2.160294 | -1.350113 |

**Table S12.** Relative electronic (ΔE, kcal/mol) and Gibbs free energies (ΔG, kcal/mol) of various conformers of (*E*)- and *(Z)*-**5** in DMSO solution.

| Conformer | (*E*)-**5** | | *(Z)*-**5** | |
|---|---|---|---|---|
| | ΔE | ΔG | ΔE | ΔG |
| **A** | 0.06 | 0.18 | 0.33 | 1.68 |
| **B** | 0.48 | 0.00 | 1.85 | 3.21 |
| **C** | 0.00 | 0.60 | 2.27 | 2.20 |
| **D** | 0.45 | 0.28 | 1.00 | 1.82 |
| **E** | 4.14 | 3.77 | - | - |
| **F** | 4.52 | 3.31 | - | - |
| **G** | 4.09 | 3.81 | - | - |
| **H** | 4.48 | 3.79 | - | - |

**Table S13.** Experimental $^{13}C$ chemical shifts of (*E*)- and *(Z)*-**5** (DMSO-$d_6$) and calculated $^{13}C$ chemical shifts for various conformers of (*E*)- and *(Z)*-**5** in DMSO solution.[a]

| $^{13}C$ assign-ment | Calculated shift of (*E*)-**5**, ppm | | | | Calculated shift of *(Z)*-**5**, ppm | | | | Observed shift, ppm | |
|---|---|---|---|---|---|---|---|---|---|---|
| | Con-former **A** | Con-former **B** | Con-former **C** | Con-former **D** | Con-former **A** | Con-former **B** | Con-former **C** | Con-former **D** | Major isomer | Minor isomer |
| C=S | 188.58 | 189.12 | 188.64 | 189.04 | 189.54 | 189.28 | 189.42 | 189.05 | 180.87 | - |
| C=N | 151.25 | 150.05 | 151.07 | 150.88 | 149.71 | 150.63 | 151.65 | 150.58 | 145.39 | 144.40 |
| $NCH_2$ | 38.44 | 40.01 | 40.32 | 41.74 | 38.13 | 37.92 | 39.51 | 34.52 | 40.18 | 38.54 |
| $CH_2C$=N | 37.74 | 36.15 | 41.24 | 39.82 | 30.65 | 27.06 | 27.01 | 28.98 | 37.72 | 28.99 |
| $CH_3$ | 14.75 | 14.80 | 13.08 | 13.57 | 24.81 | 24.77 | 22.08 | 25.32 | 14.21 | 22.88 |

[a] Calculations were performed by the GIAO method at the WC04/6-311+G(2d,p) level of theory using the DFT B3LYP/6-311++G(d,p) optimized geometries.

## EtOH solution

*Computational data for various conformers of hydrazone (E)-**5***

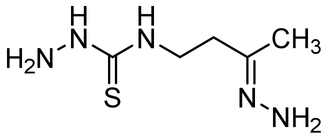


**Data 13:** Cartesian coordinates and energies of the optimized geometry for the conformer **A** of *(E)*-**5** in EtOH solution.

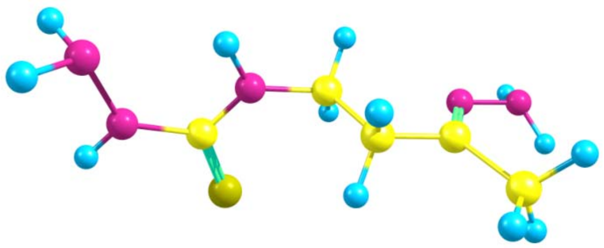

Electronic Energy = -870.448854328 a.u.
Zero-point correction= 0.201428 (Hartree/Particle)
Thermal correction to Energy= 0.214965
Thermal correction to Enthalpy= 0.215909
Thermal correction to Gibbs Free Energy= 0.159524
Sum of electronic and zero-point Energies= -870.247426
Sum of electronic and thermal Energies= -870.233889
Sum of electronic and thermal Enthalpies= -870.232945
Sum of electronic and thermal Free Energies= -870.289331

Standard orientation:

| Center Number | Atomic Number | Atomic Type | Coordinates (Angstroms) X | Y | Z |
|---|---|---|---|---|---|
| 1 | 7 | 0 | 3.671894 | -1.633583 | -0.177563 |
| 2 | 7 | 0 | 3.408190 | -0.287943 | 0.125451 |
| 3 | 6 | 0 | 2.170018 | 0.244631 | -0.049874 |
| 4 | 16 | 0 | 1.923301 | 1.899551 | 0.300762 |
| 5 | 7 | 0 | 1.230313 | -0.594615 | -0.498091 |
| 6 | 6 | 0 | -0.175150 | -0.277031 | -0.731829 |
| 7 | 6 | 0 | -1.041620 | -0.571545 | 0.495409 |
| 8 | 6 | 0 | -2.515781 | -0.300951 | 0.316574 |
| 9 | 7 | 0 | -2.931431 | 0.111848 | -0.822626 |
| 10 | 6 | 0 | -3.414821 | -0.547596 | 1.502900 |
| 11 | 7 | 0 | -4.300994 | 0.300916 | -0.993539 |
| 12 | 1 | 0 | 4.030031 | -2.106711 | 0.646861 |
| 13 | 1 | 0 | 4.365644 | -1.692130 | -0.917814 |
| 14 | 1 | 0 | 4.146214 | 0.331845 | 0.430024 |
| 15 | 1 | 0 | 1.547264 | -1.547824 | -0.643199 |
| 16 | 1 | 0 | -4.445321 | 0.939045 | -1.765578 |
| 17 | 1 | 0 | -4.788499 | 0.646025 | -0.168711 |
| 18 | 1 | 0 | -0.506975 | -0.871483 | -1.583799 |
| 19 | 1 | 0 | -0.249636 | 0.772659 | -1.009661 |
| 20 | 1 | 0 | -0.914792 | -1.621316 | 0.791473 |
| 21 | 1 | 0 | -0.676711 | 0.020803 | 1.343313 |
| 22 | 1 | 0 | -4.223436 | -1.238430 | 1.241300 |
| 23 | 1 | 0 | -2.856516 | -0.968757 | 2.338914 |
| 24 | 1 | 0 | -3.881775 | 0.382057 | 1.850173 |

**Data 14:** Cartesian coordinates and energies of the optimized geometry for the conformer **B** of *(E)*-**5** in EtOH solution.

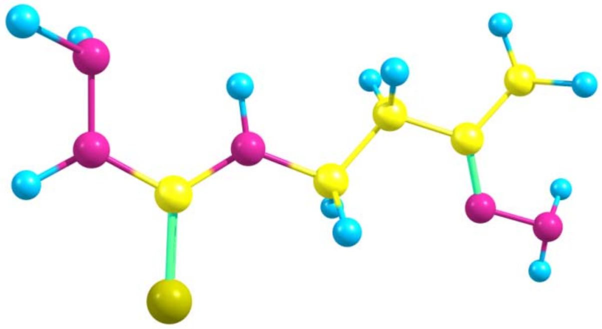

| | |
|---|---|
| Electronic Energy = | -870.448116207 a.u. |
| Zero-point correction= | 0.201152 (Hartree/Particle) |
| Thermal correction to Energy= | 0.214864 |
| Thermal correction to Enthalpy= | 0.215808 |
| Thermal correction to Gibbs Free Energy= | 0.158800 |
| Sum of electronic and zero-point Energies= | -870.246964 |
| Sum of electronic and thermal Energies= | -870.233252 |
| Sum of electronic and thermal Enthalpies= | -870.232308 |
| Sum of electronic and thermal Free Energies= | -870.289316 |

Standard orientation:

| Center Number | Atomic Number | Atomic Type | Coordinates (Angstroms) X | Y | Z |
|---|---|---|---|---|---|
| 1 | 7 | 0 | -3.352246 | -1.943062 | -0.016142 |
| 2 | 7 | 0 | -3.421749 | -0.540955 | 0.001011 |
| 3 | 6 | 0 | -2.294513 | 0.218928 | 0.003510 |
| 4 | 16 | 0 | -2.430878 | 1.920848 | 0.019467 |
| 5 | 7 | 0 | -1.135894 | -0.446704 | -0.006033 |
| 6 | 6 | 0 | 0.192918 | 0.162839 | -0.010590 |
| 7 | 6 | 0 | 1.252377 | -0.934499 | 0.003364 |
| 8 | 6 | 0 | 2.678479 | -0.439111 | -0.001165 |
| 9 | 7 | 0 | 2.890792 | 0.822909 | -0.041199 |
| 10 | 6 | 0 | 3.770494 | -1.479217 | 0.031972 |
| 11 | 7 | 0 | 4.208178 | 1.266527 | -0.105091 |
| 12 | 1 | 0 | -3.816200 | -2.301374 | -0.845969 |
| 13 | 1 | 0 | -3.812216 | -2.321219 | 0.807037 |
| 14 | 1 | 0 | -4.311962 | -0.062354 | 0.005291 |
| 15 | 1 | 0 | -1.223368 | -1.457952 | -0.018620 |
| 16 | 1 | 0 | 4.244213 | 2.224608 | 0.218428 |
| 17 | 1 | 0 | 4.874792 | 0.701475 | 0.417561 |
| 18 | 1 | 0 | 0.301981 | 0.811773 | 0.860567 |
| 19 | 1 | 0 | 0.306034 | 0.793838 | -0.894388 |
| 20 | 1 | 0 | 1.113225 | -1.573297 | 0.885524 |
| 21 | 1 | 0 | 1.115873 | -1.593911 | -0.864371 |
| 22 | 1 | 0 | 4.358710 | -1.411750 | 0.955211 |
| 23 | 1 | 0 | 3.357499 | -2.486450 | -0.023224 |
| 24 | 1 | 0 | 4.463364 | -1.341601 | -0.804895 |

**Data 15:** Cartesian coordinates and energies of the optimized geometry for the conformer **C** of *(E)*-**5** in EtOH solution.

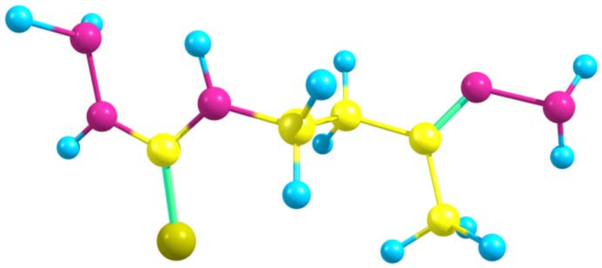

| | |
|---|---|
| Electronic Energy = | -870.449006986 a.u. |
| Zero-point correction= | 0.201787 (Hartree/Particle) |
| Thermal correction to Energy= | 0.215197 |
| Thermal correction to Enthalpy= | 0.216142 |
| Thermal correction to Gibbs Free Energy= | 0.160324 |
| Sum of electronic and zero-point Energies= | -870.247220 |
| Sum of electronic and thermal Energies= | -870.233810 |
| Sum of electronic and thermal Enthalpies= | -870.232865 |

Sum of electronic and thermal Free Energies= -870.288683

Standard orientation:

| Center Number | Atomic Number | Atomic Type | Coordinates (Angstroms) X | Y | Z |
|---|---|---|---|---|---|
| 1 | 7 | 0 | 3.580869 | -1.802159 | -0.103209 |
| 2 | 7 | 0 | 3.398915 | -0.413564 | -0.207357 |
| 3 | 6 | 0 | 2.200150 | 0.162379 | 0.069635 |
| 4 | 16 | 0 | 2.048159 | 1.860566 | -0.045404 |
| 5 | 7 | 0 | 1.218233 | -0.674949 | 0.425373 |
| 6 | 6 | 0 | -0.163139 | -0.312746 | 0.712010 |
| 7 | 6 | 0 | -1.076387 | -0.440969 | -0.524652 |
| 8 | 6 | 0 | -2.500818 | -0.041155 | -0.229297 |
| 9 | 7 | 0 | -3.375651 | -0.978247 | -0.175862 |
| 10 | 6 | 0 | -2.816926 | 1.416071 | 0.002209 |
| 11 | 7 | 0 | -4.680253 | -0.642687 | 0.164108 |
| 12 | 1 | 0 | 4.283358 | -2.007097 | 0.601888 |
| 13 | 1 | 0 | 3.892059 | -2.174935 | -0.995453 |
| 14 | 1 | 0 | 4.168728 | 0.196441 | -0.446114 |
| 15 | 1 | 0 | 1.475486 | -1.656890 | 0.423736 |
| 16 | 1 | 0 | -5.304701 | -1.361212 | -0.178600 |
| 17 | 1 | 0 | -4.989228 | 0.271941 | -0.159737 |
| 18 | 1 | 0 | -0.170985 | 0.706756 | 1.096577 |
| 19 | 1 | 0 | -0.518820 | -0.977278 | 1.502628 |
| 20 | 1 | 0 | -0.672643 | 0.195666 | -1.319583 |
| 21 | 1 | 0 | -1.063698 | -1.474021 | -0.878703 |
| 22 | 1 | 0 | -3.281470 | 1.567689 | 0.982700 |
| 23 | 1 | 0 | -1.919335 | 2.031391 | -0.053719 |
| 24 | 1 | 0 | -3.521365 | 1.792246 | -0.749957 |

**Data 16:** Cartesian coordinates and energies of the optimized geometry for the conformer **D** of *(E)*-**5** in EtOH solution.

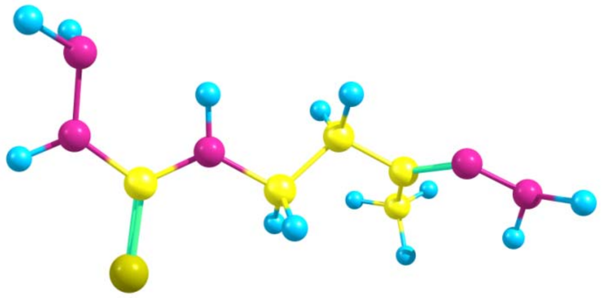

Electronic Energy = -870.448247277 a.u.
Zero-point correction= 0.201450 (Hartree/Particle)
Thermal correction to Energy= 0.215060
Thermal correction to Enthalpy= 0.216004
Thermal correction to Gibbs Free Energy= 0.158967
Sum of electronic and zero-point Energies= -870.246797
Sum of electronic and thermal Energies= -870.233188
Sum of electronic and thermal Enthalpies= -870.232243
Sum of electronic and thermal Free Energies= -870.289280

Standard orientation:

| Center Number | Atomic Number | Atomic Type | Coordinates (Angstroms) X | Y | Z |
|---|---|---|---|---|---|
| 1 | 7 | 0 | -3.327826 | -1.981405 | 0.179255 |
| 2 | 7 | 0 | -3.424320 | -0.590434 | 0.014604 |
| 3 | 6 | 0 | -2.313095 | 0.185104 | -0.082910 |

| | | | | | |
|---|---|---|---|---|---|
| 4 | 16 | 0 | -2.483895 | 1.872587 | -0.279289 |
| 5 | 7 | 0 | -1.141439 | -0.456404 | -0.013012 |
| 6 | 6 | 0 | 0.174753 | 0.157655 | -0.152418 |
| 7 | 6 | 0 | 1.260815 | -0.819154 | 0.319600 |
| 8 | 6 | 0 | 2.642852 | -0.217361 | 0.248022 |
| 9 | 7 | 0 | 3.445536 | -0.695411 | -0.630776 |
| 10 | 6 | 0 | 3.010340 | 0.887977 | 1.206524 |
| 11 | 7 | 0 | 4.740353 | -0.195380 | -0.677189 |
| 12 | 1 | 0 | -3.806186 | -2.451662 | -0.583678 |
| 13 | 1 | 0 | -3.759243 | -2.256363 | 1.057178 |
| 14 | 1 | 0 | -4.323430 | -0.129675 | -0.016423 |
| 15 | 1 | 0 | -1.209637 | -1.466443 | 0.064874 |
| 16 | 1 | 0 | 5.138402 | -0.397240 | -1.585031 |
| 17 | 1 | 0 | 4.827266 | 0.797785 | -0.470925 |
| 18 | 1 | 0 | 0.192679 | 1.077461 | 0.434428 |
| 19 | 1 | 0 | 0.351895 | 0.434007 | -1.197448 |
| 20 | 1 | 0 | 1.046438 | -1.112245 | 1.354587 |
| 21 | 1 | 0 | 1.242091 | -1.720946 | -0.297388 |
| 22 | 1 | 0 | 3.190257 | 1.834822 | 0.682990 |
| 23 | 1 | 0 | 2.220686 | 1.059645 | 1.937752 |
| 24 | 1 | 0 | 3.930988 | 0.637370 | 1.744638 |

*Computational data for various conformers of hydrazone (Z)-**5***

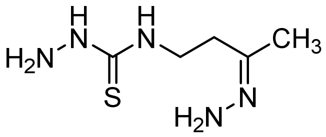


**Data 17:** Cartesian coordinates and energies of the optimized geometry for the conformer **A** of *(Z)*-**5** in EtOH solution.

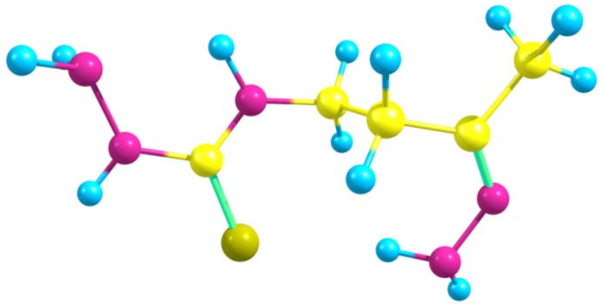

| | |
|---|---|
| Electronic Energy = | -870.448467233 a.u. |
| Zero-point correction= | 0.202128 (Hartree/Particle) |
| Thermal correction to Energy= | 0.215314 |
| Thermal correction to Enthalpy= | 0.216258 |
| Thermal correction to Gibbs Free Energy= | 0.161527 |
| Sum of electronic and zero-point Energies= | -870.246339 |
| Sum of electronic and thermal Energies= | -870.233154 |
| Sum of electronic and thermal Enthalpies= | -870.232209 |
| Sum of electronic and thermal Free Energies= | -870.286940 |

Standard orientation:

| Center Number | Atomic Number | Atomic Type | Coordinates (Angstroms) X | Y | Z |
|---|---|---|---|---|---|
| 1 | 7 | 0 | -3.188746 | -0.121053 | -0.235361 |
| 2 | 6 | 0 | -1.907452 | 0.172529 | 0.099431 |
| 3 | 16 | 0 | -1.413888 | 1.808377 | 0.166037 |
| 4 | 7 | 0 | -1.112962 | -0.871975 | 0.364611 |

| | | | | | |
|---|---|---|---|---|---|
| 5 | 6 | 0 | 0.299643 | -0.788324 | 0.706371 |
| 6 | 6 | 0 | 1.218543 | -0.745336 | -0.536850 |
| 7 | 6 | 0 | 2.656535 | -0.454750 | -0.164528 |
| 8 | 6 | 0 | 3.568664 | -1.625936 | 0.081966 |
| 9 | 7 | 0 | -3.640503 | -1.449797 | -0.281615 |
| 10 | 7 | 0 | 3.146051 | 0.724722 | -0.039423 |
| 11 | 7 | 0 | 2.348837 | 1.827188 | -0.325161 |
| 12 | 1 | 0 | -3.815977 | 0.649874 | -0.420323 |
| 13 | 1 | 0 | -1.548814 | -1.782642 | 0.263294 |
| 14 | 1 | 0 | 1.357811 | 1.743090 | -0.094887 |
| 15 | 1 | 0 | 2.744690 | 2.640421 | 0.127413 |
| 16 | 1 | 0 | -3.994050 | -1.659107 | -1.210696 |
| 17 | 1 | 0 | -4.388853 | -1.583278 | 0.392728 |
| 18 | 1 | 0 | 0.447513 | 0.098817 | 1.322335 |
| 19 | 1 | 0 | 0.536804 | -1.660223 | 1.318111 |
| 20 | 1 | 0 | 0.846234 | 0.017158 | -1.226527 |
| 21 | 1 | 0 | 1.161291 | -1.706942 | -1.053431 |
| 22 | 1 | 0 | 3.665513 | -2.236486 | -0.823036 |
| 23 | 1 | 0 | 4.559316 | -1.286657 | 0.385771 |
| 24 | 1 | 0 | 3.166381 | -2.280740 | 0.862958 |

**Data 18:** Cartesian coordinates and energies of the optimized geometry for the conformer **B** of *(Z)*-**5** in EtOH solution.

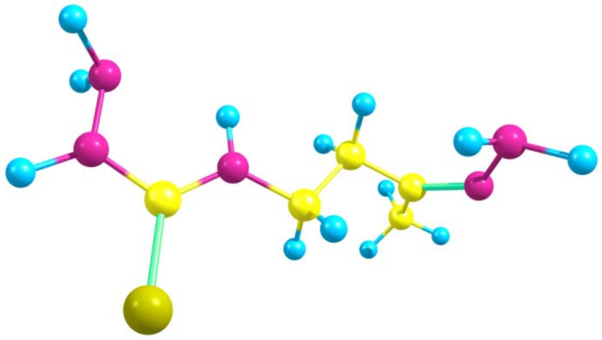

Electronic Energy = -870.445990569 a.u.
Zero-point correction= 0.201397 (Hartree/Particle)
Thermal correction to Energy= 0.214137
Thermal correction to Enthalpy= 0.215081
Thermal correction to Gibbs Free Energy= 0.161419
Sum of electronic and zero-point Energies= -870.244593
Sum of electronic and thermal Energies= -870.231854
Sum of electronic and thermal Enthalpies= -870.230910
Sum of electronic and thermal Free Energies= -870.284571

Standard orientation:

| Center Number | Atomic Number | Atomic Type | Coordinates (Angstroms) X | Y | Z |
|---|---|---|---|---|---|
| 1 | 7 | 0 | 3.243917 | -0.615026 | 0.080766 |
| 2 | 6 | 0 | 2.156568 | 0.194558 | -0.002126 |
| 3 | 16 | 0 | 2.362038 | 1.888174 | 0.019712 |
| 4 | 7 | 0 | 0.971823 | -0.420251 | -0.095836 |
| 5 | 6 | 0 | -0.320356 | 0.248965 | -0.200726 |
| 6 | 6 | 0 | -1.445179 | -0.798387 | -0.243768 |
| 7 | 6 | 0 | -2.815888 | -0.166153 | -0.382210 |
| 8 | 6 | 0 | -3.327957 | 0.077818 | -1.775836 |
| 9 | 7 | 0 | 3.111408 | -2.012488 | 0.059069 |
| 10 | 7 | 0 | -3.574141 | 0.160388 | 0.598345 |
| 11 | 7 | 0 | -3.161129 | -0.105983 | 1.900313 |
| 12 | 1 | 0 | 4.152584 | -0.177167 | 0.147048 |
| 13 | 1 | 0 | 1.010332 | -1.434609 | -0.102715 |
| 14 | 1 | 0 | -2.167310 | 0.020532 | 2.075161 |
| 15 | 1 | 0 | -3.691415 | 0.480123 | 2.531797 |
| 16 | 1 | 0 | 3.491670 | -2.408036 | 0.914164 |

| | | | | | |
|---|---|---|---|---|---|
| 17 | 1 | 0 | 3.618406 | -2.394692 | -0.734144 |
| 18 | 1 | 0 | -0.446981 | 0.926445 | 0.647883 |
| 19 | 1 | 0 | -0.342272 | 0.864441 | -1.104006 |
| 20 | 1 | 0 | -1.406233 | -1.422476 | 0.654691 |
| 21 | 1 | 0 | -1.280029 | -1.458416 | -1.100553 |
| 22 | 1 | 0 | -3.425766 | -0.867909 | -2.320625 |
| 23 | 1 | 0 | -4.299375 | 0.571621 | -1.747509 |
| 24 | 1 | 0 | -2.632498 | 0.702069 | -2.347175 |

**Data 19:** Cartesian coordinates and energies of the optimized geometry for the conformer **C** of *(Z)*-**5** in EtOH solution.

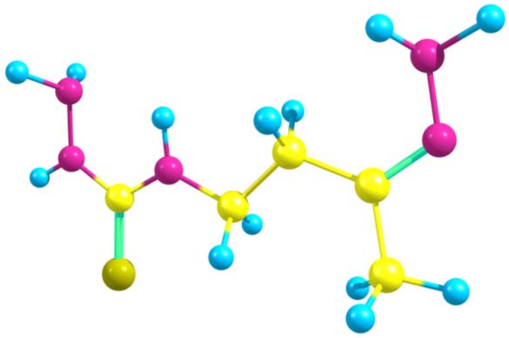

| | |
|---|---|
| Electronic Energy = | -870.445300819 a.u. |
| Zero-point correction= | 0.201382 (Hartree/Particle) |
| Thermal correction to Energy= | 0.214984 |
| Thermal correction to Enthalpy= | 0.215928 |
| Thermal correction to Gibbs Free Energy= | 0.159191 |
| Sum of electronic and zero-point Energies= | -870.243919 |
| Sum of electronic and thermal Energies= | -870.230317 |
| Sum of electronic and thermal Enthalpies= | -870.229373 |
| Sum of electronic and thermal Free Energies= | -870.286109 |

Standard orientation:

| Center Number | Atomic Number | Atomic Type | Coordinates (Angstroms) X | Y | Z |
|---|---|---|---|---|---|
| 1 | 7 | 0 | -3.205843 | 0.896341 | 0.029049 |
| 2 | 6 | 0 | -2.247574 | -0.065178 | -0.019004 |
| 3 | 16 | 0 | -2.703548 | -1.704909 | -0.140983 |
| 4 | 7 | 0 | -0.981623 | 0.364001 | 0.035694 |
| 5 | 6 | 0 | 0.200674 | -0.491115 | -0.004582 |
| 6 | 6 | 0 | 1.455331 | 0.378143 | 0.087782 |
| 7 | 6 | 0 | 2.771078 | -0.375166 | 0.056361 |
| 8 | 6 | 0 | 2.802564 | -1.870298 | 0.226914 |
| 9 | 7 | 0 | -2.866010 | 2.255302 | 0.124206 |
| 10 | 7 | 0 | 3.897510 | 0.219508 | -0.098136 |
| 11 | 7 | 0 | 3.898843 | 1.600210 | -0.287086 |
| 12 | 1 | 0 | -4.171766 | 0.601555 | -0.014109 |
| 13 | 1 | 0 | -0.870414 | 1.370789 | 0.102474 |
| 14 | 1 | 0 | 3.223575 | 2.110296 | 0.278799 |
| 15 | 1 | 0 | 4.829268 | 1.947857 | -0.093916 |
| 16 | 1 | 0 | -3.266037 | 2.651900 | 0.969737 |
| 17 | 1 | 0 | -3.230458 | 2.758870 | -0.679509 |
| 18 | 1 | 0 | 0.155308 | -1.205689 | 0.820144 |
| 19 | 1 | 0 | 0.197743 | -1.066756 | -0.933751 |
| 20 | 1 | 0 | 1.409105 | 0.971641 | 1.012767 |
| 21 | 1 | 0 | 1.455978 | 1.101005 | -0.737915 |
| 22 | 1 | 0 | 2.203955 | -2.376388 | -0.537043 |
| 23 | 1 | 0 | 3.830405 | -2.225834 | 0.156757 |
| 24 | 1 | 0 | 2.397539 | -2.166536 | 1.200386 |

**Data 20:** Cartesian coordinates and energies of the optimized geometry for the conformer **D** of *(Z)*-**5** in EtOH solution.

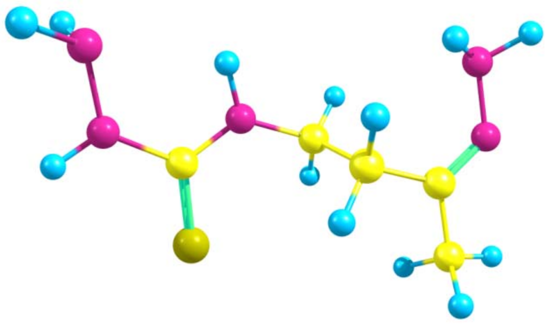

| | |
|---|---|
| Electronic Energy = | -870.447355692 a.u. |
| Zero-point correction= | 0.202015 (Hartree/Particle) |
| Thermal correction to Energy= | 0.215322 |
| Thermal correction to Enthalpy= | 0.216266 |
| Thermal correction to Gibbs Free Energy= | 0.160736 |
| Sum of electronic and zero-point Energies= | -870.245341 |
| Sum of electronic and thermal Energies= | -870.232034 |
| Sum of electronic and thermal Enthalpies= | -870.231089 |
| Sum of electronic and thermal Free Energies= | -870.286620 |

Standard orientation:

| Center Number | Atomic Number | Atomic Type | Coordinates (Angstroms) X | Y | Z |
|---|---|---|---|---|---|
| 1 | 7 | 0 | 3.216888 | -0.460443 | 0.238976 |
| 2 | 6 | 0 | 2.052075 | 0.153253 | -0.094604 |
| 3 | 16 | 0 | 2.007954 | 1.857774 | -0.194755 |
| 4 | 7 | 0 | 1.011492 | -0.657049 | -0.328159 |
| 5 | 6 | 0 | -0.345844 | -0.241791 | -0.651016 |
| 6 | 6 | 0 | -1.230759 | -0.074356 | 0.604250 |
| 7 | 6 | 0 | -2.645122 | 0.330300 | 0.242732 |
| 8 | 6 | 0 | -2.983401 | 1.794721 | 0.310601 |
| 9 | 7 | 0 | 3.310404 | -1.860359 | 0.301812 |
| 10 | 7 | 0 | -3.565229 | -0.477580 | -0.139696 |
| 11 | 7 | 0 | -3.271093 | -1.829840 | -0.280984 |
| 12 | 1 | 0 | 4.029935 | 0.122828 | 0.382610 |
| 13 | 1 | 0 | 1.207395 | -1.646049 | -0.210877 |
| 14 | 1 | 0 | -2.582297 | -2.191847 | 0.376849 |
| 15 | 1 | 0 | -4.130757 | -2.357507 | -0.199030 |
| 16 | 1 | 0 | 3.620584 | -2.141357 | 1.227375 |
| 17 | 1 | 0 | 3.981506 | -2.193301 | -0.384885 |
| 18 | 1 | 0 | -0.293450 | 0.699053 | -1.197987 |
| 19 | 1 | 0 | -0.770191 | -0.997910 | -1.313055 |
| 20 | 1 | 0 | -0.788026 | 0.692222 | 1.243602 |
| 21 | 1 | 0 | -1.219219 | -1.007400 | 1.178763 |
| 22 | 1 | 0 | -2.294678 | 2.382795 | -0.306201 |
| 23 | 1 | 0 | -4.002977 | 1.971496 | -0.032742 |
| 24 | 1 | 0 | -2.884024 | 2.166727 | 1.336234 |

**Table S14.** Relative electronic (ΔE, kcal/mol) and Gibbs free energies (ΔG, kcal/mol) of various conformers of (*E*)- and *(Z)*-**5** in EtOH solution.

| Conformer | (*E*)-**5** | | (*Z*)-**5** | |
|---|---|---|---|---|
| | ΔE | ΔG | ΔE | ΔG |
| **A** | 0.10 | 0.00 | 0.34 | 1.50 |
| **B** | 0.56 | 0.01 | 1.89 | 2.99 |
| **C** | 0.00 | 0.41 | 2.33 | 2.02 |
| **D** | 0.48 | 0.03 | 1.04 | 1.70 |

### 14-Membered cyclic bis-thiosemicarbazone 6

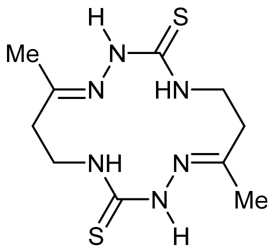


DMSO solution

**Data 21:** Cartesian coordinates and energies of the optimized geometry for the conformer **A** of macrocycle **6** in DMSO solution.

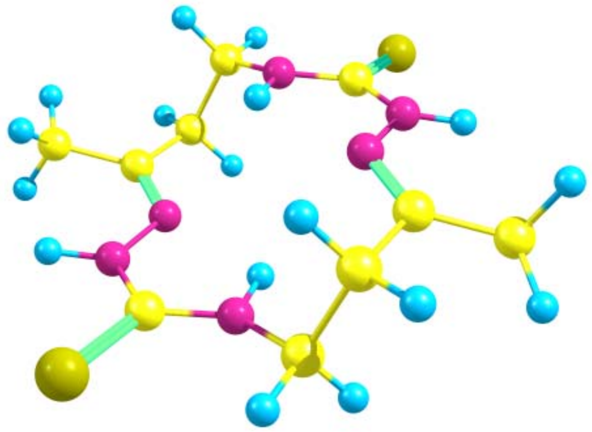

| | |
|---|---|
| Electronic Energy = | -1517.04666301 a.u. |
| Zero-point correction= | 0.295140 (Hartree/Particle) |
| Thermal correction to Energy= | 0.314454 |
| Thermal correction to Enthalpy= | 0.315398 |
| Thermal correction to Gibbs Free Energy= | 0.246360 |
| Sum of electronic and zero-point Energies= | -1516.751523 |
| Sum of electronic and thermal Energies= | -1516.732209 |
| Sum of electronic and thermal Enthalpies= | -1516.731265 |
| Sum of electronic and thermal Free Energies= | -1516.800303 |

Standard orientation:

| Center Number | Atomic Number | Atomic Type | Coordinates (Angstroms) X | Y | Z |
|---|---|---|---|---|---|
| 1 | 16 | 0 | -1.695863 | 0.257499 | -3.948672 |
| 2 | 7 | 0 | -1.123459 | -1.449553 | -0.457243 |
| 3 | 7 | 0 | -1.658867 | -1.094117 | -1.669813 |
| 4 | 1 | 0 | -2.632364 | -1.252625 | -1.900707 |
| 5 | 7 | 0 | 0.320975 | -0.054198 | -2.160317 |
| 6 | 1 | 0 | 0.570266 | -0.426910 | -1.249524 |
| 7 | 6 | 0 | -0.943359 | -0.303970 | -2.529591 |
| 8 | 6 | 0 | 1.182958 | 0.984302 | -2.709261 |
| 9 | 1 | 0 | 0.790385 | 1.269715 | -3.683327 |
| 10 | 1 | 0 | 2.182630 | 0.567217 | -2.853372 |
| 11 | 6 | 0 | 1.237278 | 2.225369 | -1.784513 |
| 12 | 1 | 0 | 1.793624 | 3.011679 | -2.300488 |
| 13 | 1 | 0 | 0.218802 | 2.582975 | -1.621638 |
| 14 | 6 | 0 | 1.893975 | 1.923440 | -0.456781 |
| 15 | 6 | 0 | 3.378341 | 2.128408 | -0.304846 |
| 16 | 1 | 0 | 3.866131 | 1.257210 | 0.143118 |
| 17 | 1 | 0 | 3.585061 | 2.992670 | 0.336734 |
| 18 | 1 | 0 | 3.844555 | 2.316503 | -1.271222 |
| 19 | 16 | 0 | 1.695863 | -0.257499 | 3.948672 |
| 20 | 7 | 0 | 1.123459 | 1.449553 | 0.457243 |
| 21 | 7 | 0 | 1.658867 | 1.094117 | 1.669813 |
| 22 | 1 | 0 | 2.632364 | 1.252625 | 1.900707 |
| 23 | 7 | 0 | -0.320975 | 0.054198 | 2.160317 |
| 24 | 1 | 0 | -0.570266 | 0.426910 | 1.249524 |
| 25 | 6 | 0 | 0.943359 | 0.303970 | 2.529591 |
| 26 | 6 | 0 | -1.182958 | -0.984302 | 2.709261 |
| 27 | 1 | 0 | -0.790385 | -1.269715 | 3.683327 |
| 28 | 1 | 0 | -2.182630 | -0.567217 | 2.853372 |
| 29 | 6 | 0 | -1.237278 | -2.225369 | 1.784513 |
| 30 | 1 | 0 | -1.793624 | -3.011679 | 2.300488 |
| 31 | 1 | 0 | -0.218802 | -2.582975 | 1.621638 |
| 32 | 6 | 0 | -1.893975 | -1.923440 | 0.456781 |
| 33 | 6 | 0 | -3.378341 | -2.128408 | 0.304846 |
| 34 | 1 | 0 | -3.866131 | -1.257210 | -0.143118 |
| 35 | 1 | 0 | -3.585061 | -2.992670 | -0.336734 |
| 36 | 1 | 0 | -3.844555 | -2.316503 | 1.271222 |

**Data 22:** Cartesian coordinates and energies of the optimized geometry for the conformer **B** of macrocycle **6** in DMSO solution.

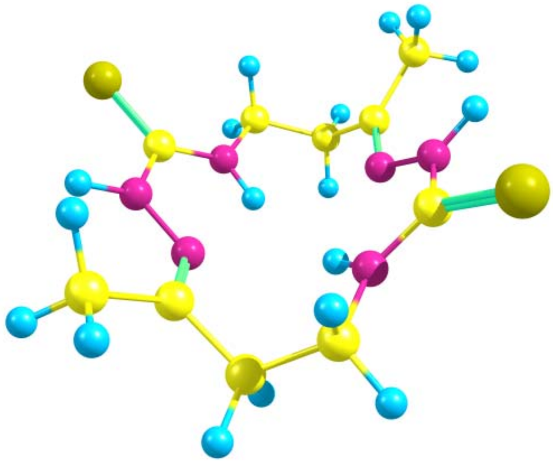

Electronic Energy = -1517.04598783 a.u.
Zero-point correction= 0.294624 (Hartree/Particle)
Thermal correction to Energy= 0.314273
Thermal correction to Enthalpy= 0.315218
Thermal correction to Gibbs Free Energy= 0.245178

Sum of electronic and zero-point Energies= -1516.751364
Sum of electronic and thermal Energies= -1516.731714
Sum of electronic and thermal Enthalpies= -1516.730770
Sum of electronic and thermal Free Energies= -1516.800810

Standard orientation:

| Center Number | Atomic Number | Atomic Type | Coordinates (Angstroms) X | Y | Z |
|---|---|---|---|---|---|
| 1 | 16 | 0 | 3.923136 | 0.726535 | 1.101877 |
| 2 | 16 | 0 | -3.923136 | -0.726533 | 1.101882 |
| 3 | 7 | 0 | 0.543836 | 1.828592 | -0.590532 |
| 4 | 7 | 0 | 1.712885 | 1.836206 | 0.126427 |
| 5 | 1 | 0 | 1.978942 | 2.608417 | 0.725280 |
| 6 | 7 | 0 | 2.081367 | -0.309288 | -0.576303 |
| 7 | 1 | 0 | 1.192582 | -0.168038 | -1.042735 |
| 8 | 7 | 0 | -0.543836 | -1.828594 | -0.590524 |
| 9 | 7 | 0 | -1.712886 | -1.836206 | 0.126434 |
| 10 | 1 | 0 | -1.978945 | -2.608416 | 0.725287 |
| 11 | 7 | 0 | -2.081368 | 0.309287 | -0.576301 |
| 12 | 1 | 0 | -1.192584 | 0.168036 | -1.042734 |
| 13 | 6 | 0 | 2.508761 | 0.720339 | 0.158547 |
| 14 | 6 | 0 | 2.632091 | -1.660567 | -0.583386 |
| 15 | 1 | 0 | 2.885226 | -1.952068 | 0.438798 |
| 16 | 1 | 0 | 3.553787 | -1.692336 | -1.171130 |
| 17 | 6 | 0 | 1.604981 | -2.628966 | -1.202006 |
| 18 | 1 | 0 | 1.360339 | -2.289888 | -2.211783 |
| 19 | 1 | 0 | 2.077864 | -3.608351 | -1.292413 |
| 20 | 6 | 0 | 0.333870 | -2.745298 | -0.386071 |
| 21 | 6 | 0 | -2.508761 | -0.720339 | 0.158552 |
| 22 | 6 | 0 | -2.632092 | 1.660566 | -0.583386 |
| 23 | 1 | 0 | -2.885225 | 1.952070 | 0.438798 |
| 24 | 1 | 0 | -3.553789 | 1.692333 | -1.171129 |
| 25 | 6 | 0 | -1.604983 | 2.628963 | -1.202010 |
| 26 | 1 | 0 | -1.360344 | 2.289883 | -2.211787 |
| 27 | 1 | 0 | -2.077865 | 3.608348 | -1.292418 |
| 28 | 6 | 0 | -0.333869 | 2.745297 | -0.386079 |
| 29 | 6 | 0 | 0.194831 | -3.861904 | 0.613053 |
| 30 | 1 | 0 | -0.198702 | -3.505091 | 1.569360 |
| 31 | 1 | 0 | 1.157839 | -4.336351 | 0.798482 |
| 32 | 1 | 0 | -0.487264 | -4.633711 | 0.238112 |
| 33 | 6 | 0 | -0.194826 | 3.861904 | 0.613042 |
| 34 | 1 | 0 | 0.198712 | 3.505092 | 1.569348 |
| 35 | 1 | 0 | -1.157834 | 4.336350 | 0.798476 |
| 36 | 1 | 0 | 0.487266 | 4.633712 | 0.238097 |

**Data 23:** Cartesian coordinates and energies of the optimized geometry for the conformer **C** of macrocycle **6** in DMSO solution.

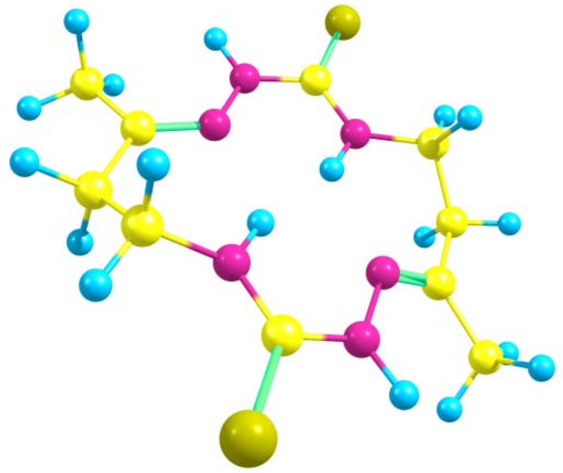

Electronic Energy = -1517.04658978 a.u.

| | |
|---|---|
| Zero-point correction= | 0.294471 (Hartree/Particle) |
| Thermal correction to Energy= | 0.314186 |
| Thermal correction to Enthalpy= | 0.315130 |
| Thermal correction to Gibbs Free Energy= | 0.244060 |
| Sum of electronic and zero-point Energies= | -1516.752119 |
| Sum of electronic and thermal Energies= | -1516.732404 |
| Sum of electronic and thermal Enthalpies= | -1516.731460 |
| Sum of electronic and thermal Free Energies= | -1516.802529 |

Standard orientation:

| Center Number | Atomic Number | Atomic Type | Coordinates (Angstroms) X | Y | Z |
|---|---|---|---|---|---|
| 1 | 7 | 0 | -1.615449 | -1.108421 | -0.111951 |
| 2 | 6 | 0 | -2.696292 | -0.329120 | -0.169085 |
| 3 | 7 | 0 | -2.462309 | 1.008892 | 0.025464 |
| 4 | 7 | 0 | -1.178598 | 1.497438 | 0.058358 |
| 5 | 7 | 0 | 1.178598 | -1.497438 | 0.058357 |
| 6 | 7 | 0 | 2.462309 | -1.008892 | 0.025459 |
| 7 | 6 | 0 | 2.696292 | 0.329120 | -0.169091 |
| 8 | 7 | 0 | 1.615448 | 1.108421 | -0.111960 |
| 9 | 6 | 0 | -0.975252 | 2.731105 | 0.355262 |
| 10 | 6 | 0 | 0.453190 | 3.243428 | 0.380338 |
| 11 | 6 | 0 | 1.502250 | 2.515990 | -0.468941 |
| 12 | 6 | 0 | -1.502251 | -2.515990 | -0.468933 |
| 13 | 6 | 0 | -0.453189 | -3.243428 | 0.380342 |
| 14 | 6 | 0 | 0.975253 | -2.731105 | 0.355263 |
| 15 | 1 | 0 | -0.722909 | -0.642478 | -0.011567 |
| 16 | 1 | 0 | -3.267185 | 1.622038 | -0.008280 |
| 17 | 1 | 0 | 3.267185 | -1.622038 | -0.008283 |
| 18 | 1 | 0 | 0.722908 | 0.642477 | -0.011575 |
| 19 | 1 | 0 | 0.438330 | 4.286481 | 0.050196 |
| 20 | 1 | 0 | 0.776199 | 3.270782 | 1.429568 |
| 21 | 1 | 0 | 2.472916 | 2.989761 | -0.328839 |
| 22 | 1 | 0 | 1.241055 | 2.600324 | -1.530303 |
| 23 | 1 | 0 | -2.472917 | -2.989762 | -0.328829 |
| 24 | 1 | 0 | -1.241059 | -2.600323 | -1.530296 |
| 25 | 1 | 0 | -0.438329 | -4.286481 | 0.050200 |
| 26 | 1 | 0 | -0.776195 | -3.270783 | 1.429573 |
| 27 | 16 | 0 | 4.294190 | 0.860296 | -0.422579 |
| 28 | 16 | 0 | -4.294190 | -0.860296 | -0.422572 |
| 29 | 6 | 0 | 2.060208 | -3.707773 | 0.722767 |
| 30 | 1 | 0 | 2.810158 | -3.253142 | 1.375388 |
| 31 | 1 | 0 | 1.639156 | -4.572475 | 1.235434 |
| 32 | 1 | 0 | 2.570434 | -4.074506 | -0.175516 |
| 33 | 6 | 0 | -2.060206 | 3.707774 | 0.722768 |
| 34 | 1 | 0 | -2.570432 | 4.074508 | -0.175514 |
| 35 | 1 | 0 | -2.810156 | 3.253143 | 1.375389 |
| 36 | 1 | 0 | -1.639153 | 4.572476 | 1.235435 |

**Data 24:** Cartesian coordinates and energies of the optimized geometry for the conformer **D** of macrocycle **6** in DMSO solution.

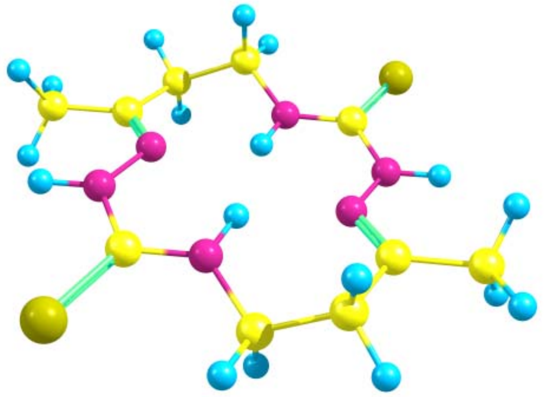

| | |
|---|---|
| Electronic Energy = | -1517.04453787 a.u. |
| Zero-point correction= | 0.294554 (Hartree/Particle) |
| Thermal correction to Energy= | 0.314213 |
| Thermal correction to Enthalpy= | 0.315157 |
| Thermal correction to Gibbs Free Energy= | 0.244986 |
| Sum of electronic and zero-point Energies= | -1516.749984 |
| Sum of electronic and thermal Energies= | -1516.730325 |
| Sum of electronic and thermal Enthalpies= | -1516.729381 |
| Sum of electronic and thermal Free Energies= | -1516.799552 |

Standard orientation:

| Center Number | Atomic Number | Atomic Type | Coordinates (Angstroms) X | Y | Z |
|---|---|---|---|---|---|
| 1 | 6 | 0 | -2.695716 | 0.392440 | 0.091575 |
| 2 | 16 | 0 | -4.299844 | 0.955521 | 0.004130 |
| 3 | 7 | 0 | -2.474765 | -0.954024 | 0.235902 |
| 4 | 7 | 0 | -1.207431 | -1.475061 | 0.131793 |
| 5 | 6 | 0 | -1.061050 | -2.741399 | -0.034547 |
| 6 | 7 | 0 | -1.600937 | 1.155292 | 0.043654 |
| 7 | 6 | 0 | -1.516232 | 2.537176 | -0.414524 |
| 8 | 6 | 0 | 2.695716 | -0.392440 | -0.091575 |
| 9 | 16 | 0 | 4.299844 | -0.955521 | -0.004130 |
| 10 | 7 | 0 | 2.474765 | 0.954024 | -0.235902 |
| 11 | 7 | 0 | 1.207431 | 1.475061 | -0.131793 |
| 12 | 6 | 0 | 1.061050 | 2.741399 | 0.034547 |
| 13 | 6 | 0 | -0.340884 | 3.294504 | 0.210691 |
| 14 | 7 | 0 | 1.600937 | -1.155292 | -0.043654 |
| 15 | 6 | 0 | 1.516232 | -2.537176 | 0.414524 |
| 16 | 1 | 0 | -3.296077 | -1.546200 | 0.231219 |
| 17 | 1 | 0 | -0.710031 | 0.674490 | 0.047029 |
| 18 | 1 | 0 | 3.296077 | 1.546200 | -0.231219 |
| 19 | 1 | 0 | 0.710031 | -0.674490 | -0.047029 |
| 20 | 6 | 0 | 0.340884 | -3.294504 | -0.210691 |
| 21 | 1 | 0 | -1.420466 | 2.547193 | -1.506898 |
| 22 | 1 | 0 | -2.445280 | 3.045912 | -0.161237 |
| 23 | 1 | 0 | -0.507151 | 3.416820 | 1.289469 |
| 24 | 1 | 0 | -0.358024 | 4.305746 | -0.205393 |
| 25 | 1 | 0 | 1.420466 | -2.547193 | 1.506898 |
| 26 | 1 | 0 | 2.445280 | -3.045912 | 0.161237 |
| 27 | 1 | 0 | 0.507151 | -3.416820 | -1.289469 |
| 28 | 1 | 0 | 0.358024 | -4.305746 | 0.205393 |
| 29 | 6 | 0 | 2.204431 | 3.717100 | 0.140021 |
| 30 | 1 | 0 | 2.960239 | 3.376294 | 0.854266 |
| 31 | 1 | 0 | 2.695258 | 3.846060 | -0.831206 |
| 32 | 1 | 0 | 1.850799 | 4.694361 | 0.465345 |
| 33 | 6 | 0 | -2.204431 | -3.717100 | -0.140021 |
| 34 | 1 | 0 | -2.960239 | -3.376294 | -0.854266 |
| 35 | 1 | 0 | -1.850799 | -4.694361 | -0.465345 |
| 36 | 1 | 0 | -2.695258 | -3.846060 | 0.831206 |

## EtOH solution

**Data 25:** Cartesian coordinates and energies of the optimized geometry for the conformer **A** of macrocycle **6** in EtOH solution.

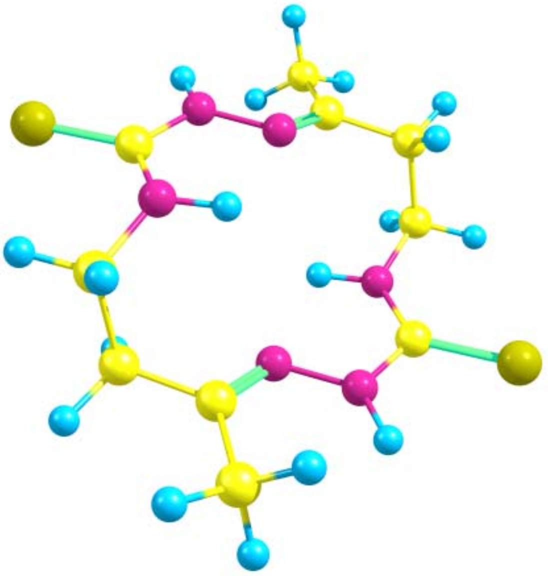

| | |
|---|---|
| Electronic Energy = | -1517.04586853 a.u. |
| Zero-point correction= | 0.295150 (Hartree/Particle) |
| Thermal correction to Energy= | 0.314461 |
| Thermal correction to Enthalpy= | 0.315405 |
| Thermal correction to Gibbs Free Energy= | 0.246372 |
| Sum of electronic and zero-point Energies= | -1516.750718 |
| Sum of electronic and thermal Energies= | -1516.731408 |
| Sum of electronic and thermal Enthalpies= | -1516.730464 |
| Sum of electronic and thermal Free Energies= | -1516.799497 |

Standard orientation:

| Center Number | Atomic Number | Atomic Type | Coordinates (Angstroms) X | Y | Z |
|---|---|---|---|---|---|
| 1 | 16 | 0 | -1.696424 | 0.258517 | -3.947331 |
| 2 | 7 | 0 | -1.124854 | -1.447806 | -0.456049 |
| 3 | 7 | 0 | -1.659811 | -1.092983 | -1.668681 |
| 4 | 1 | 0 | -2.633308 | -1.250963 | -1.899933 |
| 5 | 7 | 0 | 0.320904 | -0.054962 | -2.160101 |
| 6 | 1 | 0 | 0.569640 | -0.427099 | -1.248969 |
| 7 | 6 | 0 | -0.943958 | -0.303386 | -2.529361 |
| 8 | 6 | 0 | 1.183128 | 0.982846 | -2.709615 |
| 9 | 1 | 0 | 0.790262 | 1.267792 | -3.683731 |
| 10 | 1 | 0 | 2.182632 | 0.565270 | -2.853889 |
| 11 | 6 | 0 | 1.238343 | 2.224440 | -1.785574 |
| 12 | 1 | 0 | 1.794895 | 3.010265 | -2.302118 |
| 13 | 1 | 0 | 0.220030 | 2.582650 | -1.623014 |
| 14 | 6 | 0 | 1.894878 | 1.923312 | -0.457538 |
| 15 | 6 | 0 | 3.378896 | 2.130694 | -0.304130 |
| 16 | 1 | 0 | 3.868322 | 1.257998 | 0.139291 |
| 17 | 1 | 0 | 3.583915 | 2.991711 | 0.342370 |
| 18 | 1 | 0 | 3.845238 | 2.325023 | -1.269221 |
| 19 | 16 | 0 | 1.696424 | -0.258517 | 3.947331 |
| 20 | 7 | 0 | 1.124854 | 1.447806 | 0.456049 |
| 21 | 7 | 0 | 1.659811 | 1.092983 | 1.668681 |
| 22 | 1 | 0 | 2.633308 | 1.250963 | 1.899933 |
| 23 | 7 | 0 | -0.320904 | 0.054962 | 2.160101 |
| 24 | 1 | 0 | -0.569640 | 0.427099 | 1.248969 |
| 25 | 6 | 0 | 0.943958 | 0.303386 | 2.529361 |
| 26 | 6 | 0 | -1.183128 | -0.982846 | 2.709615 |
| 27 | 1 | 0 | -0.790262 | -1.267792 | 3.683731 |
| 28 | 1 | 0 | -2.182632 | -0.565270 | 2.853889 |
| 29 | 6 | 0 | -1.238343 | -2.224440 | 1.785574 |
| 30 | 1 | 0 | -1.794895 | -3.010265 | 2.302118 |
| 31 | 1 | 0 | -0.220030 | -2.582650 | 1.623014 |
| 32 | 6 | 0 | -1.894878 | -1.923312 | 0.457538 |
| 33 | 6 | 0 | -3.378896 | -2.130694 | 0.304130 |
| 34 | 1 | 0 | -3.868322 | -1.257998 | -0.139291 |

| | | | | | |
|---|---|---|---|---|---|
| 35 | 1 | 0 | -3.583915 | -2.991711 | -0.342370 |
| 36 | 1 | 0 | -3.845238 | -2.325023 | 1.269221 |

**Data 26:** Cartesian coordinates and energies of the optimized geometry for the conformer **B** of macrocycle **6** in EtOH solution.

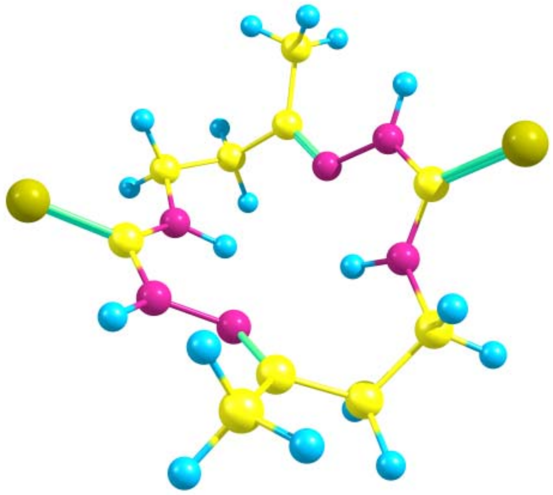

| | |
|---|---|
| Electronic Energy = | -1517.04518730 a.u. |
| Zero-point correction= | 0.294691 (Hartree/Particle) |
| Thermal correction to Energy= | 0.314298 |
| Thermal correction to Enthalpy= | 0.315242 |
| Thermal correction to Gibbs Free Energy= | 0.245470 |
| Sum of electronic and zero-point Energies= | -1516.750496 |
| Sum of electronic and thermal Energies= | -1516.730890 |
| Sum of electronic and thermal Enthalpies= | -1516.729945 |
| Sum of electronic and thermal Free Energies= | -1516.799717 |

Standard orientation:

| Center Number | Atomic Number | Atomic Type | Coordinates (Angstroms) X | Y | Z |
|---|---|---|---|---|---|
| 1 | 16 | 0 | 3.916348 | -0.735806 | -1.106445 |
| 2 | 16 | 0 | -3.916346 | 0.735803 | -1.106453 |
| 3 | 7 | 0 | 0.537781 | -1.829606 | 0.591488 |
| 4 | 7 | 0 | 1.706093 | -1.840425 | -0.126103 |
| 5 | 1 | 0 | 1.967111 | -2.610995 | -0.729272 |
| 6 | 7 | 0 | 2.082954 | 0.303082 | 0.578748 |
| 7 | 1 | 0 | 1.194220 | 0.164189 | 1.045884 |
| 8 | 7 | 0 | -0.537782 | 1.829607 | 0.591482 |
| 9 | 7 | 0 | -1.706093 | 1.840425 | -0.126110 |
| 10 | 1 | 0 | -1.967110 | 2.610993 | -0.729282 |
| 11 | 7 | 0 | -2.082955 | -0.303080 | 0.578745 |
| 12 | 1 | 0 | -1.194221 | -0.164186 | 1.045883 |
| 13 | 6 | 0 | 2.505586 | -0.726610 | -0.159213 |
| 14 | 6 | 0 | 2.637502 | 1.652663 | 0.585353 |
| 15 | 1 | 0 | 2.891534 | 1.942909 | -0.436979 |
| 16 | 1 | 0 | 3.559513 | 1.681955 | 1.172786 |
| 17 | 6 | 0 | 1.613172 | 2.624249 | 1.203714 |
| 18 | 1 | 0 | 1.367475 | 2.286102 | 2.213592 |
| 19 | 1 | 0 | 2.088883 | 3.602304 | 1.294135 |
| 20 | 6 | 0 | 0.342395 | 2.744093 | 0.387786 |
| 21 | 6 | 0 | -2.505586 | 0.726609 | -0.159219 |
| 22 | 6 | 0 | -2.637503 | -1.652662 | 0.585353 |
| 23 | 1 | 0 | -2.891533 | -1.942910 | -0.436979 |
| 24 | 1 | 0 | -3.559515 | -1.681952 | 1.172785 |
| 25 | 6 | 0 | -1.613174 | -2.624246 | 1.203719 |
| 26 | 1 | 0 | -1.367479 | -2.286097 | 2.213596 |
| 27 | 1 | 0 | -2.088885 | -3.602301 | 1.294141 |

| | | | | | |
|---|---|---|---|---|---|
| 28 | 6 | 0 | -0.342395 | -2.744092 | 0.387792 |
| 29 | 6 | 0 | 0.205731 | 3.860977 | -0.611540 |
| 30 | 1 | 0 | -0.181080 | 3.503042 | -1.570268 |
| 31 | 1 | 0 | 1.168181 | 4.338583 | -0.791533 |
| 32 | 1 | 0 | -0.481301 | 4.630413 | -0.240694 |
| 33 | 6 | 0 | -0.205730 | -3.860978 | -0.611531 |
| 34 | 1 | 0 | 0.181083 | -3.503046 | -1.570259 |
| 35 | 1 | 0 | -1.168181 | -4.338583 | -0.791525 |
| 36 | 1 | 0 | 0.481299 | -4.630414 | -0.240682 |

**Data 27:** Cartesian coordinates and energies of the optimized geometry for the conformer **C** of macrocycle **6** in EtOH solution.

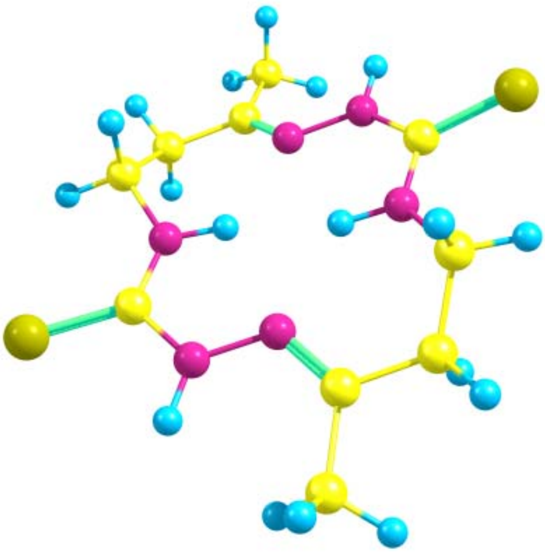

| | |
|---|---|
| Electronic Energy = | -1517.04581037 a.u. |
| Zero-point correction= | 0.294504 (Hartree/Particle) |
| Thermal correction to Energy= | 0.314205 |
| Thermal correction to Enthalpy= | 0.315149 |
| Thermal correction to Gibbs Free Energy= | 0.244158 |
| Sum of electronic and zero-point Energies= | -1516.751306 |
| Sum of electronic and thermal Energies= | -1516.731606 |
| Sum of electronic and thermal Enthalpies= | -1516.730661 |
| Sum of electronic and thermal Free Energies= | -1516.801652 |

Standard orientation:

| Center Number | Atomic Number | Atomic Type | Coordinates (Angstroms) X | Y | Z |
|---|---|---|---|---|---|
| 1 | 7 | 0 | -1.614525 | -1.109210 | -0.111517 |
| 2 | 6 | 0 | -2.696405 | -0.330889 | -0.167887 |
| 3 | 7 | 0 | -2.462827 | 1.007571 | 0.027311 |
| 4 | 7 | 0 | -1.179783 | 1.497098 | 0.059198 |
| 5 | 7 | 0 | 1.179783 | -1.497098 | 0.059196 |
| 6 | 7 | 0 | 2.462827 | -1.007571 | 0.027307 |
| 7 | 6 | 0 | 2.696404 | 0.330889 | -0.167893 |
| 8 | 7 | 0 | 1.614524 | 1.109210 | -0.111526 |
| 9 | 6 | 0 | -0.976860 | 2.731344 | 0.353873 |
| 10 | 6 | 0 | 0.451453 | 3.244295 | 0.378456 |
| 11 | 6 | 0 | 1.500845 | 2.516234 | -0.469958 |
| 12 | 6 | 0 | -1.500846 | -2.516234 | -0.469950 |
| 13 | 6 | 0 | -0.451452 | -3.244295 | 0.378460 |
| 14 | 6 | 0 | 0.976861 | -2.731344 | 0.353873 |
| 15 | 1 | 0 | -0.722339 | -0.642604 | -0.011731 |
| 16 | 1 | 0 | -3.268441 | 1.619740 | -0.006176 |
| 17 | 1 | 0 | 3.268441 | -1.619740 | -0.006179 |

| | | | | | |
|---|---|---|---|---|---|
| 18 | 1 | 0 | 0.722338 | 0.642603 | -0.011740 |
| 19 | 1 | 0 | 0.436279 | 4.287009 | 0.047128 |
| 20 | 1 | 0 | 0.774619 | 3.272907 | 1.427634 |
| 21 | 1 | 0 | 2.471490 | 2.990253 | -0.330403 |
| 22 | 1 | 0 | 1.239759 | 2.599474 | -1.531471 |
| 23 | 1 | 0 | -2.471491 | -2.990254 | -0.330393 |
| 24 | 1 | 0 | -1.239763 | -2.599473 | -1.531465 |
| 25 | 1 | 0 | -0.436278 | -4.287009 | 0.047132 |
| 26 | 1 | 0 | -0.774615 | -3.272908 | 1.427639 |
| 27 | 16 | 0 | 4.293417 | 0.862518 | -0.420562 |
| 28 | 16 | 0 | -4.293418 | -0.862517 | -0.420556 |
| 29 | 6 | 0 | 2.062571 | -3.708048 | 0.719579 |
| 30 | 1 | 0 | 2.811414 | -3.254441 | 1.374252 |
| 31 | 1 | 0 | 1.642104 | -4.574666 | 1.229526 |
| 32 | 1 | 0 | 2.574276 | -4.071789 | -0.179115 |
| 33 | 6 | 0 | -2.062569 | 3.708049 | 0.719580 |
| 34 | 1 | 0 | -2.574274 | 4.071791 | -0.179113 |
| 35 | 1 | 0 | -2.811412 | 3.254442 | 1.374253 |
| 36 | 1 | 0 | -1.642101 | 4.574667 | 1.229527 |

**Data 28:** Cartesian coordinates and energies of the optimized geometry for the conformer **D** of macrocycle **6** in EtOH solution.

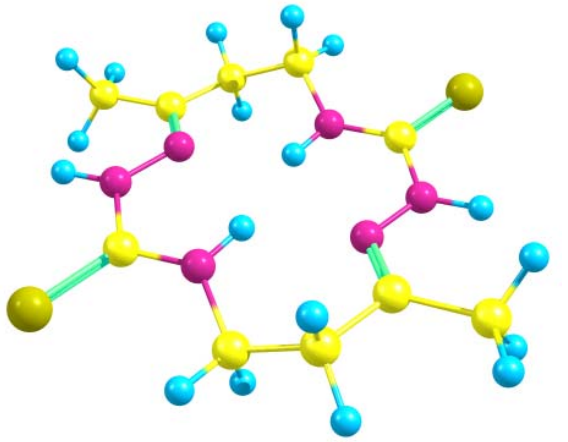

| | |
|---|---|
| Electronic Energy = | -1517.04377017 a.u. |
| Zero-point correction= | 0.294544 (Hartree/Particle) |
| Thermal correction to Energy= | 0.314216 |
| Thermal correction to Enthalpy= | 0.315160 |
| Thermal correction to Gibbs Free Energy= | 0.244912 |
| Sum of electronic and zero-point Energies= | -1516.749226 |
| Sum of electronic and thermal Energies= | -1516.729555 |
| Sum of electronic and thermal Enthalpies= | -1516.728610 |
| Sum of electronic and thermal Free Energies= | -1516.798858 |

Standard orientation:

| Center Number | Atomic Number | Atomic Type | Coordinates (Angstroms) X | Y | Z |
|---|---|---|---|---|---|
| 1 | 6 | 0 | -2.696058 | 0.392411 | 0.091409 |
| 2 | 16 | 0 | -4.299347 | 0.954464 | -0.001236 |
| 3 | 7 | 0 | -2.474788 | -0.954461 | 0.236268 |
| 4 | 7 | 0 | -1.207783 | -1.475495 | 0.131310 |
| 5 | 6 | 0 | -1.061231 | -2.741855 | -0.034473 |
| 6 | 7 | 0 | -1.600917 | 1.155458 | 0.047424 |
| 7 | 6 | 0 | -1.516319 | 2.536817 | -0.412144 |
| 8 | 6 | 0 | 2.696058 | -0.392411 | -0.091409 |
| 9 | 16 | 0 | 4.299347 | -0.954464 | 0.001236 |
| 10 | 7 | 0 | 2.474788 | 0.954461 | -0.236268 |
| 11 | 7 | 0 | 1.207783 | 1.475495 | -0.131310 |
| 12 | 6 | 0 | 1.061231 | 2.741855 | 0.034473 |

```
 13          6          0       -0.340705    3.294715    0.211882
 14          7          0        1.600917   -1.155458   -0.047424
 15          6          0        1.516319   -2.536817    0.412144
 16          1          0       -3.296370   -1.546217    0.226544
 17          1          0       -0.710015    0.674748    0.052345
 18          1          0        3.296370    1.546217   -0.226544
 19          1          0        0.710015   -0.674748   -0.052345
 20          6          0        0.340705   -3.294715   -0.211882
 21          1          0       -1.421172    2.546031   -1.504620
 22          1          0       -2.445371    3.045648   -0.158963
 23          1          0       -0.506327    3.417200    1.290761
 24          1          0       -0.358569    4.305885   -0.204441
 25          1          0        1.421172   -2.546031    1.504620
 26          1          0        2.445371   -3.045648    0.158963
 27          1          0        0.506327   -3.417200   -1.290761
 28          1          0        0.358569   -4.305885    0.204441
 29          6          0        2.204934    3.717540    0.138352
 30          1          0        2.959529    3.378747    0.854922
 31          1          0        2.697565    3.843091   -0.832427
 32          1          0        1.851503    4.696091    0.460040
 33          6          0       -2.204934   -3.717540   -0.138352
 34          1          0       -2.959529   -3.378747   -0.854922
 35          1          0       -1.851503   -4.696091   -0.460040
 36          1          0       -2.697565   -3.843091    0.832427
---------------------------------------------------------------------
```

**Table S15.** Relative electronic (ΔE, kcal/mol) and Gibbs free energies (ΔG, kcal/mol) of various conformers of **6** in DMSO and EtOH solution.

| Conformer | DMSO | | EtOH | |
|---|---|---|---|---|
| | ΔE | ΔG | ΔE | ΔG |
| **A** | 0.00 | 1.40 | 0.00 | 1.35 |
| **B** | 0.42 | 1.08 | 0.43 | 1.21 |
| **C** | 0.05 | 0.00 | 0.04 | 0.00 |
| **D** | 1.33 | 1.87 | 1.32 | 1.75 |

**28-Membered cyclic bis-thiosemicarbazone 7**

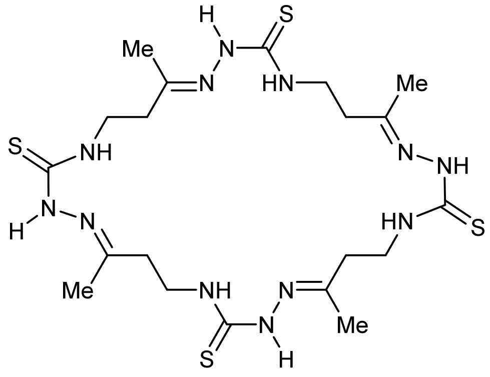

DMSO solution

**Data 29:** Cartesian coordinates and energies of the optimized geometry of macrocycle **7** in DMSO solution.

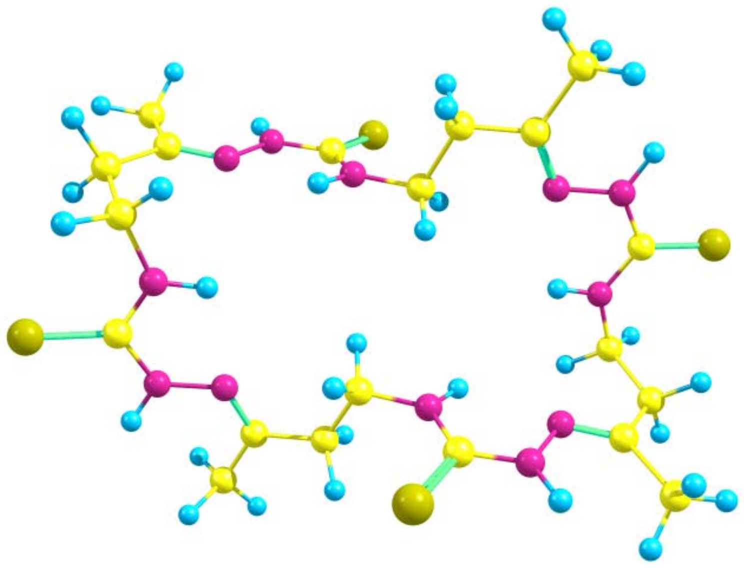

| | |
|---|---|
| Electronic Energy = | -3034.10654939 a.u. |
| Zero-point correction= | 0.589864 (Hartree/Particle) |
| Thermal correction to Energy= | 0.631246 |
| Thermal correction to Enthalpy= | 0.632190 |
| Thermal correction to Gibbs Free Energy= | 0.510025 |
| Sum of electronic and zero-point Energies= | -3033.516685 |
| Sum of electronic and thermal Energies= | -3033.475304 |
| Sum of electronic and thermal Enthalpies= | -3033.474359 |
| Sum of electronic and thermal Free Energies= | -3033.596524 |

Standard orientation:

| Center Number | Atomic Number | Atomic Type | Coordinates (Angstroms) X | Y | Z |
|---|---|---|---|---|---|
| 1 | 16 | 0 | -3.254529 | -3.202906 | 0.107148 |
| 2 | 16 | 0 | 1.282095 | -2.066184 | 6.753241 |
| 3 | 7 | 0 | -1.931200 | -2.010830 | -3.405752 |
| 4 | 7 | 0 | -2.677090 | -2.566537 | -2.394070 |
| 5 | 1 | 0 | -3.626382 | -2.886195 | -2.541711 |
| 6 | 7 | 0 | -0.973703 | -2.160836 | -0.909067 |
| 7 | 1 | 0 | -0.475585 | -1.851086 | -1.735061 |
| 8 | 7 | 0 | 0.790791 | -2.610700 | 2.893006 |
| 9 | 7 | 0 | 1.195309 | -2.762271 | 4.197512 |
| 10 | 1 | 0 | 1.423180 | -3.670539 | 4.584157 |
| 11 | 7 | 0 | 0.635693 | -0.574862 | 4.589667 |
| 12 | 1 | 0 | 0.535080 | -0.544632 | 3.582509 |
| 13 | 6 | 0 | -2.220722 | -2.603738 | -1.109158 |
| 14 | 6 | 0 | -0.291053 | -2.136705 | 0.381234 |
| 15 | 1 | 0 | -1.026638 | -1.917542 | 1.152853 |
| 16 | 1 | 0 | 0.424938 | -1.314643 | 0.356917 |
| 17 | 6 | 0 | 0.413311 | -3.462853 | 0.686203 |
| 18 | 1 | 0 | -0.287547 | -4.287190 | 0.508731 |
| 19 | 1 | 0 | 1.244033 | -3.623013 | -0.012137 |
| 20 | 6 | 0 | 0.928677 | -3.606522 | 2.094160 |
| 21 | 6 | 0 | 1.013020 | -1.750547 | 5.100534 |
| 22 | 6 | 0 | 0.376483 | 0.644551 | 5.349247 |
| 23 | 1 | 0 | -0.149430 | 0.373792 | 6.265419 |
| 24 | 1 | 0 | -0.291000 | 1.257667 | 4.743644 |
| 25 | 6 | 0 | 1.636841 | 1.426800 | 5.721388 |
| 26 | 1 | 0 | 1.355358 | 2.231084 | 6.413930 |
| 27 | 1 | 0 | 2.309052 | 0.776998 | 6.291686 |
| 28 | 6 | 0 | 2.421906 | 2.042764 | 4.592537 |
| 29 | 6 | 0 | 1.537367 | -4.930296 | 2.478329 |
| 30 | 1 | 0 | 1.649011 | -5.572425 | 1.605539 |
| 31 | 1 | 0 | 0.904616 | -5.458618 | 3.200705 |
| 32 | 1 | 0 | 2.523791 | -4.796573 | 2.933123 |
| 33 | 6 | 0 | 3.749008 | 2.664127 | 4.946763 |

| | | | | | |
|---|---|---|---|---|---|
| 34 | 1 | 0 | 3.808091 | 3.701418 | 4.602035 |
| 35 | 1 | 0 | 4.577655 | 2.111303 | 4.489772 |
| 36 | 1 | 0 | 3.902356 | 2.657746 | 6.025044 |
| 37 | 16 | 0 | 3.254529 | 3.202906 | -0.107148 |
| 38 | 16 | 0 | -1.282095 | 2.066184 | -6.753241 |
| 39 | 7 | 0 | 1.931200 | 2.010830 | 3.405752 |
| 40 | 7 | 0 | 2.677090 | 2.566537 | 2.394070 |
| 41 | 1 | 0 | 3.626382 | 2.886195 | 2.541711 |
| 42 | 7 | 0 | 0.973703 | 2.160836 | 0.909067 |
| 43 | 1 | 0 | 0.475585 | 1.851086 | 1.735061 |
| 44 | 7 | 0 | -0.790791 | 2.610700 | -2.893006 |
| 45 | 7 | 0 | -1.195309 | 2.762271 | -4.197512 |
| 46 | 1 | 0 | -1.423180 | 3.670539 | -4.584157 |
| 47 | 7 | 0 | -0.635693 | 0.574862 | -4.589667 |
| 48 | 1 | 0 | -0.535080 | 0.544632 | -3.582509 |
| 49 | 6 | 0 | 2.220722 | 2.603738 | 1.109158 |
| 50 | 6 | 0 | 0.291053 | 2.136705 | -0.381234 |
| 51 | 1 | 0 | 1.026638 | 1.917542 | -1.152853 |
| 52 | 1 | 0 | -0.424938 | 1.314643 | -0.356917 |
| 53 | 6 | 0 | -0.413311 | 3.462853 | -0.686203 |
| 54 | 1 | 0 | 0.287547 | 4.287190 | -0.508731 |
| 55 | 1 | 0 | -1.244033 | 3.623013 | 0.012137 |
| 56 | 6 | 0 | -0.928677 | 3.606522 | -2.094160 |
| 57 | 6 | 0 | -1.013020 | 1.750547 | -5.100534 |
| 58 | 6 | 0 | -0.376483 | -0.644551 | -5.349247 |
| 59 | 1 | 0 | 0.149430 | -0.373792 | -6.265419 |
| 60 | 1 | 0 | 0.291000 | -1.257667 | -4.743644 |
| 61 | 6 | 0 | -1.636841 | -1.426800 | -5.721388 |
| 62 | 1 | 0 | -1.355358 | -2.231084 | -6.413930 |
| 63 | 1 | 0 | -2.309052 | -0.776998 | -6.291686 |
| 64 | 6 | 0 | -2.421906 | -2.042764 | -4.592537 |
| 65 | 6 | 0 | -1.537367 | 4.930296 | -2.478329 |
| 66 | 1 | 0 | -1.649011 | 5.572425 | -1.605539 |
| 67 | 1 | 0 | -0.904616 | 5.458618 | -3.200705 |
| 68 | 1 | 0 | -2.523791 | 4.796573 | -2.933123 |
| 69 | 6 | 0 | -3.749008 | -2.664127 | -4.946763 |
| 70 | 1 | 0 | -3.808091 | -3.701418 | -4.602035 |
| 71 | 1 | 0 | -4.577655 | -2.111303 | -4.489772 |
| 72 | 1 | 0 | -3.902356 | -2.657746 | -6.025044 |

EtOH solution

**Data 30:** Cartesian coordinates and energies of the optimized geometry of macrocycle **7** in EtOH solution.

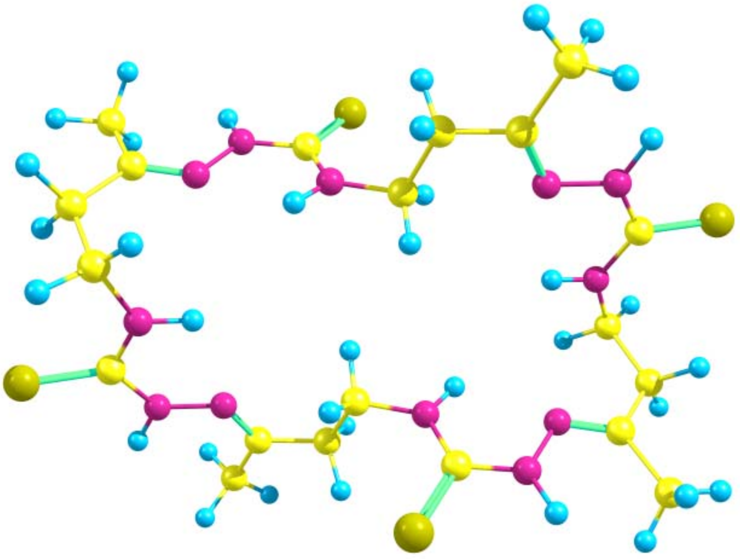

Electronic Energy = -3034.10497030 a.u.

| | |
|---|---|
| Zero-point correction= | 0.589909 (Hartree/Particle) |
| Thermal correction to Energy= | 0.631274 |
| Thermal correction to Enthalpy= | 0.632218 |
| Thermal correction to Gibbs Free Energy= | 0.510031 |
| Sum of electronic and zero-point Energies= | -3033.515062 |
| Sum of electronic and thermal Energies= | -3033.473697 |
| Sum of electronic and thermal Enthalpies= | -3033.472753 |
| Sum of electronic and thermal Free Energies= | -3033.594940 |

Standard orientation:

| Center Number | Atomic Number | Atomic Type | Coordinates (Angstroms) X | Y | Z |
|---|---|---|---|---|---|
| 1 | 16 | 0 | -3.251300 | -3.192577 | 0.108514 |
| 2 | 16 | 0 | 1.286085 | -2.066317 | 6.752408 |
| 3 | 7 | 0 | -1.929112 | -2.009101 | -3.407317 |
| 4 | 7 | 0 | -2.674613 | -2.562803 | -2.394371 |
| 5 | 1 | 0 | -3.624645 | -2.880962 | -2.540449 |
| 6 | 7 | 0 | -0.970655 | -2.153907 | -0.910797 |
| 7 | 1 | 0 | -0.472965 | -1.847041 | -1.738042 |
| 8 | 7 | 0 | 0.793987 | -2.606274 | 2.891847 |
| 9 | 7 | 0 | 1.198584 | -2.759020 | 4.196022 |
| 10 | 1 | 0 | 1.426214 | -3.667666 | 4.581896 |
| 11 | 7 | 0 | 0.634937 | -0.573221 | 4.591841 |
| 12 | 1 | 0 | 0.534257 | -0.541762 | 3.584826 |
| 13 | 6 | 0 | -2.218340 | -2.597110 | -1.109057 |
| 14 | 6 | 0 | -0.286887 | -2.130209 | 0.378888 |
| 15 | 1 | 0 | -1.021575 | -1.909107 | 1.150852 |
| 16 | 1 | 0 | 0.431107 | -1.309815 | 0.353428 |
| 17 | 6 | 0 | 0.414256 | -3.457852 | 0.684797 |
| 18 | 1 | 0 | -0.289261 | -4.280100 | 0.508174 |
| 19 | 1 | 0 | 1.244279 | -3.620898 | -0.013755 |
| 20 | 6 | 0 | 0.929489 | -3.602204 | 2.092759 |
| 21 | 6 | 0 | 1.014987 | -1.748970 | 5.101165 |
| 22 | 6 | 0 | 0.375857 | 0.644880 | 5.353258 |
| 23 | 1 | 0 | -0.147991 | 0.372394 | 6.270105 |
| 24 | 1 | 0 | -0.293394 | 1.258255 | 4.749765 |
| 25 | 6 | 0 | 1.636176 | 1.427873 | 5.723981 |
| 26 | 1 | 0 | 1.355009 | 2.232723 | 6.416038 |
| 27 | 1 | 0 | 2.308625 | 0.778525 | 6.294534 |
| 28 | 6 | 0 | 2.420446 | 2.042464 | 4.593843 |
| 29 | 6 | 0 | 1.534978 | -4.927511 | 2.476978 |
| 30 | 1 | 0 | 1.644883 | -5.570080 | 1.604267 |
| 31 | 1 | 0 | 0.900926 | -5.454185 | 3.199437 |
| 32 | 1 | 0 | 2.521846 | -4.796378 | 2.931620 |
| 33 | 6 | 0 | 3.748066 | 2.663880 | 4.946206 |
| 34 | 1 | 0 | 3.807611 | 3.700449 | 4.599337 |
| 35 | 1 | 0 | 4.576306 | 2.109702 | 4.490074 |
| 36 | 1 | 0 | 3.901966 | 2.659536 | 6.024432 |
| 37 | 16 | 0 | 3.251300 | 3.192577 | -0.108514 |
| 38 | 16 | 0 | -1.286085 | 2.066317 | -6.752408 |
| 39 | 7 | 0 | 1.929112 | 2.009101 | 3.407317 |
| 40 | 7 | 0 | 2.674613 | 2.562803 | 2.394371 |
| 41 | 1 | 0 | 3.624645 | 2.880962 | 2.540449 |
| 42 | 7 | 0 | 0.970655 | 2.153907 | 0.910797 |
| 43 | 1 | 0 | 0.472965 | 1.847041 | 1.738042 |
| 44 | 7 | 0 | -0.793987 | 2.606274 | -2.891847 |
| 45 | 7 | 0 | -1.198584 | 2.759020 | -4.196022 |
| 46 | 1 | 0 | -1.426214 | 3.667666 | -4.581896 |
| 47 | 7 | 0 | -0.634937 | 0.573221 | -4.591841 |
| 48 | 1 | 0 | -0.534257 | 0.541762 | -3.584826 |
| 49 | 6 | 0 | 2.218340 | 2.597110 | 1.109057 |
| 50 | 6 | 0 | 0.286887 | 2.130209 | -0.378888 |
| 51 | 1 | 0 | 1.021575 | 1.909107 | -1.150852 |
| 52 | 1 | 0 | -0.431107 | 1.309815 | -0.353428 |
| 53 | 6 | 0 | -0.414256 | 3.457852 | -0.684797 |
| 54 | 1 | 0 | 0.289261 | 4.280100 | -0.508174 |
| 55 | 1 | 0 | -1.244279 | 3.620898 | 0.013755 |
| 56 | 6 | 0 | -0.929489 | 3.602204 | -2.092759 |
| 57 | 6 | 0 | -1.014987 | 1.748970 | -5.101165 |
| 58 | 6 | 0 | -0.375857 | -0.644880 | -5.353258 |
| 59 | 1 | 0 | 0.147991 | -0.372394 | -6.270105 |
| 60 | 1 | 0 | 0.293394 | -1.258255 | -4.749765 |

| | | | | | |
|---|---|---|---|---|---|
| 61 | 6 | 0 | -1.636176 | -1.427873 | -5.723981 |
| 62 | 1 | 0 | -1.355009 | -2.232723 | -6.416038 |
| 63 | 1 | 0 | -2.308625 | -0.778525 | -6.294534 |
| 64 | 6 | 0 | -2.420446 | -2.042464 | -4.593843 |
| 65 | 6 | 0 | -1.534978 | 4.927511 | -2.476978 |
| 66 | 1 | 0 | -1.644883 | 5.570080 | -1.604267 |
| 67 | 1 | 0 | -0.900926 | 5.454185 | -3.199437 |
| 68 | 1 | 0 | -2.521846 | 4.796378 | -2.931620 |
| 69 | 6 | 0 | -3.748066 | -2.663880 | -4.946206 |
| 70 | 1 | 0 | -3.807611 | -3.700449 | -4.599337 |
| 71 | 1 | 0 | -4.576306 | -2.109702 | -4.490074 |
| 72 | 1 | 0 | -3.901966 | -2.659536 | -6.024432 |

***Discussion on acid-catalyzed cyclooligomerization of hydrazone 5:*** We assume that the acid-catalyzed transformation of hydrazone **5** proceeds through its dimerization to give **A**, which then futher dimerizes to form **B**. Cyclizations of dimer **A** and tetramer **B** afford macrocycles **6** and **7**, respectively (Scheme 1).

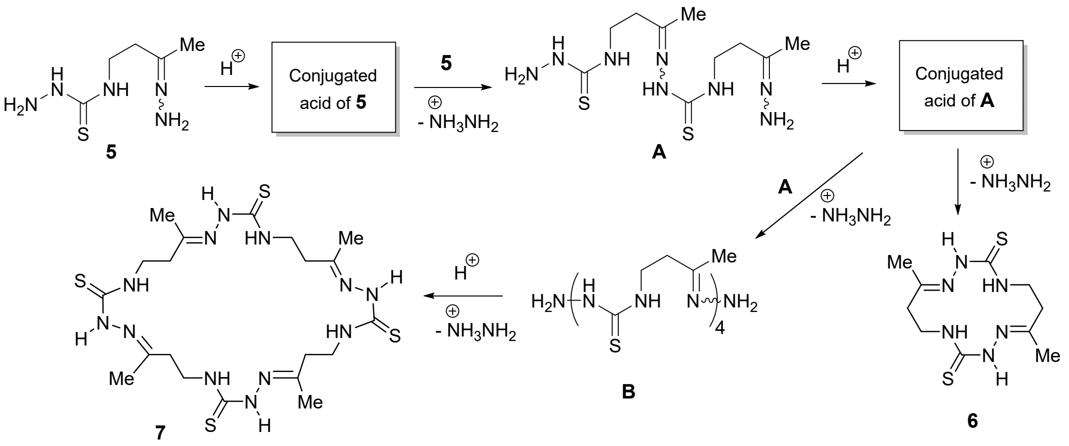


**Scheme 1.** Plausible pathways for the acid-catalyzed transformation of **5** into macrocycles **6** and **7**.

The formation of dimer **A** starts with the activation of hydrazone **5** by protonation with a Brønsted acid to give a conjugated acid of **5**. The favorable site of protonation of **5** possessing six potential basic centers (5 nitrogen and 1 sulfur atoms) was confirmed by the DFT calculations.

*Protonation of hydrazone (E)-**5** to give the conjugated acid (DMSO solution)*

**Data 31:** Cartesian coordinates and energies of the optimized geometry for the conformer-1 of the conjugated acid of (*E*)-**5** formed by protonation at the nitrogen of the C=N bond (DMSO solution).

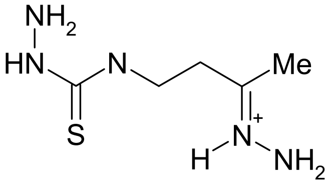


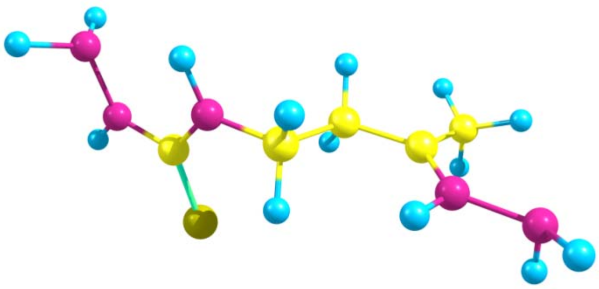

| | |
|---|---|
| Electronic Energy = | -870.889447507 a.u. |
| Zero-point correction= | 0.215139 (Hartree/Particle) |
| Thermal correction to Energy= | 0.228984 |
| Thermal correction to Enthalpy= | 0.229928 |
| Thermal correction to Gibbs Free Energy= | 0.173130 |
| Sum of electronic and zero-point Energies= | -870.674308 |
| Sum of electronic and thermal Energies= | -870.660463 |
| Sum of electronic and thermal Enthalpies= | -870.659519 |
| Sum of electronic and thermal Free Energies= | -870.716318 |

Standard orientation:

| Center Number | Atomic Number | Atomic Type | Coordinates (Angstroms) X | Y | Z |
|---|---|---|---|---|---|
| 1 | 7 | 0 | -3.788594 | -1.520990 | -0.078800 |
| 2 | 7 | 0 | -3.436567 | -0.179632 | 0.143757 |
| 3 | 6 | 0 | -2.171234 | 0.255990 | -0.064370 |
| 4 | 16 | 0 | -1.785363 | 1.897228 | 0.190483 |
| 5 | 7 | 0 | -1.294226 | -0.676276 | -0.473023 |
| 6 | 6 | 0 | 0.109328 | -0.441073 | -0.755292 |
| 7 | 6 | 0 | 0.962105 | -0.555266 | 0.517410 |
| 8 | 6 | 0 | 2.431223 | -0.320795 | 0.402014 |
| 9 | 7 | 0 | 2.958401 | -0.011483 | -0.739659 |
| 10 | 6 | 0 | 3.280641 | -0.408455 | 1.616563 |
| 11 | 7 | 0 | 4.328438 | 0.238409 | -0.906679 |
| 12 | 1 | 0 | -4.501115 | -1.573343 | -0.801501 |
| 13 | 1 | 0 | -4.159140 | -1.923586 | 0.777267 |
| 14 | 1 | 0 | -4.126081 | 0.498412 | 0.439029 |
| 15 | 1 | 0 | -1.671984 | -1.612820 | -0.573114 |
| 16 | 1 | 0 | 4.553458 | 0.096002 | -1.886415 |
| 17 | 1 | 0 | 4.520151 | 1.210345 | -0.669671 |
| 18 | 1 | 0 | 0.204139 | 0.553589 | -1.195875 |
| 19 | 1 | 0 | 0.413166 | -1.184649 | -1.494934 |
| 20 | 1 | 0 | 0.594416 | 0.154949 | 1.268052 |
| 21 | 1 | 0 | 0.841502 | -1.546797 | 0.968293 |
| 22 | 1 | 0 | 3.409551 | 0.597053 | 2.033482 |
| 23 | 1 | 0 | 2.798271 | -1.026358 | 2.371626 |
| 24 | 1 | 0 | 4.271380 | -0.789700 | 1.367228 |
| 25 | 1 | 0 | 2.373544 | 0.058661 | -1.568321 |

**Data 32:** Cartesian coordinates and energies of the optimized geometry for the conformer-2 of the conjugated acid of (*E*)-**5** formed by protonation at the nitrogen of the C=N bond (DMSO solution).

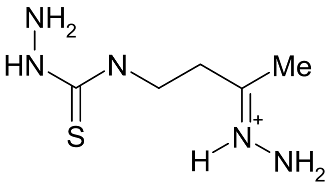


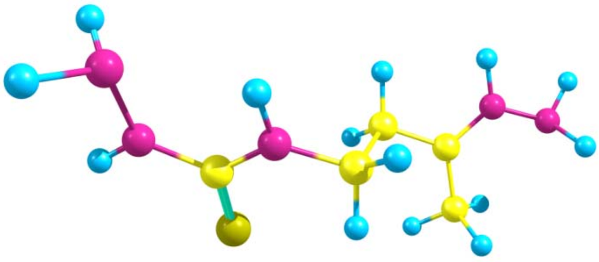

| | |
|---|---|
| Electronic Energy = | -870.891417374 a.u. |
| Zero-point correction= | 0.215899 (Hartree/Particle) |
| Thermal correction to Energy= | 0.229474 |
| Thermal correction to Enthalpy= | 0.230419 |
| Thermal correction to Gibbs Free Energy= | 0.174643 |
| Sum of electronic and zero-point Energies= | -870.675518 |
| Sum of electronic and thermal Energies= | -870.661943 |
| Sum of electronic and thermal Enthalpies= | -870.660999 |
| Sum of electronic and thermal Free Energies= | -870.716774 |

Standard orientation:

| Center Number | Atomic Number | Atomic Type | Coordinates (Angstroms) X | Y | Z |
|---|---|---|---|---|---|
| 1 | 7 | 0 | -3.643888 | -1.770688 | 0.017431 |
| 2 | 7 | 0 | -3.422643 | -0.398210 | 0.217189 |
| 3 | 6 | 0 | -2.216904 | 0.162006 | -0.042403 |
| 4 | 16 | 0 | -1.989282 | 1.834751 | 0.195358 |
| 5 | 7 | 0 | -1.267974 | -0.679128 | -0.487784 |
| 6 | 6 | 0 | 0.098553 | -0.316547 | -0.798712 |
| 7 | 6 | 0 | 0.997746 | -0.353485 | 0.467286 |
| 8 | 6 | 0 | 2.404403 | 0.048958 | 0.180177 |
| 9 | 7 | 0 | 3.339340 | -0.834494 | 0.334137 |
| 10 | 6 | 0 | 2.740492 | 1.413556 | -0.295528 |
| 11 | 7 | 0 | 4.693113 | -0.576173 | 0.104321 |
| 12 | 1 | 0 | -4.374408 | -1.903397 | -0.676421 |
| 13 | 1 | 0 | -3.938287 | -2.199769 | 0.890013 |
| 14 | 1 | 0 | -4.165095 | 0.208397 | 0.538514 |
| 15 | 1 | 0 | -1.550622 | -1.651533 | -0.556661 |
| 16 | 1 | 0 | 5.236192 | -1.322953 | 0.524695 |
| 17 | 1 | 0 | 4.874942 | -0.560830 | -0.897223 |
| 18 | 1 | 0 | 0.107222 | 0.679511 | -1.239840 |
| 19 | 1 | 0 | 0.469164 | -1.027580 | -1.537996 |
| 20 | 1 | 0 | 0.594032 | 0.363483 | 1.189267 |
| 21 | 1 | 0 | 0.962248 | -1.349594 | 0.912562 |
| 22 | 1 | 0 | 2.812261 | 1.415875 | -1.389604 |
| 23 | 1 | 0 | 1.963739 | 2.119367 | -0.006532 |
| 24 | 1 | 0 | 3.713125 | 1.720953 | 0.093233 |
| 25 | 1 | 0 | 3.092620 | -1.764024 | 0.668293 |

**Data 33:** Cartesian coordinates and energies of the optimized geometry for the conformer-1 of the conjugated acid of (*E*)-**5** formed by protonation at the $NH_2$ group of the hydrazone fragment (DMSO solution).

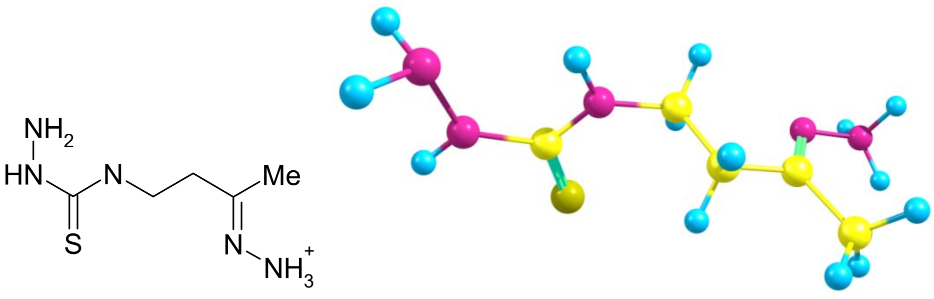


| | |
|---|---|
| Electronic Energy = | -870.882944144 a.u. |
| Zero-point correction= | 0.216236 (Hartree/Particle) |
| Thermal correction to Energy= | 0.229876 |
| Thermal correction to Enthalpy= | 0.230821 |
| Thermal correction to Gibbs Free Energy= | 0.174695 |
| Sum of electronic and zero-point Energies= | -870.666708 |

Sum of electronic and thermal Energies= -870.653068
Sum of electronic and thermal Enthalpies= -870.652124
Sum of electronic and thermal Free Energies= -870.708249

Standard orientation:

| Center Number | Atomic Number | Atomic Type | Coordinates (Angstroms) X | Y | Z |
|---|---|---|---|---|---|
| 1 | 7 | 0 | 3.765506 | -1.576710 | -0.122644 |
| 2 | 7 | 0 | 3.453285 | -0.230780 | 0.129272 |
| 3 | 6 | 0 | 2.198493 | 0.247280 | -0.063394 |
| 4 | 16 | 0 | 1.876986 | 1.899053 | 0.229166 |
| 5 | 7 | 0 | 1.290994 | -0.644447 | -0.484368 |
| 6 | 6 | 0 | -0.115090 | -0.372045 | -0.744420 |
| 7 | 6 | 0 | -0.966219 | -0.546512 | 0.517974 |
| 8 | 6 | 0 | -2.438339 | -0.292149 | 0.360919 |
| 9 | 7 | 0 | -2.831603 | 0.051592 | -0.810738 |
| 10 | 6 | 0 | -3.302105 | -0.457503 | 1.581695 |
| 11 | 7 | 0 | -4.270741 | 0.291101 | -0.933655 |
| 12 | 1 | 0 | 4.132778 | -2.006350 | 0.721510 |
| 13 | 1 | 0 | 4.468971 | -1.635751 | -0.853644 |
| 14 | 1 | 0 | 4.165098 | 0.422436 | 0.427012 |
| 15 | 1 | 0 | 1.640888 | -1.589966 | -0.600581 |
| 16 | 1 | 0 | -4.394198 | 1.001353 | -1.658921 |
| 17 | 1 | 0 | -4.737895 | 0.620983 | -0.081153 |
| 18 | 1 | 0 | -0.443456 | -1.060031 | -1.523958 |
| 19 | 1 | 0 | -0.209239 | 0.642019 | -1.129432 |
| 20 | 1 | 0 | -0.853818 | -1.560942 | 0.918701 |
| 21 | 1 | 0 | -0.605483 | 0.126017 | 1.305075 |
| 22 | 1 | 0 | -4.084522 | -1.202705 | 1.415033 |
| 23 | 1 | 0 | -2.701902 | -0.780315 | 2.429810 |
| 24 | 1 | 0 | -3.778013 | 0.489100 | 1.857648 |
| 25 | 1 | 0 | -4.743519 | -0.560428 | -1.255477 |

**Data 34:** Cartesian coordinates and energies of the optimized geometry for the conformer-2 of the conjugated acid of (*E*)-**5** formed by protonation at the $NH_2$ group of the hydrazone fragment (DMSO solution).

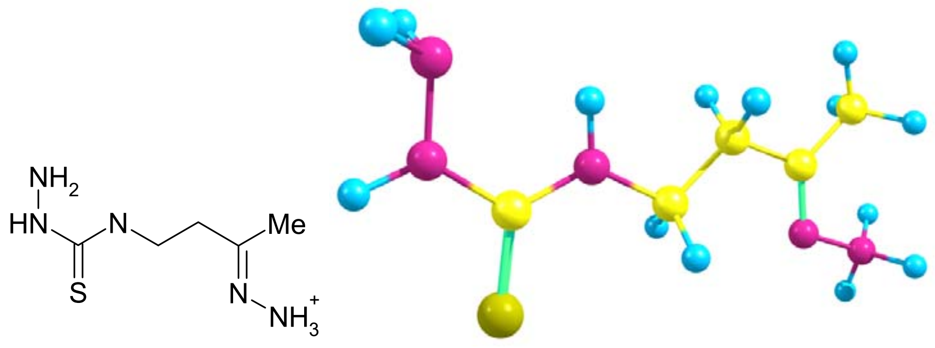


Electronic Energy = -870.881478936 a.u.
Zero-point correction= 0.216369 (Hartree/Particle)
Thermal correction to Energy= 0.230054
Thermal correction to Enthalpy= 0.230999
Thermal correction to Gibbs Free Energy= 0.174900
Sum of electronic and zero-point Energies= -870.665110
Sum of electronic and thermal Energies= -870.651425
Sum of electronic and thermal Enthalpies= -870.650480
Sum of electronic and thermal Free Energies= -870.706579

Standard orientation:

| Center Number | Atomic Number | Atomic Type | Coordinates (Angstroms) X | Y | Z |
|---|---|---|---|---|---|
| 1 | 7 | 0 | 3.411725 | -1.921985 | 0.073815 |
| 2 | 7 | 0 | 3.471117 | -0.520814 | 0.005655 |
| 3 | 6 | 0 | 2.339368 | 0.228443 | -0.006914 |
| 4 | 16 | 0 | 2.452220 | 1.929225 | -0.082564 |
| 5 | 7 | 0 | 1.186458 | -0.450349 | 0.040810 |
| 6 | 6 | 0 | -0.139895 | 0.155590 | 0.064765 |
| 7 | 6 | 0 | -1.193225 | -0.945942 | -0.015341 |
| 8 | 6 | 0 | -2.621871 | -0.479719 | -0.014706 |
| 9 | 7 | 0 | -2.806393 | 0.784926 | 0.086706 |
| 10 | 6 | 0 | -3.687771 | -1.531507 | -0.148046 |
| 11 | 7 | 0 | -4.210337 | 1.201715 | 0.081791 |
| 12 | 1 | 0 | 3.897375 | -2.246370 | 0.905207 |
| 13 | 1 | 0 | 3.857258 | -2.325610 | -0.745323 |
| 14 | 1 | 0 | 4.357941 | -0.036998 | -0.030753 |
| 15 | 1 | 0 | 1.286065 | -1.458906 | 0.103827 |
| 16 | 1 | 0 | -4.225132 | 2.181614 | -0.209143 |
| 17 | 1 | 0 | -4.817826 | 0.673549 | -0.554913 |
| 18 | 1 | 0 | -0.241334 | 0.843614 | -0.776055 |
| 19 | 1 | 0 | -0.268393 | 0.741386 | 0.978115 |
| 20 | 1 | 0 | -1.054803 | -1.548054 | -0.921749 |
| 21 | 1 | 0 | -1.089207 | -1.644643 | 0.824103 |
| 22 | 1 | 0 | -4.133015 | -1.492328 | -1.148312 |
| 23 | 1 | 0 | -3.260854 | -2.524228 | -0.019148 |
| 24 | 1 | 0 | -4.484063 | -1.403304 | 0.588467 |
| 25 | 1 | 0 | -4.607153 | 1.157034 | 1.026711 |

**Data 35:** Cartesian coordinates and energies of the optimized geometry for the conformer-1 of the conjugated acid of (*E*)-**5** formed by protonation at the $NH_2$ group of the thiosemicarbazone fragment (DMSO solution).

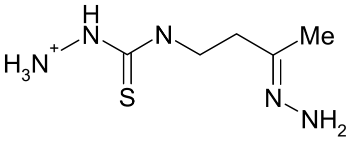


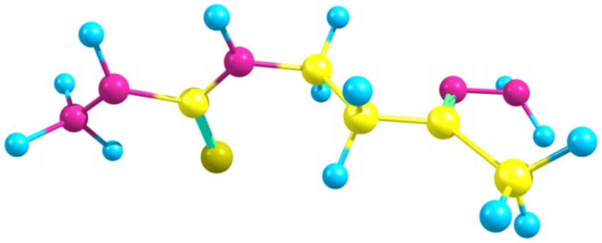

| | |
|---|---|
| Electronic Energy = | -870.873314713 a.u. |
| Zero-point correction= | 0.215227 (Hartree/Particle) |
| Thermal correction to Energy= | 0.228672 |
| Thermal correction to Enthalpy= | 0.229616 |
| Thermal correction to Gibbs Free Energy= | 0.173660 |
| Sum of electronic and zero-point Energies= | -870.658088 |
| Sum of electronic and thermal Energies= | -870.644643 |
| Sum of electronic and thermal Enthalpies= | -870.643698 |
| Sum of electronic and thermal Free Energies= | -870.699655 |

Standard orientation:

| Center Number | Atomic Number | Atomic Type | Coordinates (Angstroms) X | Y | Z |
|---|---|---|---|---|---|
| 1 | 7 | 0 | 4.393524 | -0.190167 | 0.134782 |
| 2 | 7 | 0 | 3.209320 | -0.877863 | -0.225006 |

| | | | | | |
|---|---|---|---|---|---|
| 3 | 6 | 0 | 2.033269 | -0.114791 | -0.191196 |
| 4 | 16 | 0 | 2.017643 | 1.366163 | 0.619259 |
| 5 | 7 | 0 | 1.001998 | -0.691748 | -0.796971 |
| 6 | 6 | 0 | -0.369230 | -0.168582 | -0.834889 |
| 7 | 6 | 0 | -1.237912 | -0.786517 | 0.262063 |
| 8 | 6 | 0 | -2.676663 | -0.328852 | 0.246930 |
| 9 | 7 | 0 | -3.037579 | 0.518164 | -0.643332 |
| 10 | 6 | 0 | -3.602614 | -0.915794 | 1.282378 |
| 11 | 7 | 0 | -4.376732 | 0.889948 | -0.694387 |
| 12 | 1 | 0 | 4.962675 | 0.082032 | -0.674516 |
| 13 | 1 | 0 | 4.956088 | -0.747151 | 0.783598 |
| 14 | 1 | 0 | 3.354181 | -1.533197 | -0.984839 |
| 15 | 1 | 0 | 1.141962 | -1.600611 | -1.222842 |
| 16 | 1 | 0 | -4.449666 | 1.782539 | -1.165454 |
| 17 | 1 | 0 | -4.841470 | 0.938166 | 0.210306 |
| 18 | 1 | 0 | -0.771650 | -0.396745 | -1.821450 |
| 19 | 1 | 0 | -0.320588 | 0.913245 | -0.736275 |
| 20 | 1 | 0 | -1.219619 | -1.880156 | 0.174018 |
| 21 | 1 | 0 | -0.804537 | -0.559830 | 1.243774 |
| 22 | 1 | 0 | -4.465933 | -1.391485 | 0.804750 |
| 23 | 1 | 0 | -3.091106 | -1.660799 | 1.891640 |
| 24 | 1 | 0 | -3.989507 | -0.142651 | 1.956926 |
| 25 | 1 | 0 | 4.042065 | 0.686909 | 0.614893 |

**Data 36:** Cartesian coordinates and energies of the optimized geometry for the conformer-2 of the conjugated acid of (*E*)-**5** formed by protonation at the $NH_2$ group of the thiosemicarbazone fragment (DMSO solution).

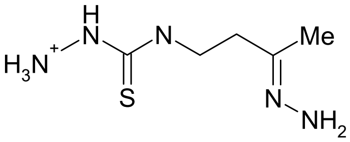


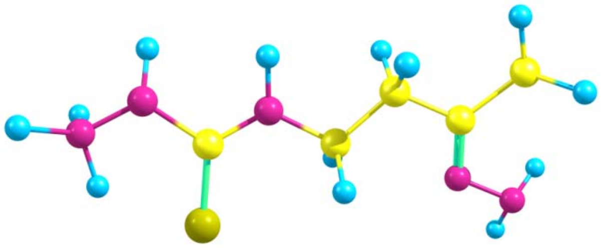

| | |
|---|---|
| Electronic Energy = | -870.872806327 a.u. |
| Zero-point correction= | 0.215063 (Hartree/Particle) |
| Thermal correction to Energy= | 0.228554 |
| Thermal correction to Enthalpy= | 0.229498 |
| Thermal correction to Gibbs Free Energy= | 0.173734 |
| Sum of electronic and zero-point Energies= | -870.657743 |
| Sum of electronic and thermal Energies= | -870.644253 |
| Sum of electronic and thermal Enthalpies= | -870.643309 |
| Sum of electronic and thermal Free Energies= | -870.699072 |

Standard orientation:

| Center Number | Atomic Number | Atomic Type | Coordinates (Angstroms) X | Y | Z |
|---|---|---|---|---|---|
| 1 | 7 | 0 | -4.469574 | -0.423949 | 0.130167 |
| 2 | 7 | 0 | -3.201090 | -1.025783 | -0.057828 |
| 3 | 6 | 0 | -2.110705 | -0.141448 | -0.028377 |
| 4 | 16 | 0 | -2.364186 | 1.523742 | -0.106912 |
| 5 | 7 | 0 | -0.927509 | -0.739625 | 0.013081 |
| 6 | 6 | 0 | 0.357331 | -0.024541 | 0.014687 |
| 7 | 6 | 0 | 1.502787 | -1.028277 | -0.042425 |
| 8 | 6 | 0 | 2.875125 | -0.398321 | -0.015398 |
| 9 | 7 | 0 | 2.958981 | 0.877252 | 0.057282 |
| 10 | 6 | 0 | 4.063454 | -1.323216 | -0.084345 |
| 11 | 7 | 0 | 4.221372 | 1.457262 | 0.024830 |

| | | | | | |
|---|---|---|---|---|---|
| 12 | 1 | 0 | -4.852929 | -0.577384 | 1.068984 |
| 13 | 1 | 0 | -5.136259 | -0.749900 | -0.574620 |
| 14 | 1 | 0 | -3.120938 | -1.919570 | 0.415027 |
| 15 | 1 | 0 | -0.894089 | -1.752516 | 0.020886 |
| 16 | 1 | 0 | 4.167968 | 2.379846 | 0.436939 |
| 17 | 1 | 0 | 4.953021 | 0.915867 | 0.481200 |
| 18 | 1 | 0 | 0.420942 | 0.588556 | 0.915172 |
| 19 | 1 | 0 | 0.387054 | 0.648423 | -0.843098 |
| 20 | 1 | 0 | 1.428458 | -1.729790 | 0.798870 |
| 21 | 1 | 0 | 1.418200 | -1.638052 | -0.951116 |
| 22 | 1 | 0 | 4.664396 | -1.269862 | 0.831403 |
| 23 | 1 | 0 | 3.750949 | -2.359082 | -0.215101 |
| 24 | 1 | 0 | 4.718292 | -1.050398 | -0.918727 |
| 25 | 1 | 0 | -4.281318 | 0.612704 | 0.007200 |

**Table S16.** Relative electronic (ΔE, kcal/mol) and Gibbs free energies (ΔG, kcal/mol) of various stereoisomers of the conjugated acid of (*E*)-**5** in DMSO solution.

| Stereoisomer of the conjugated acid of (*E*)-**5** | | ΔE | ΔG |
|---|---|---|---|
| 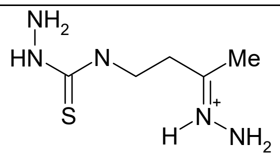 | Conformer-1 | 1.24 | 0.29 |
| | Conformer-2 | 0.00 | 0.00 |
| 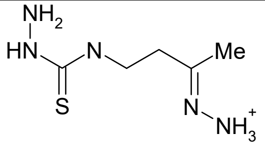 | Conformer-1 | 5.32 | 5.35 |
| | Conformer-2 | 6.24 | 6.40 |
| 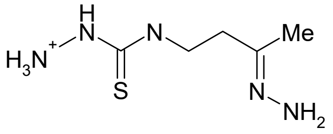 | Conformer-1 | 11.36 | 10.74 |
| | Conformer-2 | 11.68 | 11.11 |

Thus, the DFT calculations have shown that protonation at the imino nitrogen of hydrazone **5** to give the conjugated acid **C** is the most favorable compated with other possible protonation sites. Nucleophilic attack of the thiosemicarbazide $NH_2$ group in **5** on the carbon atom of the protonated imino group of **C** provides intermediate **D** which then converts to intermediate **E** (Scheme 2). Two-step elimination of the hydrazinium cation from the latter leads to the formation of a complex of dimer **A** with this cation, namely, to complex **G**. In this complex, the hydrazinium cation located in the cavity of dimer **A** is linked by at least three hydrogen bonds with donor atoms in **A** (see, data 37, 38, and 39 for two conformers of complex **G**). As a result, the possibilities of intramolecular cyclisation of **G** to form a 14-membered macrocycle **6** are blocked. Thus, at low temperatures, dimerisation of **G** takes place, followed by cyclisation of the intermediate tetramer **B** to 28-membered macrocycle **7**. An increase in the reaction temperature, for example, boiling in EtOH or MeCN, promotes the destruction of complex **G** and cyclisation of free dimer **B** into 14-membered macrocycle **6**.

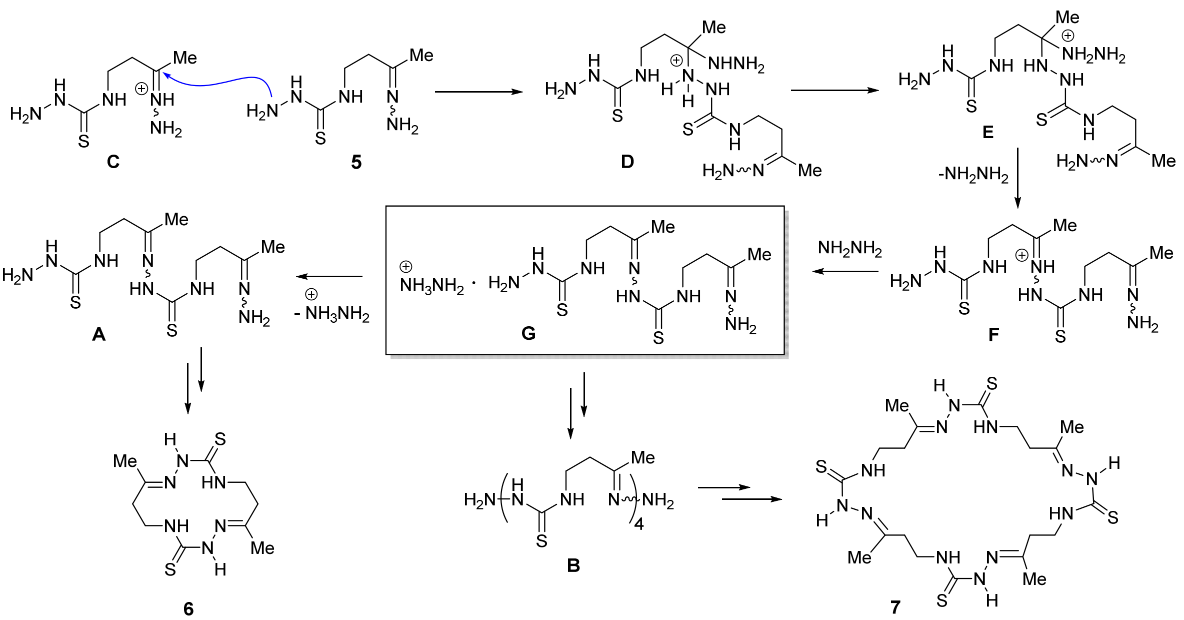

**Scheme 2.** Formation of complex **G** as one of the intermediates of the acid-catalyzed transformation of **5** into macrocycles **6** and **7**.

**Data 37:** Cartesian coordinates and energies of the optimized geometry for the 1st conformer of the complex **G** (DMSO solution).

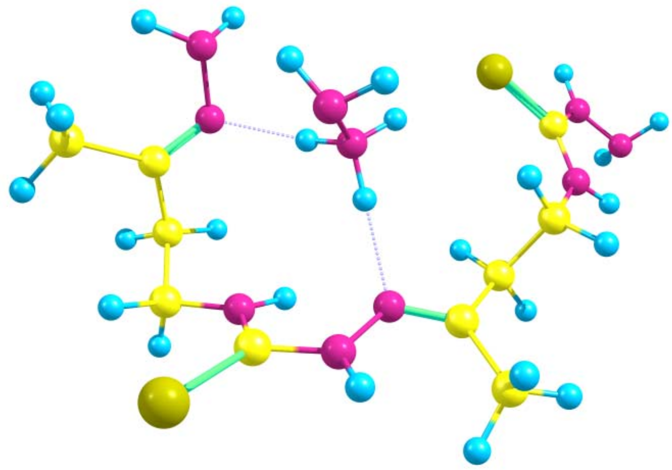

| | |
|---|---|
| Electronic Energy = | -1741.35891773 a.u. |
| Zero-point correction= | 0.420623 (Hartree/Particle) |
| Thermal correction to Energy= | 0.448345 |
| Thermal correction to Enthalpy= | 0.449289 |
| Thermal correction to Gibbs Free Energy= | 0.360553 |
| Sum of electronic and zero-point Energies= | -1740.938295 |
| Sum of electronic and thermal Energies= | -1740.910573 |
| Sum of electronic and thermal Enthalpies= | -1740.909629 |
| Sum of electronic and thermal Free Energies= | -1740.998365 |

Standard orientation:

| Center Number | Atomic Number | Atomic Type | Coordinates (Angstroms) X | Y | Z |
|---|---|---|---|---|---|
| 1 | 7 | 0 | -6.454000 | 0.428181 | 1.076095 |
| 2 | 7 | 0 | -5.471313 | 1.281886 | 0.550115 |

| | | | | | |
|---|---|---|---|---|---|
| 3 | 6 | 0 | -4.339287 | 0.784705 | -0.000707 |
| 4 | 16 | 0 | -3.218914 | 1.894131 | -0.682592 |
| 5 | 7 | 0 | -4.214008 | -0.546359 | 0.017021 |
| 6 | 6 | 0 | -3.088496 | -1.318403 | -0.482326 |
| 7 | 6 | 0 | -2.136826 | -1.747053 | 0.658472 |
| 8 | 6 | 0 | -0.903384 | -2.451071 | 0.156187 |
| 9 | 7 | 0 | 0.024296 | 1.083320 | -0.518395 |
| 10 | 7 | 0 | 0.552234 | 1.067590 | -1.867975 |
| 11 | 6 | 0 | -1.019947 | -3.829861 | -0.429138 |
| 12 | 7 | 0 | 0.203065 | -1.797270 | 0.254111 |
| 13 | 7 | 0 | 1.357801 | -2.376171 | -0.221037 |
| 14 | 6 | 0 | 2.583727 | -1.828877 | 0.051221 |
| 15 | 16 | 0 | 3.945309 | -2.418024 | -0.773700 |
| 16 | 7 | 0 | 2.590492 | -0.860785 | 0.980045 |
| 17 | 6 | 0 | 3.768359 | -0.172877 | 1.501419 |
| 18 | 6 | 0 | 3.467302 | 1.305508 | 1.804939 |
| 19 | 6 | 0 | 3.373722 | 2.205388 | 0.596416 |
| 20 | 6 | 0 | 4.625028 | 2.491516 | -0.191947 |
| 21 | 7 | 0 | 2.227542 | 2.715207 | 0.319849 |
| 22 | 7 | 0 | 2.100846 | 3.531086 | -0.808140 |
| 23 | 1 | 0 | -6.612093 | 0.652648 | 2.054159 |
| 24 | 1 | 0 | -7.324124 | 0.550137 | 0.565394 |
| 25 | 1 | 0 | -5.607416 | 2.283643 | 0.529601 |
| 26 | 1 | 0 | -4.988111 | -1.034933 | 0.458223 |
| 27 | 1 | 0 | 0.655537 | 1.677557 | 0.062804 |
| 28 | 1 | 0 | -0.956611 | 1.426373 | -0.455756 |
| 29 | 1 | 0 | 0.949980 | 1.995290 | -2.025310 |
| 30 | 1 | 0 | -0.233262 | 0.940012 | -2.500524 |
| 31 | 1 | 0 | 1.333560 | -3.112501 | -0.915647 |
| 32 | 1 | 0 | 1.727441 | -0.741907 | 1.497365 |
| 33 | 1 | 0 | 1.377472 | 4.218503 | -0.628771 |
| 34 | 1 | 0 | 2.956610 | 4.005458 | -1.088395 |
| 35 | 1 | 0 | -2.563102 | -0.708308 | -1.215526 |
| 36 | 1 | 0 | -3.485408 | -2.193988 | -0.999657 |
| 37 | 1 | 0 | -1.841889 | -0.867706 | 1.233879 |
| 38 | 1 | 0 | -2.675703 | -2.418523 | 1.335255 |
| 39 | 1 | 0 | -2.005320 | -4.253233 | -0.239786 |
| 40 | 1 | 0 | -0.267557 | -4.497941 | 0.000829 |
| 41 | 1 | 0 | -0.866139 | -3.812527 | -1.514301 |
| 42 | 1 | 0 | 4.094933 | -0.665413 | 2.422722 |
| 43 | 1 | 0 | 4.569279 | -0.281770 | 0.771908 |
| 44 | 1 | 0 | 4.281021 | 1.674535 | 2.438744 |
| 45 | 1 | 0 | 2.547442 | 1.386972 | 2.389524 |
| 46 | 1 | 0 | 4.869624 | 3.559895 | -0.162026 |
| 47 | 1 | 0 | 5.479229 | 1.948330 | 0.209755 |
| 48 | 1 | 0 | 4.502706 | 2.212467 | -1.243379 |
| 49 | 1 | 0 | 0.059715 | 0.118574 | -0.139462 |

---------------------------------------------------------------------

**Data 38:** Cartesian coordinates and energies of the optimized geometry for the 2nd conformer of the complex **G** (DMSO solution).

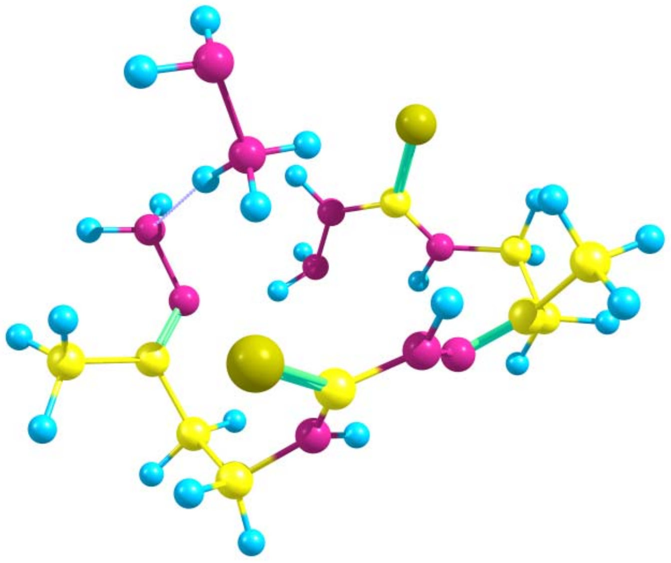

| | |
|---|---|
| Electronic Energy = | -1741.36338305 a.u. |
| Zero-point correction= | 0.420810 (Hartree/Particle) |
| Thermal correction to Energy= | 0.448384 |
| Thermal correction to Enthalpy= | 0.449328 |
| Thermal correction to Gibbs Free Energy= | 0.362311 |
| Sum of electronic and zero-point Energies= | -1740.942573 |
| Sum of electronic and thermal Energies= | -1740.914999 |
| Sum of electronic and thermal Enthalpies= | -1740.914055 |
| Sum of electronic and thermal Free Energies= | -1741.001072 |

Standard orientation:

| Center Number | Atomic Number | Atomic Type | Coordinates (Angstroms) X | Y | Z |
|---|---|---|---|---|---|
| 1 | 7 | 0 | 0.670726 | 2.391515 | -2.132363 |
| 2 | 7 | 0 | 1.073716 | 2.450827 | -0.787567 |
| 3 | 6 | 0 | 2.099453 | 1.704662 | -0.315966 |
| 4 | 16 | 0 | 2.377551 | 1.679663 | 1.380646 |
| 5 | 7 | 0 | 2.820389 | 1.047934 | -1.228602 |
| 6 | 6 | 0 | 3.950269 | 0.159205 | -0.994689 |
| 7 | 6 | 0 | 3.596575 | -1.331766 | -1.209954 |
| 8 | 6 | 0 | 2.636150 | -1.829917 | -0.166027 |
| 9 | 7 | 0 | -1.125676 | 1.362596 | 3.574789 |
| 10 | 7 | 0 | -0.897299 | 1.034802 | 2.184530 |
| 11 | 6 | 0 | 3.153569 | -2.196764 | 1.200318 |
| 12 | 7 | 0 | 1.395554 | -1.841276 | -0.501090 |
| 13 | 7 | 0 | 0.470161 | -2.150730 | 0.465871 |
| 14 | 6 | 0 | -0.862667 | -2.067253 | 0.185085 |
| 15 | 16 | 0 | -1.968508 | -2.135562 | 1.489926 |
| 16 | 7 | 0 | -1.179340 | -1.925985 | -1.102865 |
| 17 | 6 | 0 | -2.515016 | -1.837882 | -1.681290 |
| 18 | 6 | 0 | -2.857362 | -0.426676 | -2.205251 |
| 19 | 6 | 0 | -3.121345 | 0.579154 | -1.114949 |
| 20 | 6 | 0 | -4.372008 | 0.423739 | -0.285316 |
| 21 | 7 | 0 | -2.264990 | 1.521471 | -0.960005 |
| 22 | 7 | 0 | -2.462106 | 2.405787 | 0.119630 |
| 23 | 1 | 0 | -0.304372 | 2.087036 | -2.150961 |
| 24 | 1 | 0 | 0.742592 | 3.315754 | -2.549136 |
| 25 | 1 | 0 | 0.576903 | 3.027391 | -0.123780 |
| 26 | 1 | 0 | 2.485923 | 1.158948 | -2.181752 |
| 27 | 1 | 0 | -0.922832 | 2.353871 | 3.680293 |
| 28 | 1 | 0 | -2.121996 | 1.243267 | 3.744405 |
| 29 | 1 | 0 | 0.113752 | 1.165676 | 1.973190 |
| 30 | 1 | 0 | -1.466079 | 1.601348 | 1.503473 |
| 31 | 1 | 0 | 0.721980 | -2.209166 | 1.445770 |
| 32 | 1 | 0 | -0.384888 | -1.862984 | -1.732162 |
| 33 | 1 | 0 | -2.074992 | 3.308151 | -0.135477 |
| 34 | 1 | 0 | -3.441188 | 2.542051 | 0.371456 |
| 35 | 1 | 0 | 4.315909 | 0.334434 | 0.016027 |
| 36 | 1 | 0 | 4.743591 | 0.435472 | -1.692599 |
| 37 | 1 | 0 | 3.161618 | -1.460279 | -2.203319 |
| 38 | 1 | 0 | 4.527680 | -1.904008 | -1.170635 |
| 39 | 1 | 0 | 4.242381 | -2.183790 | 1.220483 |
| 40 | 1 | 0 | 2.820618 | -3.200473 | 1.484155 |
| 41 | 1 | 0 | 2.798961 | -1.493500 | 1.960951 |
| 42 | 1 | 0 | -2.558817 | -2.541364 | -2.515525 |
| 43 | 1 | 0 | -3.230086 | -2.166903 | -0.928849 |
| 44 | 1 | 0 | -3.757016 | -0.522848 | -2.822859 |
| 45 | 1 | 0 | -2.051935 | -0.067836 | -2.849373 |
| 46 | 1 | 0 | -5.005304 | 1.314830 | -0.366137 |
| 47 | 1 | 0 | -4.961638 | -0.428494 | -0.620495 |
| 48 | 1 | 0 | -4.134970 | 0.276523 | 0.771842 |
| 49 | 1 | 0 | -1.134158 | 0.033683 | 2.043817 |

**Data 39:** Cartesian coordinates and energies of the optimized geometry for the 2nd conformer of the complex **G** (EtOH solution).

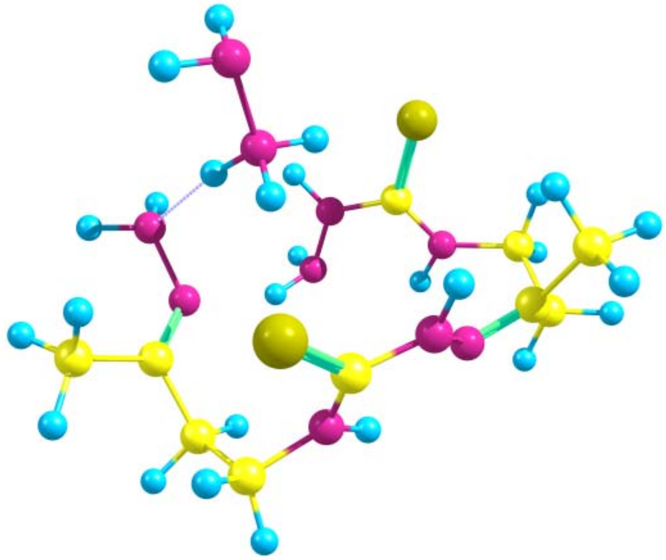

| | |
|---|---|
| Electronic Energy = | -1741.36131353 a.u. |
| Zero-point correction= | 0.421004 (Hartree/Particle) |
| Thermal correction to Energy= | 0.448453 |
| Thermal correction to Enthalpy= | 0.449397 |
| Thermal correction to Gibbs Free Energy= | 0.362997 |
| Sum of electronic and zero-point Energies= | -1740.940310 |
| Sum of electronic and thermal Energies= | -1740.912860 |
| Sum of electronic and thermal Enthalpies= | -1740.911916 |
| Sum of electronic and thermal Free Energies= | -1740.998316 |

Standard orientation:

| Center Number | Atomic Number | Atomic Type | Coordinates (Angstroms) X | Y | Z |
|---|---|---|---|---|---|
| 1 | 7 | 0 | -0.659485 | -2.395251 | -2.128810 |
| 2 | 7 | 0 | -1.056841 | -2.447148 | -0.782258 |
| 3 | 6 | 0 | -2.087826 | -1.706738 | -0.312280 |
| 4 | 16 | 0 | -2.363815 | -1.676692 | 1.384317 |
| 5 | 7 | 0 | -2.814796 | -1.059174 | -1.226530 |
| 6 | 6 | 0 | -3.948834 | -0.175372 | -0.993843 |
| 7 | 6 | 0 | -3.602621 | 1.316801 | -1.213707 |
| 8 | 6 | 0 | -2.643830 | 1.822321 | -0.171732 |
| 9 | 7 | 0 | 1.114303 | -1.343409 | 3.576409 |
| 10 | 7 | 0 | 0.895948 | -1.022909 | 2.182987 |
| 11 | 6 | 0 | -3.162694 | 2.198028 | 1.191638 |
| 12 | 7 | 0 | -1.402843 | 1.831189 | -0.505514 |
| 13 | 7 | 0 | -0.478169 | 2.144722 | 0.460730 |
| 14 | 6 | 0 | 0.854821 | 2.065045 | 0.180008 |
| 15 | 16 | 0 | 1.960886 | 2.138390 | 1.484062 |
| 16 | 7 | 0 | 1.171640 | 1.922575 | -1.107984 |
| 17 | 6 | 0 | 2.507762 | 1.837334 | -1.685683 |
| 18 | 6 | 0 | 2.856148 | 0.425485 | -2.204013 |
| 19 | 6 | 0 | 3.123326 | -0.575296 | -1.109830 |
| 20 | 6 | 0 | 4.371234 | -0.409909 | -0.278021 |
| 21 | 7 | 0 | 2.271540 | -1.521608 | -0.953912 |
| 22 | 7 | 0 | 2.470940 | -2.402525 | 0.128414 |
| 23 | 1 | 0 | 0.315382 | -2.090893 | -2.152821 |
| 24 | 1 | 0 | -0.733813 | -3.321238 | -2.541336 |
| 25 | 1 | 0 | -0.559339 | -3.022536 | -0.118049 |
| 26 | 1 | 0 | -2.481191 | -1.171729 | -2.179771 |
| 27 | 1 | 0 | 0.903508 | -2.332295 | 3.688039 |
| 28 | 1 | 0 | 2.109956 | -1.228641 | 3.752417 |
| 29 | 1 | 0 | -0.114866 | -1.153348 | 1.966784 |
| 30 | 1 | 0 | 1.467123 | -1.594150 | 1.508550 |

| | | | | | |
|---|---|---|---|---|---|
| 31 | 1 | 0 | -0.730393 | 2.209527 | 1.440124 |
| 32 | 1 | 0 | 0.377320 | 1.857928 | -1.737267 |
| 33 | 1 | 0 | 2.089405 | -3.307332 | -0.126572 |
| 34 | 1 | 0 | 3.450447 | -2.533308 | 0.381837 |
| 35 | 1 | 0 | -4.312495 | -0.349674 | 0.017743 |
| 36 | 1 | 0 | -4.741486 | -0.457686 | -1.690086 |
| 37 | 1 | 0 | -3.168698 | 1.444364 | -2.207665 |
| 38 | 1 | 0 | -4.536397 | 1.884691 | -1.175821 |
| 39 | 1 | 0 | -4.251593 | 2.190449 | 1.209350 |
| 40 | 1 | 0 | -2.825517 | 3.201396 | 1.471636 |
| 41 | 1 | 0 | -2.813522 | 1.496595 | 1.956484 |
| 42 | 1 | 0 | 2.549158 | 2.537726 | -2.522626 |
| 43 | 1 | 0 | 3.221262 | 2.172243 | -0.934327 |
| 44 | 1 | 0 | 3.756014 | 0.522728 | -2.821164 |
| 45 | 1 | 0 | 2.052935 | 0.060710 | -2.847553 |
| 46 | 1 | 0 | 5.004267 | -1.302373 | -0.343231 |
| 47 | 1 | 0 | 4.962894 | 0.437004 | -0.622959 |
| 48 | 1 | 0 | 4.129922 | -0.246226 | 0.775820 |
| 49 | 1 | 0 | 1.136000 | -0.022519 | 2.038447 |

## Thermodynamic parameters for the TsOH-promoted transformations of hydrazone 5 into macrocycles 6 and 7 (EtOH solution)

The DFT B3LYP/6-311++G(d,p) calculations were also performed to estimate thermodynamic parameters for the TsOH-promoted transformation of hydrazone **5** (EtOH solution) into dimer **13a** followed by the conversion of the latter to either macrocycle **6** or tetramer **13b** and then macrocycle **7**. Relative Gibbs free energies of the starting (**A**), final (**C** and **E**) and intermediate (**B** and **D**) molecular systems (Scheme 3) were calculated using the Gibbs free energies for the most stable conformers of hydrazone (*E*)-**5**, macrocycles **6** and **7**, dimer (*E*,*E*)-**13a**, tetramer (*E*,*E*,*E*,*E*)-**13b**, TsOH, and hydrazonium tosylate.

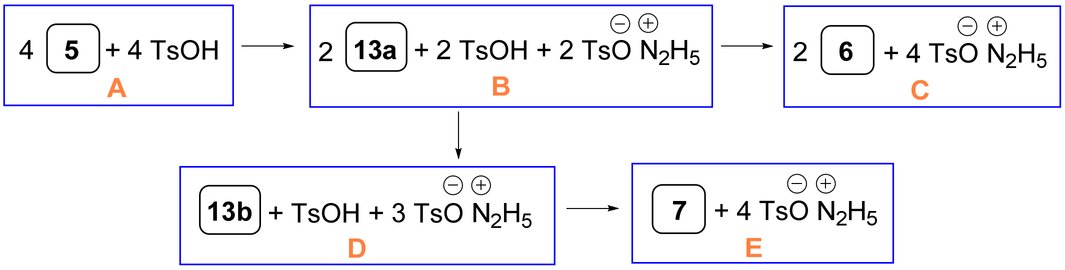


**Scheme 3.** The TsOH-promoted transformations of hydrazone **5**.

**Data 40:** Cartesian coordinates and energies of the optimized geometry for the most stable conformer of TsOH (EtOH solution).

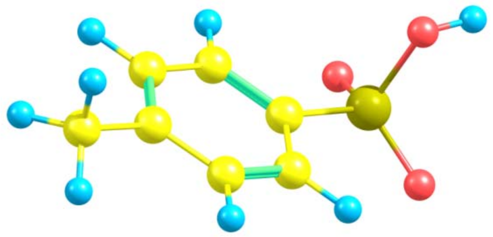

| | |
|---|---|
| Electronic Energy = | -895.543331919 a.u. |
| Zero-point correction= | 0.140676 (Hartree/Particle) |
| Thermal correction to Energy= | 0.151755 |
| Thermal correction to Enthalpy= | 0.152699 |
| Thermal correction to Gibbs Free Energy= | 0.099691 |
| Sum of electronic and zero-point Energies= | -895.402656 |
| Sum of electronic and thermal Energies= | -895.391577 |
| Sum of electronic and thermal Enthalpies= | -895.390633 |
| Sum of electronic and thermal Free Energies= | -895.443641 |

Standard orientation:

| Center Number | Atomic Number | Atomic Type | Coordinates (Angstroms) X | Y | Z |
|---|---|---|---|---|---|
| 1 | 6 | 0 | -0.560919 | 1.216835 | -0.066965 |
| 2 | 6 | 0 | 0.118438 | -0.000263 | -0.086731 |
| 3 | 6 | 0 | -0.561049 | -1.217175 | -0.066216 |
| 4 | 6 | 0 | -1.950901 | -1.204981 | -0.026657 |
| 5 | 6 | 0 | -2.666213 | -0.000045 | -0.000754 |
| 6 | 6 | 0 | -1.950679 | 1.204857 | -0.027379 |
| 7 | 6 | 0 | -4.170880 | 0.000322 | 0.073829 |
| 8 | 16 | 0 | 1.895060 | -0.000437 | -0.118431 |
| 9 | 8 | 0 | 2.398230 | -1.258580 | -0.664428 |
| 10 | 8 | 0 | 2.398681 | 1.254655 | -0.671079 |
| 11 | 8 | 0 | 2.165819 | 0.004029 | 1.502352 |
| 12 | 1 | 0 | 3.122650 | 0.007434 | 1.676963 |
| 13 | 1 | 0 | -0.016543 | 2.151962 | -0.093542 |
| 14 | 1 | 0 | -0.016760 | -2.152362 | -0.092147 |
| 15 | 1 | 0 | -2.487030 | -2.147266 | -0.018954 |
| 16 | 1 | 0 | -2.486693 | 2.147222 | -0.020226 |
| 17 | 1 | 0 | -4.592705 | 0.883300 | -0.409724 |
| 18 | 1 | 0 | -4.499935 | 0.008888 | 1.118483 |
| 19 | 1 | 0 | -4.592561 | -0.890311 | -0.395483 |

**Data 41:** Cartesian coordinates and energies of the optimized geometry for the 1st conformer of hydrazonium tosylate (EtOH solution).

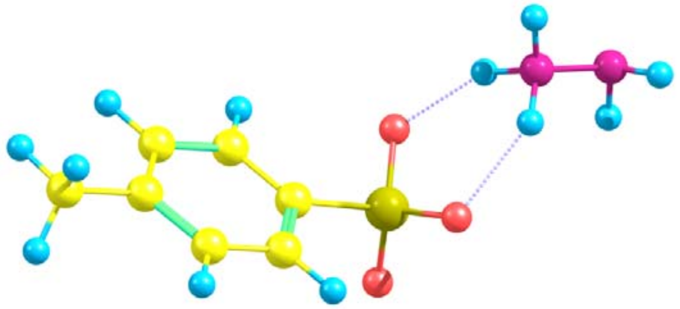

| | |
|---|---|
| Electronic Energy = | -1007.49235355 a.u. |
| Zero-point correction= | 0.199088 (Hartree/Particle) |
| Thermal correction to Energy= | 0.213953 |
| Thermal correction to Enthalpy= | 0.214897 |
| Thermal correction to Gibbs Free Energy= | 0.153086 |
| Sum of electronic and zero-point Energies= | -1007.293266 |
| Sum of electronic and thermal Energies= | -1007.278401 |
| Sum of electronic and thermal Enthalpies= | -1007.277457 |
| Sum of electronic and thermal Free Energies= | -1007.339267 |

Standard orientation:

| Center Number | Atomic Number | Atomic Type | Coordinates (Angstroms) X | Y | Z |
|---|---|---|---|---|---|
| 1 | 16 | 0 | -1.000755 | -0.770209 | 0.266494 |
| 2 | 8 | 0 | -1.610627 | -0.662741 | -1.100402 |
| 3 | 8 | 0 | -1.635170 | 0.213900 | 1.212154 |
| 4 | 8 | 0 | -0.991265 | -2.154702 | 0.793533 |
| 5 | 6 | 0 | 0.717444 | -0.256255 | 0.093269 |
| 6 | 6 | 0 | 1.442981 | -0.661163 | -1.028436 |
| 7 | 6 | 0 | 2.785192 | -0.314266 | -1.136119 |
| 8 | 6 | 0 | 3.425592 | 0.432717 | -0.136375 |
| 9 | 6 | 0 | 2.676669 | 0.824285 | 0.977769 |
| 10 | 6 | 0 | 1.329450 | 0.483491 | 1.100582 |
| 11 | 6 | 0 | 4.881255 | 0.804773 | -0.271833 |
| 12 | 7 | 0 | -3.454367 | 1.336764 | -0.491831 |
| 13 | 7 | 0 | -4.857081 | 1.068163 | -0.277563 |
| 14 | 1 | 0 | -3.339348 | 2.312204 | -0.768227 |
| 15 | 1 | 0 | -2.979076 | 0.710951 | -1.169336 |
| 16 | 1 | 0 | -2.914836 | 1.166860 | 0.386857 |
| 17 | 1 | 0 | -5.308273 | 1.090992 | -1.189348 |
| 18 | 1 | 0 | -4.919645 | 0.111699 | 0.064024 |
| 19 | 1 | 0 | 0.961416 | -1.232082 | -1.812841 |
| 20 | 1 | 0 | 3.344376 | -0.626849 | -2.011934 |
| 21 | 1 | 0 | 3.148697 | 1.405429 | 1.762692 |
| 22 | 1 | 0 | 0.759387 | 0.797921 | 1.965541 |
| 23 | 1 | 0 | 5.236177 | 1.348853 | 0.604929 |
| 24 | 1 | 0 | 5.504209 | -0.085913 | -0.394990 |
| 25 | 1 | 0 | 5.044134 | 1.435648 | -1.150939 |

**Data 42:** Cartesian coordinates and energies of the optimized geometry for the 2nd conformer of hydrazonium tosylate (EtOH solution).

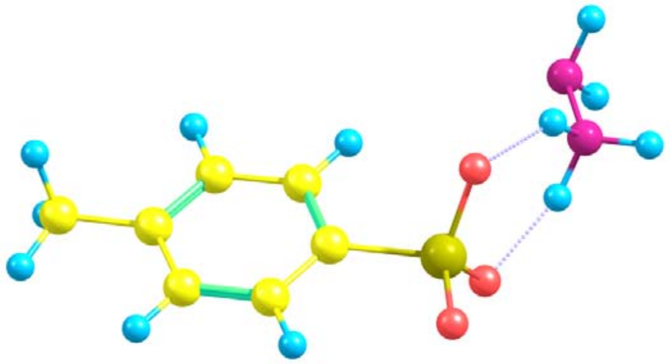

| | |
|---|---|
| Electronic Energy = | -1007.49297865 a.u. |
| Zero-point correction= | 0.199020 (Hartree/Particle) |
| Thermal correction to Energy= | 0.213785 |
| Thermal correction to Enthalpy= | 0.214729 |
| Thermal correction to Gibbs Free Energy= | 0.153355 |
| Sum of electronic and zero-point Energies= | -1007.293959 |
| Sum of electronic and thermal Energies= | -1007.279194 |
| Sum of electronic and thermal Enthalpies= | -1007.278250 |
| Sum of electronic and thermal Free Energies= | -1007.339624 |

Standard orientation:

| Center Number | Atomic Number | Atomic Type | Coordinates (Angstroms) X | Y | Z |
|---|---|---|---|---|---|
| 1 | 6 | 0 | 0.823333 | -0.972808 | 0.046781 |
| 2 | 6 | 0 | 0.648074 | 0.412260 | 0.007134 |
| 3 | 6 | 0 | 1.748232 | 1.261148 | -0.043033 |
| 4 | 6 | 0 | 3.033799 | 0.717589 | -0.051627 |

| | | | | | |
|---|---|---|---|---|---|
| 5 | 6 | 0 | 3.236541 | -0.664534 | -0.009445 |
| 6 | 6 | 0 | 2.109263 | -1.499344 | 0.037977 |
| 7 | 6 | 0 | 4.625774 | -1.252120 | -0.001571 |
| 8 | 16 | 0 | -1.018761 | 1.096188 | 0.014378 |
| 9 | 8 | 0 | -0.880182 | 2.567835 | 0.014855 |
| 10 | 8 | 0 | -1.698628 | 0.561046 | -1.218573 |
| 11 | 8 | 0 | -1.690940 | 0.554306 | 1.245755 |
| 12 | 1 | 0 | -0.033780 | -1.634591 | 0.083923 |
| 13 | 1 | 0 | 1.601098 | 2.332893 | -0.075028 |
| 14 | 1 | 0 | 3.889041 | 1.383540 | -0.092344 |
| 15 | 1 | 0 | 2.241540 | -2.575877 | 0.067975 |
| 16 | 1 | 0 | 4.855869 | -1.693935 | 0.973308 |
| 17 | 1 | 0 | 5.380520 | -0.492320 | -0.211029 |
| 18 | 1 | 0 | 4.722656 | -2.046608 | -0.746364 |
| 19 | 7 | 0 | -3.638098 | -0.940895 | -0.004165 |
| 20 | 7 | 0 | -3.588461 | -2.379419 | -0.047933 |
| 21 | 1 | 0 | -3.107925 | -0.580574 | 0.814366 |
| 22 | 1 | 0 | -4.580734 | -0.540635 | -0.005776 |
| 23 | 1 | 0 | -3.080190 | -0.530001 | -0.784524 |
| 24 | 1 | 0 | -4.118775 | -2.725090 | 0.747815 |
| 25 | 1 | 0 | -4.075339 | -2.672245 | -0.891258 |

**Data 43:** Cartesian coordinates and energies of the optimized geometry for the 3rd conformer of hydrazonium tosylate (EtOH solution).

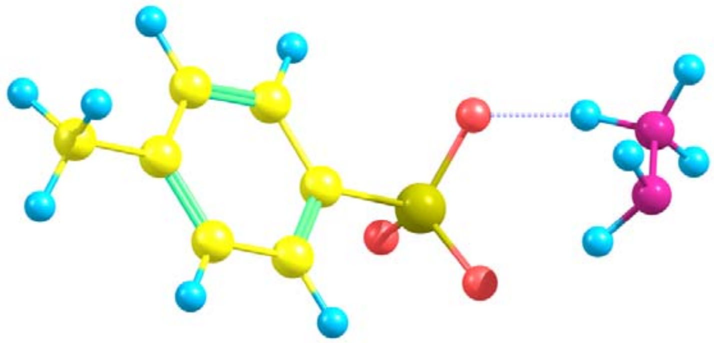

Electronic Energy = -1007.49428651 a.u.
Zero-point correction= 0.199444 (Hartree/Particle)
Thermal correction to Energy= 0.213913
Thermal correction to Enthalpy= 0.214858
Thermal correction to Gibbs Free Energy= 0.155458
Sum of electronic and zero-point Energies= -1007.294843
Sum of electronic and thermal Energies= -1007.280373
Sum of electronic and thermal Enthalpies= -1007.279429
Sum of electronic and thermal Free Energies= -1007.338829

Standard orientation:

| Center Number | Atomic Number | Atomic Type | Coordinates (Angstroms) X | Y | Z |
|---|---|---|---|---|---|
| 1 | 16 | 0 | 1.082831 | 0.894636 | -0.232139 |
| 2 | 8 | 0 | 1.699168 | 0.101775 | -1.337396 |
| 3 | 8 | 0 | 1.736630 | 0.572537 | 1.091504 |
| 4 | 8 | 0 | 1.037442 | 2.352340 | -0.492387 |
| 5 | 6 | 0 | -0.620781 | 0.327941 | -0.080551 |
| 6 | 6 | 0 | -1.248663 | -0.272830 | -1.168990 |
| 7 | 6 | 0 | -2.585041 | -0.653921 | -1.063692 |
| 8 | 6 | 0 | -3.309065 | -0.443802 | 0.115579 |
| 9 | 6 | 0 | -2.655175 | 0.167108 | 1.194147 |
| 10 | 6 | 0 | -1.321395 | 0.554626 | 1.103887 |
| 11 | 6 | 0 | -4.745869 | -0.887943 | 0.232776 |
| 12 | 1 | 0 | -0.697821 | -0.444822 | -2.084979 |
| 13 | 1 | 0 | -3.070178 | -1.121856 | -1.913814 |
| 14 | 1 | 0 | -3.196401 | 0.343211 | 2.117796 |

| | | | | | |
|---|---|---|---|---|---|
| 15 | 1 | 0 | -0.830479 | 1.021782 | 1.948815 |
| 16 | 1 | 0 | -5.304820 | -0.248833 | 0.919497 |
| 17 | 1 | 0 | -5.246561 | -0.874080 | -0.737489 |
| 18 | 1 | 0 | -4.804259 | -1.911903 | 0.617416 |
| 19 | 7 | 0 | 3.858238 | -1.056082 | 0.814942 |
| 20 | 7 | 0 | 3.472901 | -2.005439 | -0.216892 |
| 21 | 1 | 0 | 4.173339 | -1.559352 | 1.645011 |
| 22 | 1 | 0 | 3.067810 | -0.389549 | 1.060864 |
| 23 | 1 | 0 | 4.654423 | -0.515296 | 0.473828 |
| 24 | 1 | 0 | 2.877429 | -1.461745 | -0.851878 |
| 25 | 1 | 0 | 2.864261 | -2.681375 | 0.240093 |

**Data 44:** Cartesian coordinates and energies of the optimized geometry for the 1st conformer of dimer **13a** (EtOH solution).

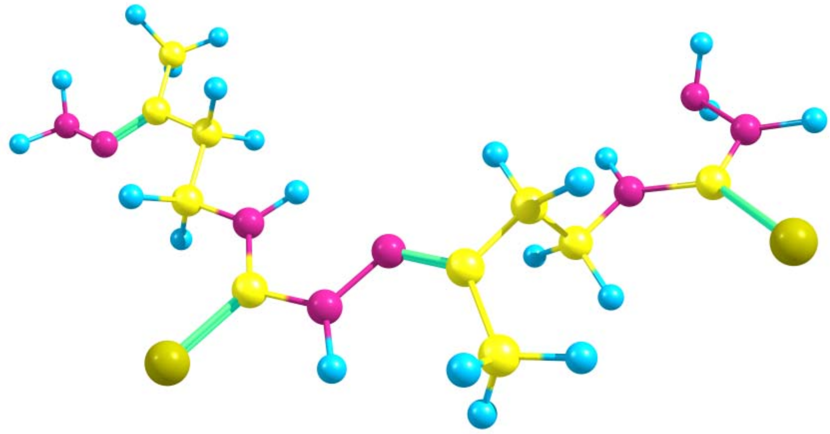

| | |
|---|---|
| Electronic Energy = | -1628.97488754 a.u. |
| Zero-point correction= | 0.348828 (Hartree/Particle) |
| Thermal correction to Energy= | 0.373541 |
| Thermal correction to Enthalpy= | 0.374485 |
| Thermal correction to Gibbs Free Energy= | 0.288607 |
| Sum of electronic and zero-point Energies= | -1628.626060 |
| Sum of electronic and thermal Energies= | -1628.601346 |
| Sum of electronic and thermal Enthalpies= | -1628.600402 |
| Sum of electronic and thermal Free Energies= | -1628.686281 |

Standard orientation:

| Center Number | Atomic Number | Atomic Type | Coordinates (Angstroms) X | Y | Z |
|---|---|---|---|---|---|
| 1 | 7 | 0 | -5.527978 | -3.354911 | -0.320135 |
| 2 | 7 | 0 | -6.110825 | -2.128505 | 0.038402 |
| 3 | 6 | 0 | -5.432768 | -0.960178 | -0.098384 |
| 4 | 16 | 0 | -6.201434 | 0.504920 | 0.324421 |
| 5 | 7 | 0 | -4.184441 | -1.062069 | -0.574271 |
| 6 | 6 | 0 | -3.244311 | 0.033397 | -0.754910 |
| 7 | 6 | 0 | -2.355831 | 0.255295 | 0.488599 |
| 8 | 6 | 0 | -1.421228 | 1.423892 | 0.321640 |
| 9 | 7 | 0 | -0.170007 | 1.151809 | 0.203795 |
| 10 | 6 | 0 | -1.992301 | 2.818608 | 0.297119 |
| 11 | 7 | 0 | 0.711316 | 2.187503 | 0.040298 |
| 12 | 6 | 0 | 2.055422 | 1.950296 | -0.066859 |
| 13 | 16 | 0 | 3.112939 | 3.263205 | -0.294495 |
| 14 | 7 | 0 | 2.431618 | 0.672217 | 0.023011 |
| 15 | 6 | 0 | 3.808829 | 0.189540 | -0.072031 |
| 16 | 6 | 0 | 3.839301 | -1.309921 | 0.206306 |
| 17 | 6 | 0 | 5.210722 | -1.937564 | 0.139076 |
| 18 | 7 | 0 | 6.211246 | -1.196187 | -0.158585 |
| 19 | 6 | 0 | 5.308448 | -3.416094 | 0.420821 |
| 20 | 7 | 0 | 7.461629 | -1.794603 | -0.276640 |

| | | | | | |
|---|---|---|---|---|---|
| 21 | 1 | 0 | -6.063275 | -3.785911 | -1.068749 |
| 22 | 1 | 0 | -5.523079 | -3.978906 | 0.481448 |
| 23 | 1 | 0 | -7.066691 | -2.081249 | 0.363906 |
| 24 | 1 | 0 | -3.874358 | -2.010760 | -0.758478 |
| 25 | 1 | 0 | 0.410700 | 3.151406 | -0.037899 |
| 26 | 1 | 0 | 1.679318 | 0.002868 | 0.143992 |
| 27 | 1 | 0 | 8.173753 | -1.085356 | -0.159478 |
| 28 | 1 | 0 | 7.630421 | -2.562716 | 0.370170 |
| 29 | 1 | 0 | -3.812739 | 0.933006 | -0.989599 |
| 30 | 1 | 0 | -2.619190 | -0.209123 | -1.616766 |
| 31 | 1 | 0 | -3.008428 | 0.438184 | 1.348833 |
| 32 | 1 | 0 | -1.772080 | -0.645776 | 0.686324 |
| 33 | 1 | 0 | -1.808760 | 3.310093 | -0.664799 |
| 34 | 1 | 0 | -3.068246 | 2.803094 | 0.465048 |
| 35 | 1 | 0 | -1.540498 | 3.439702 | 1.078227 |
| 36 | 1 | 0 | 4.208948 | 0.404676 | -1.065397 |
| 37 | 1 | 0 | 4.430617 | 0.726872 | 0.645801 |
| 38 | 1 | 0 | 3.187412 | -1.834146 | -0.505658 |
| 39 | 1 | 0 | 3.416049 | -1.510710 | 1.199266 |
| 40 | 1 | 0 | 5.795474 | -3.938220 | -0.409617 |
| 41 | 1 | 0 | 4.322365 | -3.854569 | 0.573807 |
| 42 | 1 | 0 | 5.902603 | -3.612857 | 1.321424 |

**Data 45:** Cartesian coordinates and energies of the optimized geometry for the 2nd conformer of dimer **13a** (EtOH solution).

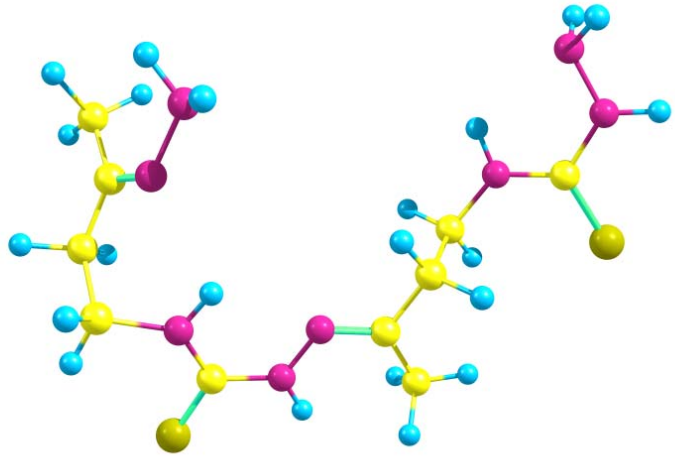

| | |
|---|---|
| Electronic Energy = | -1628.97732567 a.u. |
| Zero-point correction= | 0.349223 (Hartree/Particle) |
| Thermal correction to Energy= | 0.373664 |
| Thermal correction to Enthalpy= | 0.374608 |
| Thermal correction to Gibbs Free Energy= | 0.289444 |
| Sum of electronic and zero-point Energies= | -1628.628103 |
| Sum of electronic and thermal Energies= | -1628.603661 |
| Sum of electronic and thermal Enthalpies= | -1628.602717 |
| Sum of electronic and thermal Free Energies= | -1628.687882 |

Standard orientation:

| Center Number | Atomic Number | Atomic Type | Coordinates (Angstroms) X | Y | Z |
|---|---|---|---|---|---|
| 1 | 7 | 0 | -5.194797 | 2.731311 | -0.171736 |
| 2 | 7 | 0 | -5.673477 | 1.411051 | -0.172225 |
| 3 | 6 | 0 | -4.859740 | 0.367332 | 0.131854 |
| 4 | 16 | 0 | -5.505529 | -1.213596 | 0.156432 |
| 5 | 7 | 0 | -3.585048 | 0.679996 | 0.400078 |
| 6 | 6 | 0 | -2.515579 | -0.260389 | 0.697255 |
| 7 | 6 | 0 | -1.733000 | -0.677554 | -0.566952 |
| 8 | 6 | 0 | -0.653538 | -1.682364 | -0.262043 |

| | | | | | |
|---|---|---|---|---|---|
| 9 | 7 | 0 | 0.560516 | -1.265490 | -0.334821 |
| 10 | 6 | 0 | -1.048078 | -3.088487 | 0.111665 |
| 11 | 7 | 0 | 1.570172 | -2.146855 | -0.052625 |
| 12 | 6 | 0 | 2.882265 | -1.760583 | -0.123203 |
| 13 | 16 | 0 | 4.083700 | -2.909617 | 0.266387 |
| 14 | 7 | 0 | 3.096238 | -0.500445 | -0.501709 |
| 15 | 6 | 0 | 4.386991 | 0.162317 | -0.640246 |
| 16 | 6 | 0 | 4.577863 | 1.297784 | 0.366623 |
| 17 | 6 | 0 | 3.615905 | 2.457972 | 0.245298 |
| 18 | 7 | 0 | 2.674662 | 2.384519 | -0.621101 |
| 19 | 6 | 0 | 3.818571 | 3.631123 | 1.170195 |
| 20 | 7 | 0 | 1.756955 | 3.428871 | -0.692797 |
| 21 | 1 | 0 | -5.685563 | 3.276922 | 0.531248 |
| 22 | 1 | 0 | -5.350693 | 3.152391 | -1.082883 |
| 23 | 1 | 0 | -6.649945 | 1.210866 | -0.340168 |
| 24 | 1 | 0 | -3.362674 | 1.667383 | 0.323309 |
| 25 | 1 | 0 | 1.393531 | -3.099278 | 0.242529 |
| 26 | 1 | 0 | 2.278796 | 0.075045 | -0.679566 |
| 27 | 1 | 0 | 1.316937 | 3.411130 | -1.603967 |
| 28 | 1 | 0 | 2.152373 | 4.351264 | -0.521172 |
| 29 | 1 | 0 | -2.955246 | -1.129711 | 1.185484 |
| 30 | 1 | 0 | -1.839674 | 0.223526 | 1.405346 |
| 31 | 1 | 0 | -2.439018 | -1.112579 | -1.282228 |
| 32 | 1 | 0 | -1.281867 | 0.205122 | -1.023946 |
| 33 | 1 | 0 | -0.706574 | -3.346700 | 1.120159 |
| 34 | 1 | 0 | -2.129738 | -3.212124 | 0.082227 |
| 35 | 1 | 0 | -0.609648 | -3.814188 | -0.582229 |
| 36 | 1 | 0 | 5.162991 | -0.587989 | -0.502212 |
| 37 | 1 | 0 | 4.463004 | 0.550924 | -1.659226 |
| 38 | 1 | 0 | 5.599327 | 1.683431 | 0.257223 |
| 39 | 1 | 0 | 4.521484 | 0.898894 | 1.387784 |
| 40 | 1 | 0 | 4.087122 | 4.536713 | 0.612695 |
| 41 | 1 | 0 | 4.617320 | 3.434828 | 1.885349 |
| 42 | 1 | 0 | 2.900527 | 3.851917 | 1.724995 |

---------------------------------------------------------------------

**Data 46:** Cartesian coordinates and energies of the optimized geometry for the 3rd conformer of dimer **13a** (EtOH solution).

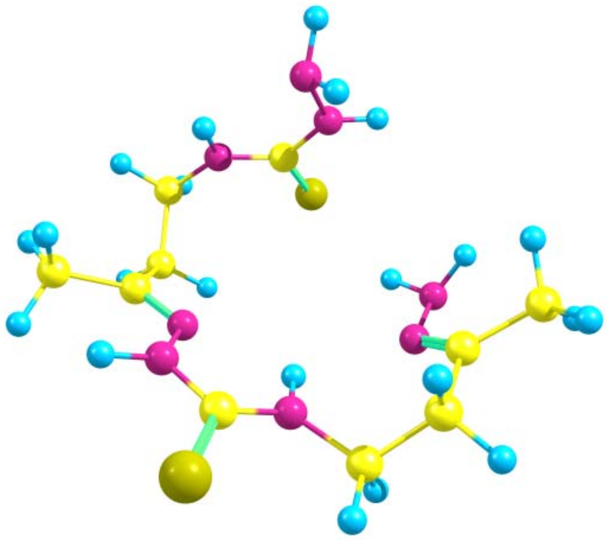

| | |
|---|---|
| Electronic Energy = | -1628.97788271 a.u. |
| Zero-point correction= | 0.349715 (Hartree/Particle) |
| Thermal correction to Energy= | 0.373746 |
| Thermal correction to Enthalpy= | 0.374690 |
| Thermal correction to Gibbs Free Energy= | 0.293022 |
| Sum of electronic and zero-point Energies= | -1628.628167 |
| Sum of electronic and thermal Energies= | -1628.604137 |
| Sum of electronic and thermal Enthalpies= | -1628.603192 |
| Sum of electronic and thermal Free Energies= | -1628.684861 |

Standard orientation:

| Center Number | Atomic Number | Atomic Type | Coordinates (Angstroms) X | Y | Z |
|---|---|---|---|---|---|
| 1 | 16 | 0 | -3.379798 | 1.470643 | -1.075689 |
| 2 | 7 | 0 | -2.250917 | 0.963562 | 2.697225 |
| 3 | 7 | 0 | -2.575160 | 1.520051 | 1.449367 |
| 4 | 1 | 0 | -2.601408 | 2.521789 | 1.316310 |
| 5 | 7 | 0 | -2.964439 | -0.581506 | 0.641370 |
| 6 | 1 | 0 | -2.676033 | -0.847206 | 1.577320 |
| 7 | 6 | 0 | -2.953872 | 0.736302 | 0.407726 |
| 8 | 6 | 0 | -3.313822 | -1.623072 | -0.315966 |
| 9 | 1 | 0 | -4.143317 | -1.270184 | -0.929342 |
| 10 | 1 | 0 | -3.667600 | -2.477858 | 0.263328 |
| 11 | 6 | 0 | -2.153003 | -2.033979 | -1.245246 |
| 12 | 1 | 0 | -2.539584 | -2.801447 | -1.926107 |
| 13 | 1 | 0 | -1.853688 | -1.173243 | -1.843785 |
| 14 | 6 | 0 | -0.946608 | -2.576341 | -0.525573 |
| 15 | 6 | 0 | -1.036184 | -3.911348 | 0.168480 |
| 16 | 1 | 0 | -0.834420 | -3.819803 | 1.241224 |
| 17 | 1 | 0 | -0.308612 | -4.618056 | -0.245983 |
| 18 | 1 | 0 | -2.025955 | -4.350423 | 0.049430 |
| 19 | 16 | 0 | 3.804955 | -2.213046 | 0.736008 |
| 20 | 7 | 0 | 0.109479 | -1.844306 | -0.555954 |
| 21 | 7 | 0 | 1.241339 | -2.290139 | 0.072972 |
| 22 | 1 | 0 | 1.294695 | -3.194962 | 0.524983 |
| 23 | 7 | 0 | 2.313884 | -0.387417 | -0.608231 |
| 24 | 1 | 0 | 1.394518 | -0.106598 | -0.934376 |
| 25 | 6 | 0 | 2.400729 | -1.561360 | 0.016371 |
| 26 | 6 | 0 | 3.388093 | 0.574699 | -0.816764 |
| 27 | 1 | 0 | 4.332429 | 0.070209 | -0.619765 |
| 28 | 1 | 0 | 3.368367 | 0.867220 | -1.869577 |
| 29 | 6 | 0 | 3.275950 | 1.818167 | 0.067260 |
| 30 | 1 | 0 | 4.162252 | 2.439091 | -0.114626 |
| 31 | 1 | 0 | 3.333771 | 1.527786 | 1.124442 |
| 32 | 6 | 0 | 2.045892 | 2.674230 | -0.135585 |
| 33 | 6 | 0 | 1.991805 | 3.989691 | 0.601666 |
| 34 | 1 | 0 | 1.908332 | 4.826741 | -0.100341 |
| 35 | 1 | 0 | 1.124962 | 4.040752 | 1.271394 |
| 36 | 1 | 0 | 2.886397 | 4.138637 | 1.206142 |
| 37 | 7 | 0 | 0.017176 | 3.065516 | -1.144554 |
| 38 | 1 | 0 | -0.217516 | 3.690177 | -0.375302 |
| 39 | 1 | 0 | -0.789093 | 2.489562 | -1.368301 |
| 40 | 7 | 0 | 1.117296 | 2.249885 | -0.910730 |
| 41 | 1 | 0 | -2.898033 | 1.300141 | 3.404854 |
| 42 | 1 | 0 | -1.307444 | 1.234650 | 2.958499 |

**Data 47:** Cartesian coordinates and energies of the optimized geometry for the 4th conformer of dimer **13a** (EtOH solution).

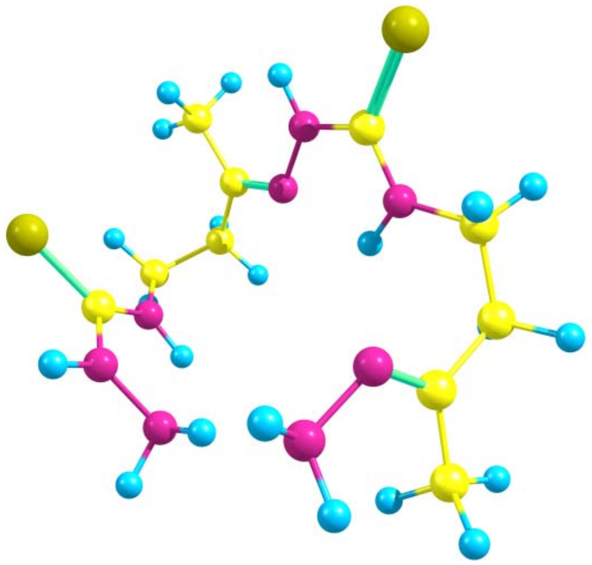

| | |
|---|---|
| Electronic Energy = | -1628.97815151 a.u. |
| Zero-point correction= | 0.349984 (Hartree/Particle) |
| Thermal correction to Energy= | 0.373742 |
| Thermal correction to Enthalpy= | 0.374686 |
| Thermal correction to Gibbs Free Energy= | 0.294337 |
| Sum of electronic and zero-point Energies= | -1628.628167 |
| Sum of electronic and thermal Energies= | -1628.604410 |
| Sum of electronic and thermal Enthalpies= | -1628.603465 |
| Sum of electronic and thermal Free Energies= | -1628.683814 |

Standard orientation:

| Center Number | Atomic Number | Atomic Type | Coordinates (Angstroms) X | Y | Z |
|---|---|---|---|---|---|
| 1 | 16 | 0 | 3.670115 | -0.612986 | -1.659717 |
| 2 | 16 | 0 | -2.212324 | 3.294092 | -1.426260 |
| 3 | 7 | 0 | 1.062869 | -3.162975 | -0.078052 |
| 4 | 7 | 0 | 1.887211 | -2.447475 | -0.964672 |
| 5 | 1 | 0 | 1.915128 | -2.674406 | -1.949240 |
| 6 | 7 | 0 | 2.617205 | -1.173789 | 0.779732 |
| 7 | 1 | 0 | 1.972402 | -1.770313 | 1.290649 |
| 8 | 7 | 0 | 0.505321 | 1.623756 | 0.879729 |
| 9 | 7 | 0 | -0.126510 | 2.429376 | -0.030615 |
| 10 | 1 | 0 | 0.343851 | 3.191124 | -0.504001 |
| 11 | 7 | 0 | -2.047220 | 1.223356 | 0.294146 |
| 12 | 1 | 0 | -1.449881 | 0.663556 | 0.892022 |
| 13 | 6 | 0 | 2.679531 | -1.435141 | -0.532588 |
| 14 | 6 | 0 | 3.343872 | -0.147257 | 1.509171 |
| 15 | 1 | 0 | 4.063114 | 0.302528 | 0.826034 |
| 16 | 1 | 0 | 3.903253 | -0.625378 | 2.318083 |
| 17 | 6 | 0 | 2.407507 | 0.915525 | 2.122077 |
| 18 | 1 | 0 | 1.634554 | 0.421672 | 2.715036 |
| 19 | 1 | 0 | 3.007976 | 1.526025 | 2.805244 |
| 20 | 6 | 0 | 1.756041 | 1.816126 | 1.105984 |
| 21 | 6 | 0 | -1.450395 | 2.242327 | -0.325081 |
| 22 | 6 | 0 | -3.439611 | 0.820774 | 0.109221 |
| 23 | 1 | 0 | -3.627078 | 0.641167 | -0.950997 |
| 24 | 1 | 0 | -4.092009 | 1.638228 | 0.426938 |
| 25 | 6 | 0 | -3.758196 | -0.425792 | 0.928419 |
| 26 | 1 | 0 | -3.473173 | -0.267871 | 1.976498 |
| 27 | 1 | 0 | -4.847645 | -0.550301 | 0.946607 |
| 28 | 6 | 0 | -3.148767 | -1.725837 | 0.446855 |
| 29 | 6 | 0 | 2.586976 | 2.877096 | 0.431660 |
| 30 | 1 | 0 | 2.548477 | 2.786096 | -0.658251 |
| 31 | 1 | 0 | 3.630527 | 2.808834 | 0.735904 |
| 32 | 1 | 0 | 2.227648 | 3.877878 | 0.697790 |
| 33 | 6 | 0 | -3.370481 | -2.948537 | 1.302164 |
| 34 | 1 | 0 | -3.962005 | -3.703704 | 0.770963 |
| 35 | 1 | 0 | -3.903556 | -2.696949 | 2.218837 |
| 36 | 1 | 0 | -2.416861 | -3.413525 | 1.572933 |
| 37 | 7 | 0 | -2.501076 | -1.726578 | -0.656309 |
| 38 | 7 | 0 | -1.915701 | -2.924543 | -1.076071 |
| 39 | 1 | 0 | 1.347645 | -4.138774 | -0.067396 |
| 40 | 1 | 0 | 0.087003 | -3.099079 | -0.385978 |
| 41 | 1 | 0 | -2.453792 | -3.758637 | -0.843539 |
| 42 | 1 | 0 | -1.793782 | -2.879474 | -2.081009 |

**Data 48:** Cartesian coordinates and energies of the optimized geometry for the 1st conformer of tetramer **13b** (EtOH solution).

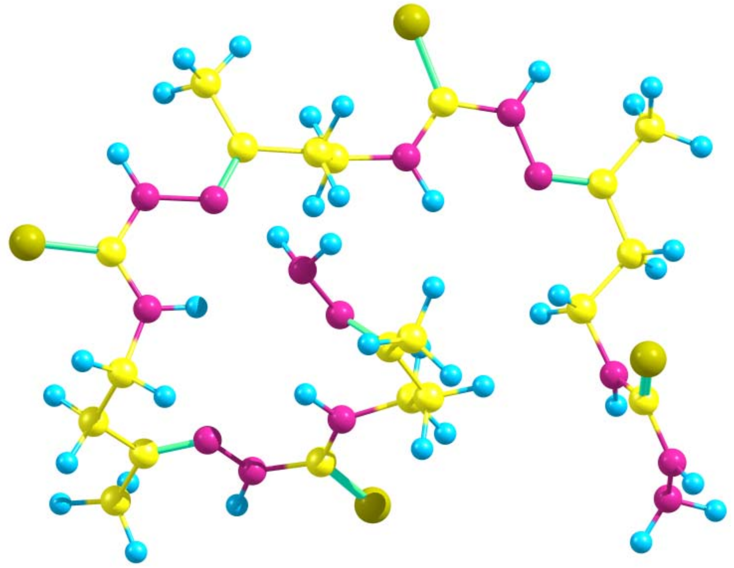

| | |
|---|---|
| Electronic Energy = | -3146.03256059 a.u. |
| Zero-point correction= | 0.644887 (Hartree/Particle) |
| Thermal correction to Energy= | 0.691044 |
| Thermal correction to Enthalpy= | 0.691988 |
| Thermal correction to Gibbs Free Energy= | 0.555216 |
| Sum of electronic and zero-point Energies= | -3145.387674 |
| Sum of electronic and thermal Energies= | -3145.341516 |
| Sum of electronic and thermal Enthalpies= | -3145.340572 |
| Sum of electronic and thermal Free Energies= | -3145.477344 |

Standard orientation:

| Center Number | Atomic Number | Atomic Type | Coordinates (Angstroms) X | Y | Z |
|---|---|---|---|---|---|
| 1 | 7 | 0 | -3.084227 | 2.043264 | 0.367642 |
| 2 | 7 | 0 | -4.445533 | 2.180868 | 0.275140 |
| 3 | 6 | 0 | -5.272421 | 1.093292 | 0.349455 |
| 4 | 16 | 0 | -6.956746 | 1.349788 | 0.351903 |
| 5 | 7 | 0 | -4.669806 | -0.097643 | 0.412345 |
| 6 | 6 | 0 | -5.343015 | -1.385570 | 0.536822 |
| 7 | 6 | 0 | -5.648307 | -2.055860 | -0.803165 |
| 8 | 6 | 0 | -4.471076 | -2.579112 | -1.586457 |
| 9 | 7 | 0 | -3.295320 | -2.470856 | -1.080190 |
| 10 | 6 | 0 | -4.767149 | -3.218812 | -2.919251 |
| 11 | 7 | 0 | -2.241030 | -2.979302 | -1.797939 |
| 12 | 6 | 0 | -0.963946 | -2.960017 | -1.309485 |
| 13 | 16 | 0 | 0.266435 | -3.678422 | -2.252955 |
| 14 | 7 | 0 | -0.794081 | -2.372792 | -0.124706 |
| 15 | 6 | 0 | 0.477772 | -2.153055 | 0.555508 |
| 16 | 6 | 0 | 0.399980 | -2.467267 | 2.048625 |
| 17 | 6 | 0 | -0.523875 | -1.595025 | 2.868549 |
| 18 | 7 | 0 | -1.296309 | -0.776327 | 2.255737 |
| 19 | 6 | 0 | -0.485775 | -1.753651 | 4.366976 |
| 20 | 7 | 0 | -2.176510 | -0.001574 | 3.003628 |
| 21 | 1 | 0 | -4.893946 | 3.086711 | 0.213297 |
| 22 | 1 | 0 | -3.658036 | -0.092286 | 0.367592 |
| 23 | 1 | 0 | -2.376192 | -3.470036 | -2.673067 |
| 24 | 1 | 0 | -1.602298 | -1.927481 | 0.297685 |
| 25 | 1 | 0 | -2.448441 | 0.799866 | 2.445223 |
| 26 | 1 | 0 | -1.809036 | 0.304923 | 3.902101 |
| 27 | 6 | 0 | -2.356777 | 3.097015 | 0.239804 |
| 28 | 6 | 0 | -2.903516 | 4.478382 | -0.011333 |
| 29 | 6 | 0 | -0.865916 | 2.913236 | 0.344909 |
| 30 | 6 | 0 | -0.166208 | 3.070174 | -1.023389 |

| | | | | | |
|---|---|---|---|---|---|
| 31 | 7 | 0 | 1.283415 | 3.005456 | -0.912306 |
| 32 | 6 | 0 | 2.104514 | 4.054327 | -0.770670 |
| 33 | 16 | 0 | 1.622007 | 5.687248 | -0.752546 |
| 34 | 7 | 0 | 3.430930 | 3.755124 | -0.645290 |
| 35 | 7 | 0 | 3.858384 | 2.451492 | -0.642497 |
| 36 | 6 | 0 | 5.118119 | 2.220700 | -0.546963 |
| 37 | 6 | 0 | 6.176179 | 3.289013 | -0.443277 |
| 38 | 6 | 0 | 5.575125 | 0.786329 | -0.536530 |
| 39 | 1 | 0 | 1.729364 | 2.095212 | -0.881330 |
| 40 | 6 | 0 | 4.457108 | -0.257005 | -0.614207 |
| 41 | 7 | 0 | 4.980043 | -1.617891 | -0.618643 |
| 42 | 6 | 0 | 5.225132 | -2.372503 | 0.459094 |
| 43 | 16 | 0 | 4.931569 | -1.896723 | 2.074027 |
| 44 | 7 | 0 | 5.743926 | -3.604105 | 0.215825 |
| 45 | 7 | 0 | 5.964172 | -4.053333 | -1.096461 |
| 46 | 1 | 0 | 4.067297 | 4.536805 | -0.546837 |
| 47 | 1 | 0 | 5.220559 | -2.049723 | -1.504990 |
| 48 | 1 | 0 | 5.896814 | -4.213427 | 1.007797 |
| 49 | 1 | 0 | 6.939542 | -4.313181 | -1.209593 |
| 50 | 1 | 0 | 5.382978 | -4.864949 | -1.287171 |
| 51 | 1 | 0 | -6.277534 | -1.224371 | 1.072587 |
| 52 | 1 | 0 | -4.704847 | -2.026615 | 1.146120 |
| 53 | 1 | 0 | -6.325777 | -2.900531 | -0.621359 |
| 54 | 1 | 0 | -6.212918 | -1.364982 | -1.440034 |
| 55 | 1 | 0 | -4.246489 | -2.700258 | -3.731519 |
| 56 | 1 | 0 | -4.450425 | -4.267416 | -2.935572 |
| 57 | 1 | 0 | -5.834657 | -3.190338 | -3.133448 |
| 58 | 1 | 0 | 1.221046 | -2.791907 | 0.082497 |
| 59 | 1 | 0 | 0.786943 | -1.112776 | 0.410351 |
| 60 | 1 | 0 | 1.412428 | -2.375526 | 2.459933 |
| 61 | 1 | 0 | 0.116736 | -3.518221 | 2.193927 |
| 62 | 1 | 0 | -1.492019 | -1.914882 | 4.767430 |
| 63 | 1 | 0 | -0.082605 | -0.855809 | 4.851483 |
| 64 | 1 | 0 | 0.143532 | -2.595079 | 4.657136 |
| 65 | 1 | 0 | -3.534120 | 4.805656 | 0.823005 |
| 66 | 1 | 0 | -2.098279 | 5.203457 | -0.119762 |
| 67 | 1 | 0 | -3.513883 | 4.508337 | -0.920179 |
| 68 | 1 | 0 | -0.648896 | 1.925288 | 0.754632 |
| 69 | 1 | 0 | -0.459818 | 3.666408 | 1.028125 |
| 70 | 1 | 0 | -0.482173 | 2.268154 | -1.693305 |
| 71 | 1 | 0 | -0.426134 | 4.022170 | -1.485160 |
| 72 | 1 | 0 | 7.169452 | 2.845659 | -0.387052 |
| 73 | 1 | 0 | 6.031978 | 3.903351 | 0.452066 |
| 74 | 1 | 0 | 6.152489 | 3.954725 | -1.312630 |
| 75 | 1 | 0 | 6.275691 | 0.645270 | -1.369429 |
| 76 | 1 | 0 | 6.162041 | 0.621299 | 0.374925 |
| 77 | 1 | 0 | 3.877052 | -0.128211 | -1.528725 |
| 78 | 1 | 0 | 3.778251 | -0.149752 | 0.230306 |

---------------------------------------------------------------------

**Data 49:** Cartesian coordinates and energies of the optimized geometry for the 2nd conformer of tetramer **13b** (EtOH solution).

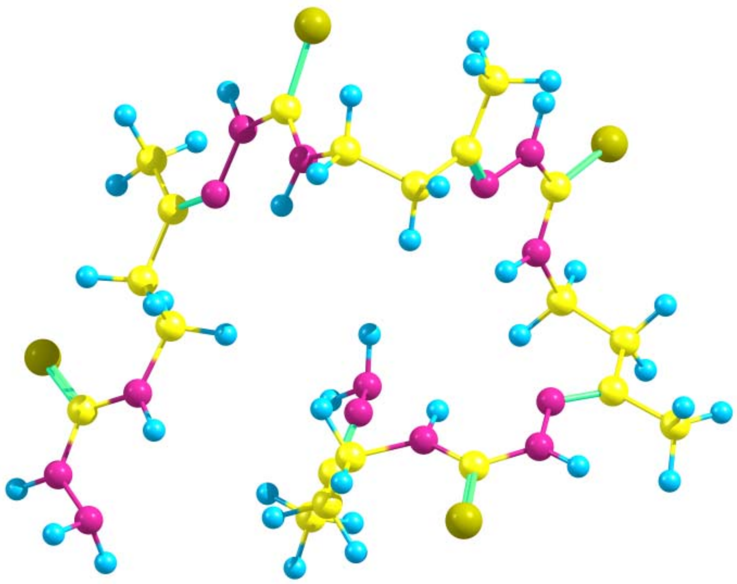

| | |
|---|---|
| Electronic Energy = | -3146.03072577 a.u. |
| Zero-point correction= | 0.645100 (Hartree/Particle) |
| Thermal correction to Energy= | 0.691137 |
| Thermal correction to Enthalpy= | 0.692081 |
| Thermal correction to Gibbs Free Energy= | 0.554600 |
| Sum of electronic and zero-point Energies= | -3145.385626 |
| Sum of electronic and thermal Energies= | -3145.339589 |
| Sum of electronic and thermal Enthalpies= | -3145.338645 |
| Sum of electronic and thermal Free Energies= | -3145.476126 |

Standard orientation:

| Center Number | Atomic Number | Atomic Type | Coordinates (Angstroms) X | Y | Z |
|---|---|---|---|---|---|
| 1 | 7 | 0 | -3.301837 | 1.561755 | -0.299719 |
| 2 | 7 | 0 | -4.026864 | 1.714082 | 0.855526 |
| 3 | 6 | 0 | -3.968579 | 0.782944 | 1.854031 |
| 4 | 16 | 0 | -4.850648 | 1.065537 | 3.285495 |
| 5 | 7 | 0 | -3.208879 | -0.289989 | 1.609583 |
| 6 | 6 | 0 | -3.090210 | -1.461679 | 2.470685 |
| 7 | 6 | 0 | -4.111744 | -2.551832 | 2.137961 |
| 8 | 6 | 0 | -3.955222 | -3.220986 | 0.794423 |
| 9 | 7 | 0 | -2.906368 | -2.950749 | 0.104877 |
| 10 | 6 | 0 | -5.041723 | -4.169569 | 0.359539 |
| 11 | 7 | 0 | -2.758505 | -3.547713 | -1.121804 |
| 12 | 6 | 0 | -1.598223 | -3.417657 | -1.833889 |
| 13 | 16 | 0 | -1.503453 | -4.156045 | -3.370831 |
| 14 | 7 | 0 | -0.631197 | -2.711410 | -1.245436 |
| 15 | 6 | 0 | 0.728534 | -2.522174 | -1.735176 |
| 16 | 6 | 0 | 1.750505 | -3.356866 | -0.959954 |
| 17 | 6 | 0 | 1.881675 | -3.037018 | 0.512186 |
| 18 | 7 | 0 | 1.132059 | -2.124227 | 1.009881 |
| 19 | 6 | 0 | 2.880278 | -3.833535 | 1.312760 |
| 20 | 7 | 0 | 1.208820 | -1.875464 | 2.376496 |
| 21 | 1 | 0 | -4.587432 | 2.537802 | 1.036603 |
| 22 | 1 | 0 | -2.788403 | -0.335803 | 0.688639 |
| 23 | 1 | 0 | -3.496698 | -4.091732 | -1.550917 |
| 24 | 1 | 0 | -0.809558 | -2.383668 | -0.301807 |
| 25 | 1 | 0 | 0.856929 | -0.944205 | 2.558610 |
| 26 | 1 | 0 | 2.140083 | -1.976377 | 2.775486 |
| 27 | 6 | 0 | -3.486086 | 2.416732 | -1.242818 |
| 28 | 6 | 0 | -4.473510 | 3.552928 | -1.173670 |
| 29 | 6 | 0 | -2.676127 | 2.231798 | -2.498326 |
| 30 | 6 | 0 | -1.658236 | 3.358380 | -2.774880 |
| 31 | 7 | 0 | -0.514964 | 3.340429 | -1.872268 |
| 32 | 6 | 0 | -0.230944 | 4.238982 | -0.921336 |
| 33 | 16 | 0 | -1.192537 | 5.576202 | -0.493238 |
| 34 | 7 | 0 | 0.945457 | 4.037105 | -0.252641 |
| 35 | 7 | 0 | 1.809057 | 3.042978 | -0.634521 |
| 36 | 6 | 0 | 2.926548 | 2.932214 | -0.008236 |
| 37 | 6 | 0 | 3.351307 | 3.819012 | 1.134010 |
| 38 | 6 | 0 | 3.859649 | 1.844784 | -0.470590 |
| 39 | 1 | 0 | 0.186369 | 2.619312 | -2.004524 |
| 40 | 6 | 0 | 3.972035 | 0.688517 | 0.546357 |
| 41 | 7 | 0 | 4.947576 | -0.309305 | 0.134597 |
| 42 | 6 | 0 | 6.239310 | -0.342894 | 0.489812 |
| 43 | 16 | 0 | 6.980404 | 0.732454 | 1.590539 |
| 44 | 7 | 0 | 6.983786 | -1.325813 | -0.078213 |
| 45 | 7 | 0 | 6.422283 | -2.226872 | -0.997806 |
| 46 | 1 | 0 | 1.188049 | 4.724554 | 0.450540 |
| 47 | 1 | 0 | 4.677069 | -1.005603 | -0.552688 |
| 48 | 1 | 0 | 7.961098 | -1.379893 | 0.174259 |
| 49 | 1 | 0 | 6.908502 | -2.157018 | -1.887290 |
| 50 | 1 | 0 | 6.513750 | -3.176401 | -0.648061 |
| 51 | 1 | 0 | -3.233333 | -1.138643 | 3.500536 |
| 52 | 1 | 0 | -2.073889 | -1.843285 | 2.368401 |
| 53 | 1 | 0 | -4.065162 | -3.329956 | 2.911257 |
| 54 | 1 | 0 | -5.123539 | -2.135630 | 2.203281 |
| 55 | 1 | 0 | -4.628858 | -5.142437 | 0.074303 |
| 56 | 1 | 0 | -5.760085 | -4.329127 | 1.162814 |
| 57 | 1 | 0 | -5.589521 | -3.773002 | -0.502784 |
| 58 | 1 | 0 | 0.747775 | -2.799080 | -2.787494 |

| | | | | | |
|---|---|---|---|---|---|
| 59 | 1 | 0 | 0.970864 | -1.460210 | -1.653465 |
| 60 | 1 | 0 | 2.732383 | -3.229627 | -1.433423 |
| 61 | 1 | 0 | 1.509433 | -4.422282 | -1.066566 |
| 62 | 1 | 0 | 3.674533 | -3.192042 | 1.712785 |
| 63 | 1 | 0 | 3.352009 | -4.602425 | 0.701010 |
| 64 | 1 | 0 | 2.394395 | -4.318010 | 2.166571 |
| 65 | 1 | 0 | -4.205384 | 4.269784 | -0.391538 |
| 66 | 1 | 0 | -4.511426 | 4.095615 | -2.117185 |
| 67 | 1 | 0 | -5.479703 | 3.176007 | -0.960392 |
| 68 | 1 | 0 | -3.361698 | 2.205545 | -3.353499 |
| 69 | 1 | 0 | -2.161210 | 1.269730 | -2.458918 |
| 70 | 1 | 0 | -1.280580 | 3.240216 | -3.793326 |
| 71 | 1 | 0 | -2.125645 | 4.339491 | -2.703429 |
| 72 | 1 | 0 | 4.322347 | 3.516475 | 1.523317 |
| 73 | 1 | 0 | 2.629932 | 3.786448 | 1.957386 |
| 74 | 1 | 0 | 3.435694 | 4.861605 | 0.807264 |
| 75 | 1 | 0 | 3.512086 | 1.456268 | -1.429744 |
| 76 | 1 | 0 | 4.857290 | 2.272787 | -0.616232 |
| 77 | 1 | 0 | 3.007119 | 0.186971 | 0.644607 |
| 78 | 1 | 0 | 4.265734 | 1.061356 | 1.526981 |

---

**Table S17.** Calculated total and relative electronic energies (E, a.u; ΔE, kcal/mol), total and relative Gibbs free energies (G, a.u.; ΔG, kcal/mol) for molecular systems **A**, **B**, **C**, **D**, and **E** in EtOH solution.

| Energy | Molecular system | | | | |
|---|---|---|---|---|---|
| | **A** | **B** | **C** | **D** | **E** |
| E, a.u. | -7063.969356 | -7064.031540 | -7064.068883 | -7064.058752 | -7064.082116 |
| ΔE, kcal/mol | 0.00 | -39.02 | -62.45 | -56.10 | -70.76 |
| G, a.u. | -7062.931888 | -7062.942294 | -7062.9618 | -7062.939857 | -7062.953597 |
| ΔG, kcal/mol | 0.00 | -6.53 | -18.77 | -5.00 | -13.62 |

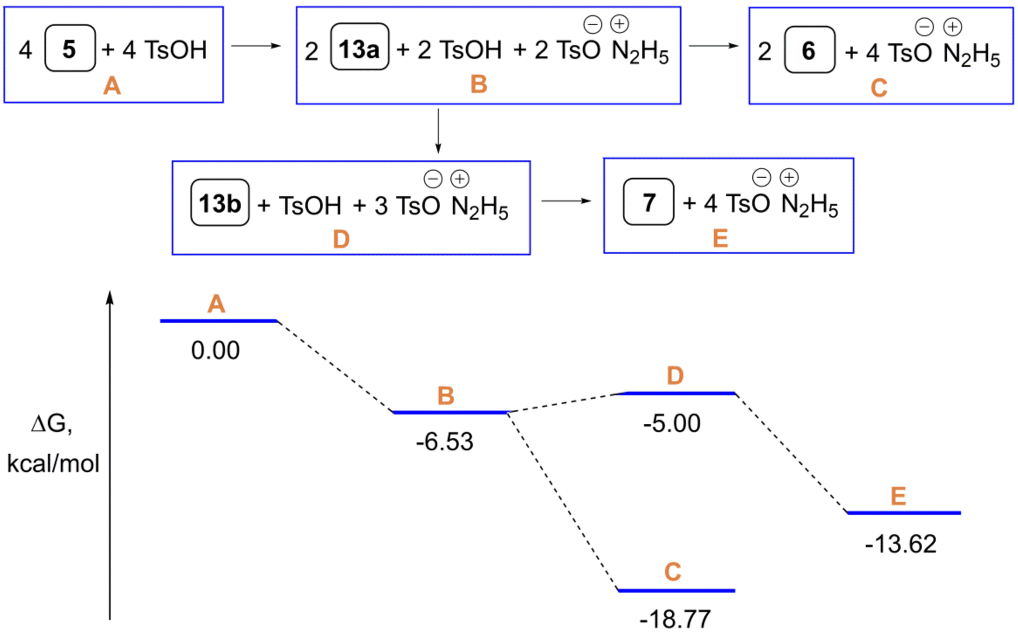


**Figure S6.** Gibbs free energy diagram (B3LYP/6-311++G(d,p)) for the TsOH-promoted transformation of hydrazone **5** into macrocycles **6** and **7** in EtOH solution. Free energies in kcal/mol at 298 K and 1 atm.

**Discussion on the acid-promoted transformation of hydrazones of 4-(1-aryl-3-oxobut-1-yl)semicarbazides 1 to give 14-membered macrocycles 3**

In contrast to the acid-promoted transformation of hydrazone **5** to give 14- and/or 28-membered macrocycles **6** and/or **7**, hydrazones of 4-(1-aryl-3-oxobut-1-yl)semicarbazides **1** under similar conditions are converted only to 14-membered macrocycles **3** (Scheme 4).[7]

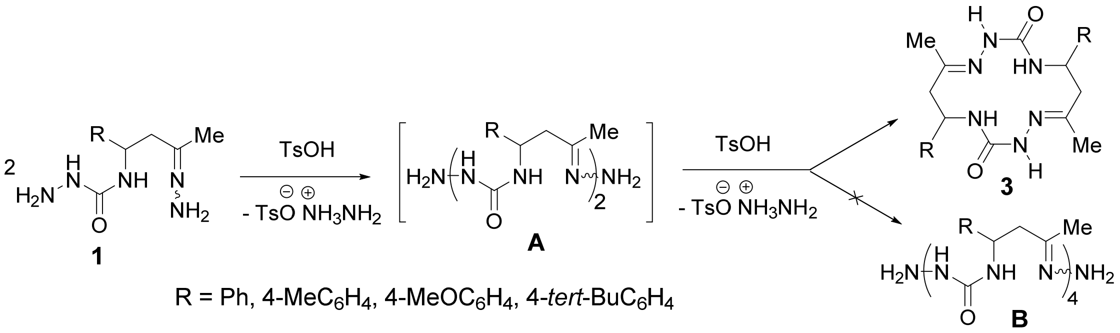


**Scheme 4.** The TsOH-promoted transformation of hydrazones of 4-(1-aryl-3-oxobut-1-yl)semicarbazides **1** to give 14-membered macrocycles **3**.

Thus, hydrazones **1** undergo dimerization to afford dimers **A** which then cyclize to macrocycles **3**. Low reactivity of dimers **A** toward to their further dimerization to give tetramers **B** can be explained mainly by the steric hindrance from the two bulky aryl groups and significant conformational rigidity of the dimers. This was confirmed by the DFT calculations (EtOH solution) for the phenyl-substituted hydrazone (*E*)-**1** (R = Ph) and its (*E,E*)-dimer.

**Data 50:** Cartesian coordinates and energies of the optimized geometry for the 1st conformer of hydrazone (*E*)-**1** (R = Ph) (EtOH solution).

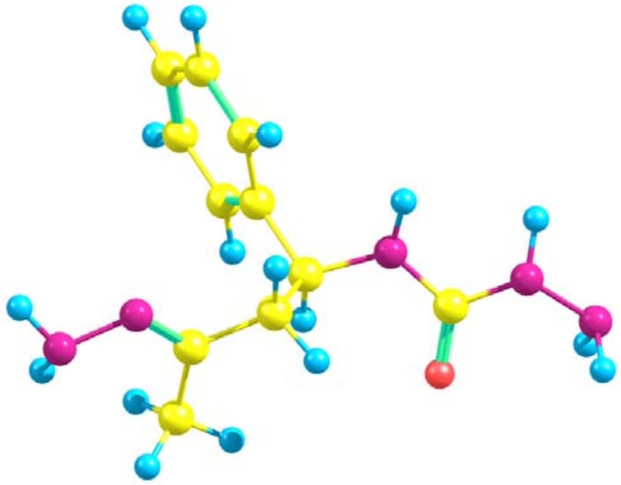

| | |
|---|---|
| Electronic Energy = | -778.594123004 a.u. |
| Zero-point correction= | 0.284156 (Hartree/Particle) |
| Thermal correction to Energy= | 0.302041 |

Thermal correction to Enthalpy= 0.302985
Thermal correction to Gibbs Free Energy= 0.236347
Sum of electronic and zero-point Energies= -778.309967
Sum of electronic and thermal Energies= -778.292082
Sum of electronic and thermal Enthalpies= -778.291138
Sum of electronic and thermal Free Energies= -778.357776

Standard orientation:

| Center Number | Atomic Number | Atomic Type | Coordinates (Angstroms) X | Y | Z |
|---|---|---|---|---|---|
| 1 | 7 | 0 | -5.242764 | 0.148373 | -0.136957 |
| 2 | 7 | 0 | -3.944308 | 0.384972 | -0.632877 |
| 3 | 6 | 0 | -2.835408 | -0.076601 | 0.060644 |
| 4 | 8 | 0 | -2.936860 | -0.879938 | 0.988203 |
| 5 | 7 | 0 | -1.642344 | 0.428851 | -0.376854 |
| 6 | 6 | 0 | -0.355137 | -0.086726 | 0.106159 |
| 7 | 6 | 0 | 0.677710 | 1.030670 | 0.109142 |
| 8 | 6 | 0 | 1.146300 | 1.545391 | 1.321874 |
| 9 | 6 | 0 | 2.079772 | 2.582217 | 1.347023 |
| 10 | 6 | 0 | 2.554858 | 3.122952 | 0.153370 |
| 11 | 6 | 0 | 2.093528 | 2.618182 | -1.063030 |
| 12 | 6 | 0 | 1.166377 | 1.577533 | -1.083868 |
| 13 | 6 | 0 | 0.077405 | -1.337430 | -0.705276 |
| 14 | 6 | 0 | 1.296903 | -2.037495 | -0.159329 |
| 15 | 7 | 0 | 2.340887 | -2.071385 | -0.904373 |
| 16 | 7 | 0 | 3.450160 | -2.772662 | -0.445978 |
| 17 | 6 | 0 | 1.222392 | -2.683154 | 1.202258 |
| 18 | 1 | 0 | -5.499996 | -0.809985 | -0.354211 |
| 19 | 1 | 0 | -5.237694 | 0.229523 | 0.877947 |
| 20 | 1 | 0 | -3.863888 | 1.258440 | -1.133425 |
| 21 | 1 | 0 | -1.633578 | 0.968335 | -1.230850 |
| 22 | 1 | 0 | 3.605334 | -2.722134 | 0.558945 |
| 23 | 1 | 0 | 4.270862 | -2.444718 | -0.938452 |
| 24 | 1 | 0 | -0.530673 | -0.389722 | 1.138308 |
| 25 | 1 | 0 | 0.777189 | 1.133512 | 2.255464 |
| 26 | 1 | 0 | 2.432406 | 2.966676 | 2.297614 |
| 27 | 1 | 0 | 3.278810 | 3.929830 | 0.169283 |
| 28 | 1 | 0 | 2.459540 | 3.031031 | -1.996469 |
| 29 | 1 | 0 | 0.831269 | 1.191067 | -2.040663 |
| 30 | 1 | 0 | 0.263589 | -1.060650 | -1.745050 |
| 31 | 1 | 0 | -0.769243 | -2.033462 | -0.691907 |
| 32 | 1 | 0 | 0.211425 | -2.646206 | 1.607832 |
| 33 | 1 | 0 | 1.888222 | -2.189933 | 1.921023 |
| 34 | 1 | 0 | 1.531691 | -3.732378 | 1.144469 |

**Data 52:** Cartesian coordinates and energies of the optimized geometry for the 2nd conformer of hydrazone (*E*)-**1** (R = Ph) (EtOH solution).

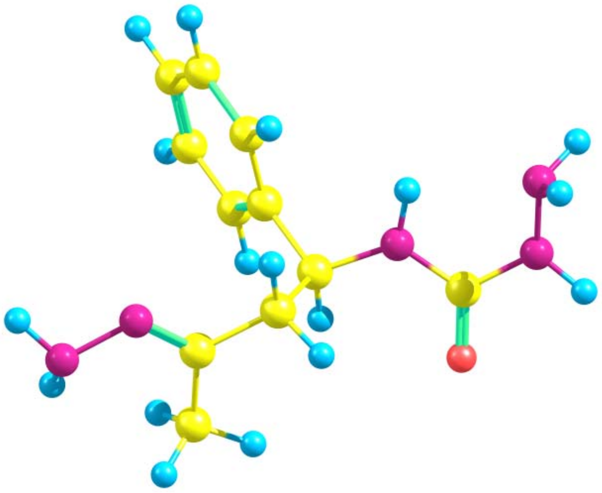

| | |
|---|---|
| Electronic Energy = | -778.597657572 a.u. |
| Zero-point correction= | 0.284205 (Hartree/Particle) |
| Thermal correction to Energy= | 0.301886 |
| Thermal correction to Enthalpy= | 0.302830 |
| Thermal correction to Gibbs Free Energy= | 0.235996 |
| Sum of electronic and zero-point Energies= | -778.313452 |
| Sum of electronic and thermal Energies= | -778.295772 |
| Sum of electronic and thermal Enthalpies= | -778.294828 |
| Sum of electronic and thermal Free Energies= | -778.361662 |

Standard orientation:

| Center Number | Atomic Number | Atomic Type | Coordinates (Angstroms) X | Y | Z |
|---|---|---|---|---|---|
| 1 | 7 | 0 | -4.199074 | 0.468556 | -1.197540 |
| 2 | 7 | 0 | -4.074923 | -0.259380 | 0.001960 |
| 3 | 6 | 0 | -2.824270 | -0.639907 | 0.459386 |
| 4 | 8 | 0 | -2.722280 | -1.440219 | 1.396392 |
| 5 | 7 | 0 | -1.774359 | -0.042442 | -0.157757 |
| 6 | 6 | 0 | -0.381964 | -0.298083 | 0.210036 |
| 7 | 6 | 0 | 0.412371 | 1.000386 | 0.196627 |
| 8 | 6 | 0 | 0.946821 | 1.506776 | 1.385063 |
| 9 | 6 | 0 | 1.666087 | 2.702864 | 1.395182 |
| 10 | 6 | 0 | 1.856791 | 3.412632 | 0.211051 |
| 11 | 6 | 0 | 1.327362 | 2.917104 | -0.981552 |
| 12 | 6 | 0 | 0.615639 | 1.718890 | -0.988422 |
| 13 | 6 | 0 | 0.225891 | -1.403961 | -0.696048 |
| 14 | 6 | 0 | 1.598473 | -1.865783 | -0.275268 |
| 15 | 7 | 0 | 2.569120 | -1.649387 | -1.086023 |
| 16 | 7 | 0 | 3.828018 | -2.130467 | -0.744151 |
| 17 | 6 | 0 | 1.756610 | -2.579416 | 1.044744 |
| 18 | 1 | 0 | -4.549730 | -0.125624 | -1.944433 |
| 19 | 1 | 0 | -4.849309 | 1.234886 | -1.059129 |
| 20 | 1 | 0 | -4.828182 | -0.874290 | 0.283963 |
| 21 | 1 | 0 | -1.997840 | 0.555455 | -0.942468 |
| 22 | 1 | 0 | 4.045075 | -2.097335 | 0.249944 |
| 23 | 1 | 0 | 4.526619 | -1.617848 | -1.266430 |
| 24 | 1 | 0 | -0.410382 | -0.670182 | 1.234628 |
| 25 | 1 | 0 | 0.797822 | 0.963022 | 2.312211 |
| 26 | 1 | 0 | 2.073301 | 3.078589 | 2.327315 |
| 27 | 1 | 0 | 2.413367 | 4.343028 | 0.215346 |
| 28 | 1 | 0 | 1.472501 | 3.461765 | -1.907873 |
| 29 | 1 | 0 | 0.221890 | 1.346275 | -1.928443 |
| 30 | 1 | 0 | 0.269876 | -1.052442 | -1.728978 |
| 31 | 1 | 0 | -0.463776 | -2.255460 | -0.662864 |
| 32 | 1 | 0 | 0.791123 | -2.770351 | 1.513023 |
| 33 | 1 | 0 | 2.359968 | -1.996108 | 1.751111 |
| 34 | 1 | 0 | 2.265569 | -3.538790 | 0.901738 |

**Data 53:** Cartesian coordinates and energies of the optimized geometry for the 3rd conformer of hydrazone (*E*)-**1** (R = Ph) (EtOH solution).

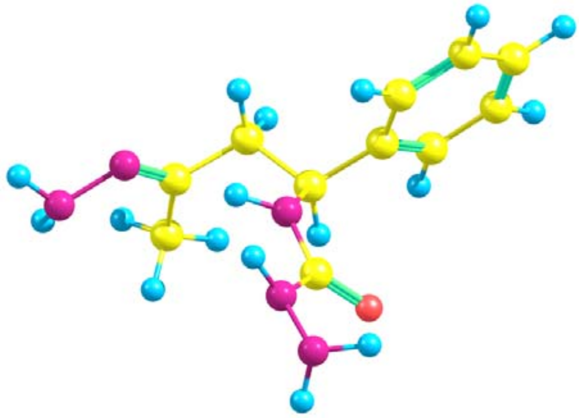

| | |
|---|---|
| Electronic Energy = | -778.596235155 a.u. |
| Zero-point correction= | 0.283730 (Hartree/Particle) |
| Thermal correction to Energy= | 0.301761 |
| Thermal correction to Enthalpy= | 0.302706 |
| Thermal correction to Gibbs Free Energy= | 0.235300 |
| Sum of electronic and zero-point Energies= | -778.312505 |
| Sum of electronic and thermal Energies= | -778.294474 |
| Sum of electronic and thermal Enthalpies= | -778.293530 |
| Sum of electronic and thermal Free Energies= | -778.360935 |

Standard orientation:

| Center Number | Atomic Number | Atomic Type | Coordinates (Angstroms) X | Y | Z |
|---|---|---|---|---|---|
| 1 | 7 | 0 | -1.155436 | 4.281266 | 0.032519 |
| 2 | 7 | 0 | -1.271295 | 2.925231 | -0.336554 |
| 3 | 6 | 0 | -0.485441 | 1.963333 | 0.281342 |
| 4 | 8 | 0 | 0.174330 | 2.208780 | 1.290318 |
| 5 | 7 | 0 | -0.522477 | 0.735626 | -0.321244 |
| 6 | 6 | 0 | 0.098465 | -0.437673 | 0.291812 |
| 7 | 6 | 0 | 1.603259 | -0.508668 | 0.058958 |
| 8 | 6 | 0 | 2.429718 | -1.048191 | 1.049469 |
| 9 | 6 | 0 | 3.802379 | -1.179728 | 0.840354 |
| 10 | 6 | 0 | 4.367812 | -0.768989 | -0.366854 |
| 11 | 6 | 0 | 3.550793 | -0.226140 | -1.359116 |
| 12 | 6 | 0 | 2.178130 | -0.098289 | -1.147408 |
| 13 | 6 | 0 | -0.592526 | -1.706689 | -0.274568 |
| 14 | 6 | 0 | -2.074919 | -1.754220 | 0.001579 |
| 15 | 7 | 0 | -2.854543 | -1.469114 | -0.978057 |
| 16 | 7 | 0 | -4.219880 | -1.415711 | -0.742095 |
| 17 | 6 | 0 | -2.556299 | -2.084640 | 1.391252 |
| 18 | 1 | 0 | -1.673166 | 4.424980 | 0.894621 |
| 19 | 1 | 0 | -0.180355 | 4.492638 | 0.236057 |
| 20 | 1 | 0 | -1.492721 | 2.797227 | -1.313612 |
| 21 | 1 | 0 | -1.266373 | 0.557104 | -0.983073 |
| 22 | 1 | 0 | -4.563265 | -2.064137 | -0.036420 |
| 23 | 1 | 0 | -4.713493 | -1.553233 | -1.614456 |
| 24 | 1 | 0 | -0.068639 | -0.393628 | 1.372259 |
| 25 | 1 | 0 | 1.998238 | -1.361351 | 1.995358 |
| 26 | 1 | 0 | 4.429256 | -1.595153 | 1.621669 |
| 27 | 1 | 0 | 5.435286 | -0.864951 | -0.530558 |
| 28 | 1 | 0 | 3.982700 | 0.100638 | -2.298776 |
| 29 | 1 | 0 | 1.551123 | 0.333859 | -1.919220 |
| 30 | 1 | 0 | -0.100967 | -2.577462 | 0.167343 |
| 31 | 1 | 0 | -0.426852 | -1.745042 | -1.354306 |
| 32 | 1 | 0 | -1.721785 | -2.246029 | 2.073065 |
| 33 | 1 | 0 | -3.164564 | -2.997449 | 1.391871 |
| 34 | 1 | 0 | -3.181880 | -1.279974 | 1.792736 |

**Data 54:** Cartesian coordinates and energies of the optimized geometry for the 4th conformer of hydrazone (*E*)-**1** (R = Ph) (EtOH solution).

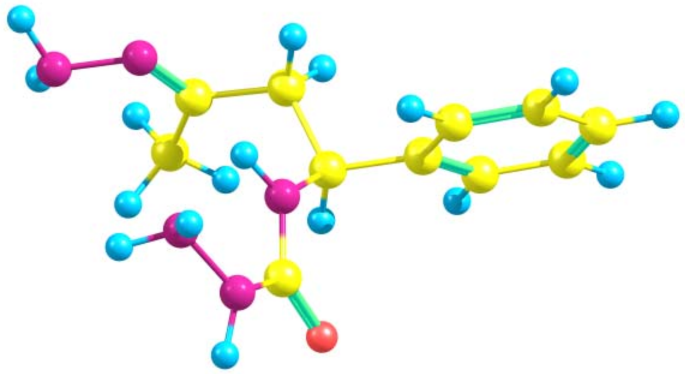

| | |
|---|---|
| Electronic Energy = | -778.599373082 a.u. |
| Zero-point correction= | 0.284091 (Hartree/Particle) |
| Thermal correction to Energy= | 0.301770 |
| Thermal correction to Enthalpy= | 0.302714 |
| Thermal correction to Gibbs Free Energy= | 0.236085 |
| Sum of electronic and zero-point Energies= | -778.315282 |
| Sum of electronic and thermal Energies= | -778.297603 |
| Sum of electronic and thermal Enthalpies= | -778.296659 |
| Sum of electronic and thermal Free Energies= | -778.363288 |

Standard orientation:

| Center Number | Atomic Number | Atomic Type | Coordinates (Angstroms) X | Y | Z |
|---|---|---|---|---|---|
| 1 | 7 | 0 | -1.928906 | 2.976425 | -0.855852 |
| 2 | 7 | 0 | -1.043500 | 3.101816 | 0.232617 |
| 3 | 6 | 0 | -0.401983 | 1.984407 | 0.739830 |
| 4 | 8 | 0 | 0.208476 | 2.049252 | 1.812353 |
| 5 | 7 | 0 | -0.470843 | 0.871528 | -0.032906 |
| 6 | 6 | 0 | 0.169952 | -0.383348 | 0.335072 |
| 7 | 6 | 0 | 1.666663 | -0.412248 | 0.036502 |
| 8 | 6 | 0 | 2.525964 | -1.125263 | 0.878505 |
| 9 | 6 | 0 | 3.890427 | -1.208752 | 0.602074 |
| 10 | 6 | 0 | 4.415479 | -0.575535 | -0.524551 |
| 11 | 6 | 0 | 3.566127 | 0.140234 | -1.368657 |
| 12 | 6 | 0 | 2.201754 | 0.220346 | -1.089443 |
| 13 | 6 | 0 | -0.546813 | -1.546887 | -0.396792 |
| 14 | 6 | 0 | -2.015635 | -1.659993 | -0.072520 |
| 15 | 7 | 0 | -2.843180 | -1.378018 | -1.011833 |
| 16 | 7 | 0 | -4.200890 | -1.406810 | -0.724904 |
| 17 | 6 | 0 | -2.431339 | -2.079223 | 1.315134 |
| 18 | 1 | 0 | -2.894622 | 3.044496 | -0.544870 |
| 19 | 1 | 0 | -1.748490 | 3.719977 | -1.521921 |
| 20 | 1 | 0 | -1.200172 | 3.836680 | 0.911471 |
| 21 | 1 | 0 | -1.060003 | 0.926267 | -0.853394 |
| 22 | 1 | 0 | -4.484009 | -2.102589 | -0.037474 |
| 23 | 1 | 0 | -4.718078 | -1.535655 | -1.584892 |
| 24 | 1 | 0 | 0.048357 | -0.513409 | 1.413578 |
| 25 | 1 | 0 | 2.127546 | -1.612956 | 1.762957 |
| 26 | 1 | 0 | 4.542816 | -1.760463 | 1.269957 |
| 27 | 1 | 0 | 5.476632 | -0.634022 | -0.739110 |
| 28 | 1 | 0 | 3.965937 | 0.639865 | -2.244318 |
| 29 | 1 | 0 | 1.551796 | 0.788691 | -1.745497 |
| 30 | 1 | 0 | -0.034957 | -2.473814 | -0.119942 |
| 31 | 1 | 0 | -0.429190 | -1.416536 | -1.475778 |
| 32 | 1 | 0 | -1.567232 | -2.267334 | 1.951723 |
| 33 | 1 | 0 | -3.026519 | -3.000132 | 1.287551 |
| 34 | 1 | 0 | -3.049964 | -1.310089 | 1.790362 |

**Table S18.** Relative electronic (ΔE, kcal/mol) and Gibbs free energies (ΔG, kcal/mol) of various conformers of (*E*)-**1** (R = Ph) in EtOH solution.

| Conformer | (*E*)-**1** | |
|---|---|---|
| | ΔE | ΔG |
| **1st** | 3.29 | 3.46 |
| **2nd** | 1.08 | 1.02 |
| **3rd** | 1.97 | 1.48 |
| **4th** | 0.00 | 0.00 |

**Data 55:** Cartesian coordinates and energies of the optimized geometry for (*E,E*)-isomer of dimer **A** (R = Ph) (EtOH solution).

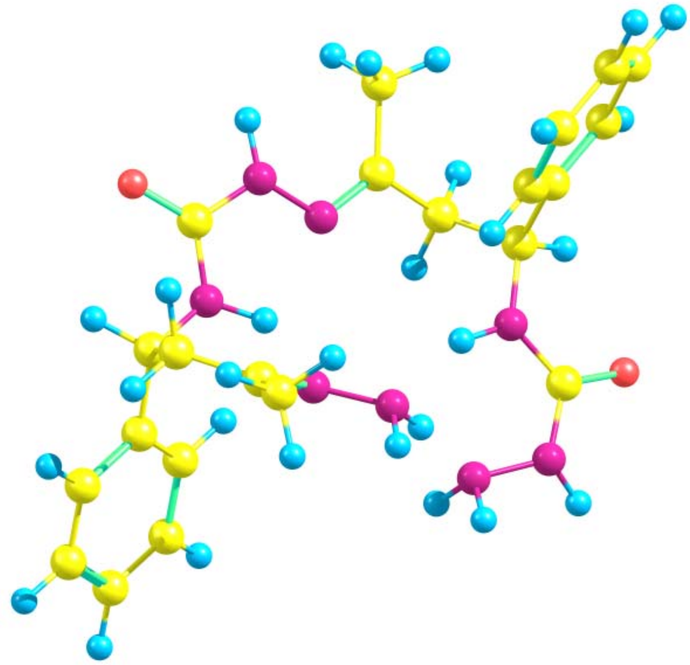

| | |
|---|---|
| Electronic Energy = | -1445.26509035 a.u. |
| Zero-point correction= | 0.515642 (Hartree/Particle) |
| Thermal correction to Energy= | 0.547967 |
| Thermal correction to Enthalpy= | 0.548911 |
| Thermal correction to Gibbs Free Energy= | 0.446472 |
| Sum of electronic and zero-point Energies= | -1444.749448 |
| Sum of electronic and thermal Energies= | -1444.717124 |
| Sum of electronic and thermal Enthalpies= | -1444.716179 |
| Sum of electronic and thermal Free Energies= | -1444.818619 |

Standard orientation:

| Center Number | Atomic Number | Atomic Type | Coordinates (Angstroms) X | Y | Z |
|---|---|---|---|---|---|
| 1 | 7 | 0 | -0.164604 | 3.395962 | 0.448122 |
| 2 | 7 | 0 | -1.466303 | 3.903674 | 0.243093 |
| 3 | 6 | 0 | -2.459161 | 3.116332 | -0.314441 |
| 4 | 7 | 0 | -2.211858 | 1.780489 | -0.386378 |
| 5 | 6 | 0 | -3.170479 | 0.874385 | -1.014024 |
| 6 | 6 | 0 | -2.495234 | 0.027699 | -2.140211 |
| 7 | 6 | 0 | -1.849856 | -1.286735 | -1.755656 |
| 8 | 7 | 0 | -0.597408 | -1.274402 | -1.465864 |
| 9 | 6 | 0 | -2.681013 | -2.543989 | -1.816473 |
| 10 | 7 | 0 | 0.009839 | -2.485680 | -1.225036 |
| 11 | 6 | 0 | 1.373469 | -2.576177 | -0.965925 |
| 12 | 7 | 0 | 1.972076 | -1.419222 | -0.591666 |
| 13 | 6 | 0 | 3.312073 | -1.405686 | -0.015715 |
| 14 | 6 | 0 | 3.278672 | -1.575191 | 1.523018 |
| 15 | 6 | 0 | 2.453982 | -0.597292 | 2.330889 |
| 16 | 7 | 0 | 1.635361 | 0.183175 | 1.729726 |
| 17 | 6 | 0 | 2.604997 | -0.650553 | 3.831117 |
| 18 | 7 | 0 | 0.810387 | 0.999941 | 2.492066 |
| 19 | 1 | 0 | 0.220246 | 3.854866 | 1.267939 |
| 20 | 1 | 0 | 0.437460 | 3.622201 | -0.342181 |
| 21 | 1 | 0 | -1.562051 | 4.884618 | 0.008122 |
| 22 | 1 | 0 | -1.293003 | 1.460918 | -0.113379 |
| 23 | 1 | 0 | -0.395995 | -3.340819 | -1.587954 |
| 24 | 1 | 0 | 1.362158 | -0.672159 | -0.280654 |
| 25 | 1 | 0 | 0.459778 | 1.744403 | 1.899185 |
| 26 | 1 | 0 | 1.243767 | 1.379896 | 3.330989 |

| | | | | | |
|---|---|---|---|---|---|
| 27 | 6 | 0 | -3.961962 | 0.042101 | -0.010676 |
| 28 | 6 | 0 | -5.300087 | -0.264615 | -0.282489 |
| 29 | 6 | 0 | -6.047038 | -1.049896 | 0.594892 |
| 30 | 6 | 0 | -5.463820 | -1.539009 | 1.763751 |
| 31 | 6 | 0 | -4.132959 | -1.232902 | 2.047536 |
| 32 | 6 | 0 | -3.388344 | -0.447751 | 1.167042 |
| 33 | 6 | 0 | 4.125047 | -0.194773 | -0.476717 |
| 34 | 6 | 0 | 5.472963 | -0.097516 | -0.102627 |
| 35 | 6 | 0 | 6.259776 | 0.974716 | -0.517524 |
| 36 | 6 | 0 | 5.713940 | 1.976171 | -1.321697 |
| 37 | 6 | 0 | 4.379319 | 1.884623 | -1.710054 |
| 38 | 6 | 0 | 3.594061 | 0.807609 | -1.293433 |
| 39 | 8 | 0 | 1.937504 | -3.669965 | -1.069507 |
| 40 | 8 | 0 | -3.511043 | 3.645845 | -0.689179 |
| 41 | 1 | 0 | -3.879833 | 1.530981 | -1.517339 |
| 42 | 1 | 0 | -1.756250 | 0.666117 | -2.627856 |
| 43 | 1 | 0 | -3.267148 | -0.194283 | -2.881873 |
| 44 | 1 | 0 | -3.741765 | -2.308498 | -1.878176 |
| 45 | 1 | 0 | -2.518848 | -3.172972 | -0.936269 |
| 46 | 1 | 0 | -2.422657 | -3.138196 | -2.702323 |
| 47 | 1 | 0 | 3.810893 | -2.295928 | -0.404984 |
| 48 | 1 | 0 | 4.305077 | -1.559369 | 1.902443 |
| 49 | 1 | 0 | 2.908603 | -2.585361 | 1.744529 |
| 50 | 1 | 0 | 1.630021 | -0.773145 | 4.314656 |
| 51 | 1 | 0 | 3.046179 | 0.275503 | 4.219887 |
| 52 | 1 | 0 | 3.249824 | -1.475505 | 4.134235 |
| 53 | 1 | 0 | -5.764906 | 0.119544 | -1.185327 |
| 54 | 1 | 0 | -7.084365 | -1.271658 | 0.369736 |
| 55 | 1 | 0 | -6.042818 | -2.146291 | 2.450361 |
| 56 | 1 | 0 | -3.672855 | -1.602336 | 2.957400 |
| 57 | 1 | 0 | -2.359179 | -0.209639 | 1.409830 |
| 58 | 1 | 0 | 5.922165 | -0.872117 | 0.509866 |
| 59 | 1 | 0 | 7.300716 | 1.024127 | -0.217372 |
| 60 | 1 | 0 | 6.324082 | 2.811575 | -1.646130 |
| 61 | 1 | 0 | 3.943419 | 2.650664 | -2.342166 |
| 62 | 1 | 0 | 2.563097 | 0.748957 | -1.617649 |

For comparison, the structures of the most stable conformations of hydrazone (*E*)-**1** (R = Ph) (see Data 54) and hydrazone (*E*)-**5** (see Data 13) are shown in Figure S7.

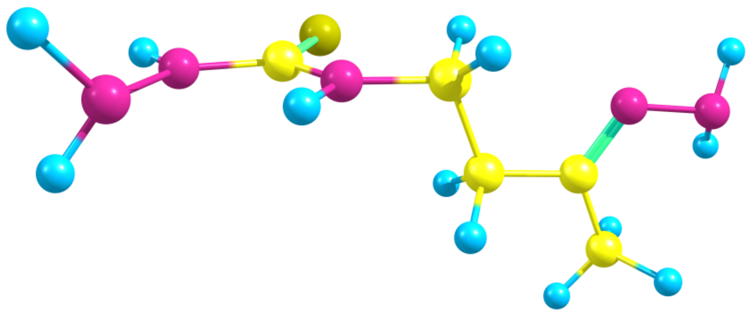

(***a***)

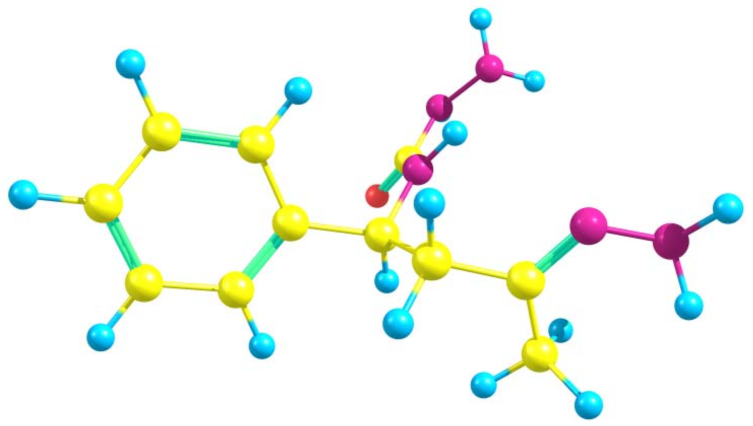

(***b***)

**Figure S7.** The most stable conformations of hydrazone (*E*)-**1** (R = Ph) (***a***) and hydrazone (*E*)-**5** (***b***)

The Figure shows that the most stable conformations of these compounds differ dramatically, apparently due to the influence of the bulky phenyl group in (*E*)-**1** (R = Ph). For example, the dihedral angles N=C-$CH_2$-C are 1.633° and 111.224° in (*E*)-**5** and (*E*)-**1** (R = Ph), respectively.

For comparison, the structures of the most stable conformations of dimer (*E,E*)-**13a** (see Data 45) and (*E,E*)-isomer of dimer **A** (R = Ph) (see Data 55) are shown in Figure S8. Obviously, the conformations of these compounds are extremely different.

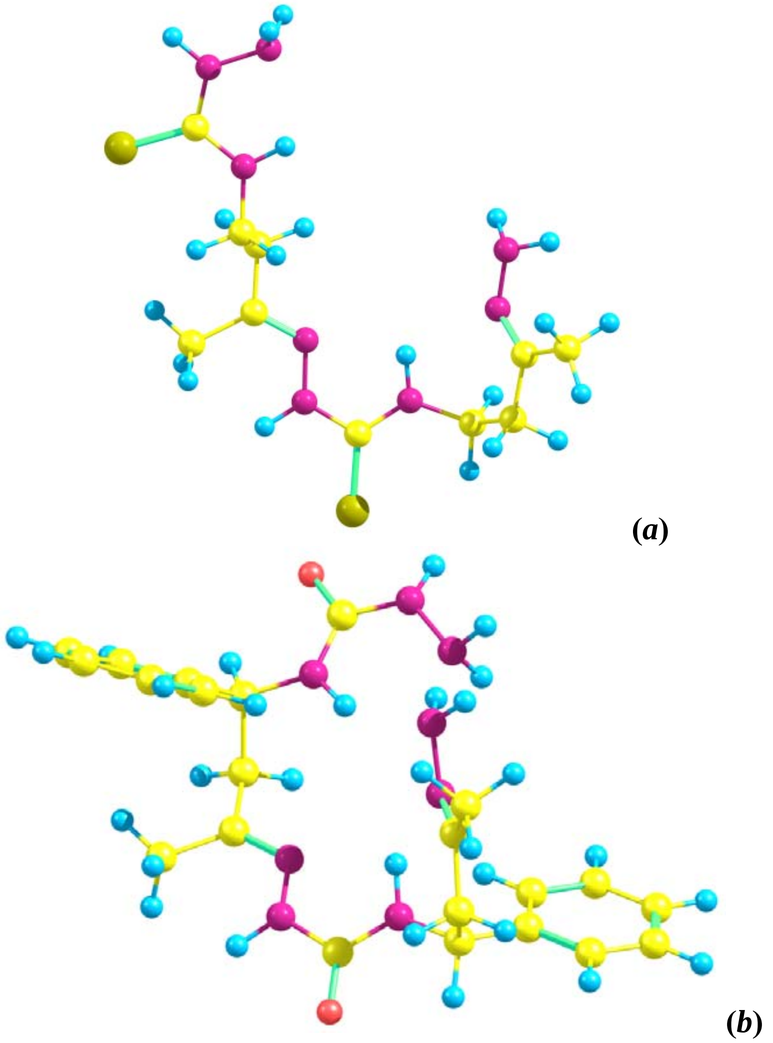

**Figure S8.** The most stable conformations of dimers (*E,E*)-**13a** (***a***) and (*E,E*)-**A** (R = Ph) (***b***).

It is noteworthy that the carbon of the hydrazone fragment in (*E,E*)-**A** (R = Ph) is strongly hindered by the bulky phenyl groups. The amino group of the semicarbazide moiety is also rather hindered. Thus, the dimer **A** (two molecules) → tetramer **B** conversion (Scheme 4) requires a significant change in the conformation of the dimer, which is hindered by their conformational rigidity due to the presence of the phenyl groups and strong hydrogen bonds.

We believe that it is the low rate of the dimer **A** → tetramer **B** conversion that is responsible for the formation of exclusively 14-membered macrocycles **3** upon the acid-promoted transformation of hydrazones of 4-(1-aryl-3-oxobut-1-yl)semicarbazides **1**.

## References and notes


1. X-ray diffraction experiments were performed at the Center for Shared Use of Physical Methods of Investigation at Frumkin Institute of Physical Chemistry and Electrochemistry, RAS.
2. SAINT-Plus (Version 7.68). // Bruker AXS Inc., Madison, Wisconsin, USA (2007).
3. Sheldrick, G. M.: SADABS. // Bruker AXS Inc., Madison, Wisconsin, USA (2008).
4. Sheldrick, G. M. A short history of SHELX // *Acta Crystallogr. Sect. A: Foundations of Crystallography. International Union of Crystallography*, 2008. Vol. 64, No 1, P. 112–122.
5. Sheldrick, G.M. Crystal structure refinement with SHELXL // *Acta Crystallogr. Sect. C Struct. Chem. International Union of Crystallography*, 2015. Vol. 71, No 1, P. 3–8.
6. *Gaussian 16, Revision A.03*, M. J. Frisch, G. W. Trucks, H. B. Schlegel, G. E. Scuseria, M. A. Robb, J. R. Cheeseman, G. Scalmani, V. Barone, G. A. Petersson, H. Nakatsuji, X. Li, M. Caricato, A. V. Marenich, J. Bloino, B. G. Janesko, R. Gomperts, B. Mennucci, H. P. Hratchian, J. V. Ortiz, A. F. Izmaylov, J. L. Sonnenberg, D. Williams-Young, F. Ding, F. Lipparini, F. Egidi, J. Goings, B. Peng, A. Petrone, T. Henderson, D. Ranasinghe, V. G. Zakrzewski, J. Gao, N. Rega, G. Zheng, W. Liang, M. Hada, M. Ehara, K. Toyota, R. Fukuda, J. Hasegawa, M. Ishida, T. Nakajima, Y. Honda, O. Kitao, H. Nakai, T. Vreven, K. Throssell, J. A. Montgomery, Jr., J. E. Peralta, F. Ogliaro, M. J. Bearpark, J. J. Heyd, E. N. Brothers, K. N. Kudin, V. N. Staroverov, T. A. Keith, R. Kobayashi, J. Normand, K. Raghavachari, A. P. Rendell, J. C. Burant, S. S. Iyengar, J. Tomasi, M. Cossi, J. M. Millam, M. Klene, C. Adamo, R. Cammi, J. W. Ochterski, R. L. Martin, K. Morokuma, O. Farkas, J. B. Foresman, and D. J. Fox, Gaussian, Inc., Wallingford CT, 2016.
7. (a) Fesenko, A. A.; Shutalev, A. D. *Tetrahedron* **2015**, *71*, 9528–9543. (b) Shutalev, A. D.; Fesenko, A. A.; Yankov, A. N.; Tafeenko, V. A.; Chernyshev, V. V. *J. Mol. Struct.* **2017**, *1150*, 349–357.
